# Supplementary material for: Quantifying and controlling the proteolytic degradation of cell adhesion peptides
Source: bioRxiv. 2024 Apr 24:2024.04.19.590329. Preprint. [Version 1] doi: 10.1101/2024.04.19.590329 (PMC11071418; doi:10.1101/2024.04.19.590329)
Supplement: Supplement 1 [file media-1.pdf]

Supporting Information for the manuscript:
Quantifying and controlling the proteolytic degradation of cell adhesion peptides

Samuel J. Rozans, Abolfazl Salehi Moghaddam, Yingjie Wu, Kayleigh Atanasoff, Liliana Nino, Katelyn Dunne, E. Thomas Pashuck

Table of Contents:

Figure S1 - Degradation of soluble peptides cultured with cells on TCP. Pages 1-5.
Figure S2 - Comparison of peptide degradation by different hMSC donors. Pages 6-9.
Figure S3 - Comparison of peptide degradation by different hUVEC donors. Pages 10-13.
Figure S4. Comparison of peptide degradation by different PBMC donors. Pages 14-17.
Figure S5. Degradation of peptides at different concentrations. Pages 18-20.
Figure S6. Effect of different peptide sequences on non-specific degradation. Pages 21-23.
Figure S7. Degradation of soluble peptide libraries by cells in PEG hydrogels.Pages 24-29.
Figure S8. PEG conjugation to peptides slows degradation. Page 30.
Figure S9. Microscopy of cells growing in gels with different RGD presentations. Page 31.
Figure S10. Quantification of viability and proliferation in hydrogels containing different RGD sequences. Page 32.
Figure S11. Standard curves validating the use of LCMS to measure the concentration of peptides. Page 33.
Figure S12. LCMS spectra of the Ac-X-RGEFV-βA-NH_2 libraries. Pages 34-42.
Figure S13. LCMS spectra of the Ac-βA-X-RGEFV-βA-NH_2 libraries. Pages 43-51.
Figure S14. LCMS spectra of the Ac-βA-RGEFV-X-NH_2 libraries. Pages 52-60.
Figure S15. LCMS spectra of the Ac-βA-RGEFV-X-βA-NH_2 libraries. Pages 61-69.
Figure S16. LCMS spectra of the Ac-βA-RGEFV-X-COOH libraries. Pages 70-78.
Figure S17. LCMS spectra of the NH_2-βA-X-RGEFV-βA-NH_2 libraries. Pages 79-87.
Figure S18. LCMS spectra of the NH_2-X-RGEFV-βA-NH_2 libraries. Pages 88-96.
Figure S19. LCMS spectra of the peptides for the concentration studies. Pages 97-100.
Figure S20. LCMS spectra of the LIAANK peptides. Pages 100-103.
Figure S21. LCMS spectra of the IVKVA peptides. Pages 104-107.
Figure S22. LCMS spectra of the Azide and PEG modified peptides. Pages 107-114.
Figure S23. LCMS spectra of the peptides used for cell culture. Pages 115-117.
Figure S24. Statistical analyses. Pages 118-135.

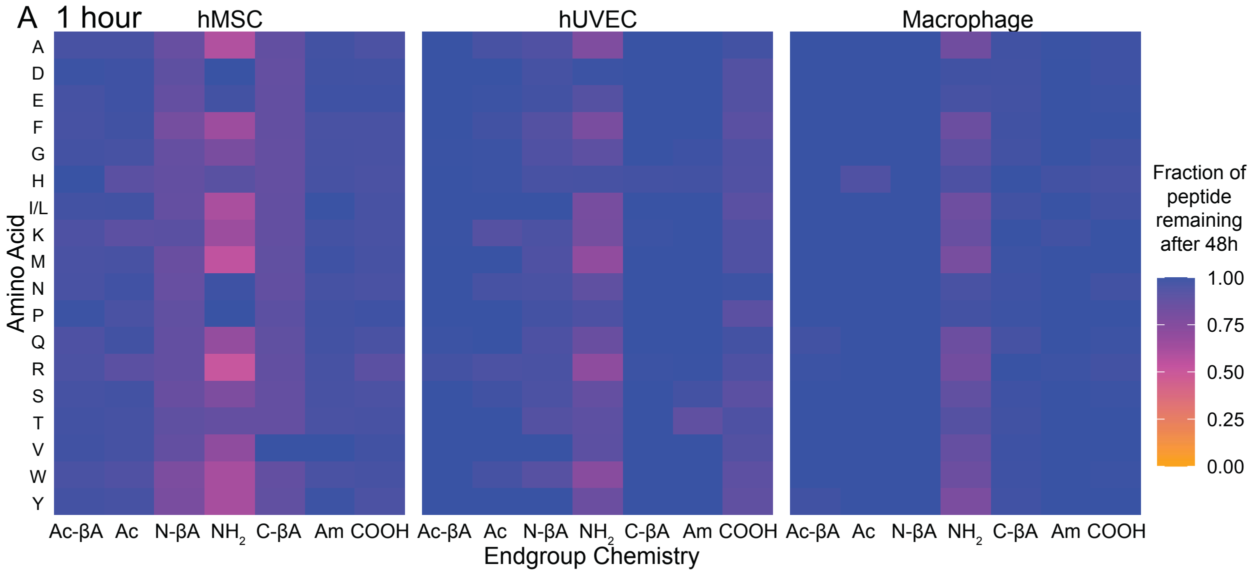

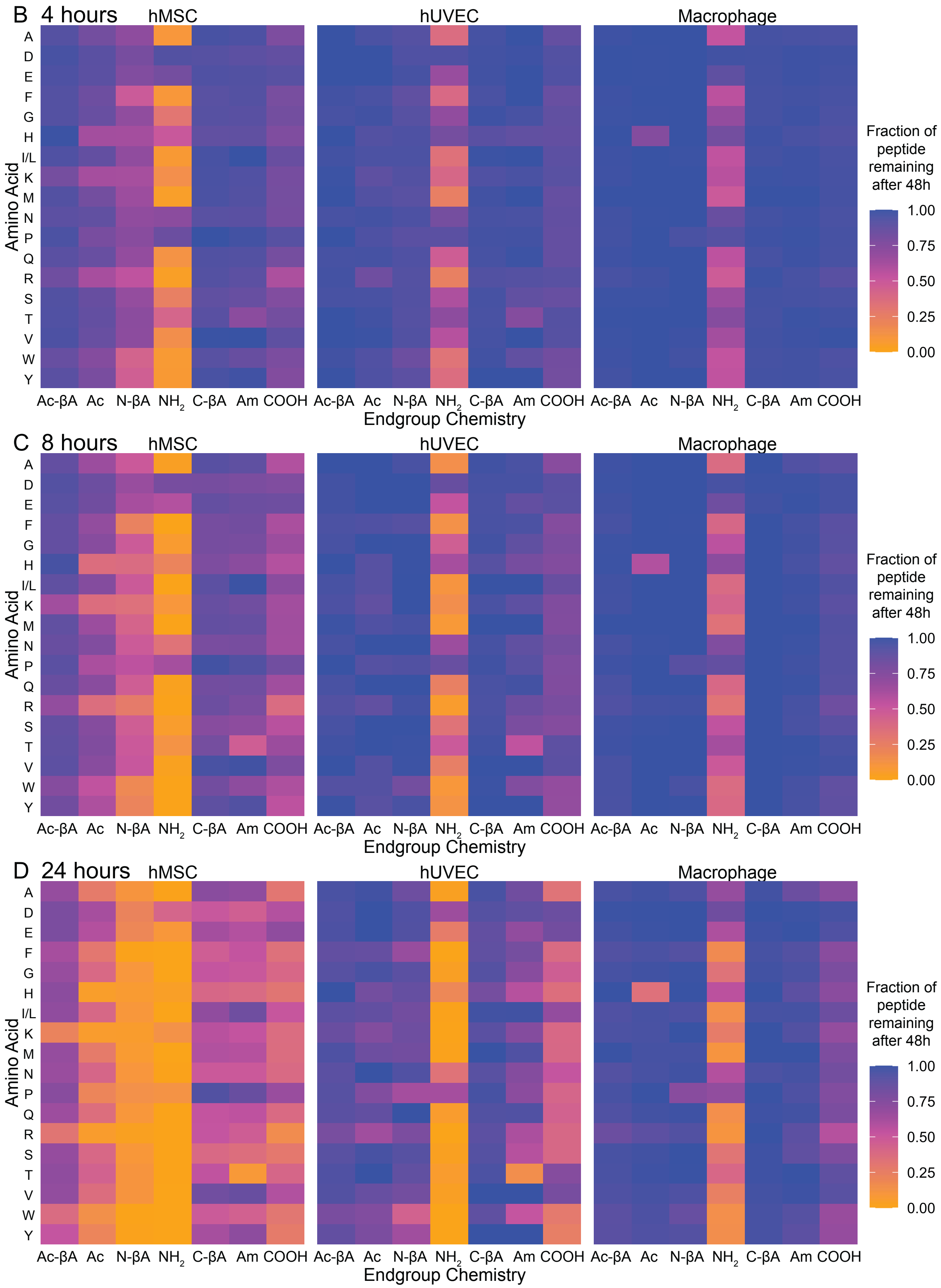
B 4 hours hMSC
hUVEC
Macrophage
C 8 hours hMSC
hUVEC
Macrophage
D 24 hours hMSC
hUVEC
Macrophage
Amino Acid
A
D
E
F
G
H
I/L
K
M
N
P
Q
R
S
T
V
W
Y
Ac-βA Ac N-βA NH2 C-βA Am COOH
Endgroup Chemistry
Fraction of peptide remaining after 48h
1.00
0.75
0.50
0.25
0.00

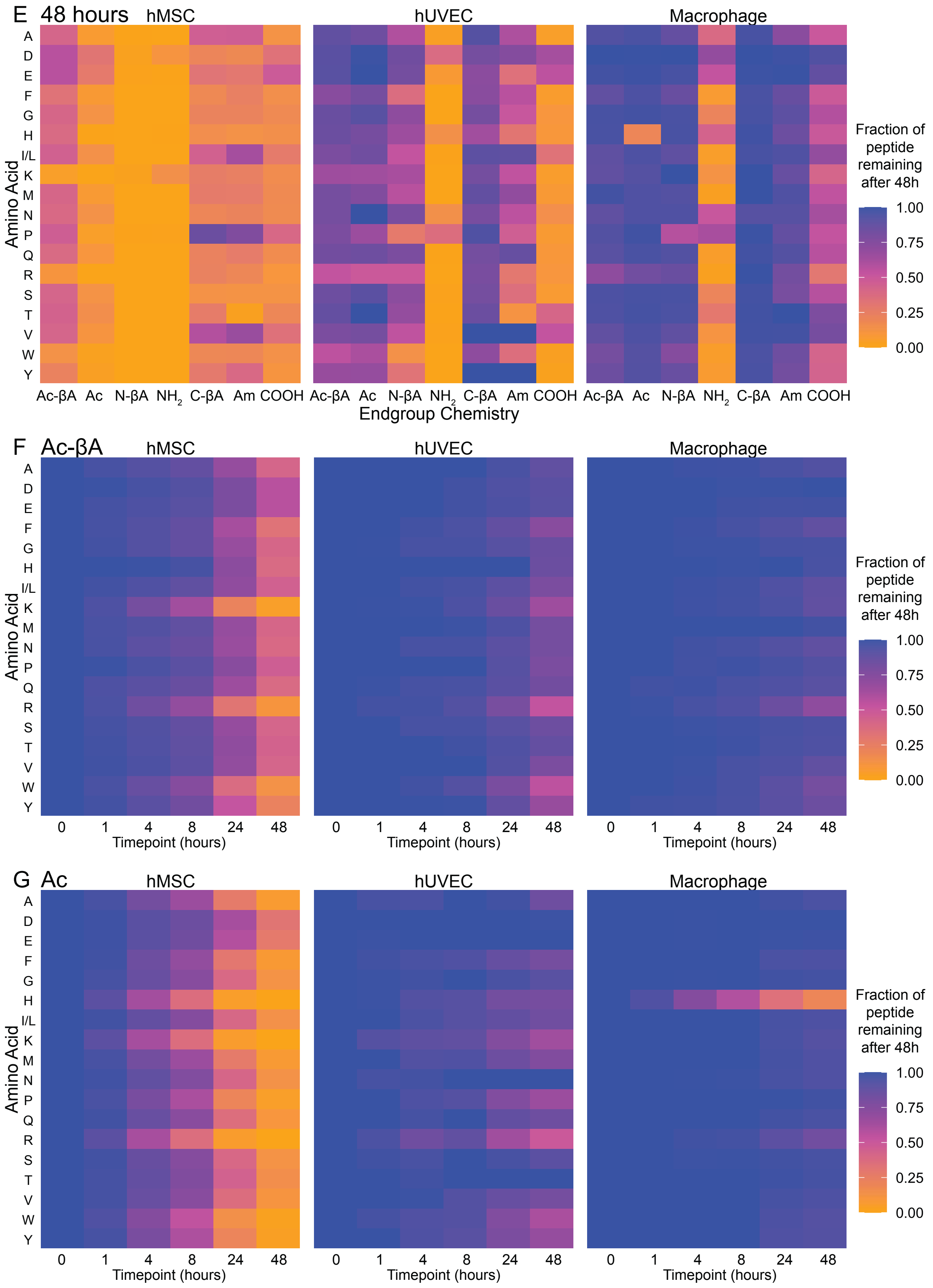
E 48 hours
hMSC
hUVEC
Macrophage
Amino Acid
A
D
E
F
G
H
I/L
K
M
N
P
Q
R
S
T
V
W
Y
Ac-βA Ac N-βA NH2 C-βA Am COOH
Endgroup Chemistry
Fraction of peptide remaining after 48h
1.00
0.75
0.50
0.25
0.00
F Ac-βA
hMSC
hUVEC
Macrophage
0 1 4 8 24 48
Timepoint (hours)
G Ac
hMSC
hUVEC
Macrophage
0 1 4 8 24 48
Timepoint (hours)

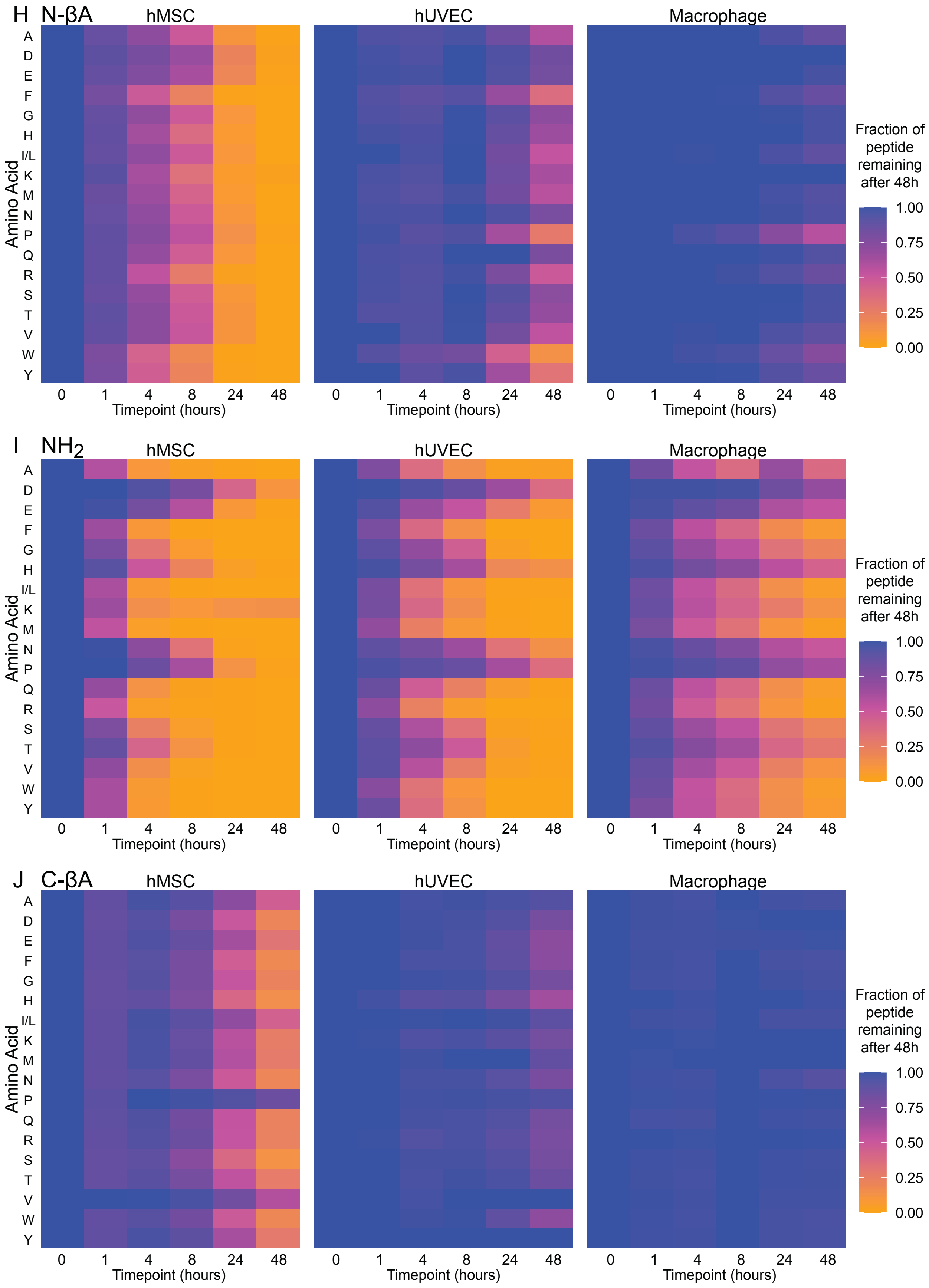
H N-βA
hMSC
hUVEC
Macrophage
Amino Acid
A
D
E
F
G
H
I/L
K
M
N
P
Q
R
S
T
V
W
Y
0 1 4 8 24 48
Timepoint (hours)
Fraction of peptide remaining after 48h
1.00
0.75
0.50
0.25
0.00
I NH2
J C-βA

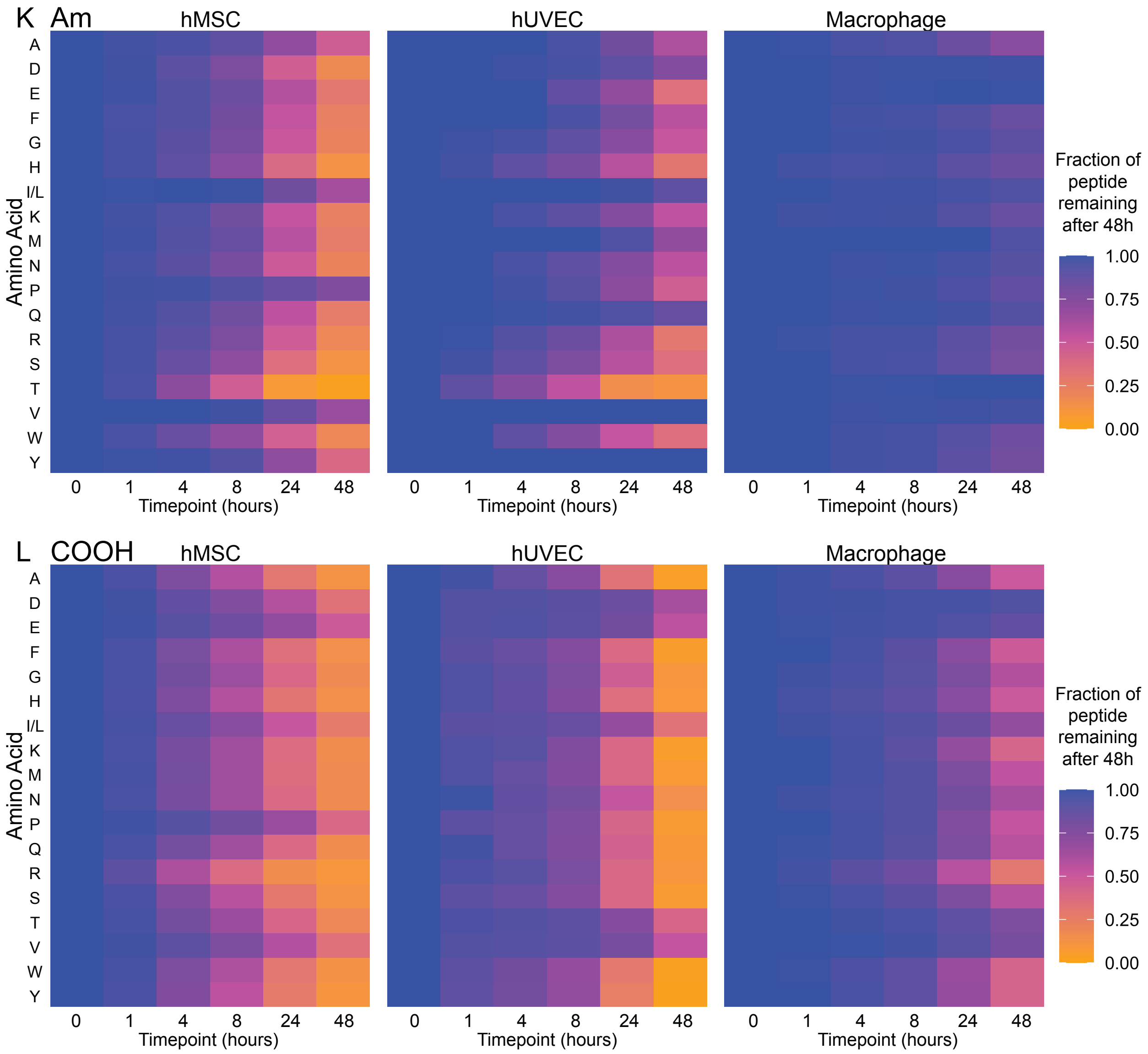

Figure S1. Degradation of soluble peptides cultured with cells on tissue culture plastic. Degradation was quantified at (**A**) 1 hour, (**B**) 4 hours, (**C**) 8 hours, (**D**) 24 hours and (**E**) 48 hours. Degradation was also quantified by the chemistry of the peptide termini, including (**F**) Ac-βA, (**G**) Ac, (**H**) N-βA, (**I**) NH_2, (**J**) C-βA, (**K**) Am, (**L**) COOH.

A hMSC: Ac-βA

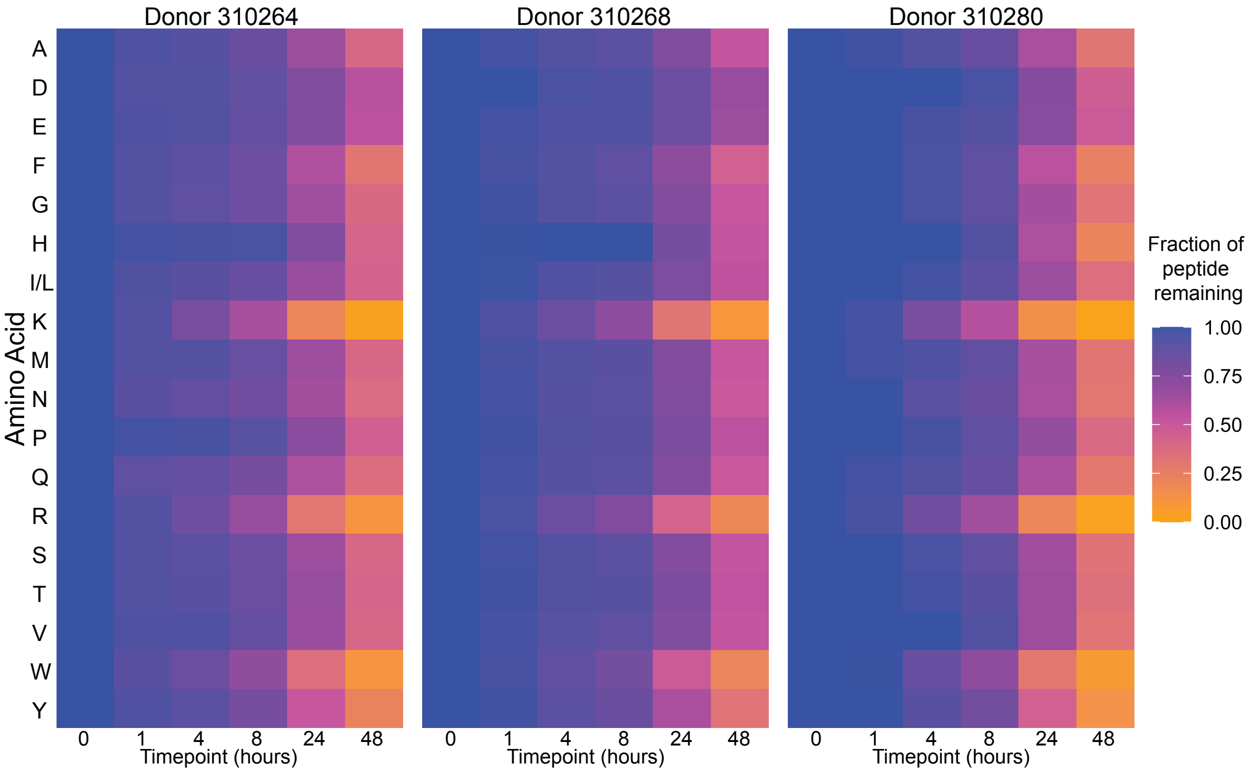

B hMSC: Ac

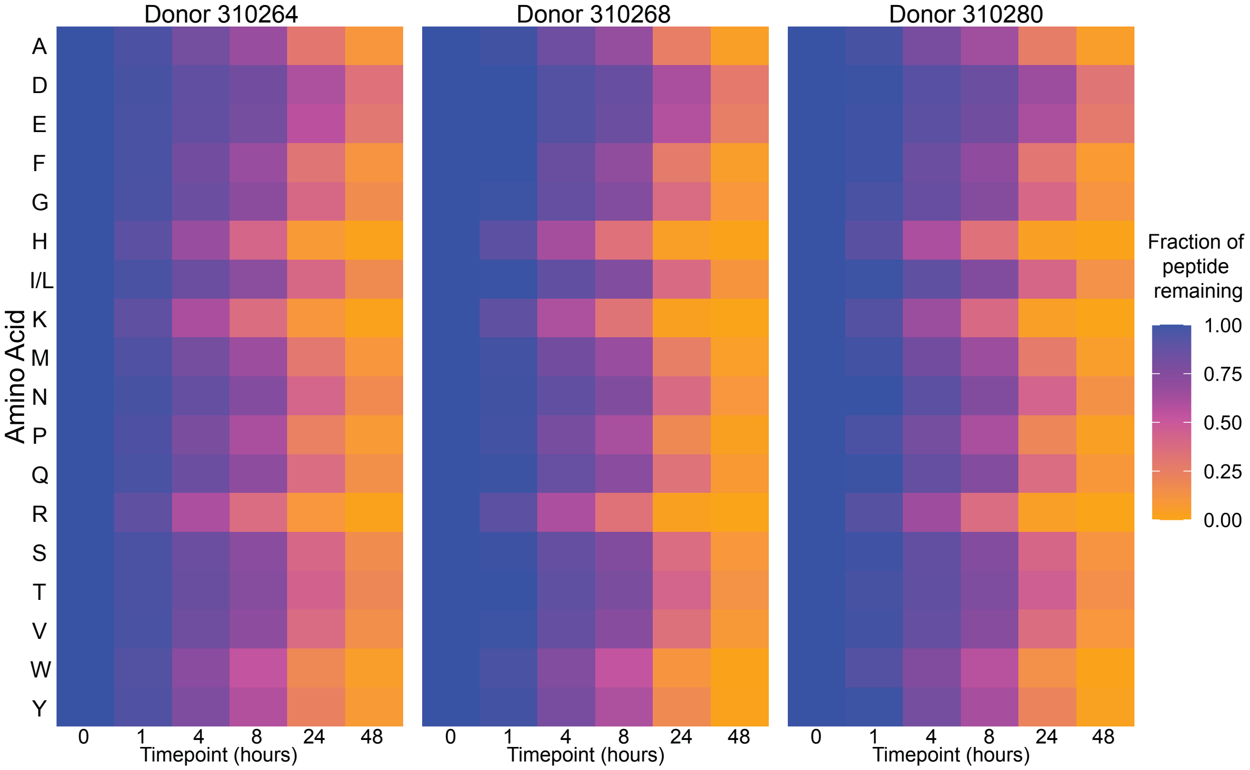

C hMSC: N-βA

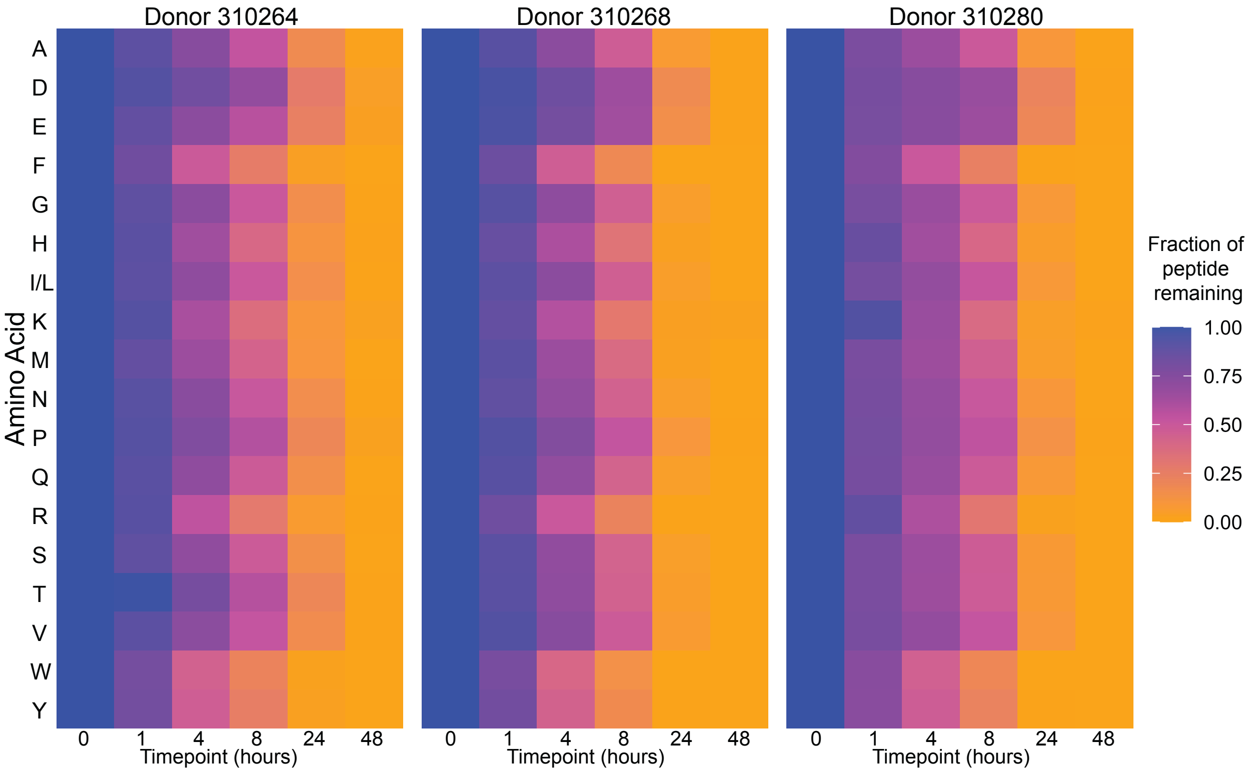

D hMSC: NH_2

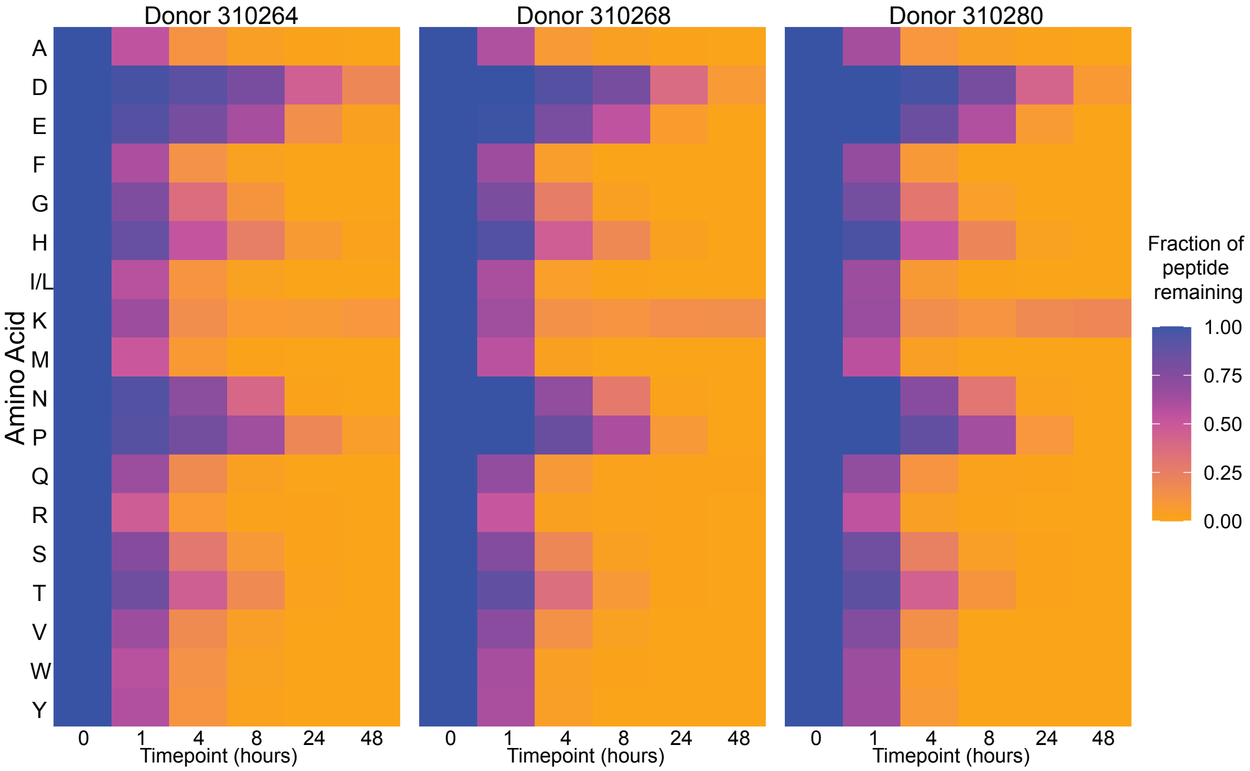

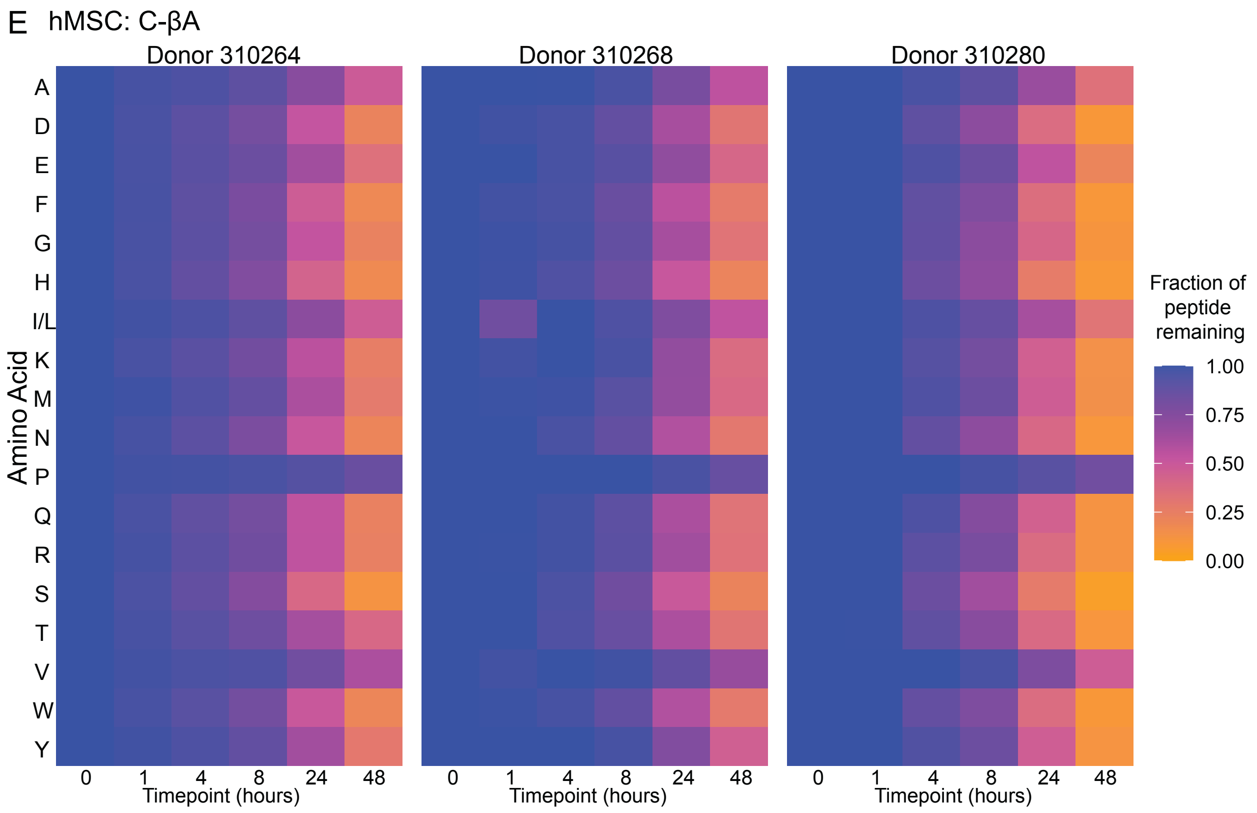
E hMSC: C-βA
Donor 310264
Donor 310268
Donor 310280
Amino Acid
A
D
E
F
G
H
I/L
K
M
N
P
Q
R
S
T
V
W
Y
0 1 4 8 24 48
Timepoint (hours)
Fraction of peptide remaining
1.00
0.75
0.50
0.25
0.00

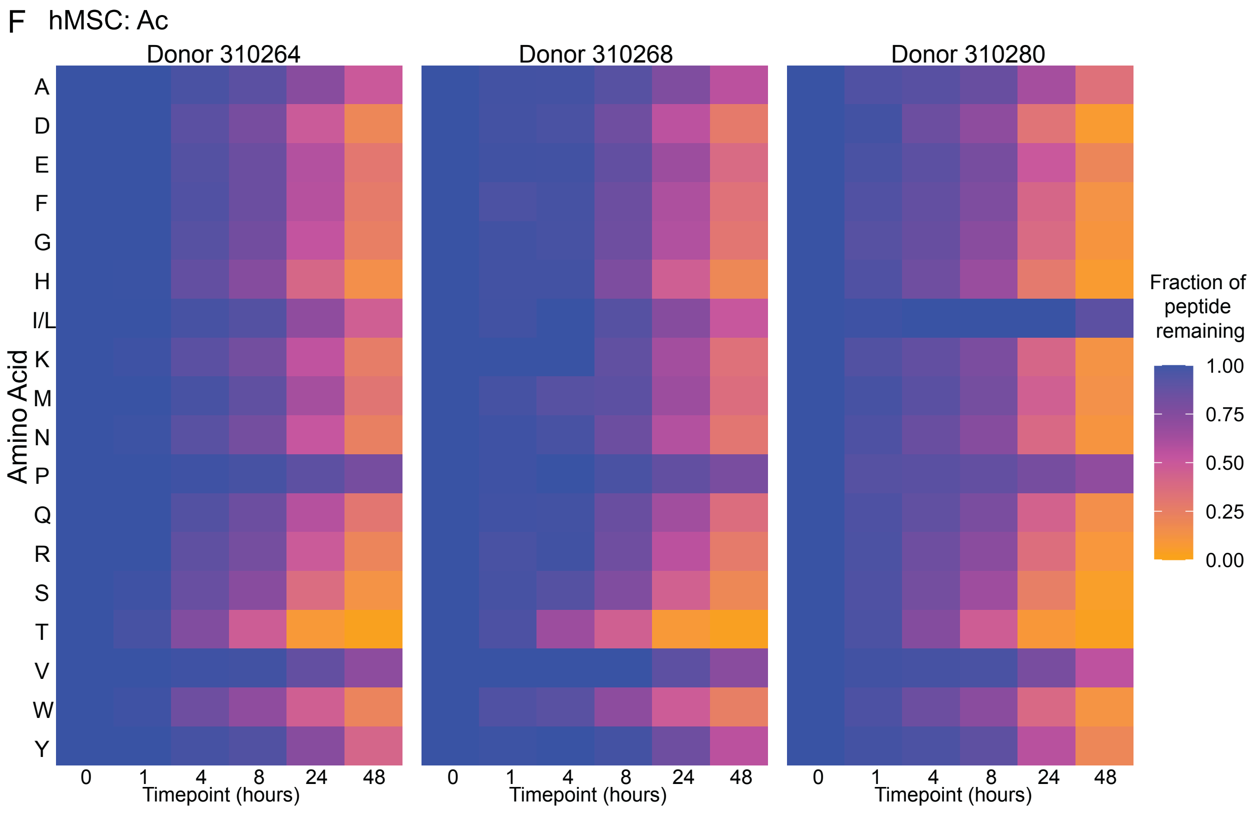
F hMSC: Ac
Donor 310264
Donor 310268
Donor 310280
Amino Acid
A
D
E
F
G
H
I/L
K
M
N
P
Q
R
S
T
V
W
Y
0 1 4 8 24 48
Timepoint (hours)
Fraction of peptide remaining
1.00
0.75
0.50
0.25
0.00

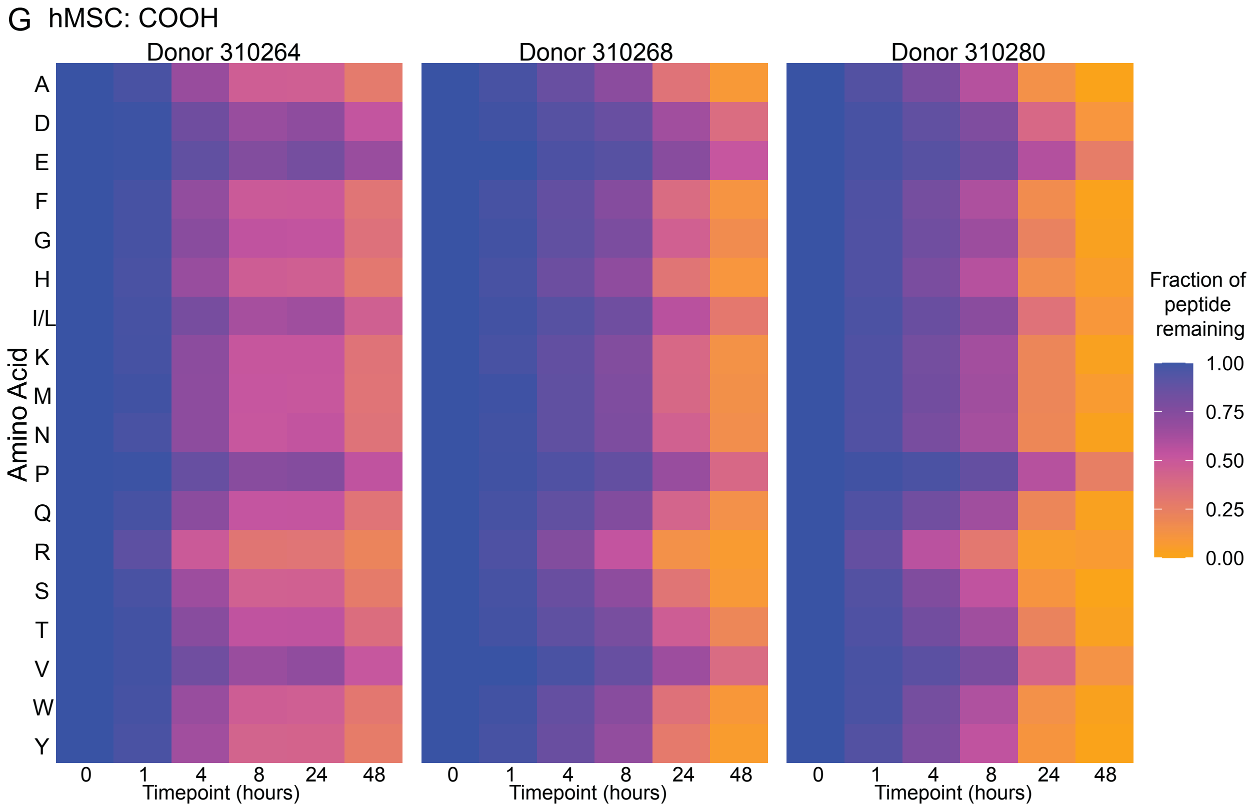

Figure S2. Comparison of peptide degradation by different hMSC donors. hMSCs were cultured with (**A**) Ac-βA, (**B**) Ac, (**C**) N-βA, (**D**) NH_2, (**E**) C-βA, (**F**) Am, (**G**) COOH.

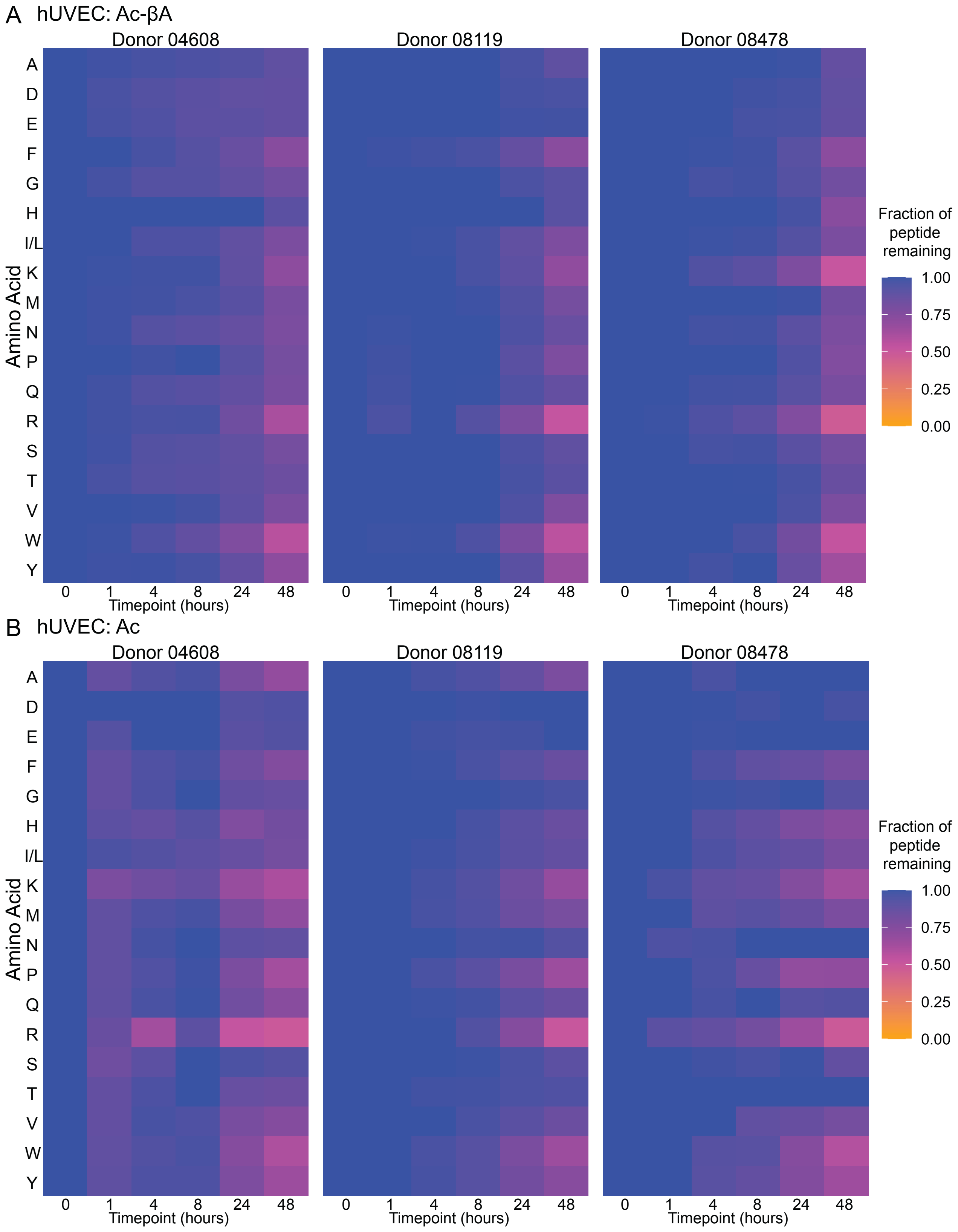

A hUVEC: Ac-βA
Donor 04608
Donor 08119
Donor 08478
A
D
E
F
G
H
I/L
K
M
N
P
Q
R
S
T
V
W
Y
Amino Acid
0 1 4 8 24 48
Timepoint (hours)
Fraction of peptide remaining
1.00
0.75
0.50
0.25
0.00
B hUVEC: Ac
Donor 04608
Donor 08119
Donor 08478
Amino Acid
Timepoint (hours)
Fraction of peptide remaining

C hUVEC: N-βA

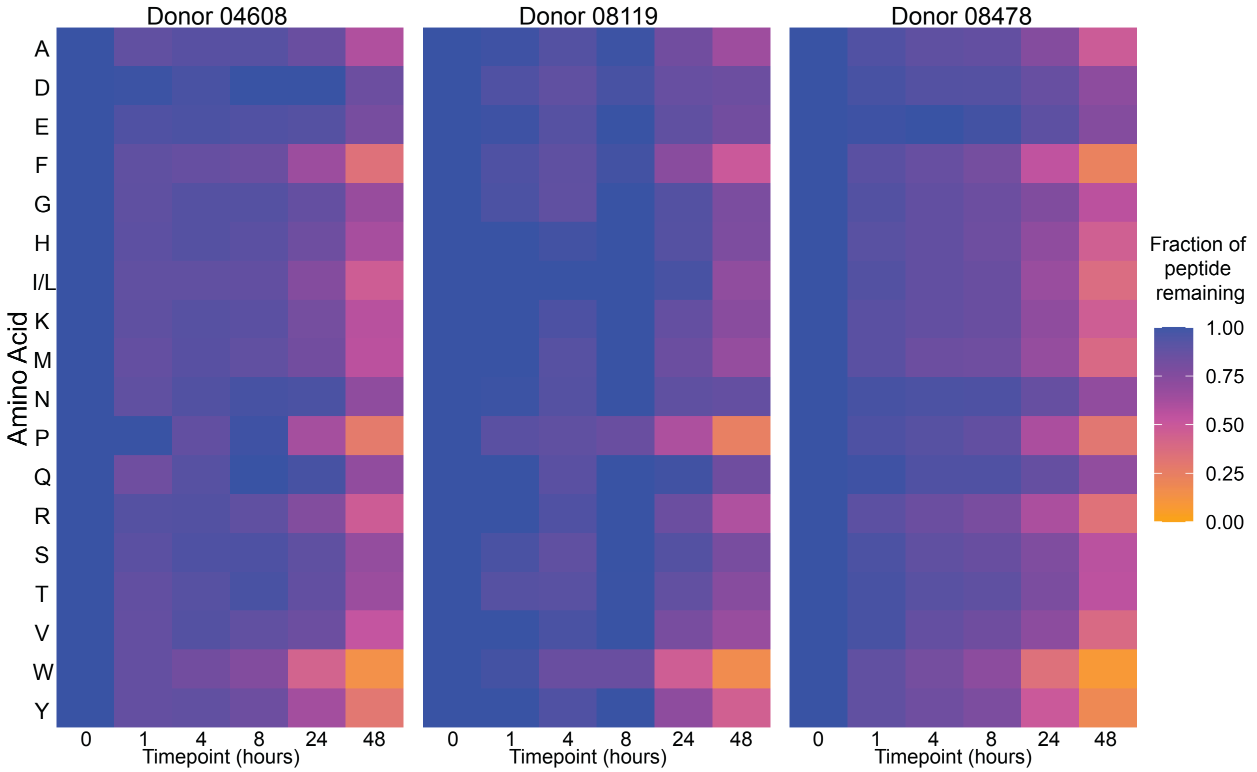

D hUVEC: NH_2

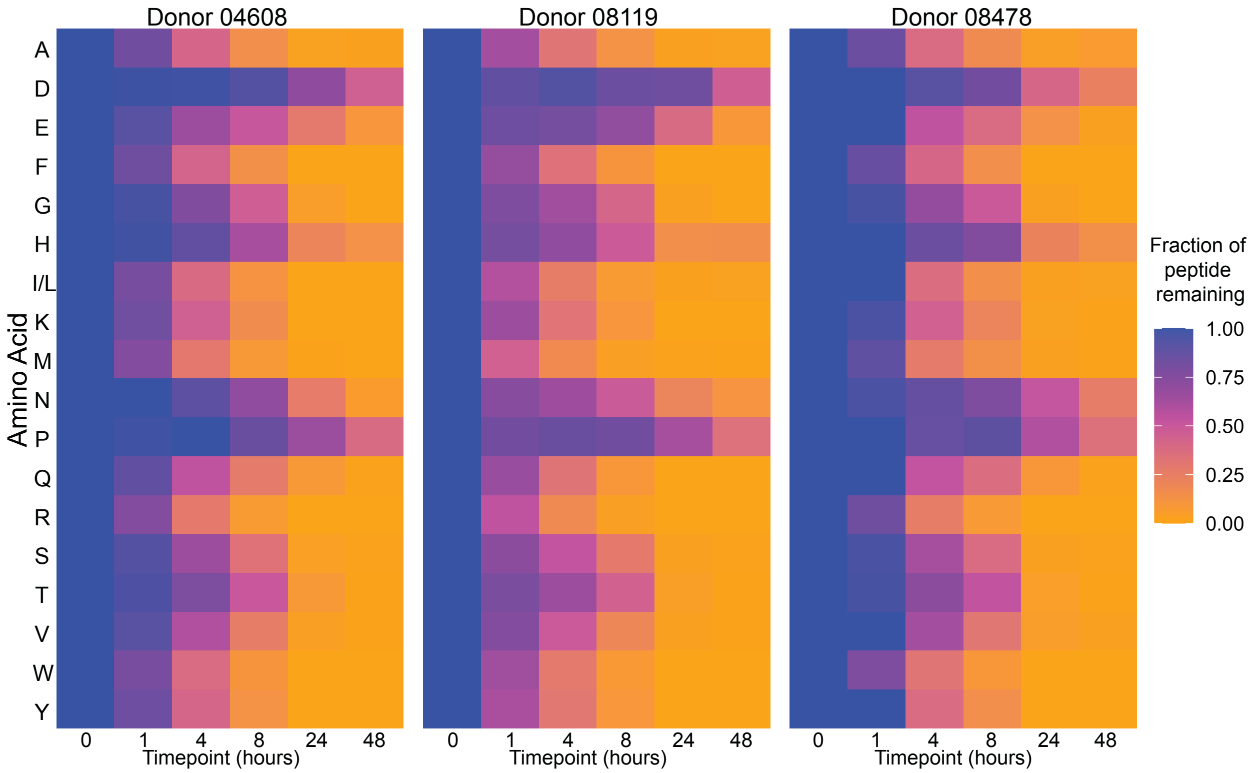

E hUVEC: C-βA

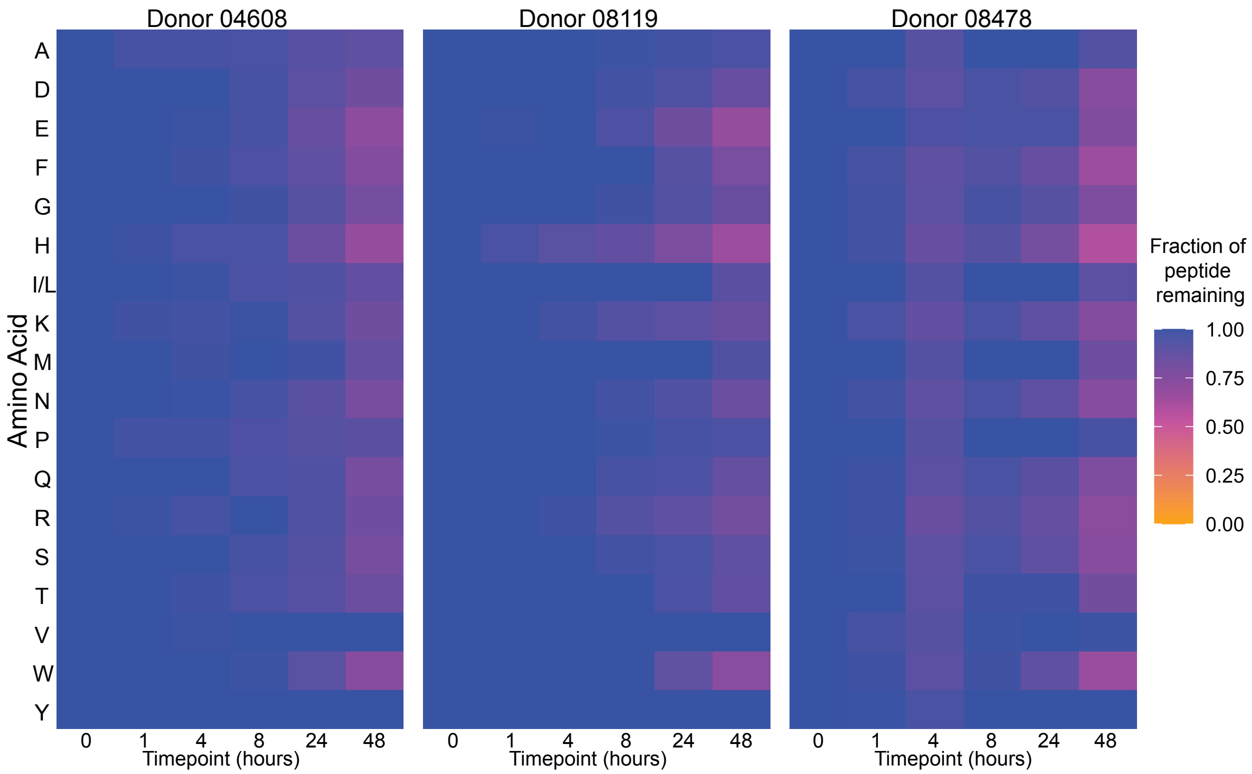

F hUVEC: Am

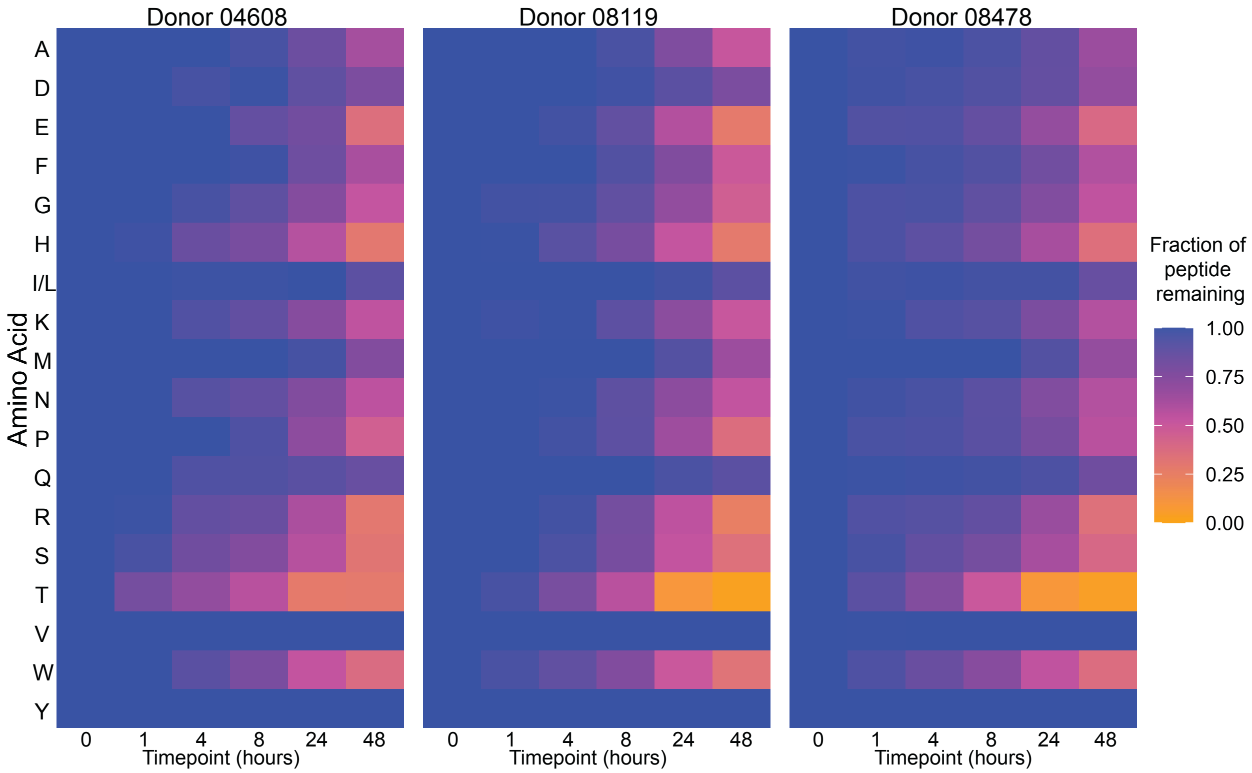

G hUVEC: COOH

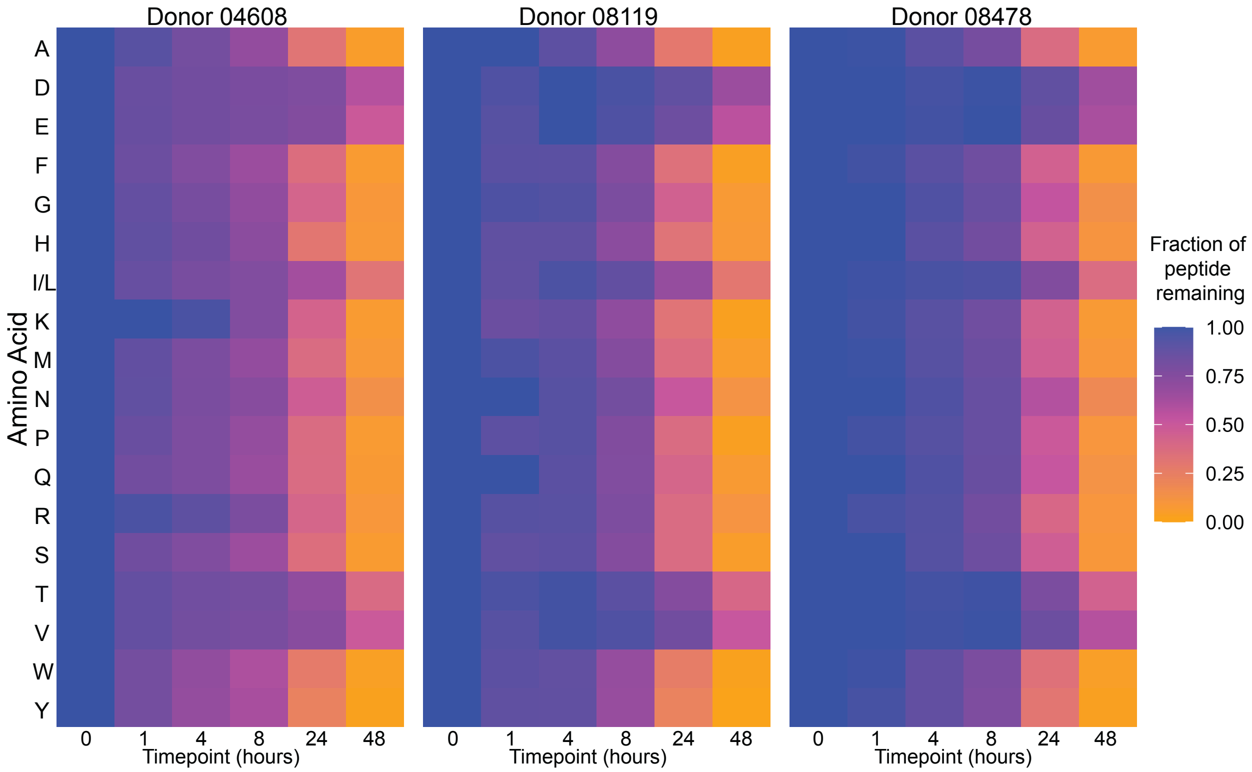

Figure S3. Comparison of peptide degradation by different hUVEC donors. hUVECs were cultured with (**A**) Ac-βA, (**B**) Ac, (**C**) N-βA, (**D**) NH_2, (**E**) C-βA, (**F**) Am, (**G**) COOH.

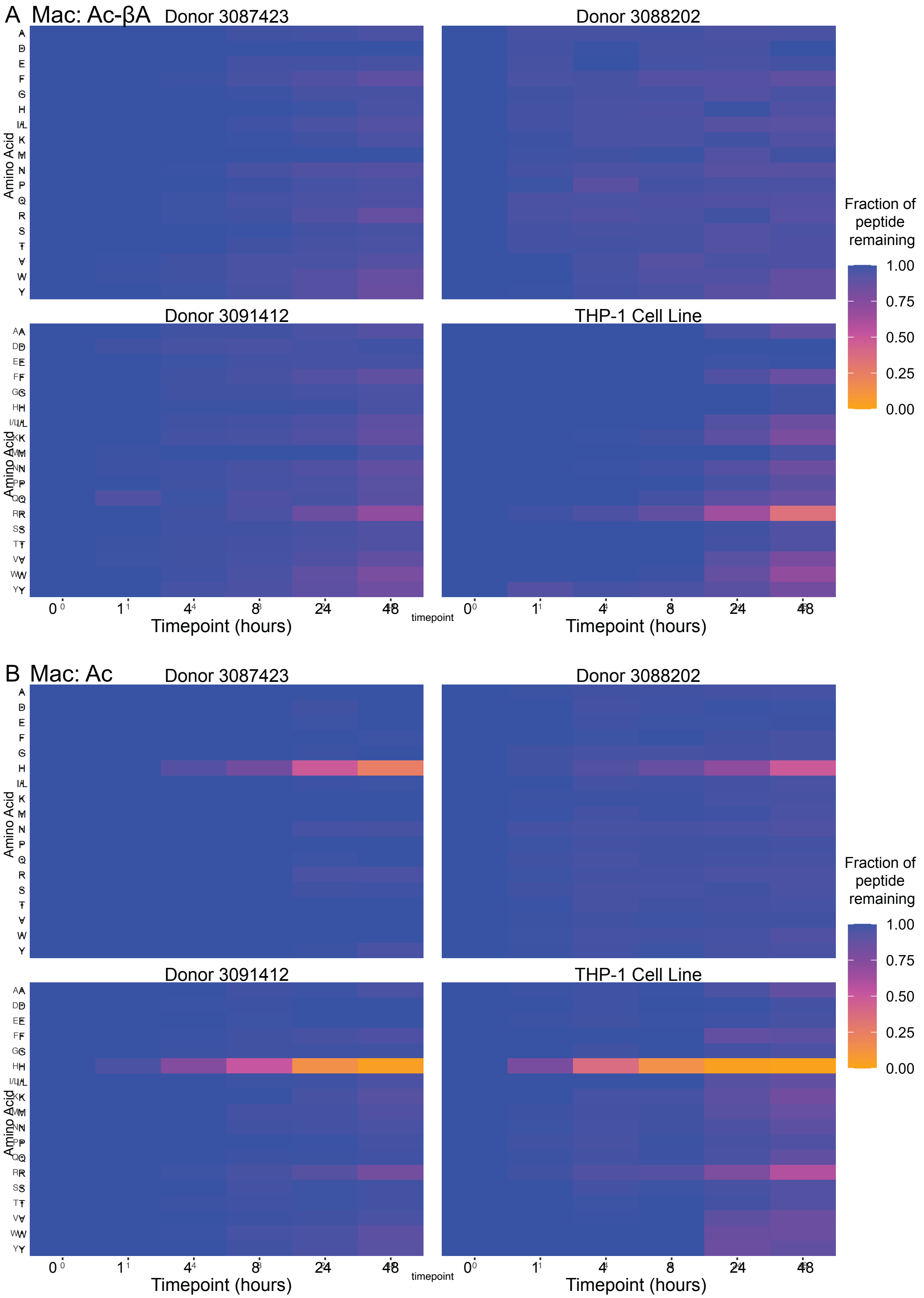
A Mac: Ac-βA
Donor 3087423
Donor 3088202
Donor 3091412
THP-1 Cell Line
Amino Acid
A
D
E
F
G
H
I/L
K
M
N
P
Q
R
S
T
V
W
Y
0
1
4
8
24
48
Timepoint (hours)
timepoint
Fraction of peptide remaining
1.00
0.75
0.50
0.25
0.00
B Mac: Ac
Donor 3087423
Donor 3088202
Donor 3091412
THP-1 Cell Line
Amino Acid
Timepoint (hours)
timepoint
Fraction of peptide remaining
1.00
0.75
0.50
0.25
0.00

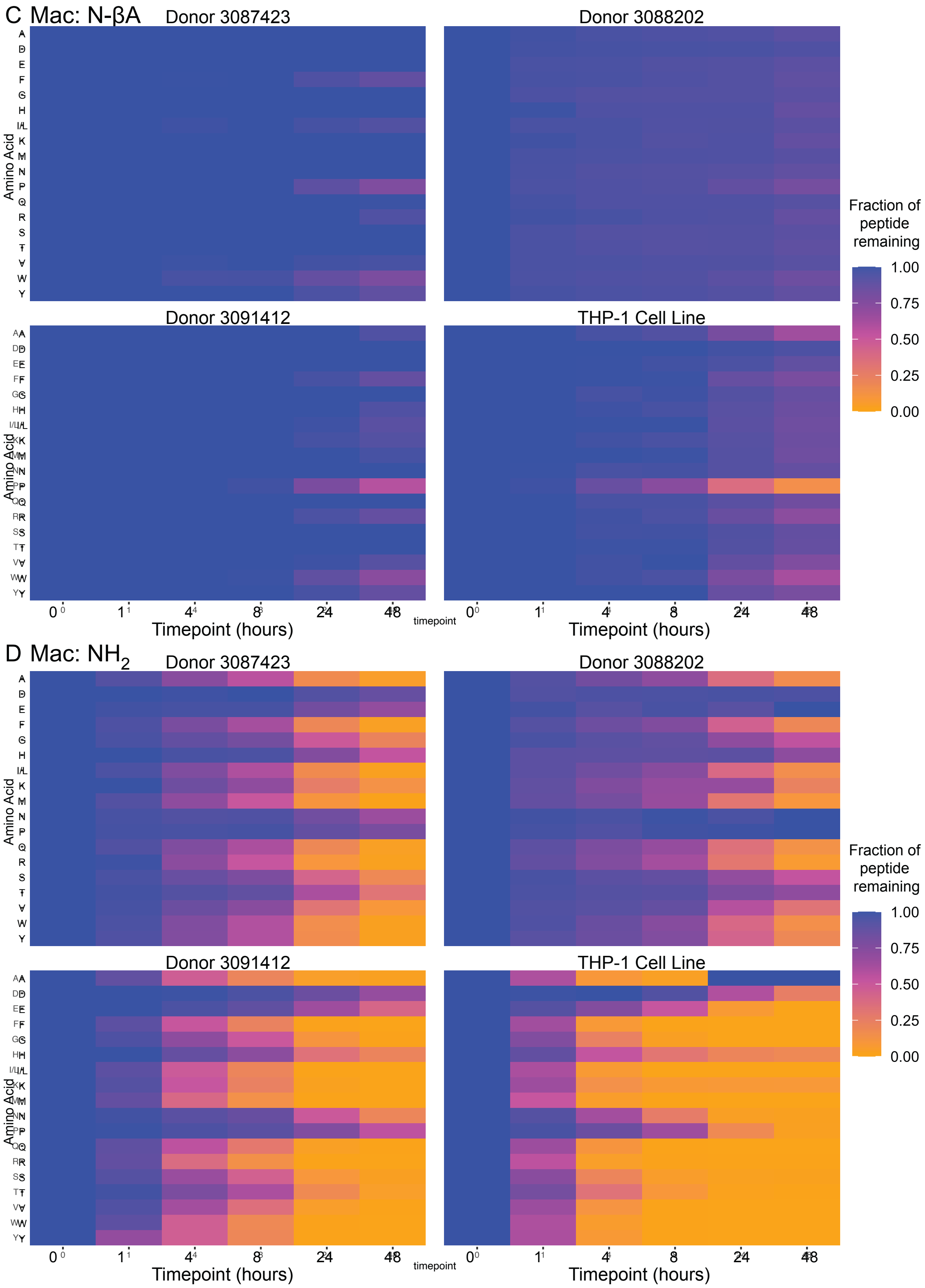
C Mac: N-βA
Donor 3087423
Donor 3088202
Donor 3091412
THP-1 Cell Line
Amino Acid
A
D
E
F
G
H
I/L
K
M
N
P
Q
R
S
T
V
W
Y
Fraction of peptide remaining
1.00
0.75
0.50
0.25
0.00
0
1
4
8
24
48
Timepoint (hours)
D Mac: NH2
Donor 3087423
Donor 3088202
Donor 3091412
THP-1 Cell Line
Amino Acid
Fraction of peptide remaining
Timepoint (hours)

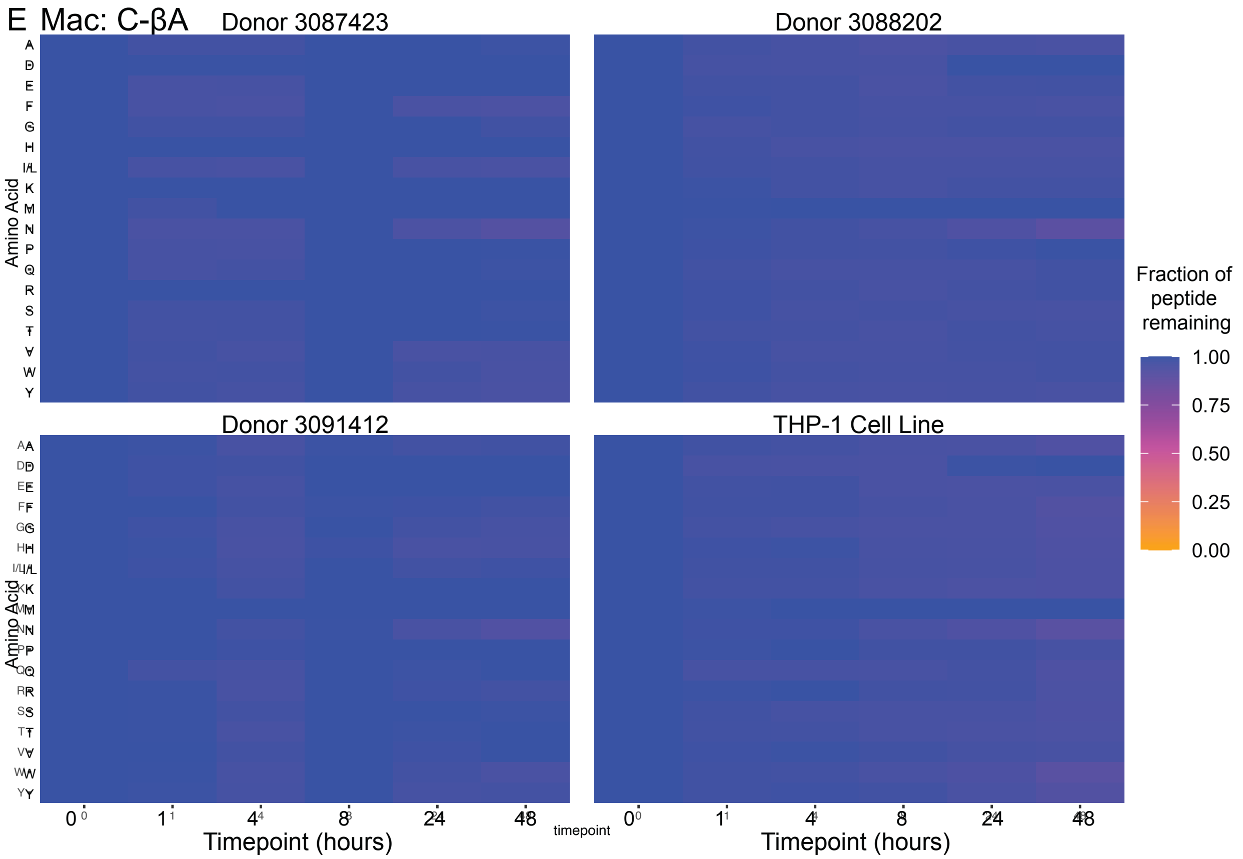
E Mac: C-βA
Donor 3087423
Donor 3088202
Donor 3091412
THP-1 Cell Line
Amino Acid
A
D
E
F
G
H
I/L
K
M
N
P
Q
R
S
T
V
W
Y
Fraction of peptide remaining
1.00
0.75
0.50
0.25
0.00
0
1
4
8
24
48
timepoint
Timepoint (hours)

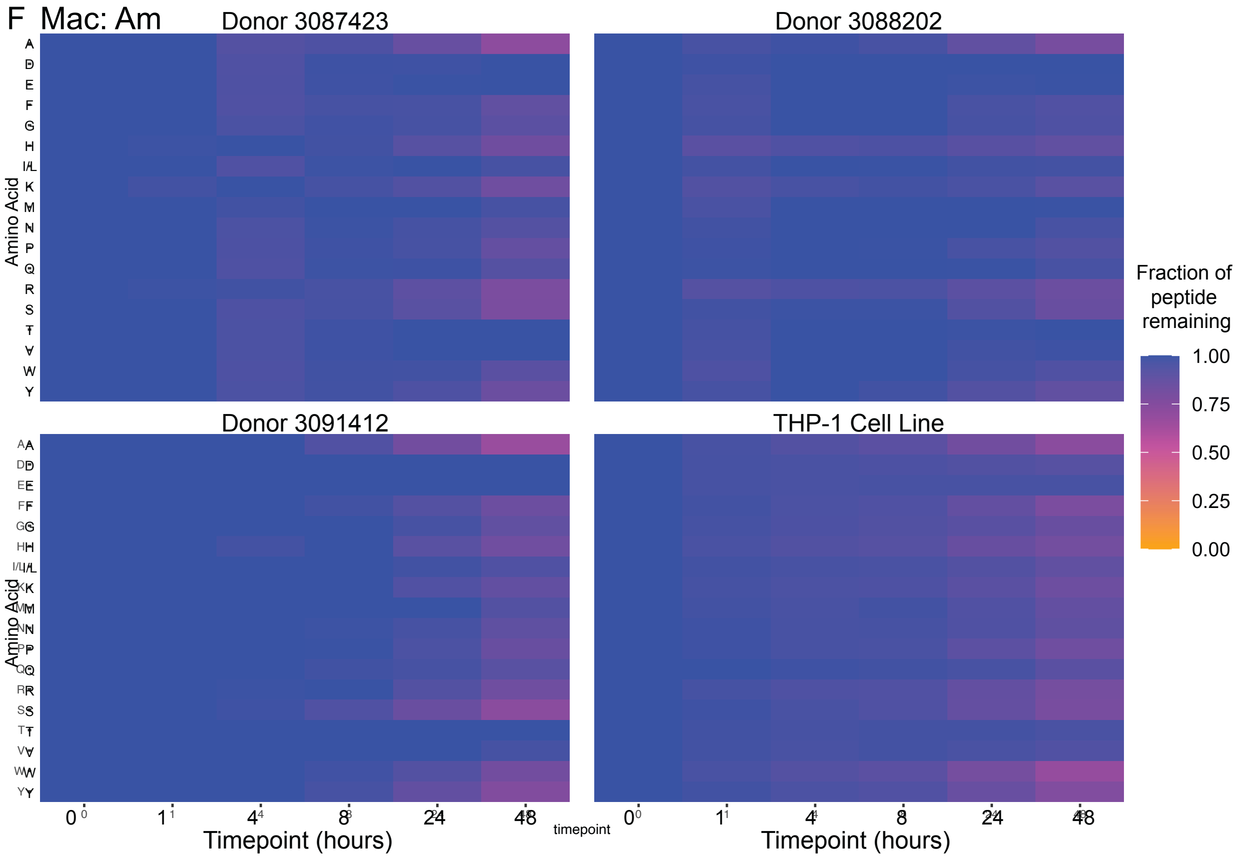
F Mac: Am
Donor 3087423
Donor 3088202
Donor 3091412
THP-1 Cell Line
Amino Acid
A
D
E
F
G
H
I/L
K
M
N
P
Q
R
S
T
V
W
Y
Fraction of peptide remaining
1.00
0.75
0.50
0.25
0.00
0
1
4
8
24
48
timepoint
Timepoint (hours)

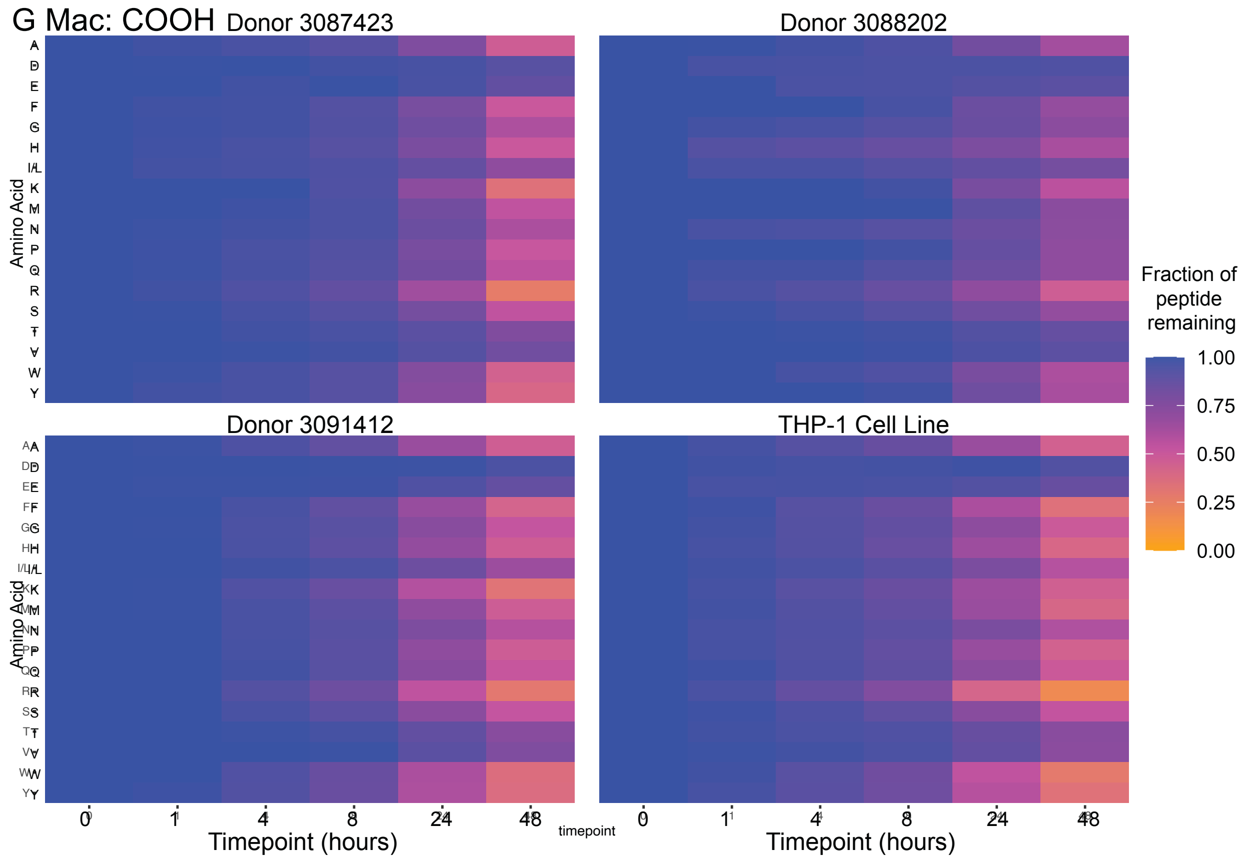

Figure S4. Comparison of peptide degradation by different donors. Macrophages were cultured with (**A**) Ac-βA, (**B**) Ac, (**C**) N-βA, (**D**) NH_2, (**E**) C-βA, (**F**) Am, (**G**) COOH.

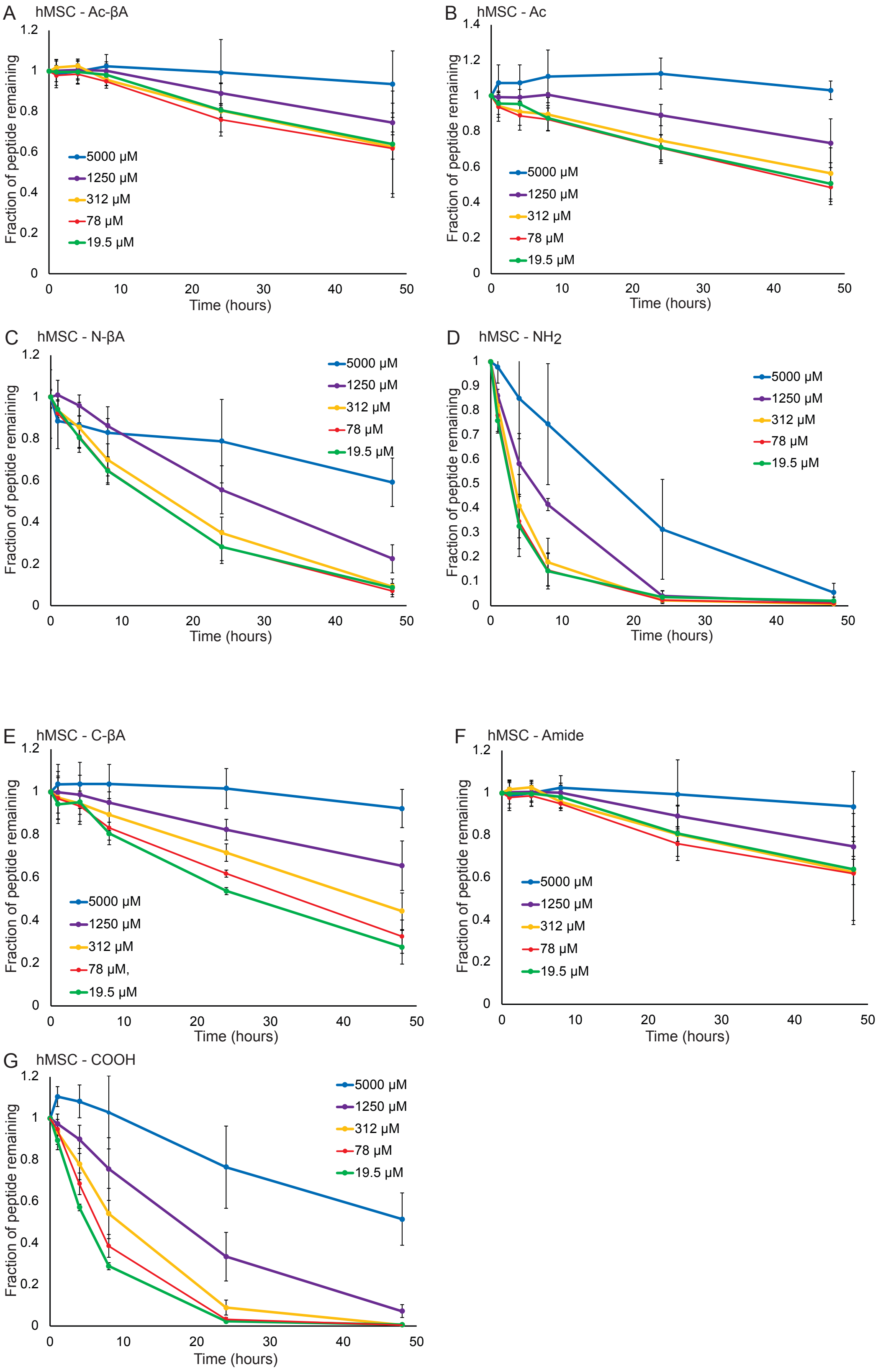
A
hMSC - Ac-βA
B
hMSC - Ac
C
hMSC - N-βA
D
hMSC - NH2
E
hMSC - C-βA
F
hMSC - Amide
G
hMSC - COOH
Fraction of peptide remaining
Time (hours)
5000 μM
1250 μM
312 μM
78 μM
78 μM,
19.5 μM

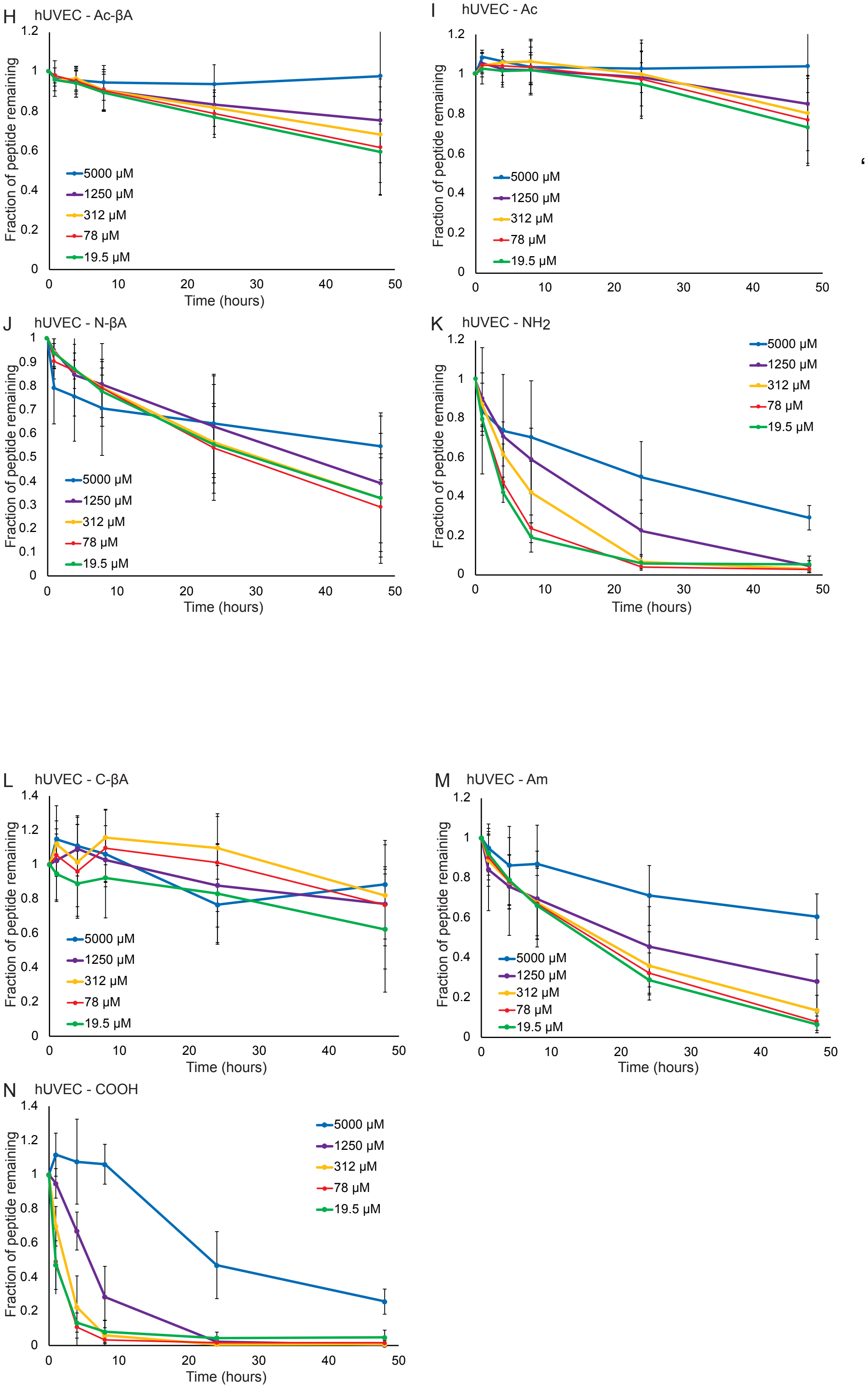
H
hUVEC - Ac-βA
Fraction of peptide remaining
Time (hours)
5000 µM
1250 µM
312 µM
78 µM
19.5 µM
I
hUVEC - Ac
Fraction of peptide remaining
Time (hours)
5000 µM
1250 µM
312 µM
78 µM
19.5 µM
J
hUVEC - N-βA
Fraction of peptide remaining
Time (hours)
5000 µM
1250 µM
312 µM
78 µM
19.5 µM
K
hUVEC - NH2
Fraction of peptide remaining
Time (hours)
5000 µM
1250 µM
312 µM
78 µM
19.5 µM
L
hUVEC - C-βA
Fraction of peptide remaining
Time (hours)
5000 µM
1250 µM
312 µM
78 µM
19.5 µM
M
hUVEC - Am
Fraction of peptide remaining
Time (hours)
5000 µM
1250 µM
312 µM
78 µM
19.5 µM
N
hUVEC - COOH
Fraction of peptide remaining
Time (hours)
5000 µM
1250 µM
312 µM
78 µM
19.5 µM

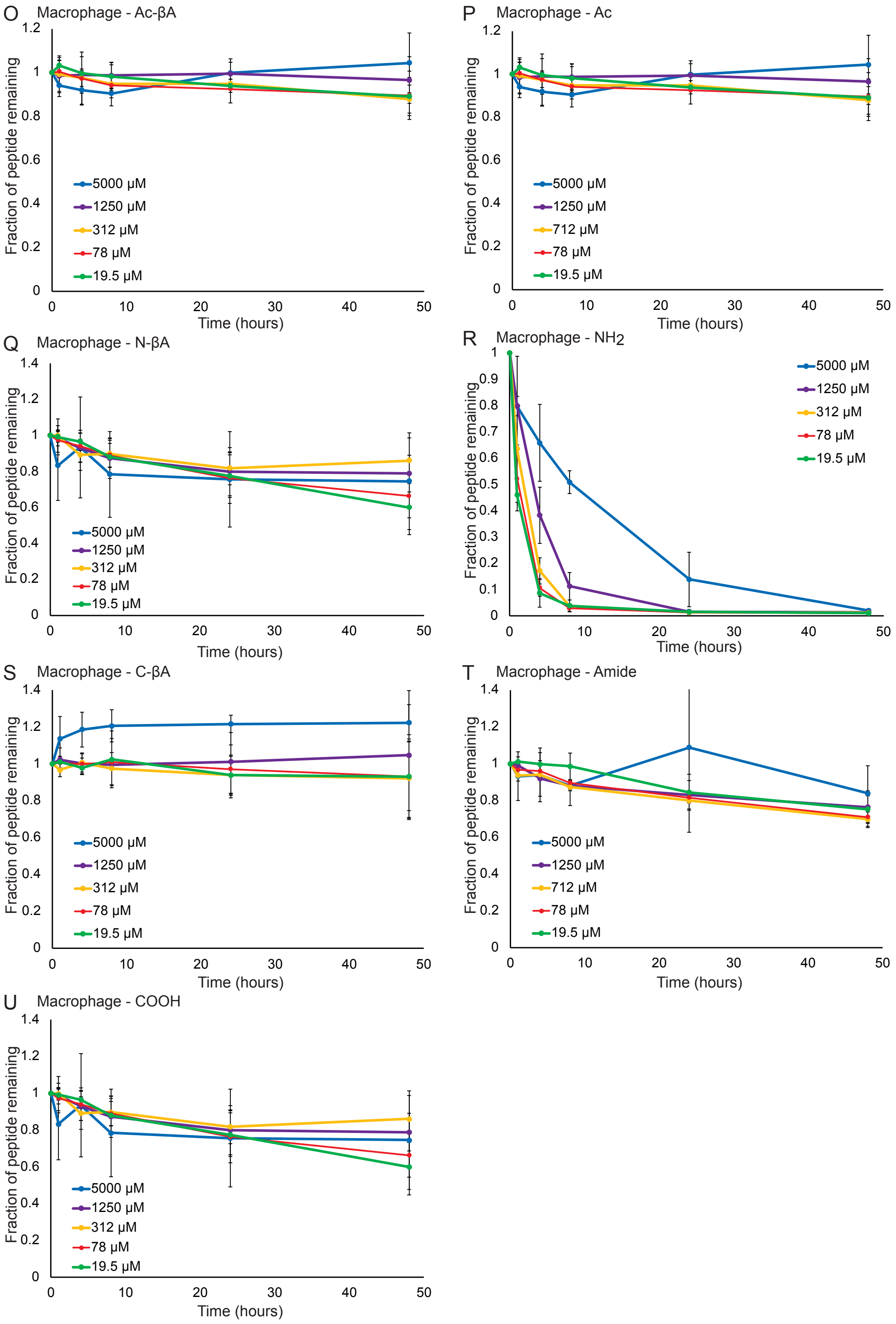

Figure S5. Degradation of peptides at different concentrations. Data was quantified for hMSCs (**A**) Ac-βA, (**B**) Ac, (**C**) N-βA, (**D**) NH_2, (**E**) C-βA, (**F**) Am, (**G**) COOH, hUVECs (**H**) Ac-βA, (**I**) Ac, (**J**) N-βA, (**K**) NH_2, (**L**) C-βA, (**M**) Am, (**N**) COOH, and macrophages (**O**) Ac-βA, (**P**) Ac, (**Q**) N-βA, (**R**) NH_2, (**S**) C-βA, (**T**) Am, (**U**) COOH.

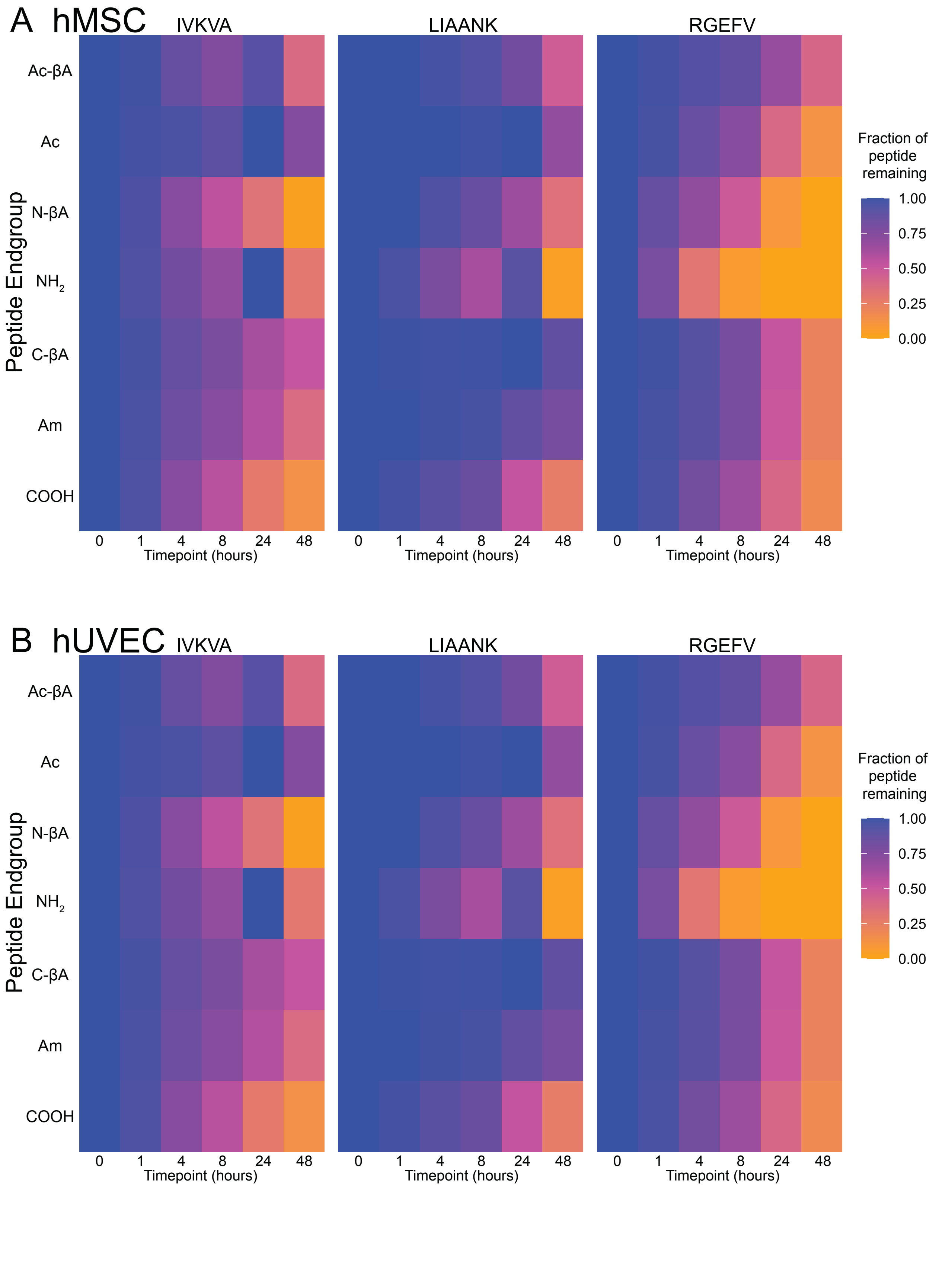

A hMSC
IVKVA
LIAANK
RGEFV
Peptide Endgroup
Ac-βA
Ac
N-βA
NH2
C-βA
Am
COOH
Fraction of peptide remaining
1.00
0.75
0.50
0.25
0.00
0
1
4
8
24
48
Timepoint (hours)
B hUVEC
IVKVA
LIAANK
RGEFV

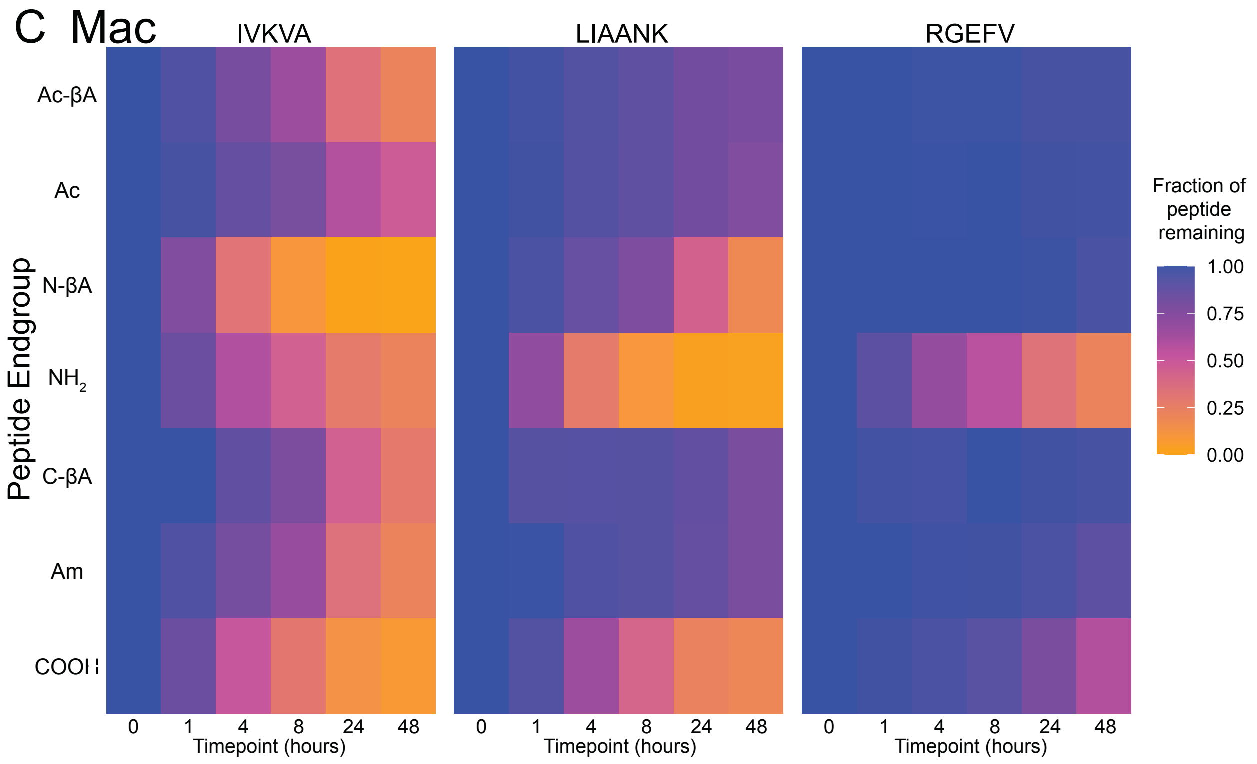
C Mac
IVKVA
LIAANK
RGEFV
Peptide Endgroup
Ac-βA
Ac
N-βA
NH2
C-βA
Am
COOI¦
0 1 4 8 24 48
Timepoint (hours)
Fraction of peptide remaining
1.00
0.75
0.50
0.25
0.00

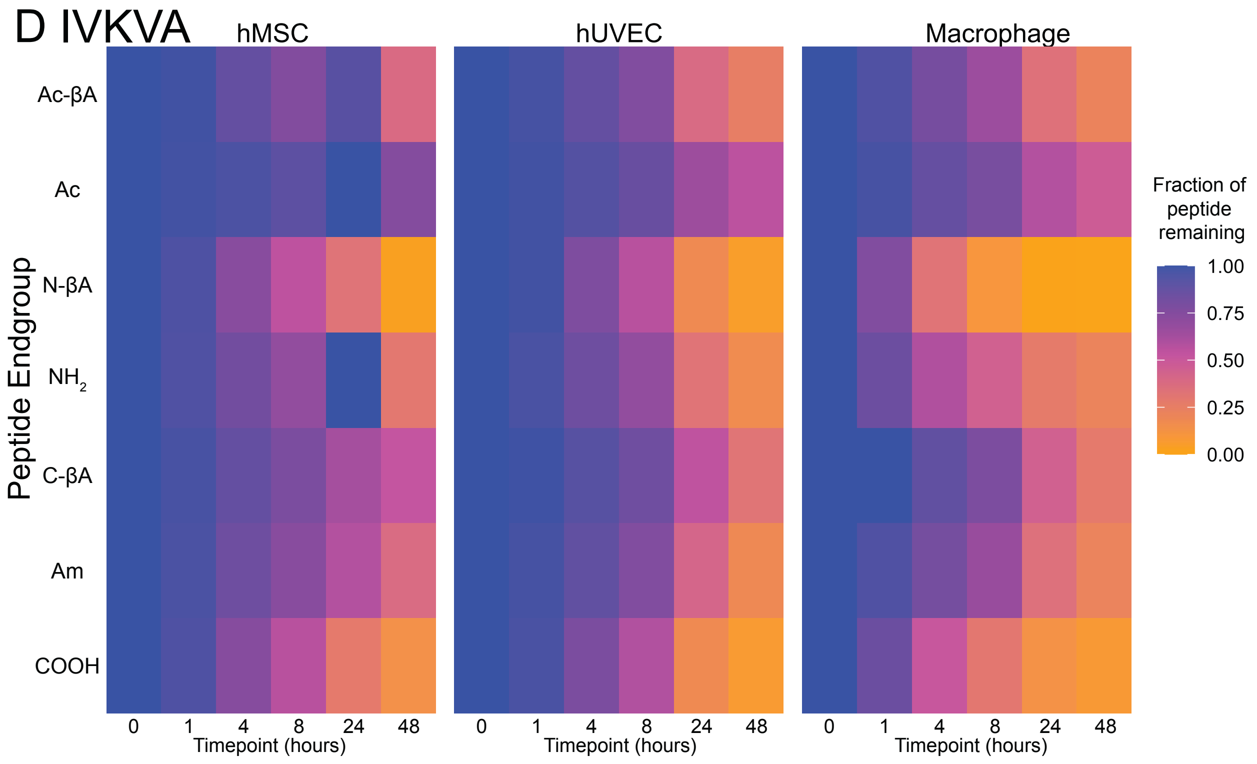
D IVKVA
hMSC
hUVEC
Macrophage
Peptide Endgroup
Ac-βA
Ac
N-βA
NH2
C-βA
Am
COOH
0 1 4 8 24 48
Timepoint (hours)
Fraction of peptide remaining
1.00
0.75
0.50
0.25
0.00

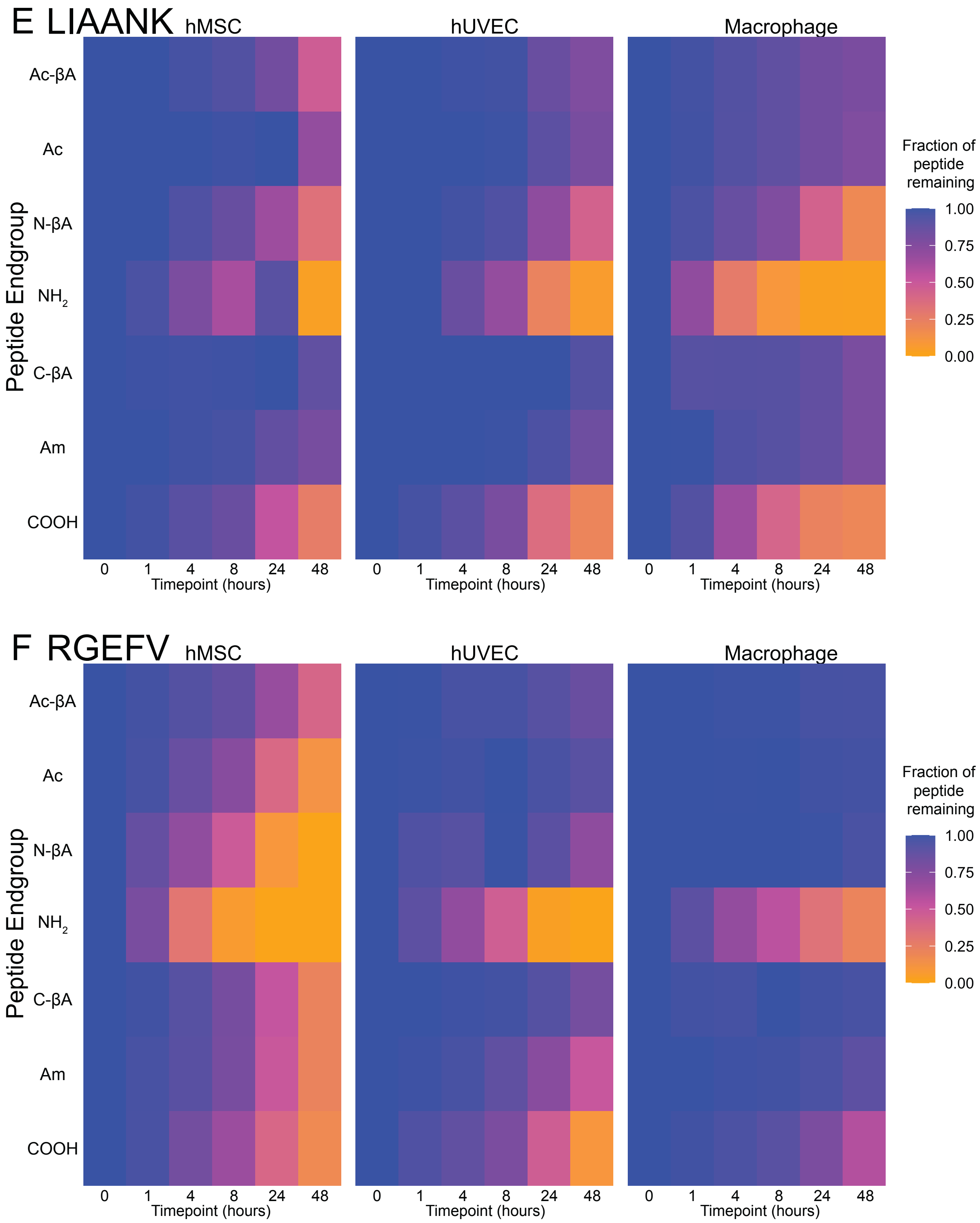

Figure S6. Effect of different peptide sequences on non-specific degradation. Degradation was quantified for the cell types (**A**) hMSCs, (**B**) hUVECs, (**C**), macrophages, and peptides (**D**) IVKVA, (**E**) LIAANK, and (**F**) RGEFV.

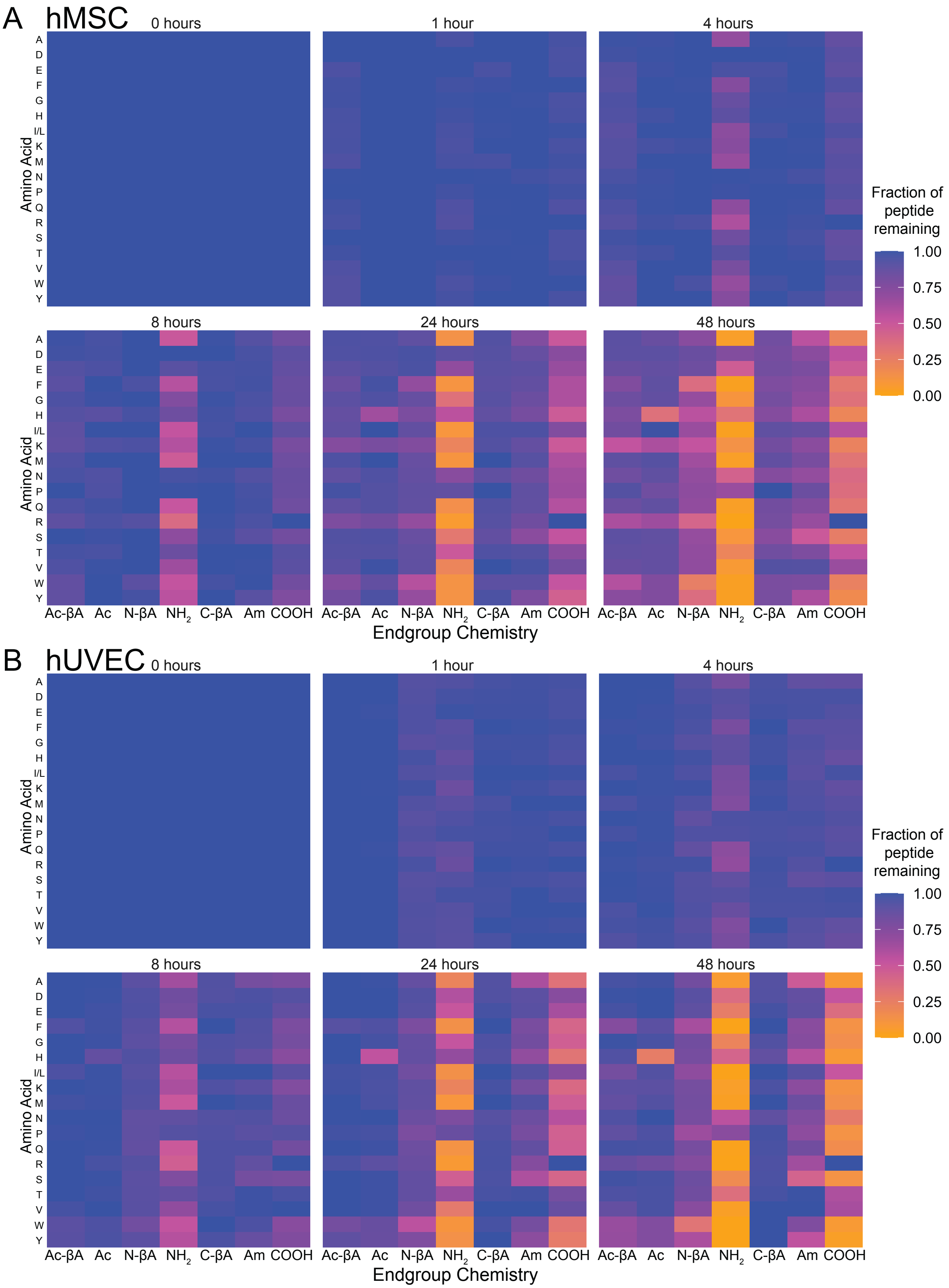
A hMSC
0 hours
1 hour
4 hours
8 hours
24 hours
48 hours
Amino Acid
A D E F G H I/L K M N P Q R S T V W Y
Ac-βA Ac N-βA NH2 C-βA Am COOH
Endgroup Chemistry
Fraction of peptide remaining
1.00
0.75
0.50
0.25
0.00
B hUVEC
0 hours
1 hour
4 hours
8 hours
24 hours
48 hours
Amino Acid
A D E F G H I/L K M N P Q R S T V W Y
Ac-βA Ac N-βA NH2 C-βA Am COOH
Endgroup Chemistry
Fraction of peptide remaining
1.00
0.75
0.50
0.25
0.00

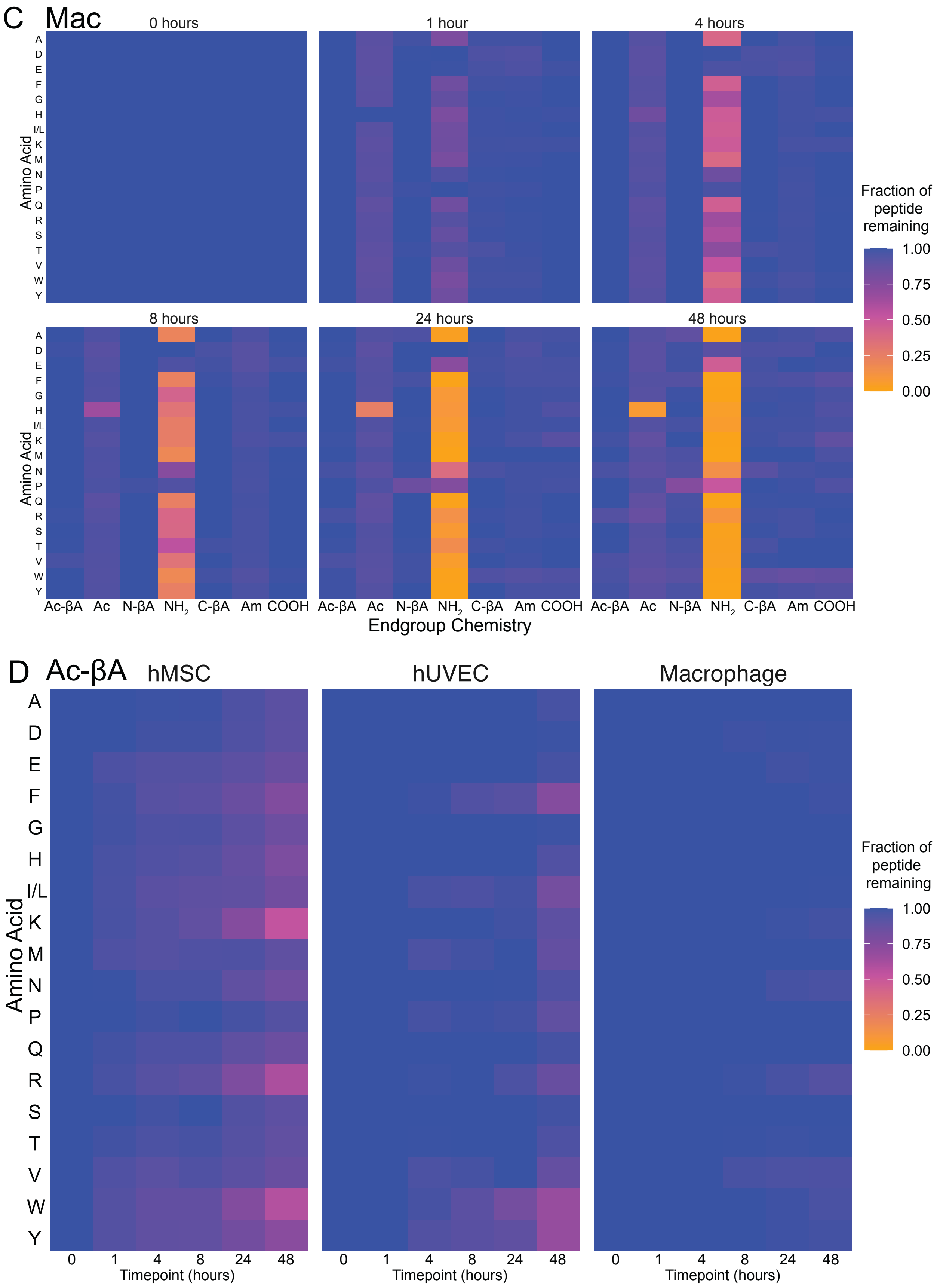
C
Mac
0 hours
1 hour
4 hours
8 hours
24 hours
48 hours
Amino Acid
A
D
E
F
G
H
I/L
K
M
N
P
Q
R
S
T
V
W
Y
Ac-βA
Ac
N-βA
NH_2
C-βA
Am
COOH
Endgroup Chemistry
Fraction of peptide remaining
1.00
0.75
0.50
0.25
0.00
D
Ac-βA
hMSC
hUVEC
Macrophage
0
1
4
8
24
48
Timepoint (hours)

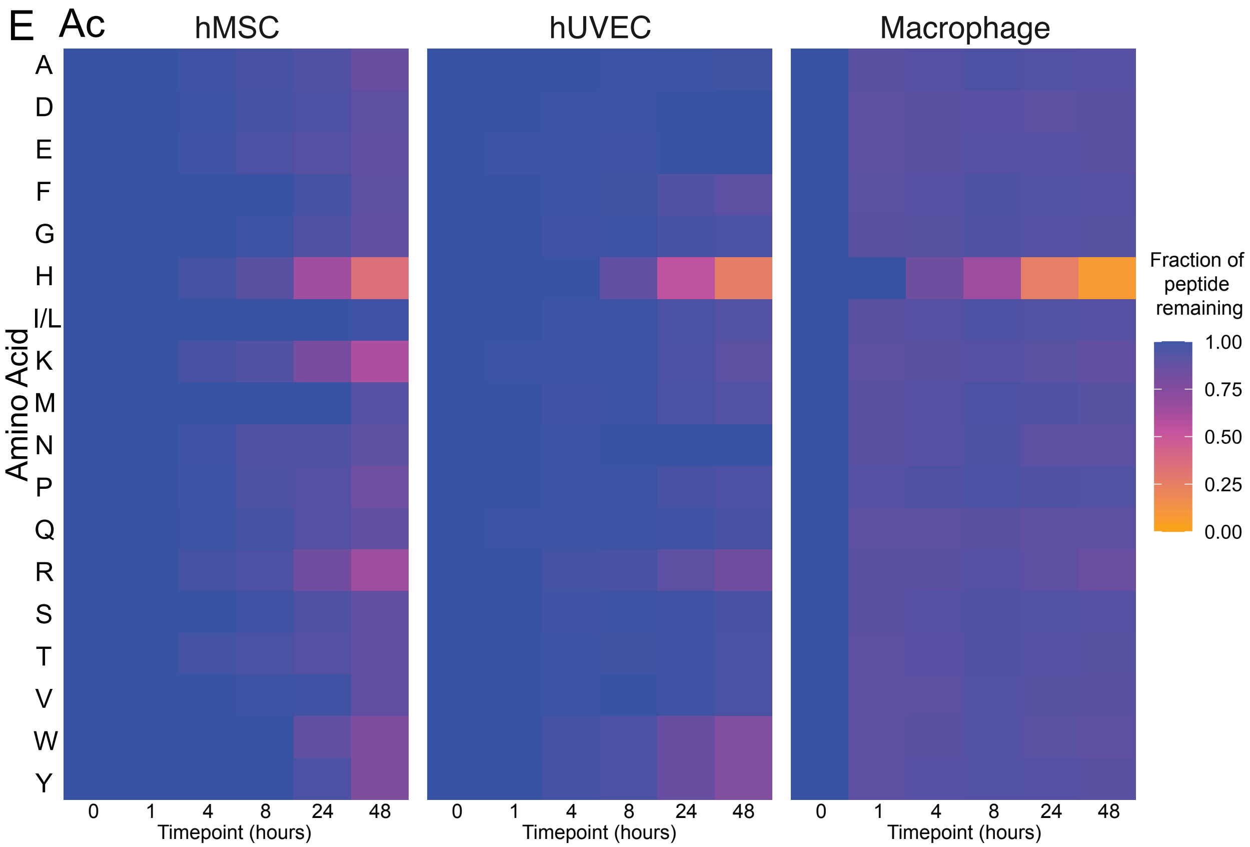
E Ac
hMSC
hUVEC
Macrophage
Amino Acid
A
D
E
F
G
H
I/L
K
M
N
P
Q
R
S
T
V
W
Y
0 1 4 8 24 48
Timepoint (hours)
Fraction of peptide remaining
1.00
0.75
0.50
0.25
0.00

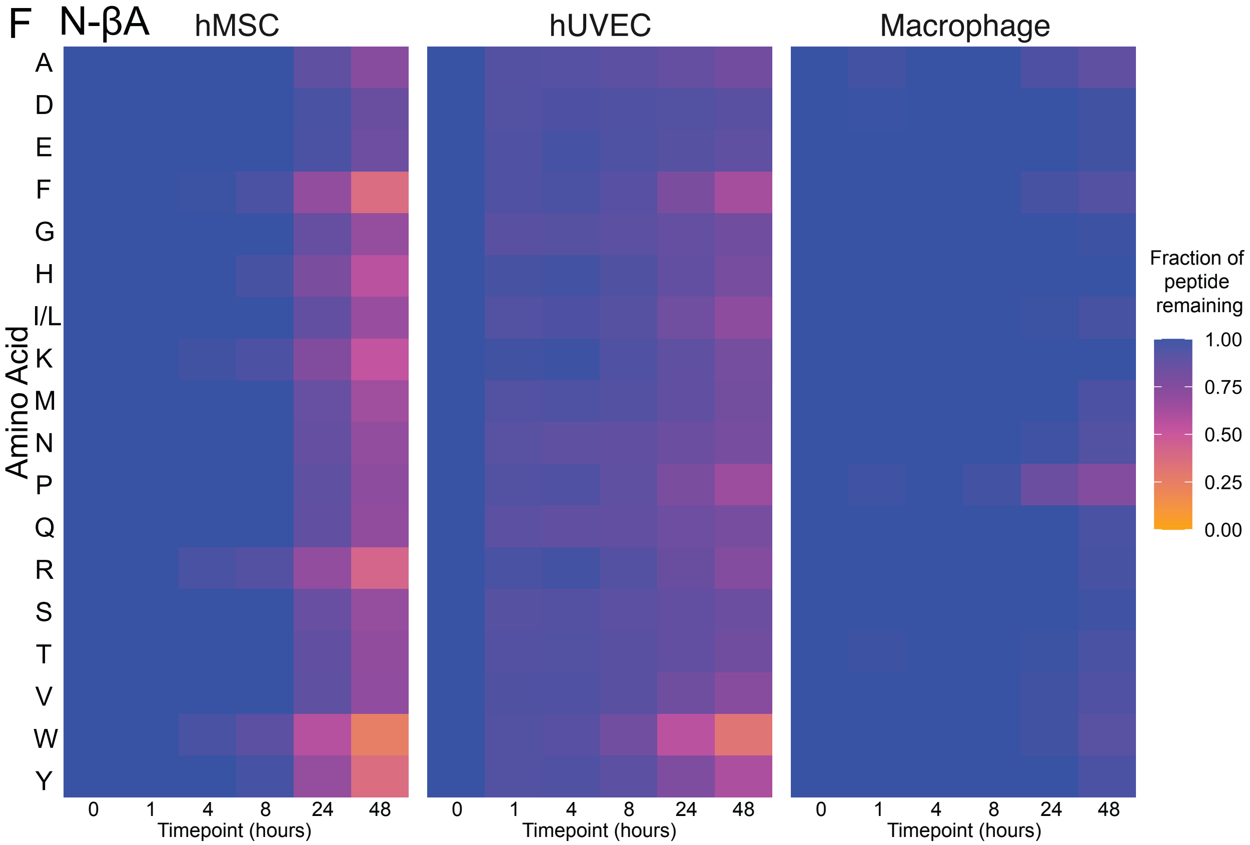
F N-βA
hMSC
hUVEC
Macrophage
Amino Acid
A
D
E
F
G
H
I/L
K
M
N
P
Q
R
S
T
V
W
Y
0 1 4 8 24 48
Timepoint (hours)
Fraction of peptide remaining
1.00
0.75
0.50
0.25
0.00

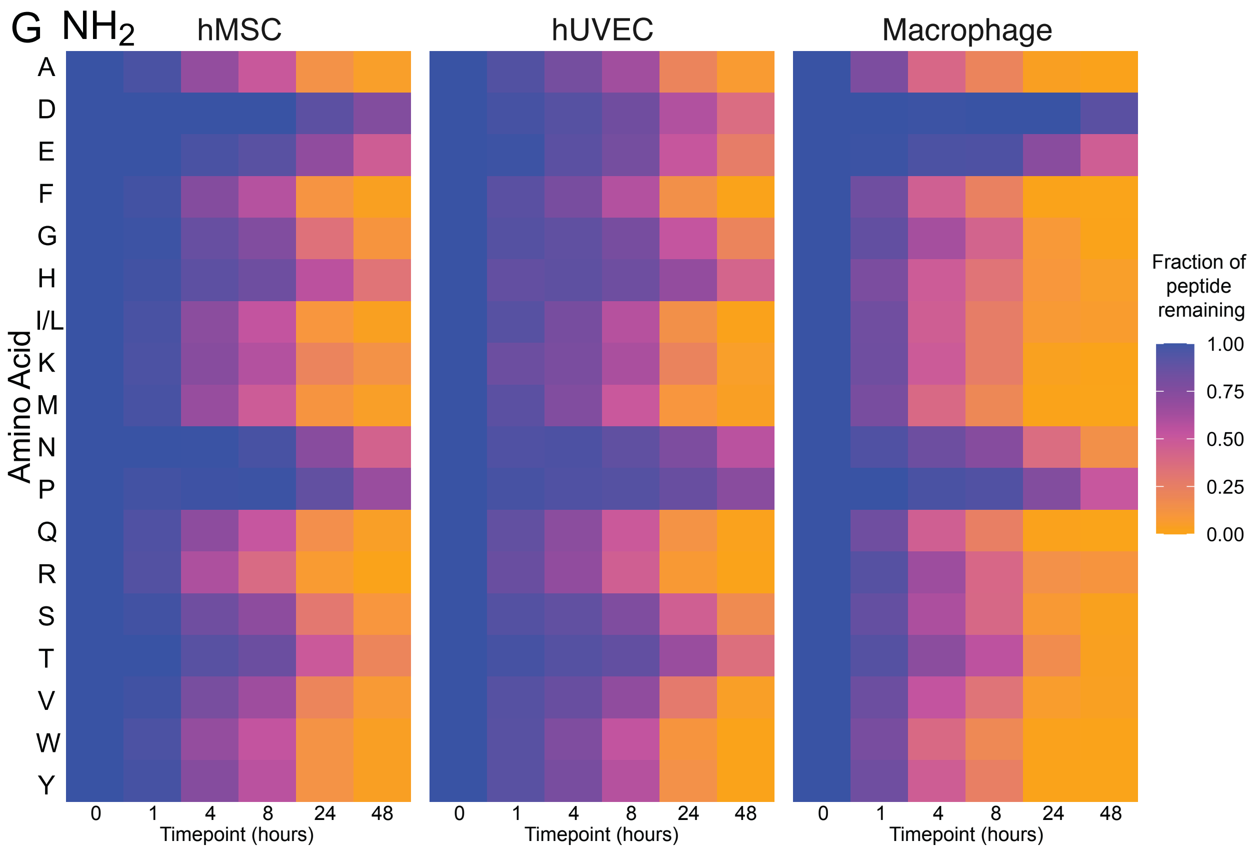
G NH2
hMSC
hUVEC
Macrophage
Amino Acid
A
D
E
F
G
H
I/L
K
M
N
P
Q
R
S
T
V
W
Y
0 1 4 8 24 48
Timepoint (hours)
Fraction of peptide remaining
1.00
0.75
0.50
0.25
0.00

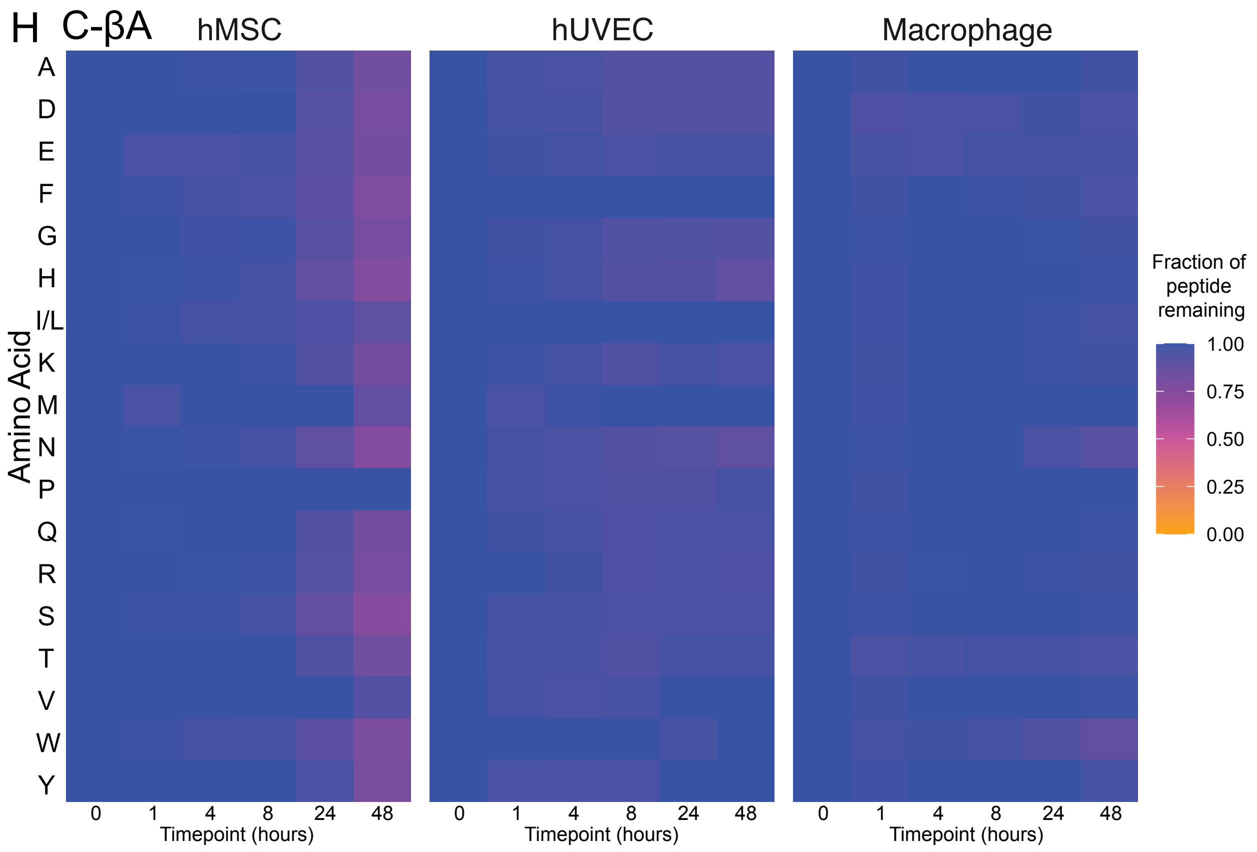
H C-βA
hMSC
hUVEC
Macrophage
Amino Acid
A
D
E
F
G
H
I/L
K
M
N
P
Q
R
S
T
V
W
Y
0 1 4 8 24 48
Timepoint (hours)
Fraction of peptide remaining
1.00
0.75
0.50
0.25
0.00

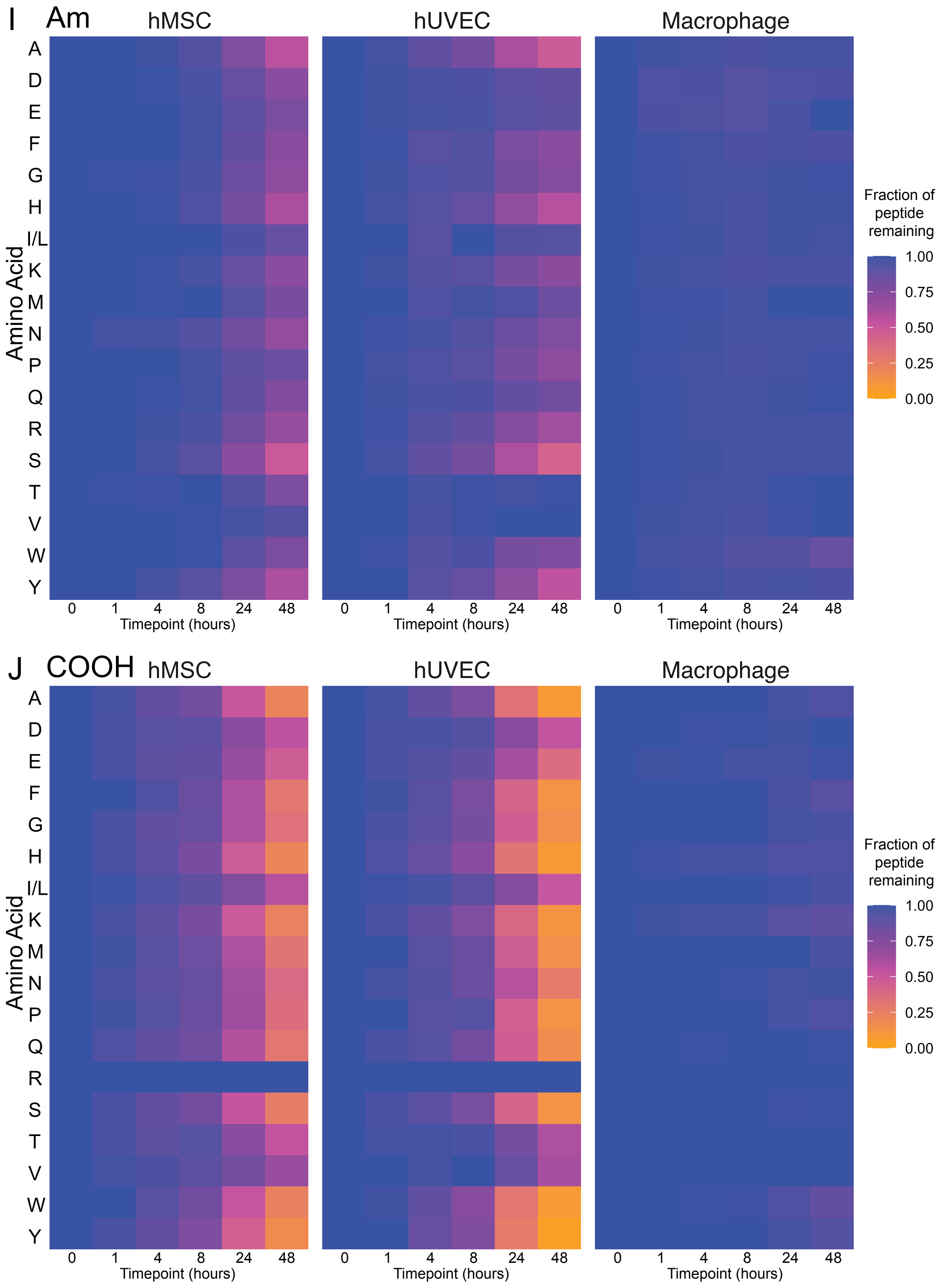
I
Am
hMSC
hUVEC
Macrophage
Amino Acid
A
D
E
F
G
H
I/L
K
M
N
P
Q
R
S
T
V
W
Y
0
1
4
8
24
48
Timepoint (hours)
Fraction of peptide remaining
1.00
0.75
0.50
0.25
0.00
J
COOH
hMSC
hUVEC
Macrophage

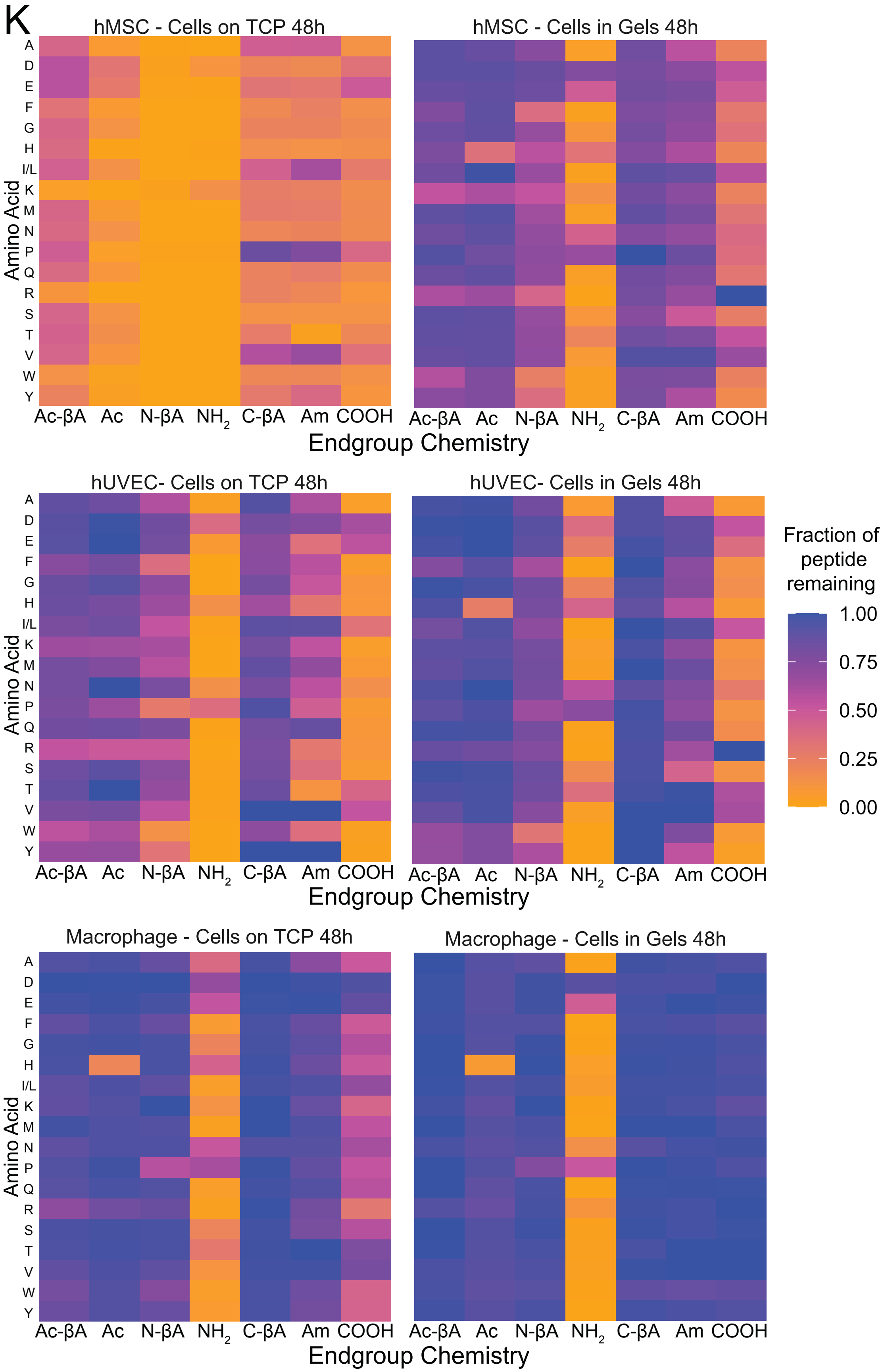
K
hMSC - Cells on TCP 48h
hMSC - Cells in Gels 48h
hUVEC- Cells on TCP 48h
hUVEC- Cells in Gels 48h
Macrophage - Cells on TCP 48h
Macrophage - Cells in Gels 48h
Amino Acid
A D E F G H I/L K M N P Q R S T V W Y
Ac-βA Ac N-βA NH2 C-βA Am COOH
Endgroup Chemistry
Fraction of peptide remaining
1.00
0.75
0.50
0.25
0.00

Figure S7. Degradation of soluble peptide libraries by cells in PEG hydrogels. (**A**) hMSCs, (**B**) hUVECs, (**C**), macrophages, and by peptide endgroup (**D**) Ac-βA, (**E**) Ac, (**F**) N-βA, (**G**) NH_2, (**H**) C-βA, I) Am, (**J**) COOH. (**K**) Direct comparison of peptide degradation by cells on tissue culture plastic (TCP) and cells in gels.

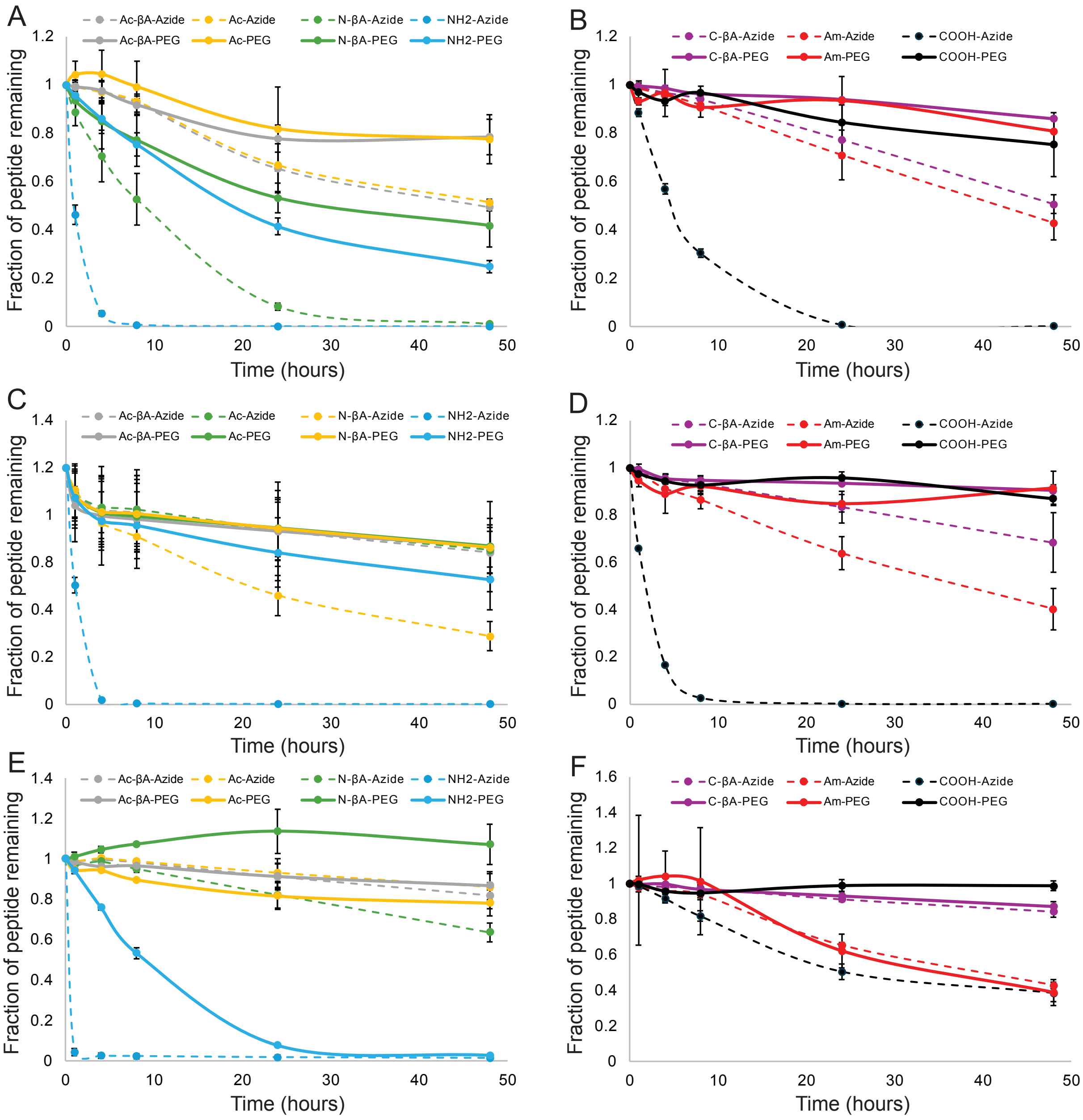

Figure S8. PEG conjugation to peptides slows degradation across endgroups and cell types. (**A**) hMSC N-terminal chemistries, (**B**) hMSC C-terminal chemistries, (**C**) hUVEC N-terminal chemistries, (**D**) hUVEC C-terminal chemistries, (**E**) macrophage N-terminal chemistries, (**F**) macrophage C-terminal chemistries.

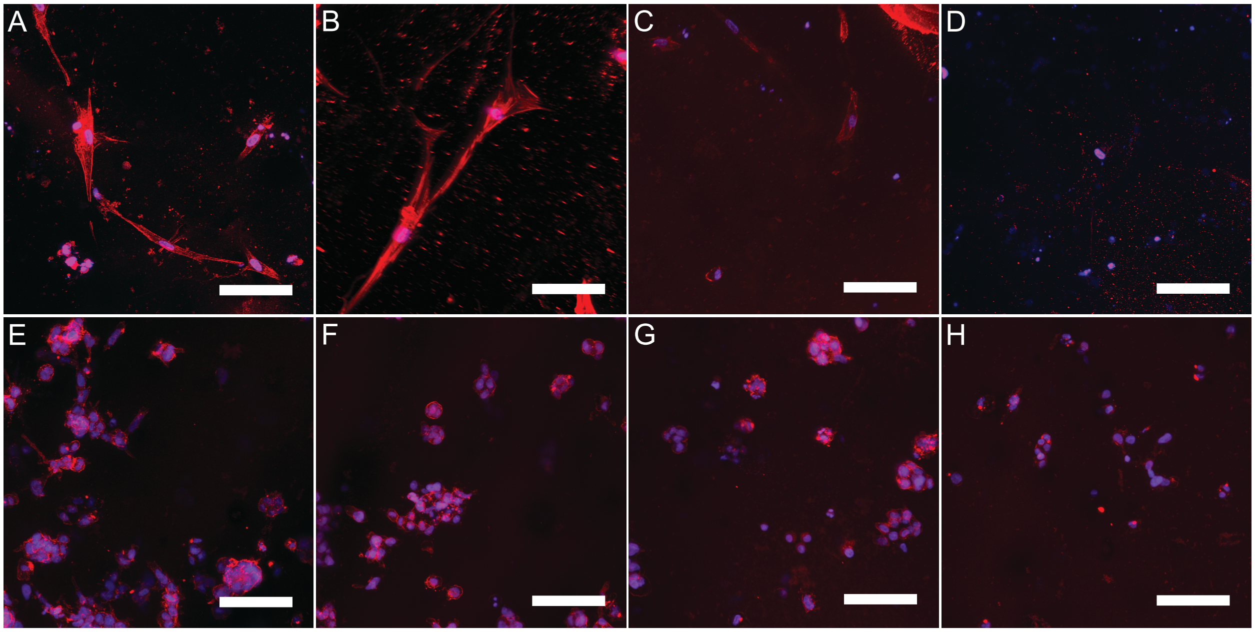

Figure S9. Cells growing in gels with different RGD presentations. hUVECs growing in gels with (**A**) cyclic RGDS, (**B**) Ac-βA-GRGDS, (**C**) NH_2-GRGDS, and (**D**) no added RGDS. Macrophages growing in gels with (**E**) cyclic RGDS, (**F**) Ac-βA-GRGDS, (**G**) NH_2-GRGDS, and (**H**) no added RGDS. It should be noted that the viability assay (Fig. S9) indicates negligible of metabolic activity within hUVEC gels lacking RGD sequences. Scale bar is 100 μm, and red is actin and blue is the cell nuclei.

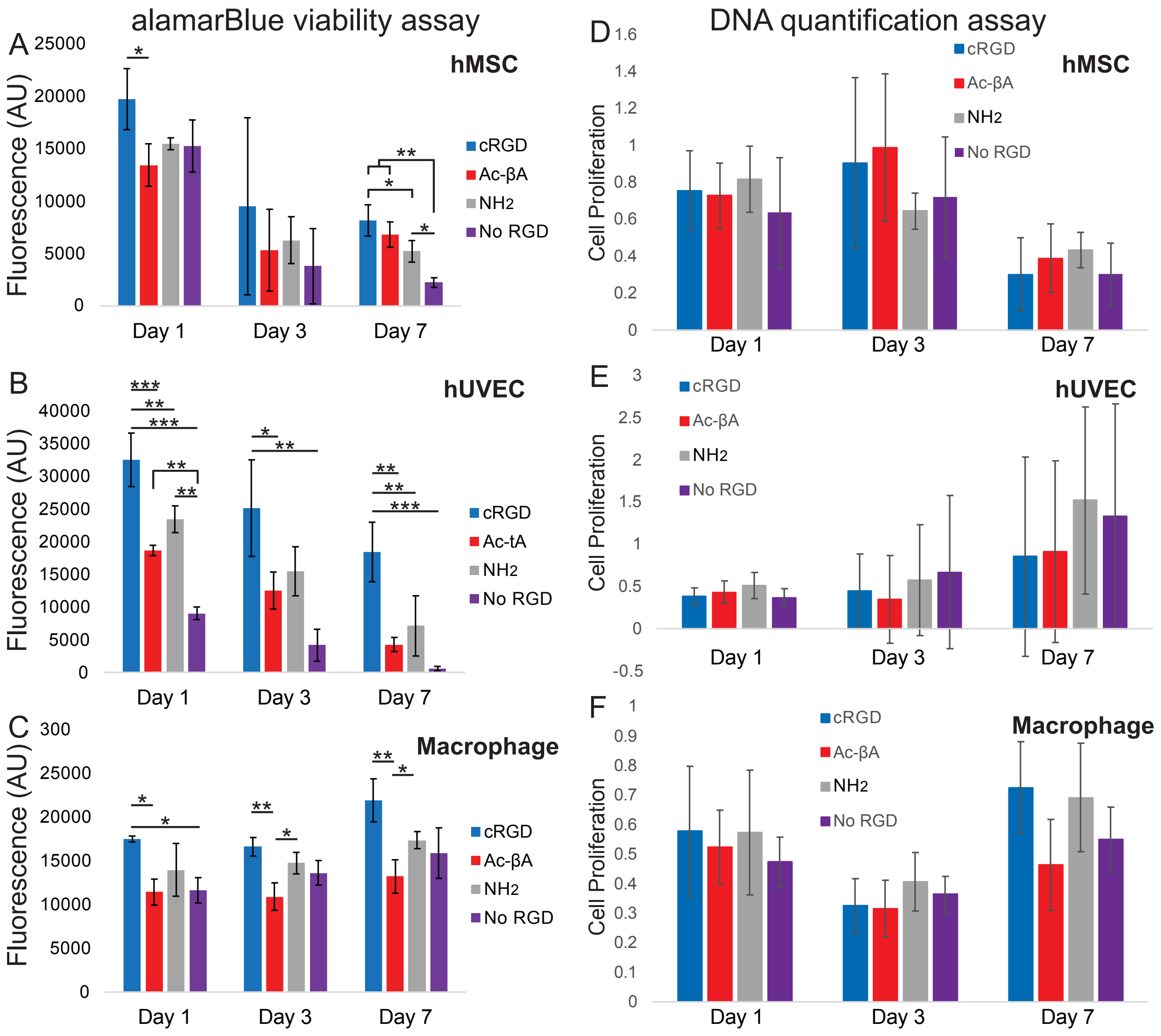

Figure S10. Quantification of viability and proliferation in hydrogels containing different RGD sequences. An alamarBlue metabolic activity assays was performed on (**A**) hMSCs, (**B**) hUVECs, and (**C**) macrophages and a DNA quantification assay was performed on (**D**) hMSCs, (**E**) hUVECs, and (**F**) macrophages. * indicates $p < 0.05$, ** indicates $p < 0.01$, *** indicates $p < 0.0001$ by Tukey's post hoc test.

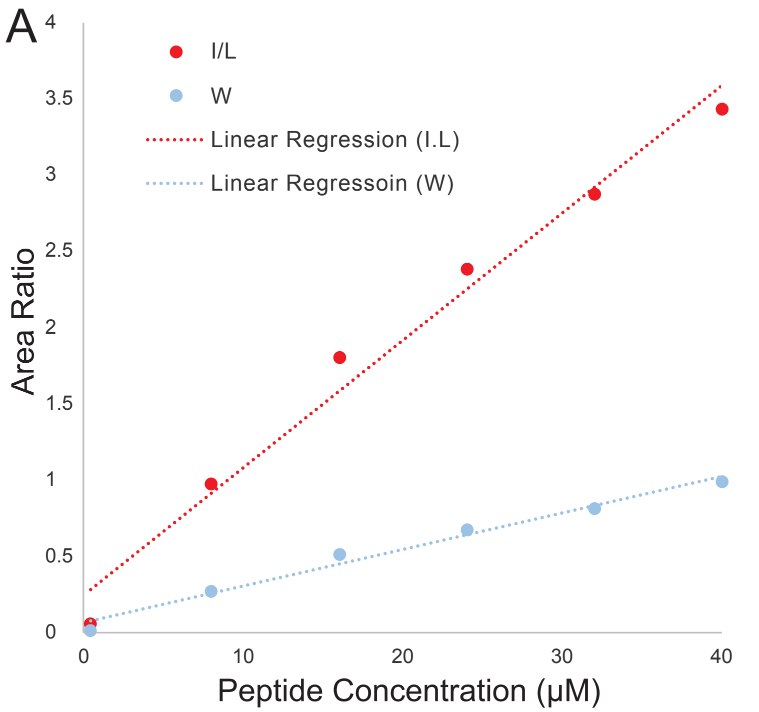

B

	K	H	R	N	S	Q	G	A	T	E
Ac-βA	0.977	0.965	0.978	0.979	0.978	0.986	0.978	0.977	0.977	0.983
Ac	0.936	0.931	0.938	0.791	0.977	0.936	0.986	0.976	0.979	0.979
N-βA	0.962	0.943	0.976	0.995	0.998	0.992	0.997	0.998	0.997	0.996
NH2	0.984	0.983	0.969	0.981	0.981	0.980	0.984	0.981	0.982	0.993
C-βA	0.994	0.989	0.982	0.992	0.992	0.991	0.989	0.994	0.993	0.995
Am	0.998	0.995	0.996	0.999	0.994	0.996	0.999	0.990	0.998	0.997
COOH	0.871	0.903		0.934	0.900	0.914	0.939	0.896	0.974	0.979

	D	P	V	Y	M	I/L	F	W	Average	St Dev
Ac-βA	0.983	0.979	0.977	0.973	0.979	0.981	0.979	0.984	0.978	0.004
Ac	0.738	0.898	0.870	0.808	0.858	0.891	0.841	0.855	0.899	0.073
N-βA	0.998	0.997	0.996	0.989	0.992	0.995	0.992	0.982	0.989	0.015
NH2	0.983	0.993	0.985	0.978	0.979	0.985	0.984	0.983	0.983	0.005
C-βA	0.992	0.992	0.983	0.972	0.991	0.987	0.991	0.988	0.989	0.006
Am	0.999	0.999	0.999	0.997	0.997	0.998	0.998	0.998	0.997	0.002
COOH	0.988	0.928	0.990	0.918	0.935	0.982	0.944	0.953	0.938	0.036

Figure S11. Standard curves validating the use of LCMS to measure the concentration of peptides. Peptide libraries were run on the LCMS at different concentrations ranging from 0.4-40 μM and the amount of peptide was quantified. (**A**) Two representative conditions, I/L and W peptides in Ac-βA and the linear regression line fitting the data points. (**B**) A table of the R-squared values for every endgroup/amino acid combinations.

A

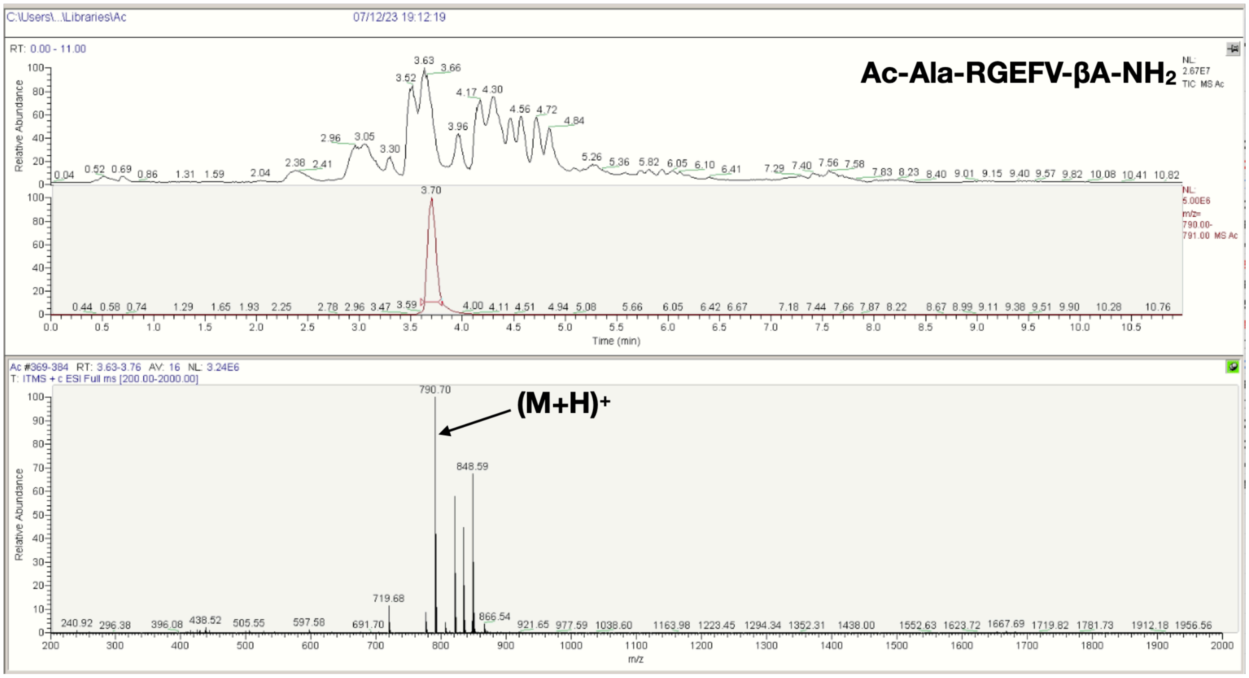
C:\Users\...\Libraries\Ac
07/12/23 19:12:19
Ac-Ala-RGEFV-βA-NH2
RT: 0.00 - 11.00
Relative Abundance
Time (min)
Ac #369-384 RT: 3.63-3.76 AV: 16 NL: 3.24E6
T: ITMS + c ESI Full ms [200.00-2000.00]
790.70
(M+H)+
848.59
m/z

B

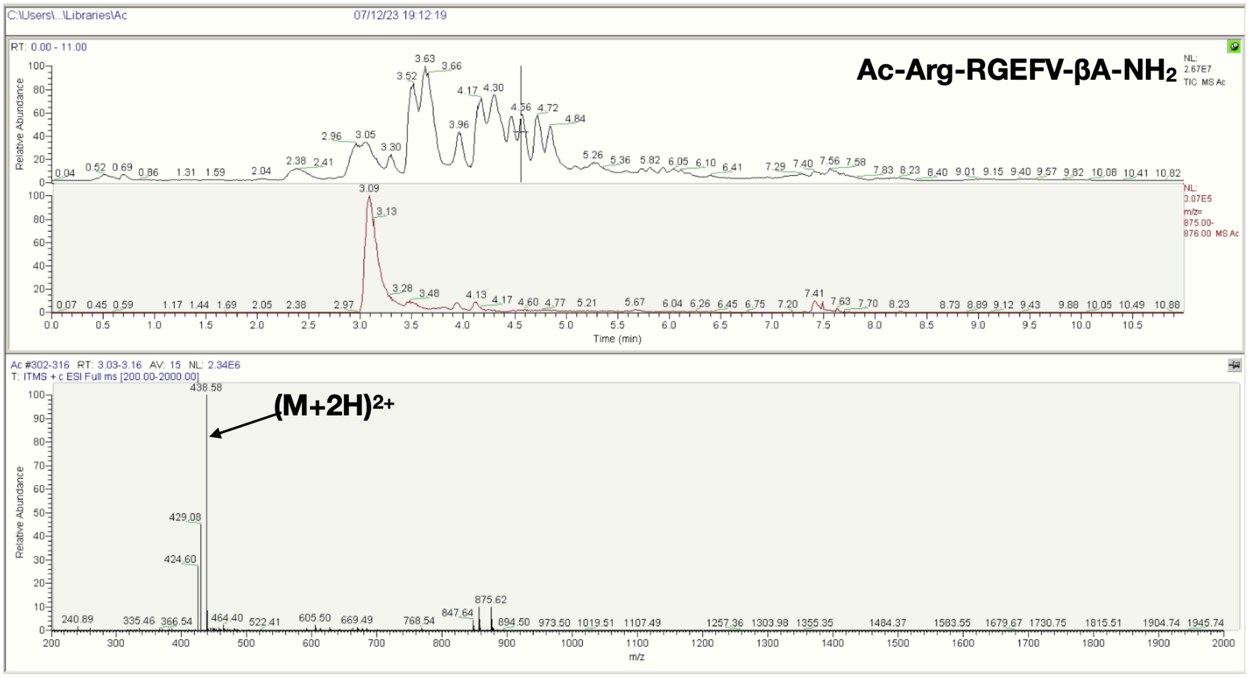
C:\Users\...\Libraries\Ac
07/12/23 19:12:19
Ac-Arg-RGEFV-βA-NH2
RT: 0.00 - 11.00
Relative Abundance
Time (min)
Ac #302-316 RT: 3.03-3.16 AV: 15 NL: 2.34E6
T: ITMS + c ESI Full ms [200.00-2000.00]
438.58
(M+2H)2+
429.08
424.60
875.62
m/z

C

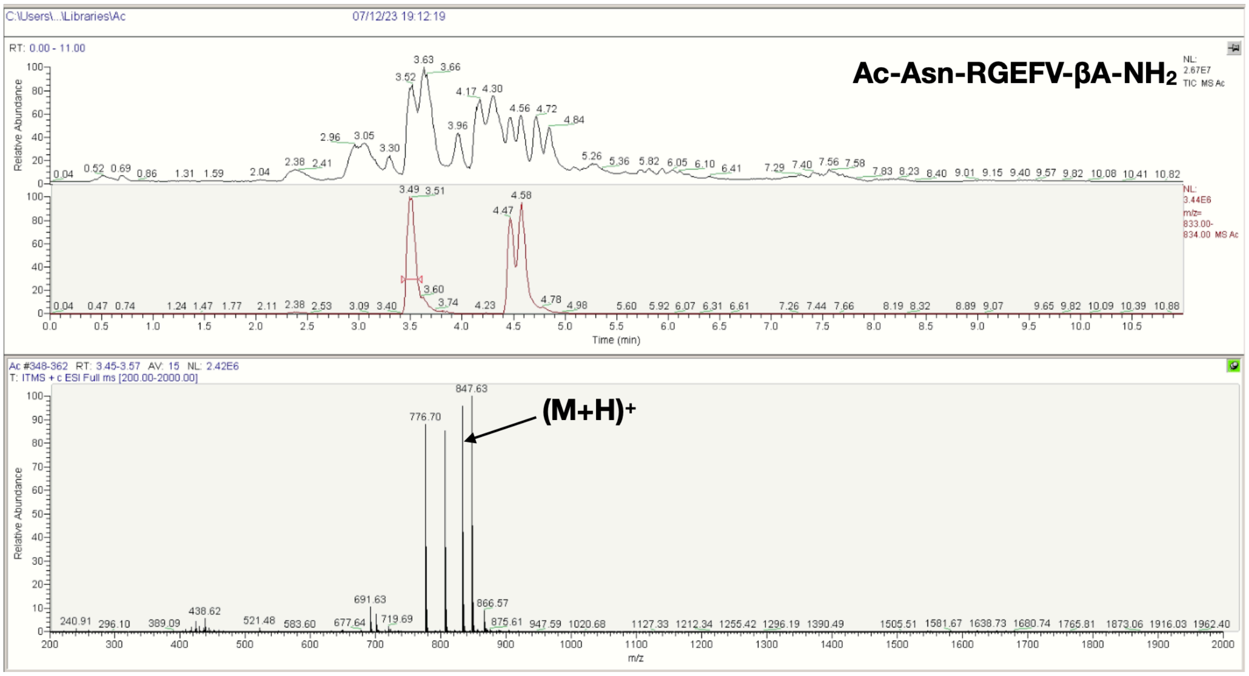
Ac-Asn-RGEFV-βA-NH2
(M+H)+
Relative Abundance
Time (min)
m/z
847.63
776.70

D

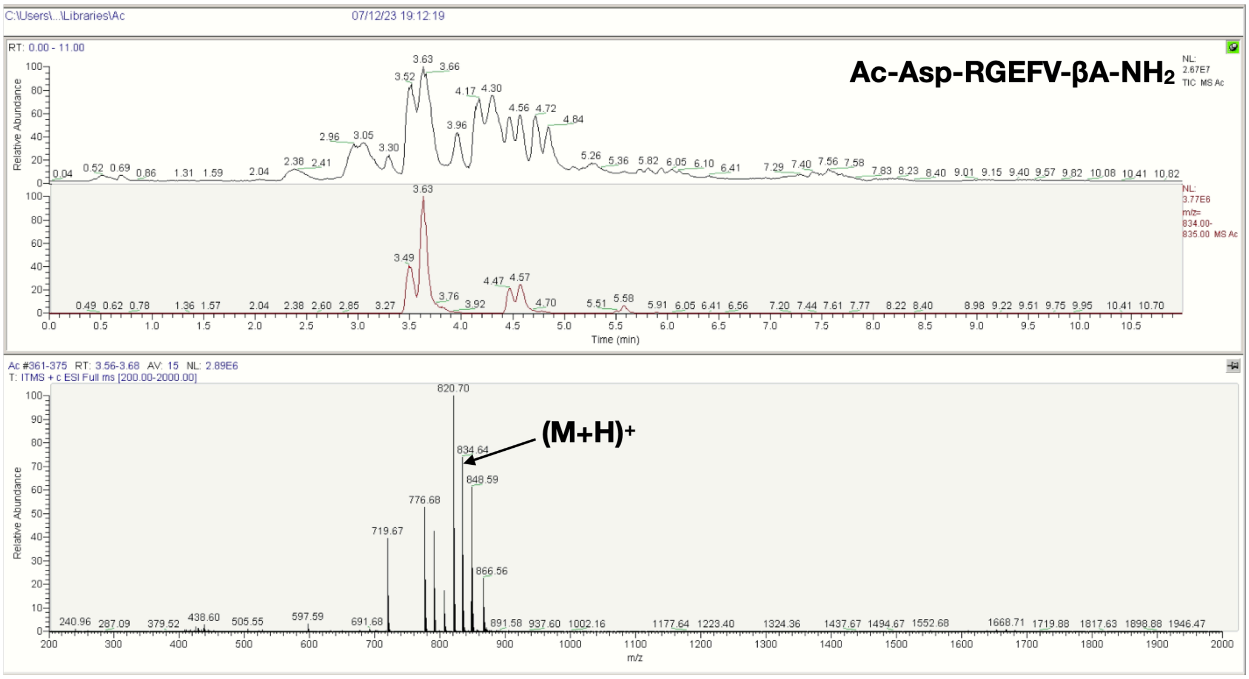
Ac-Asp-RGEFV-βA-NH2
(M+H)+
Relative Abundance
Time (min)
m/z
820.70
834.64
848.59
776.68
719.67

E

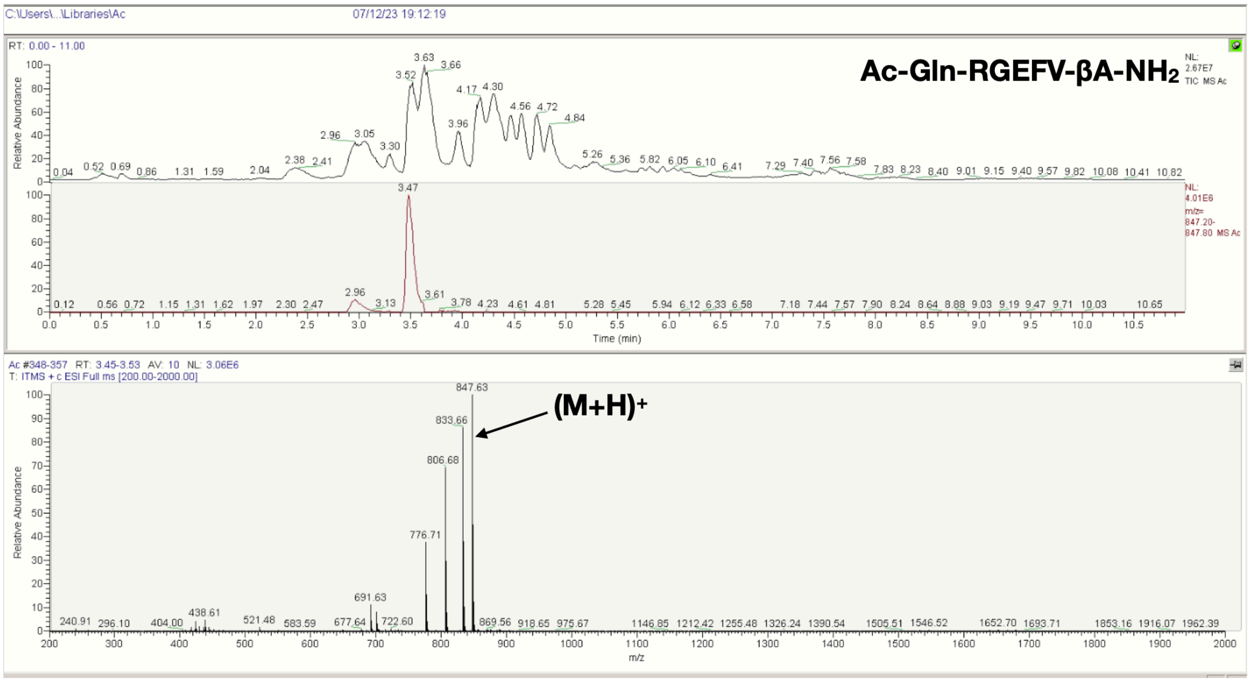
C:\Users\...\Libraries\Ac
07/12/23 19:12:19
Ac-Gln-RGEFV-βA-NH2
Relative Abundance
Time (min)
Ac #348-357 RT: 3.45-3.53 AV: 10 NL: 3.06E6
T: ITMS + c ESI Full ms [200.00-2000.00]
847.63
833.66
806.68
776.71
691.63
(M+H)+
m/z

F

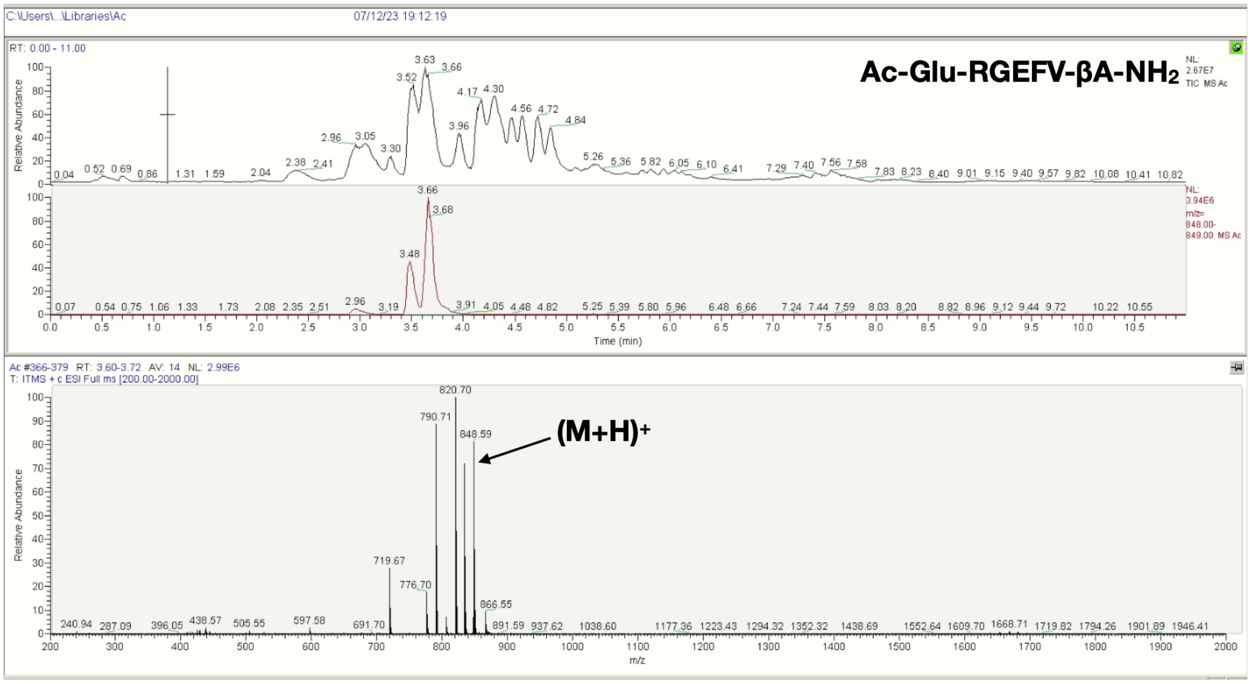
C:\Users\...\Libraries\Ac
07/12/23 19:12:19
Ac-Glu-RGEFV-βA-NH2
Relative Abundance
Time (min)
Ac #366-379 RT: 3.60-3.72 AV: 14 NL: 2.99E6
T: ITMS + c ESI Full ms [200.00-2000.00]
820.70
790.71
848.59
719.67
776.70
866.55
(M+H)+
m/z

G

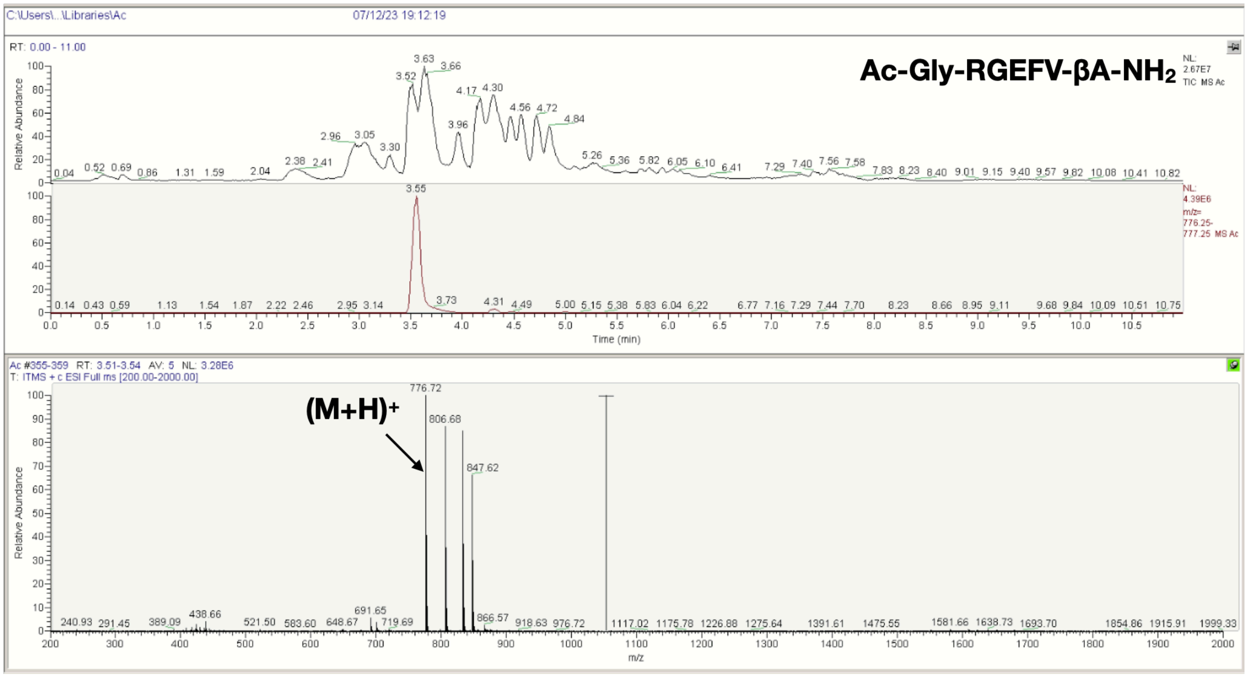

C:\Users\...\Libraries\Ac
07/12/23 19:12:19
RT: 0.00 - 11.00
Ac-Gly-RGEFV-βA-NH2
NL: 2.67E7 TIC MS Ac
NL: 4.39E6 m/z= 776.25-777.25 MS Ac
Relative Abundance
Time (min)
Ac #355-359 RT: 3.51-3.54 AV: 5 NL: 3.28E6
T: ITMS + c ESI Full ms [200.00-2000.00]
(M+H)+
776.72
806.68
847.62
m/z

H

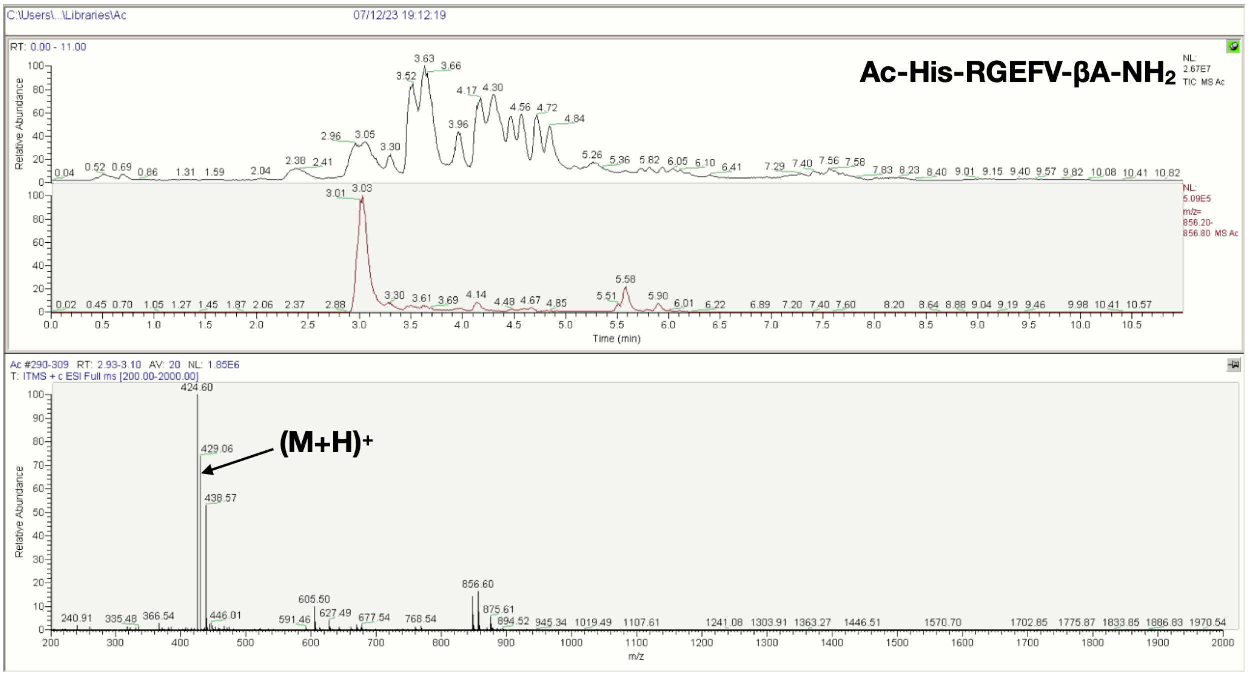

C:\Users\...\Libraries\Ac
07/12/23 19:12:19
RT: 0.00 - 11.00
Ac-His-RGEFV-βA-NH2
NL: 2.67E7 TIC MS Ac
NL: 5.09E5 m/z= 856.20-856.80 MS Ac
Relative Abundance
Time (min)
Ac #290-309 RT: 2.93-3.10 AV: 20 NL: 1.85E6
T: ITMS + c ESI Full ms [200.00-2000.00]
(M+H)+
424.60
429.06
438.57
856.60
m/z

I

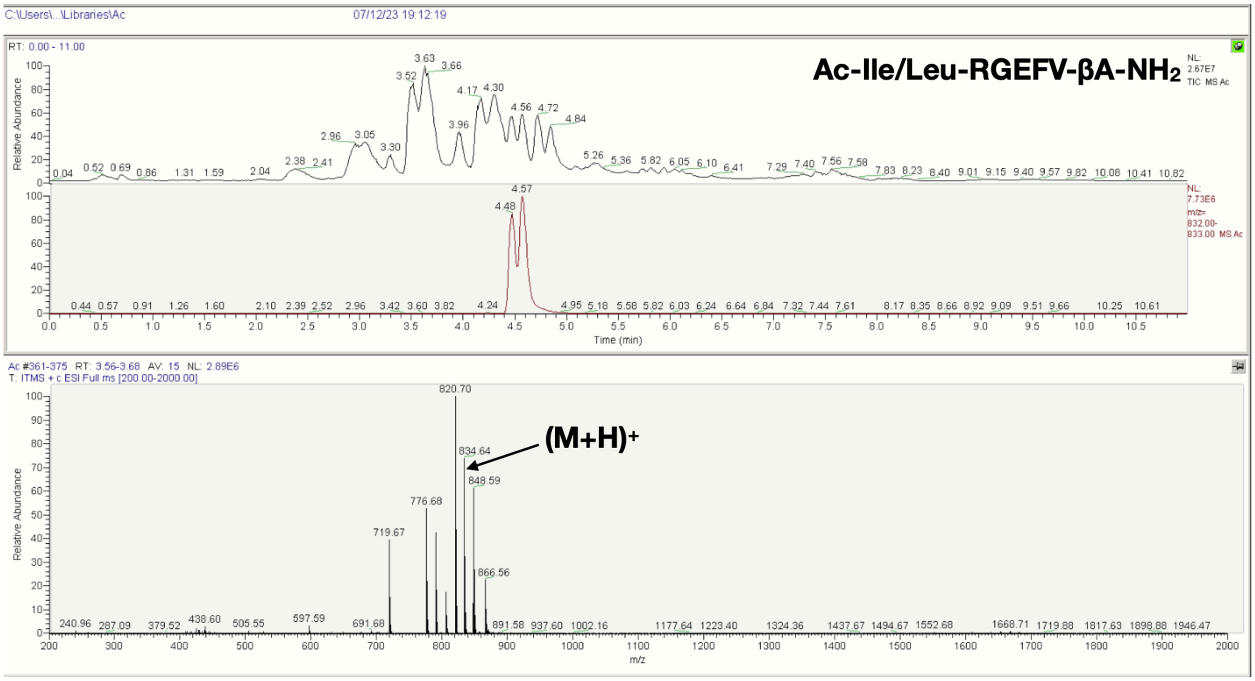

J

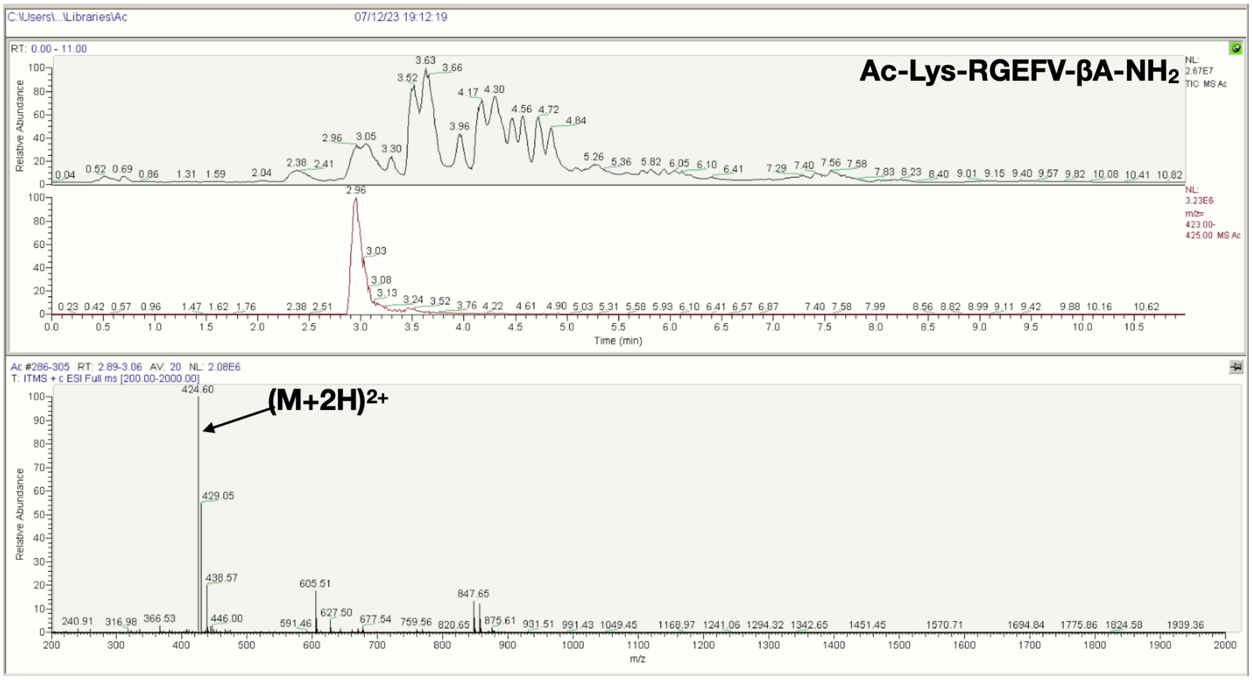

K

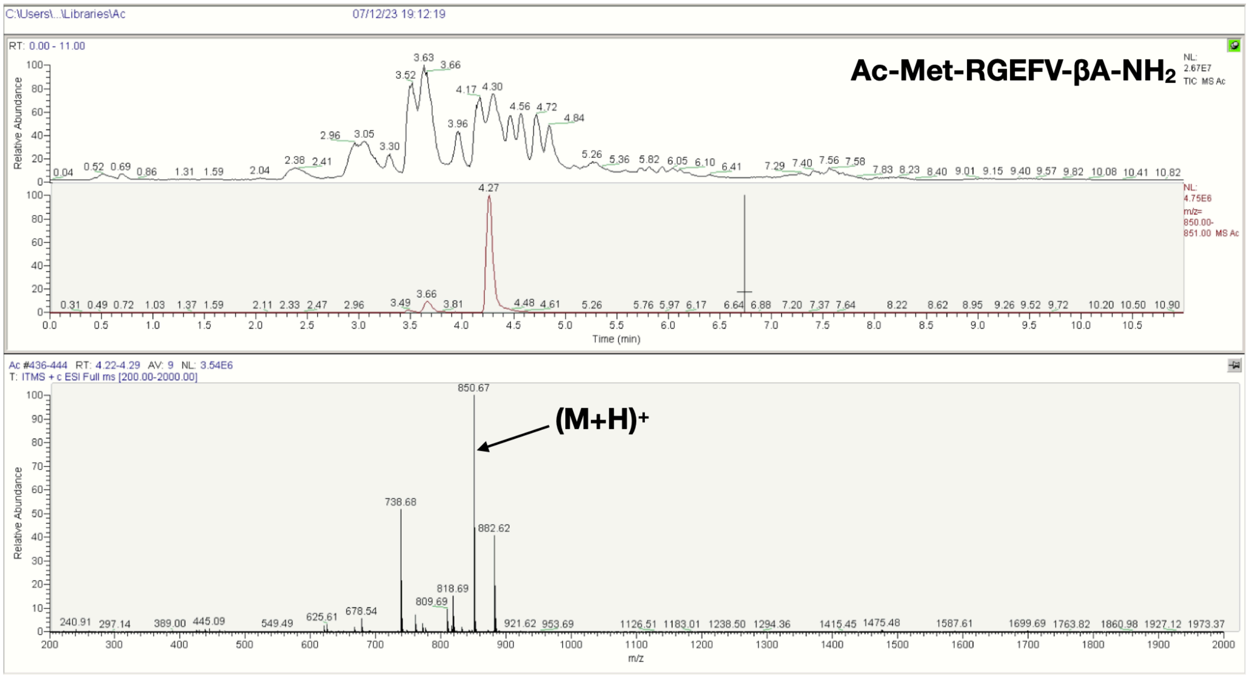
Ac-Met-RGEFV-βA-NH2
(M+H)+
850.67
738.68
882.62
818.69
809.69
Time (min)
m/z
Relative Abundance

L

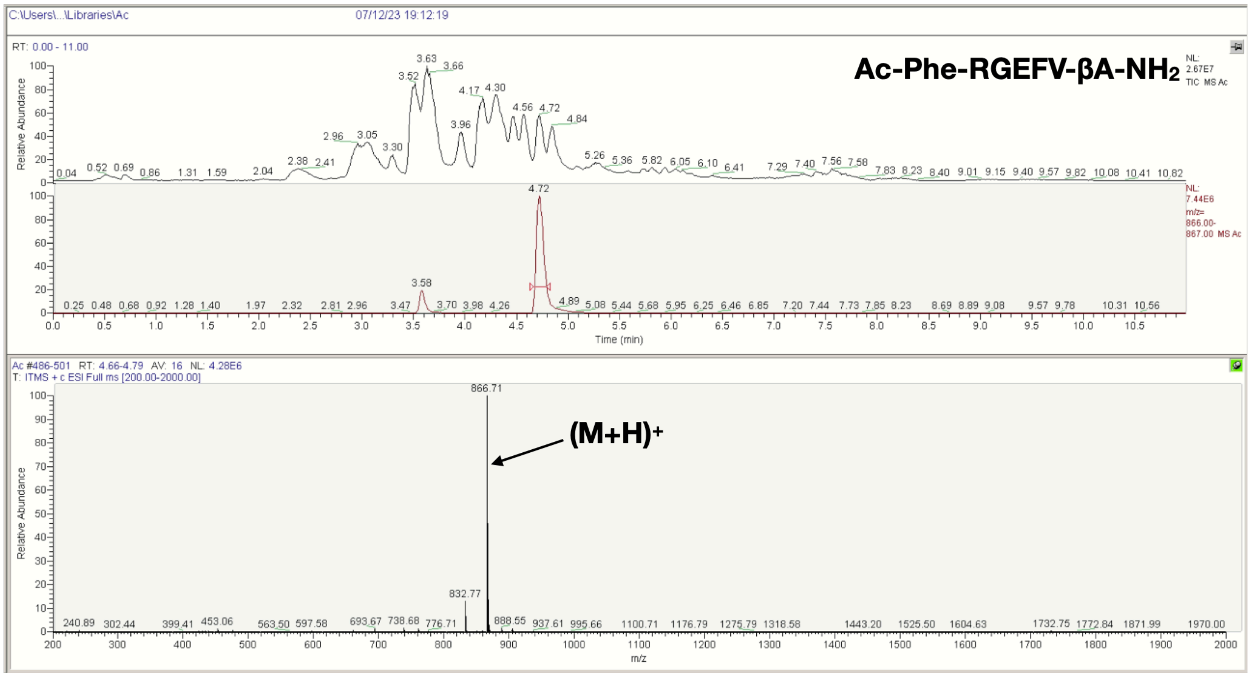
Ac-Phe-RGEFV-βA-NH2
(M+H)+
866.71
832.77
Time (min)
m/z
Relative Abundance

M

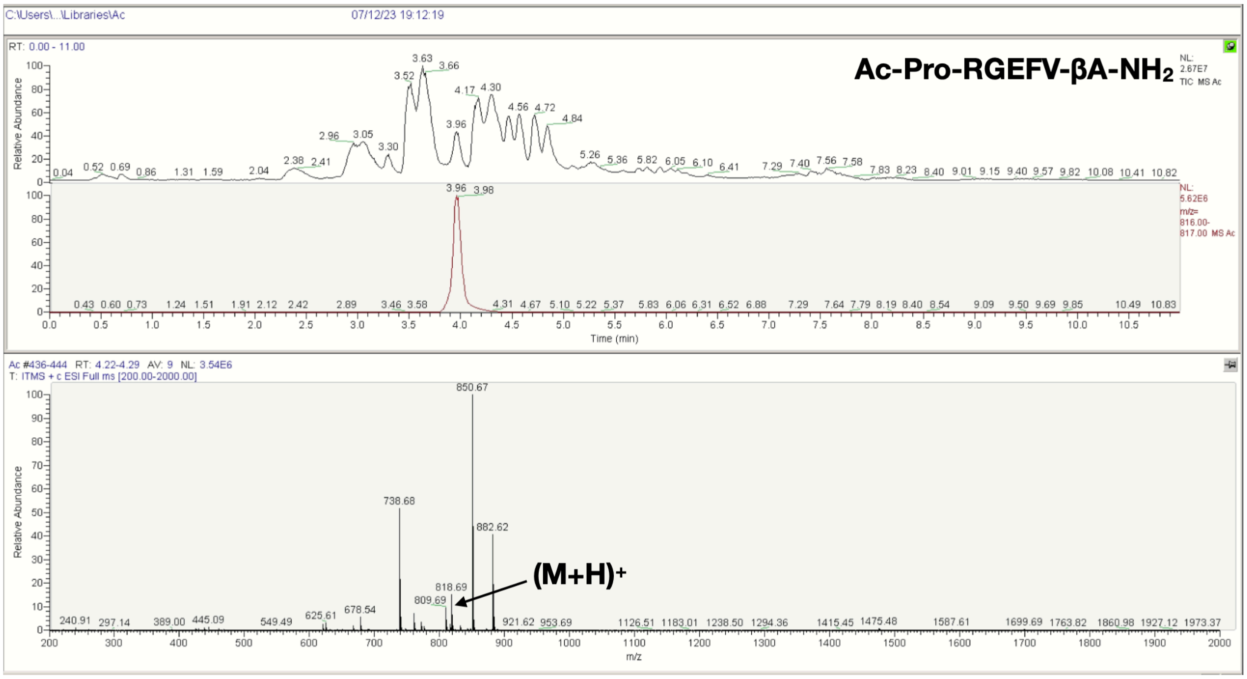

N

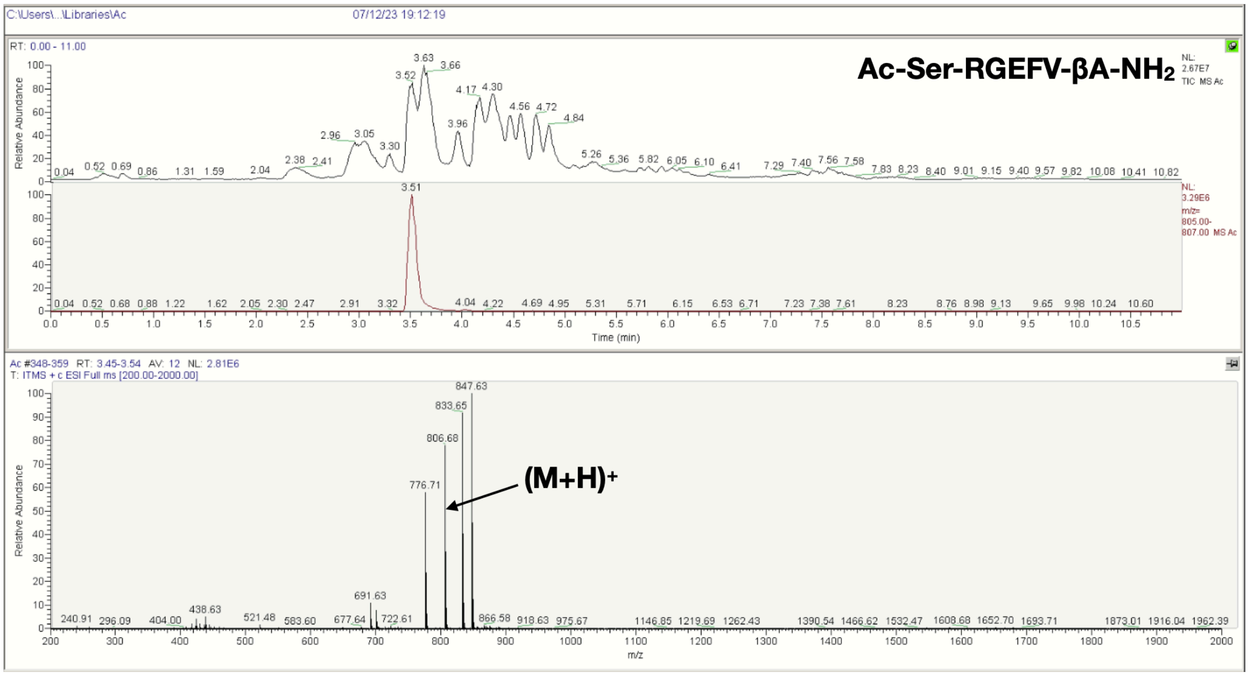

O

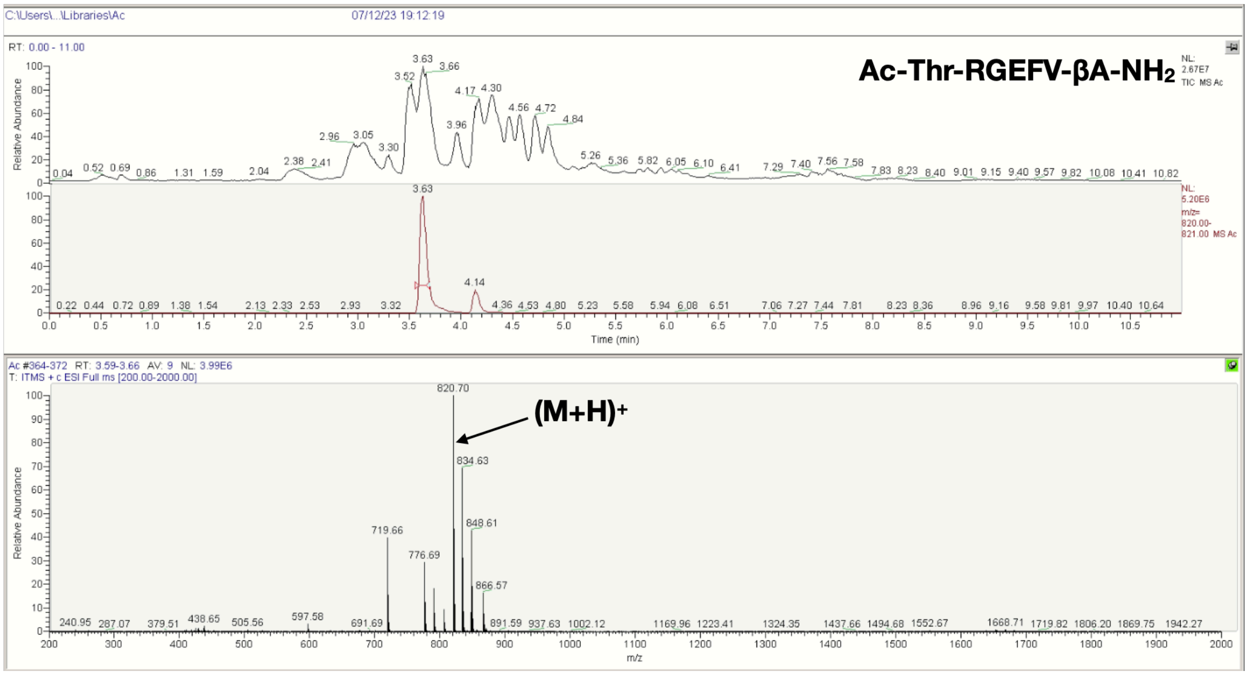
Ac-Thr-RGEFV-βA-NH2
(M+H)+
820.70
834.63
848.61
719.66
776.69
866.57
3.63
4.14
Time (min)
m/z
Relative Abundance

P

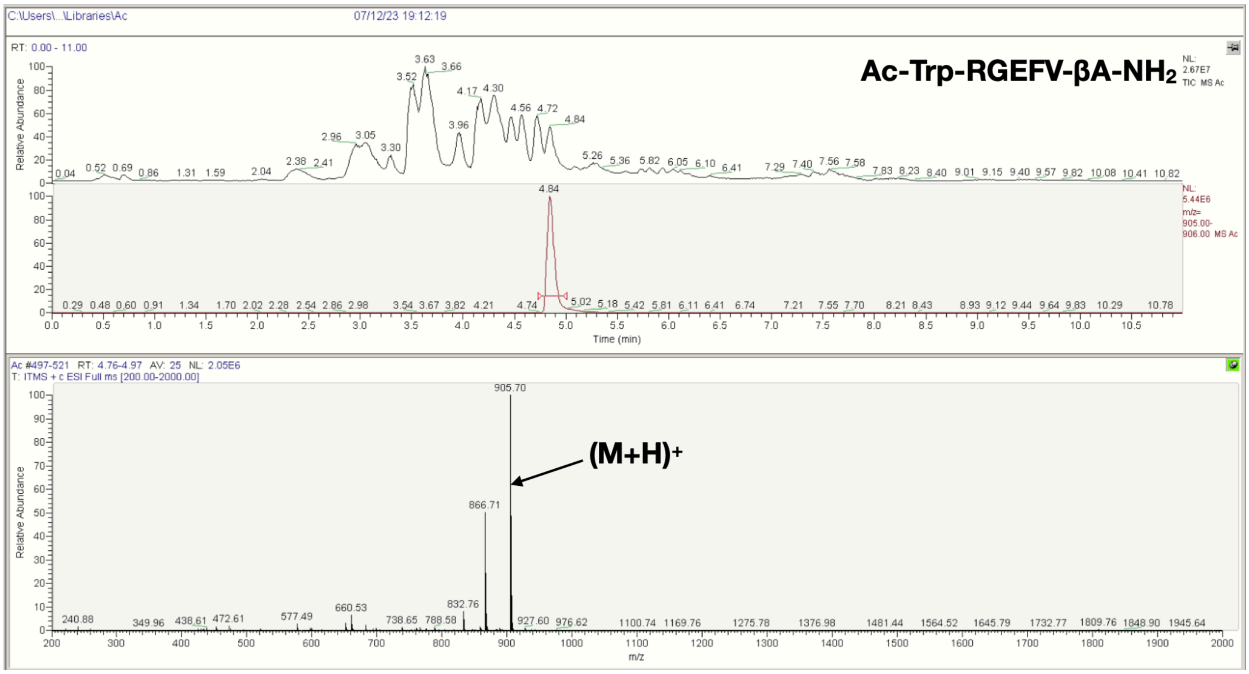
Ac-Trp-RGEFV-βA-NH2
(M+H)+
905.70
866.71
832.76
660.53
4.84
Time (min)
m/z
Relative Abundance

Q

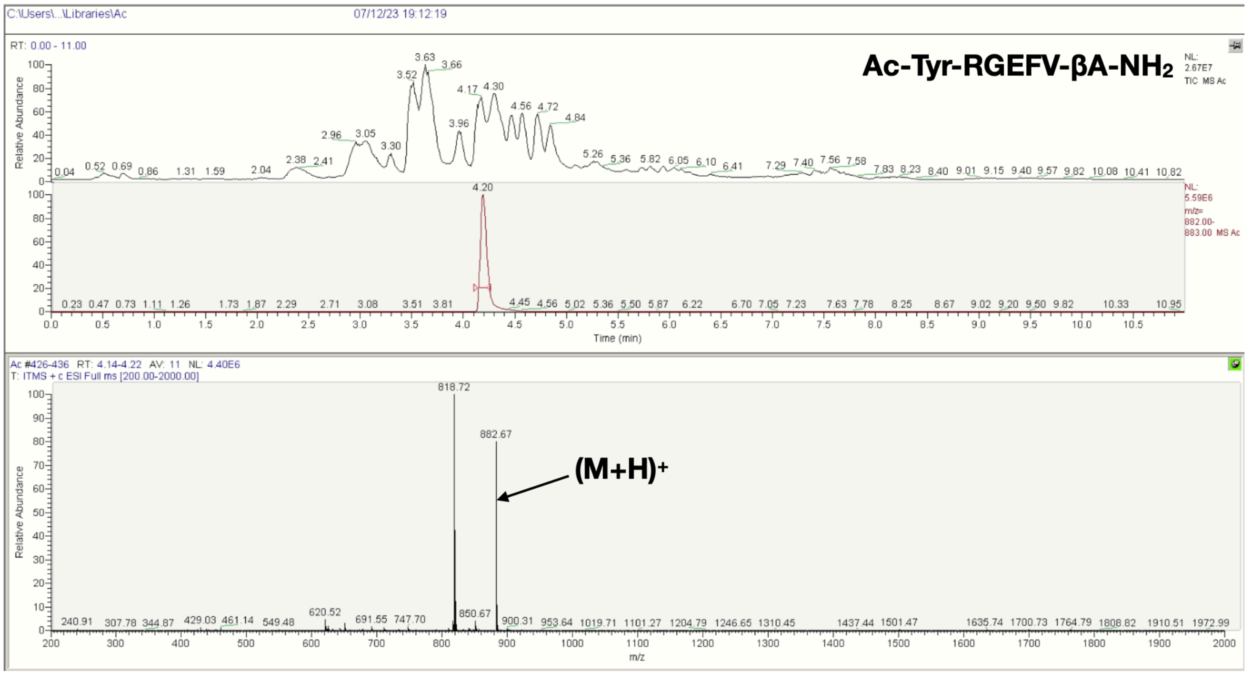

R

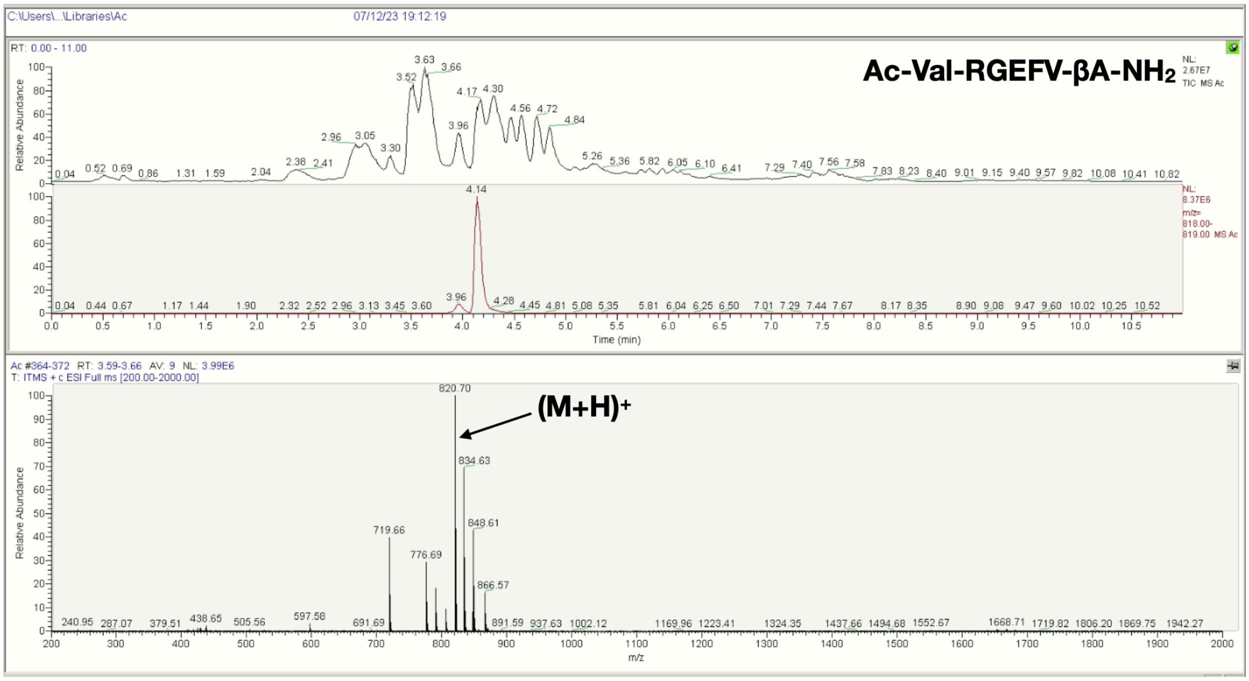

Figure S12. LCMS spectra of the Ac-X-RGEFV-βA-NH_2 libraries, where X = a) Ala, b) Arg, c) Asn, d) Asp, e) Gln, f) Glu, g) Gly, h) His, i) Ile/Leu, j) Lys, k) Met, l) Phe, m) Pro, n) Ser, o) Thr, p) Trp, q) Tyr, r) Val.

A

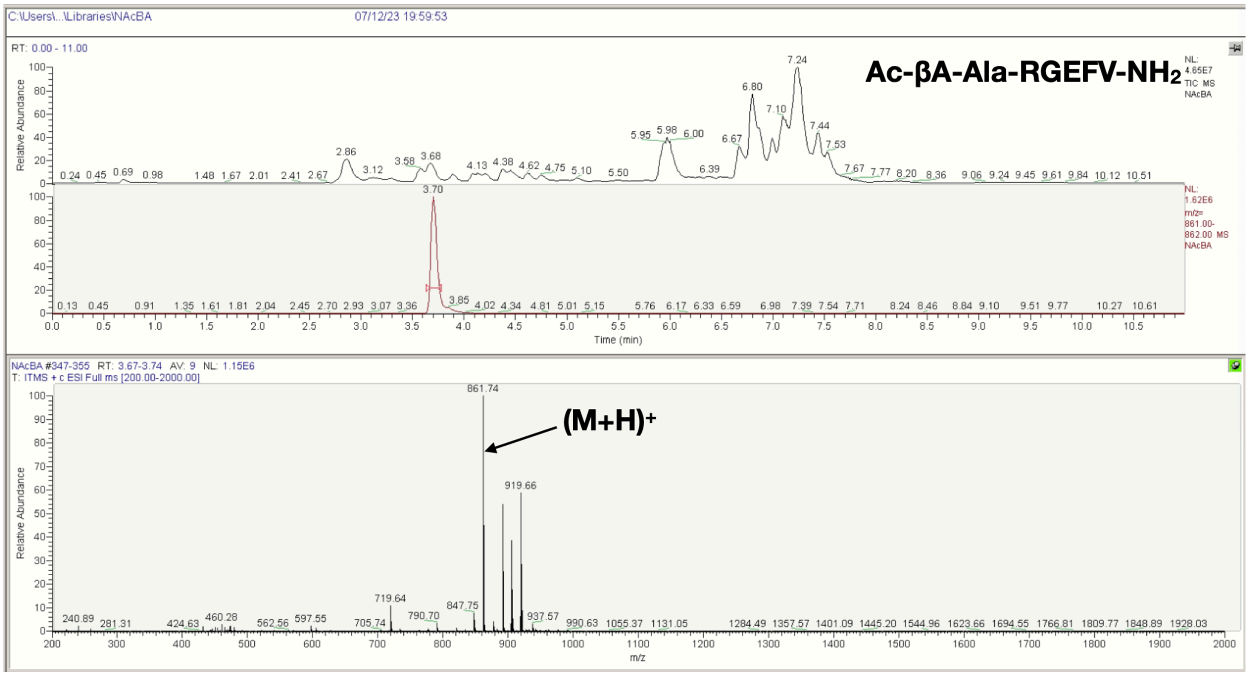

C:\Users\...\Libraries\NAcBA
07/12/23 19:59:53
Ac-βA-Ala-RGEFV-NH2
Relative Abundance
Time (min)
NAcBA #347-355 RT: 3.67-3.74 AV: 9 NL: 1.15E6
T: ITMS + c ESI Full ms [200.00-2000.00]
(M+H)+
861.74
919.66
m/z

B

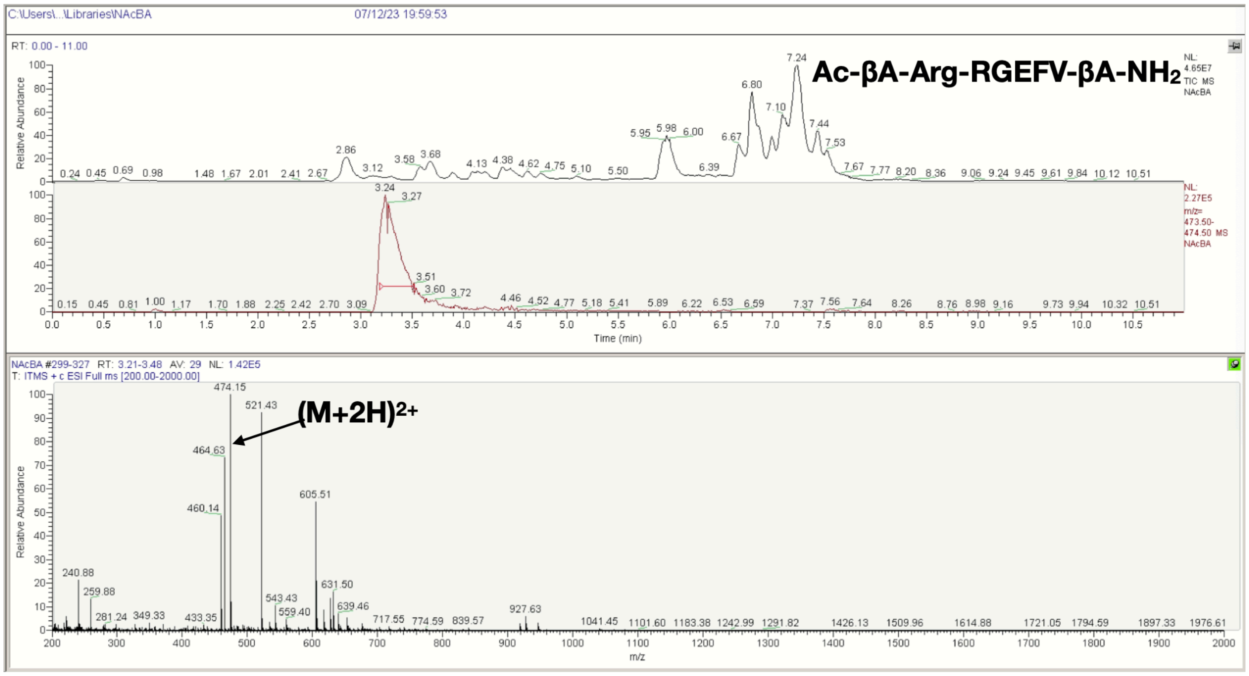

C:\Users\...\Libraries\NAcBA
07/12/23 19:59:53
Ac-βA-Arg-RGEFV-βA-NH2
Relative Abundance
Time (min)
NAcBA #299-327 RT: 3.21-3.48 AV: 29 NL: 1.42E5
T: ITMS + c ESI Full ms [200.00-2000.00]
(M+2H)2+
474.15
521.43
464.63
605.51
m/z

C

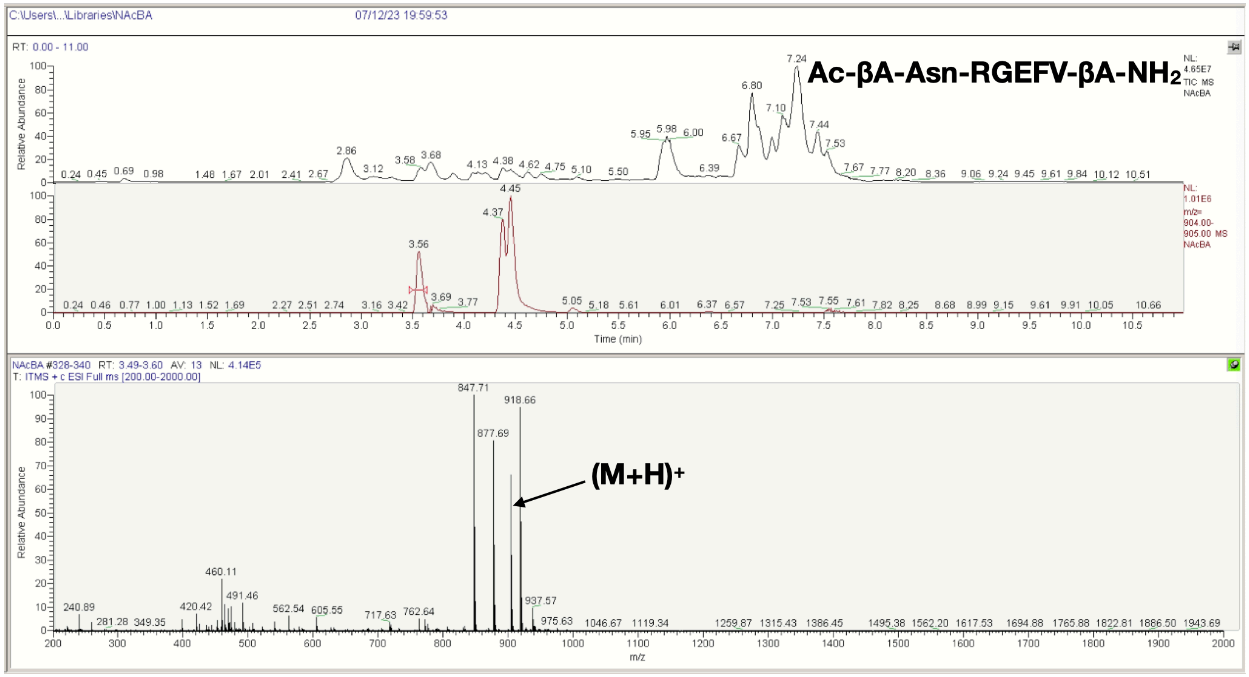
C:\Users\...\Libraries\NAcBA
07/12/23 19:59:53
Ac-βA-Asn-RGEFV-βA-NH2
(M+H)+
NAcBA #328-340 RT: 3.49-3.60 AV: 13 NL: 4.14E5
T: ITMS + c ESI Full ms [200.00-2000.00]
Relative Abundance
Time (min)
m/z

D

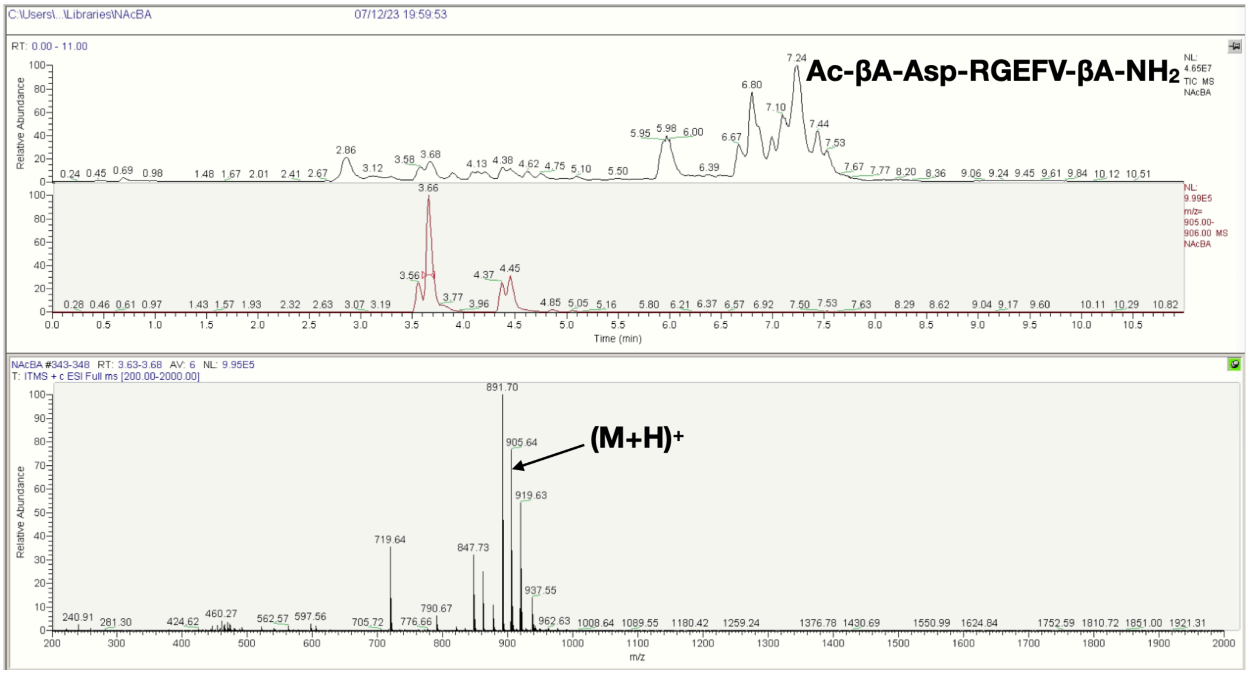
C:\Users\...\Libraries\NAcBA
07/12/23 19:59:53
Ac-βA-Asp-RGEFV-βA-NH2
(M+H)+
NAcBA #343-348 RT: 3.63-3.68 AV: 6 NL: 9.95E5
T: ITMS + c ESI Full ms [200.00-2000.00]
Relative Abundance
Time (min)
m/z

E

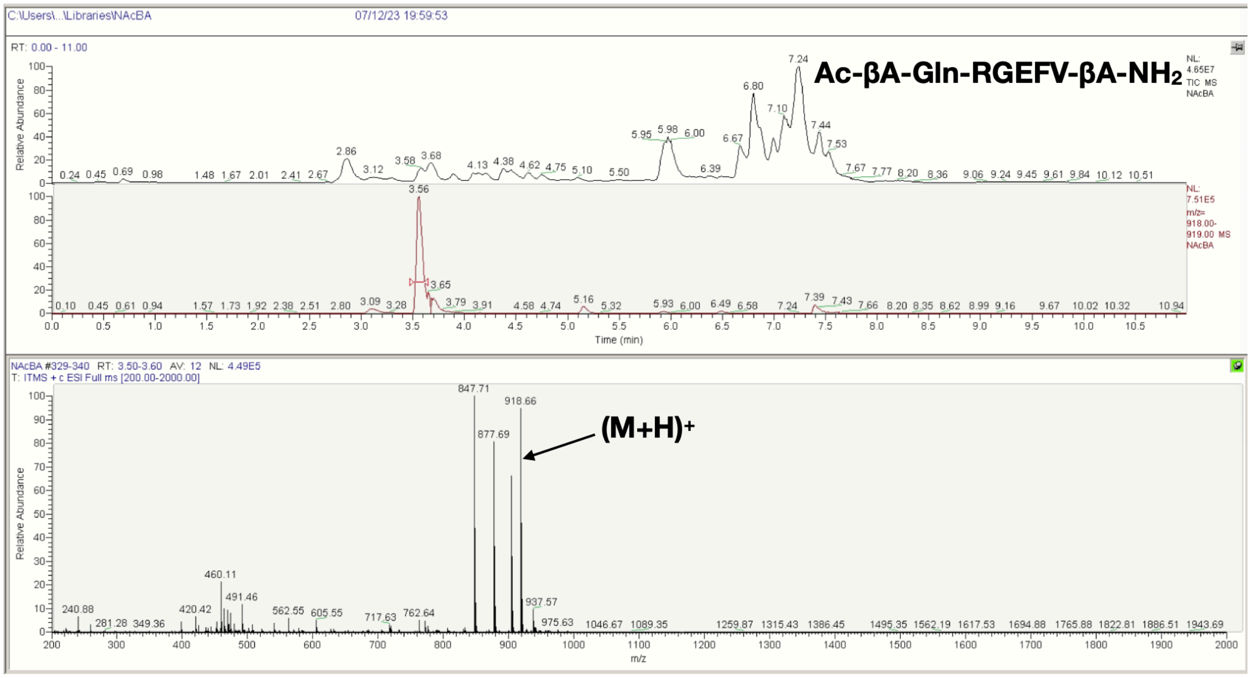
C:\Users\...\Libraries\NAcBA
07/12/23 19:59:53
RT: 0.00 - 11.00
Ac-βA-Gln-RGEFV-βA-NH2
NL: 4.65E7 TIC MS NAcBA
NL: 7.51E5 m/z= 918.00-919.00 MS NAcBA
Relative Abundance
Time (min)
NAcBA #329-340 RT: 3.50-3.60 AV: 12 NL: 4.49E5
T: ITMS + c ESI Full ms [200.00-2000.00]
(M+H)+
847.71
877.69
918.66
m/z

F

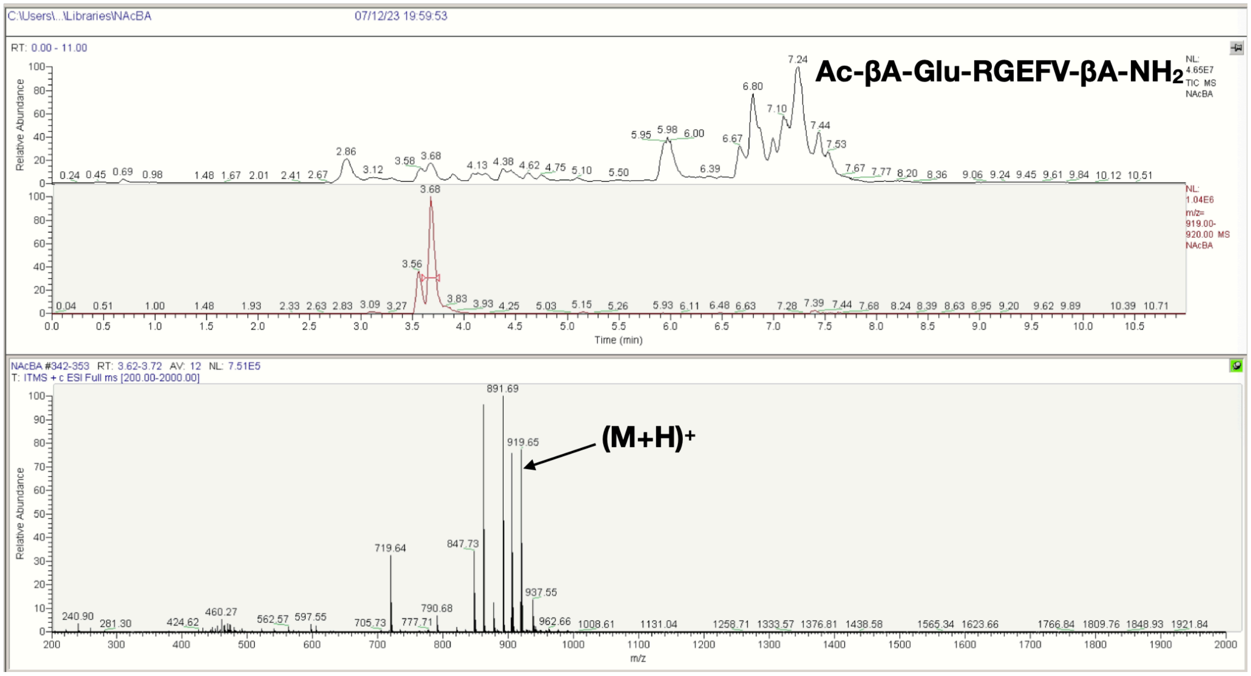
C:\Users\...\Libraries\NAcBA
07/12/23 19:59:53
RT: 0.00 - 11.00
Ac-βA-Glu-RGEFV-βA-NH2
NL: 4.65E7 TIC MS NAcBA
NL: 1.04E6 m/z= 919.00-920.00 MS NAcBA
Relative Abundance
Time (min)
NAcBA #342-353 RT: 3.62-3.72 AV: 12 NL: 7.51E5
T: ITMS + c ESI Full ms [200.00-2000.00]
(M+H)+
891.69
919.65
719.64
847.73
m/z

G

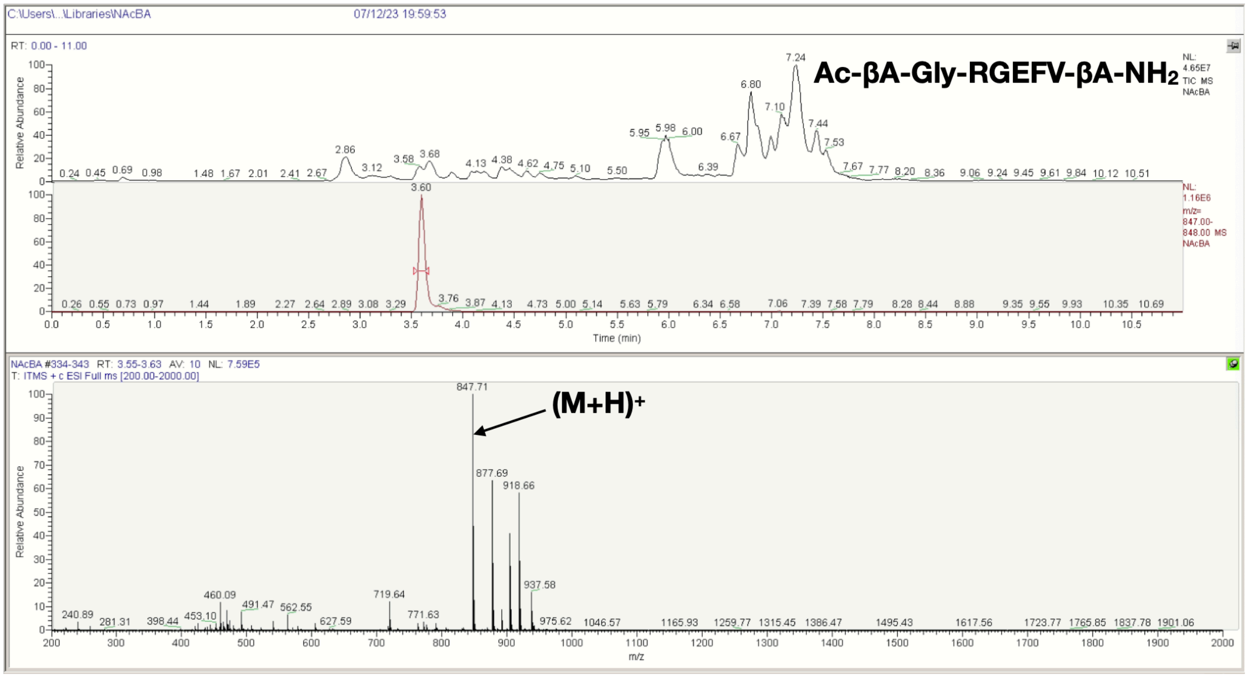
C:\Users\...\Libraries\NAcBA
07/12/23 19:59:53
Ac-βA-Gly-RGEFV-βA-NH2
Time (min)
NAcBA #334-343 RT: 3.55-3.63 AV: 10 NL: 7.59E5
T: ITMS + c ESI Full ms [200.00-2000.00]
(M+H)+
847.71
Relative Abundance
m/z

H

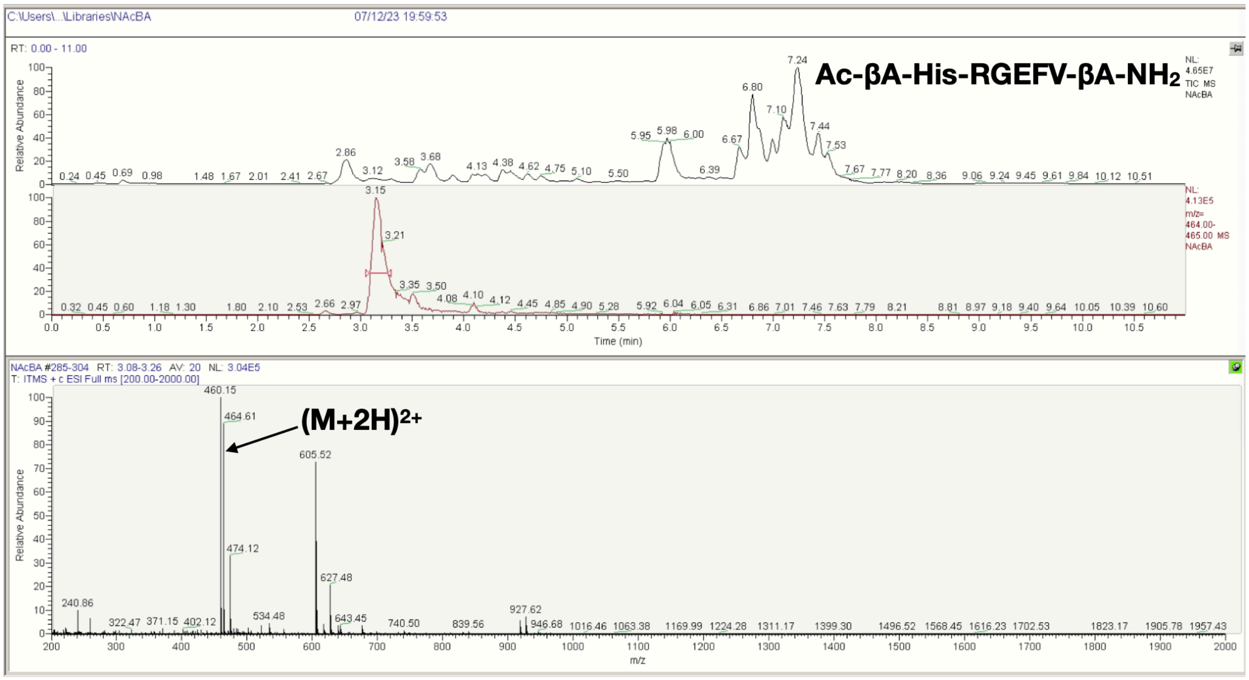
C:\Users\...\Libraries\NAcBA
07/12/23 19:59:53
Ac-βA-His-RGEFV-βA-NH2
Time (min)
NAcBA #285-304 RT: 3.08-3.26 AV: 20 NL: 3.04E5
T: ITMS + c ESI Full ms [200.00-2000.00]
(M+2H)2+
464.61
Relative Abundance
m/z

I

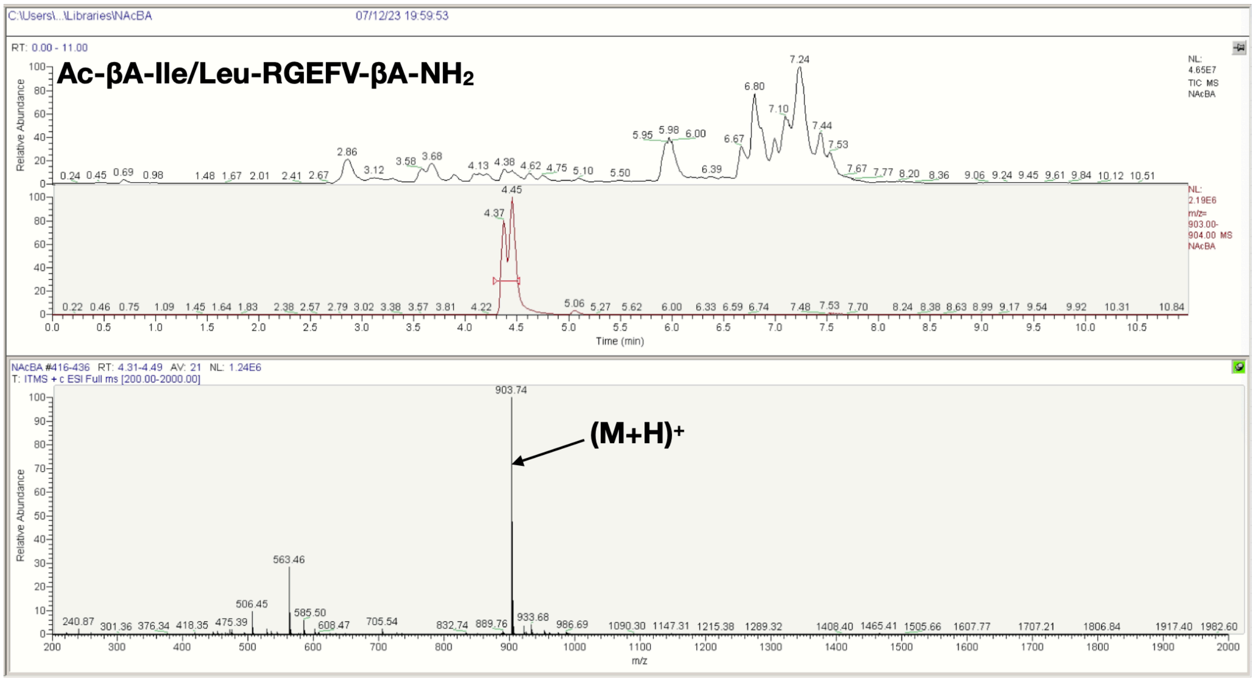
C:\Users\...\Libraries\NAcBA
07/12/23 19:59:53
RT: 0.00 - 11.00
Ac-βA-Ile/Leu-RGEFV-βA-NH2
NL: 4.65E7 TIC MS NAcBA
NL: 2.19E6 m/z= 903.00-904.00 MS NAcBA
Relative Abundance
Time (min)
NAcBA #416-436 RT: 4.31-4.49 AV: 21 NL: 1.24E6
T: ITMS + c ESI Full ms [200.00-2000.00]
903.74
(M+H)+
563.46
m/z

J

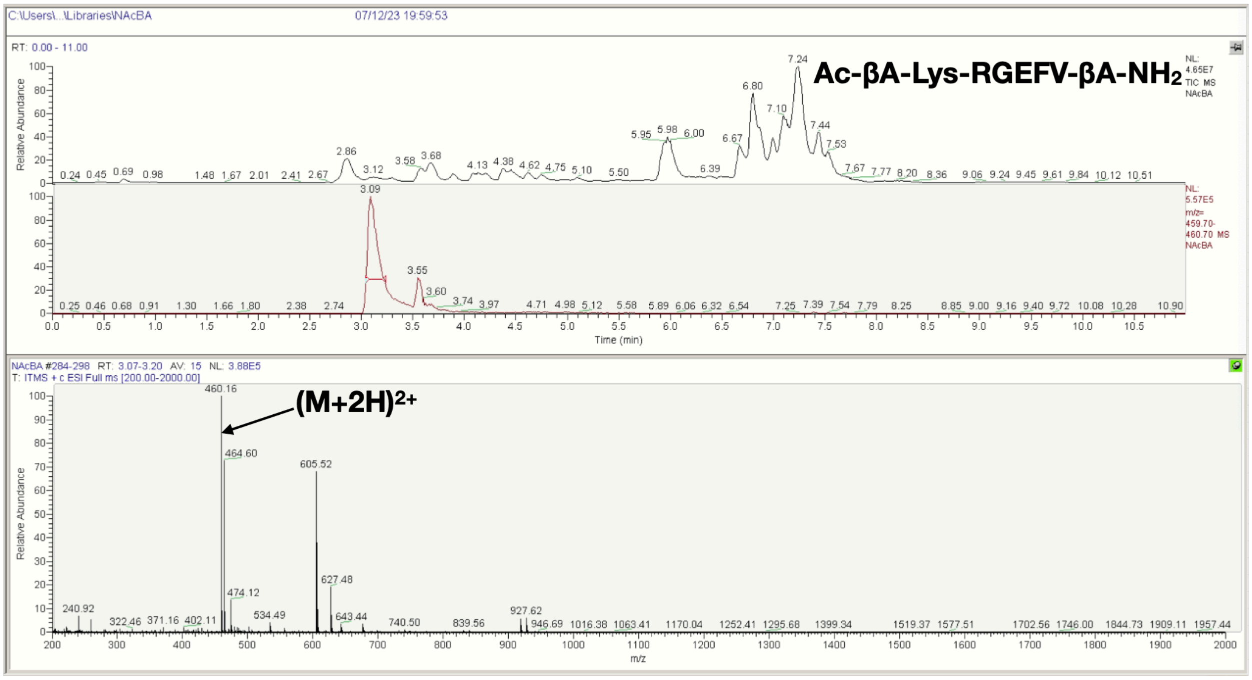
C:\Users\...\Libraries\NAcBA
07/12/23 19:59:53
RT: 0.00 - 11.00
Ac-βA-Lys-RGEFV-βA-NH2
NL: 4.65E7 TIC MS NAcBA
NL: 5.57E5 m/z= 459.70-460.70 MS NAcBA
Relative Abundance
Time (min)
NAcBA #284-298 RT: 3.07-3.20 AV: 15 NL: 3.88E5
T: ITMS + c ESI Full ms [200.00-2000.00]
460.16
(M+2H)2+
464.60
605.52
m/z

K

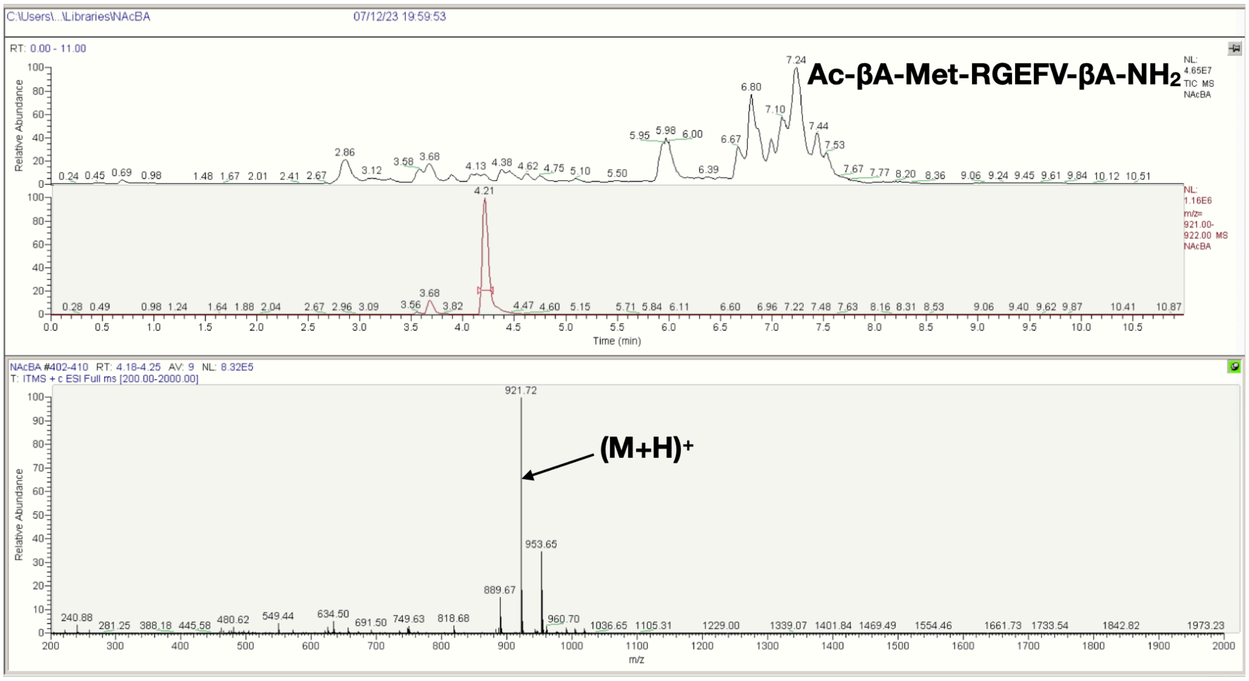

L

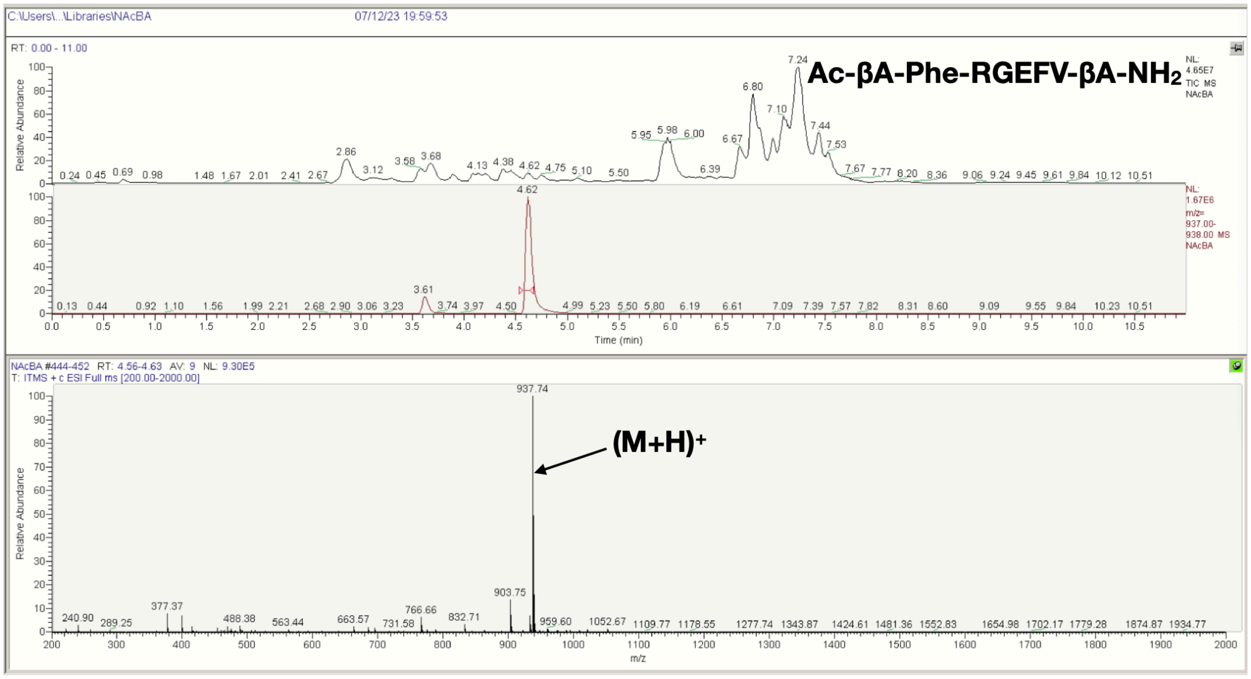

M

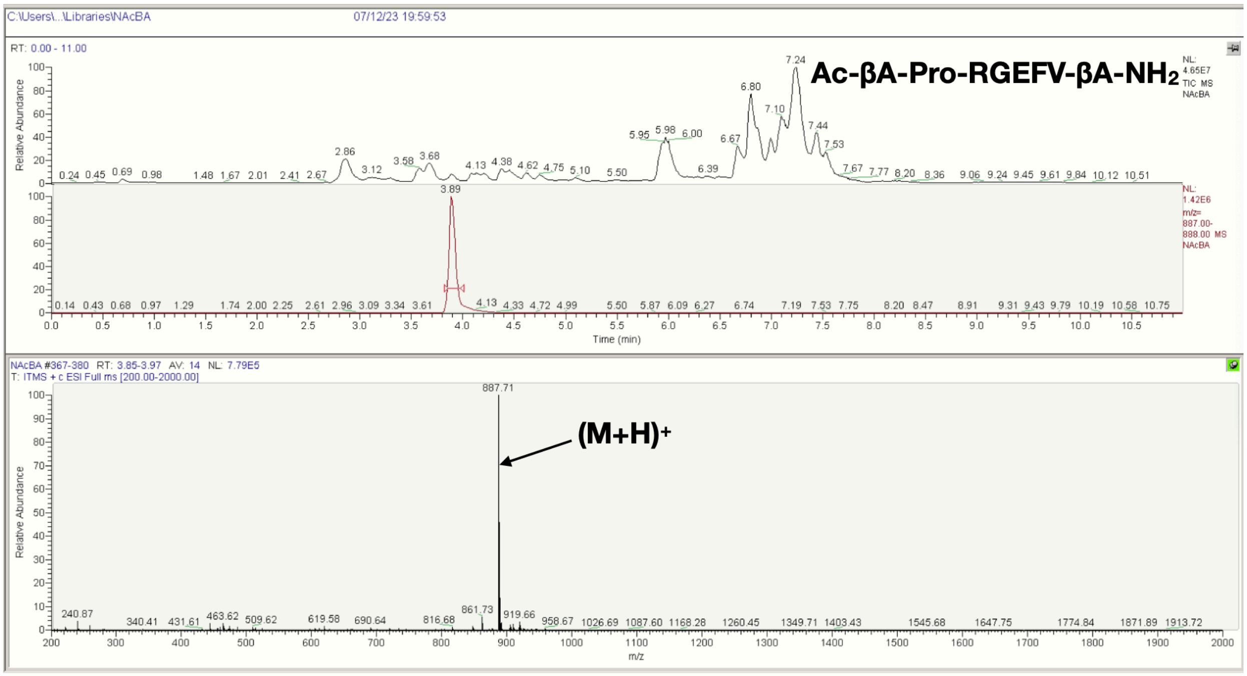
C:\Users\...\Libraries\NAcBA
07/12/23 19:59:53
Ac-βA-Pro-RGEFV-βA-NH2
Time (min)
Relative Abundance
NAcBA #367-380 RT: 3.85-3.97 AV: 14 NL: 7.79E5
T: ITMS + c ESI Full ms [200.00-2000.00]
887.71
(M+H)+
m/z

N

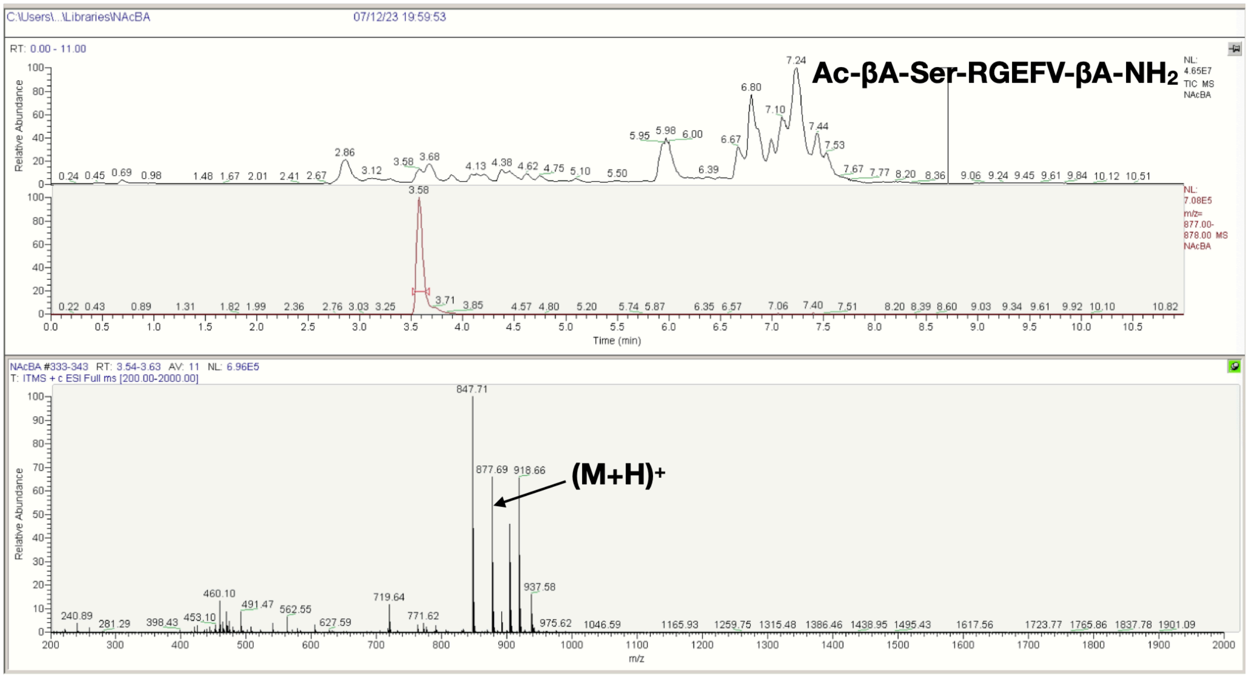
C:\Users\...\Libraries\NAcBA
07/12/23 19:59:53
Ac-βA-Ser-RGEFV-βA-NH2
Time (min)
Relative Abundance
NAcBA #333-343 RT: 3.54-3.63 AV: 11 NL: 6.96E5
T: ITMS + c ESI Full ms [200.00-2000.00]
847.71
877.69
918.66
(M+H)+
m/z

O

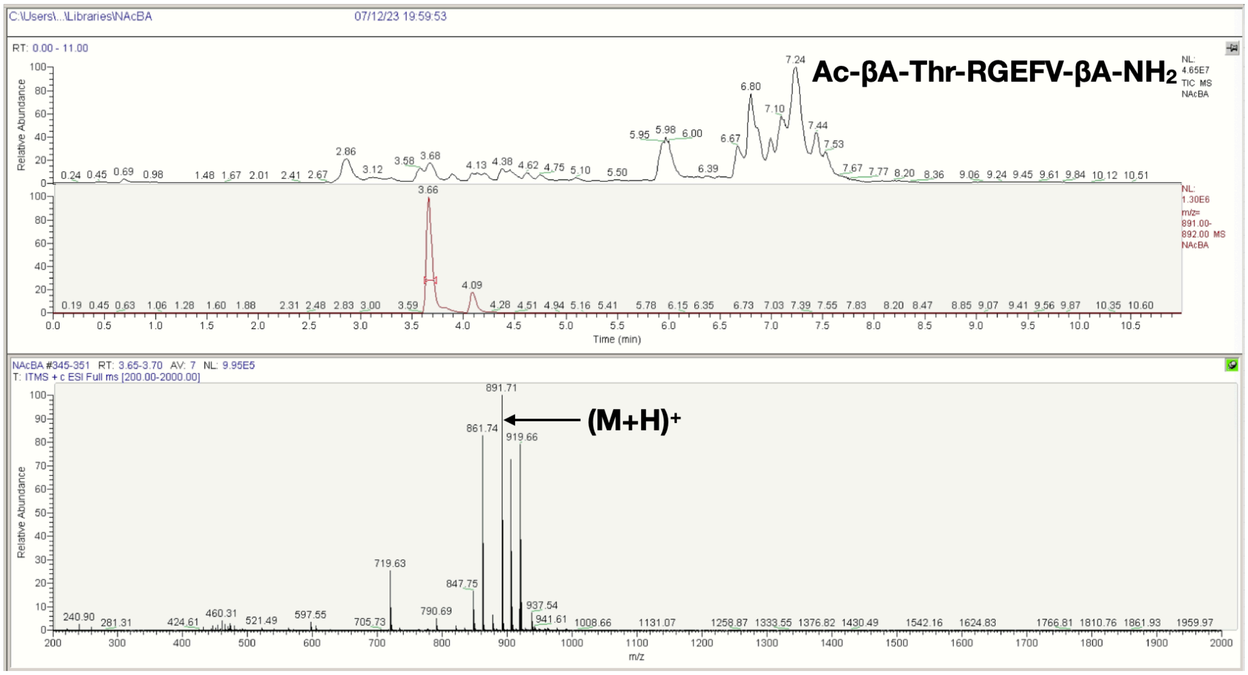

C:\Users\...\Libraries\NAcBA
07/12/23 19:59:53
RT: 0.00 - 11.00
Ac-βA-Thr-RGEFV-βA-NH2
NL: 4.65E7 TIC MS NAcBA
NL: 1.30E6 m/z= 891.00-892.00 MS NAcBA
Relative Abundance
Time (min)
NAcBA #345-351 RT: 3.65-3.70 AV: 7 NL: 9.95E5
T: ITMS + c ESI Full ms [200.00-2000.00]
891.71
(M+H)+
861.74
919.66
719.63
m/z

P

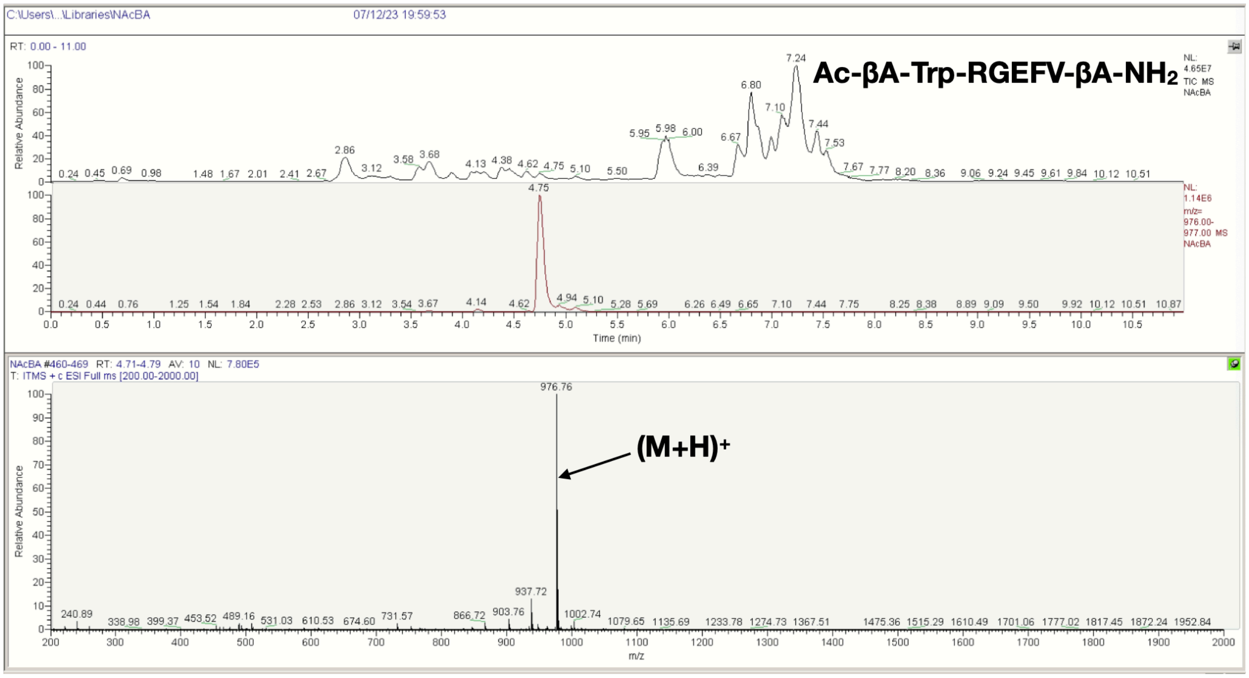

C:\Users\...\Libraries\NAcBA
07/12/23 19:59:53
RT: 0.00 - 11.00
Ac-βA-Trp-RGEFV-βA-NH2
NL: 4.65E7 TIC MS NAcBA
NL: 1.14E6 m/z= 976.00-977.00 MS NAcBA
Relative Abundance
Time (min)
NAcBA #460-469 RT: 4.71-4.79 AV: 10 NL: 7.80E5
T: ITMS + c ESI Full ms [200.00-2000.00]
976.76
(M+H)+
937.72
m/z

Q

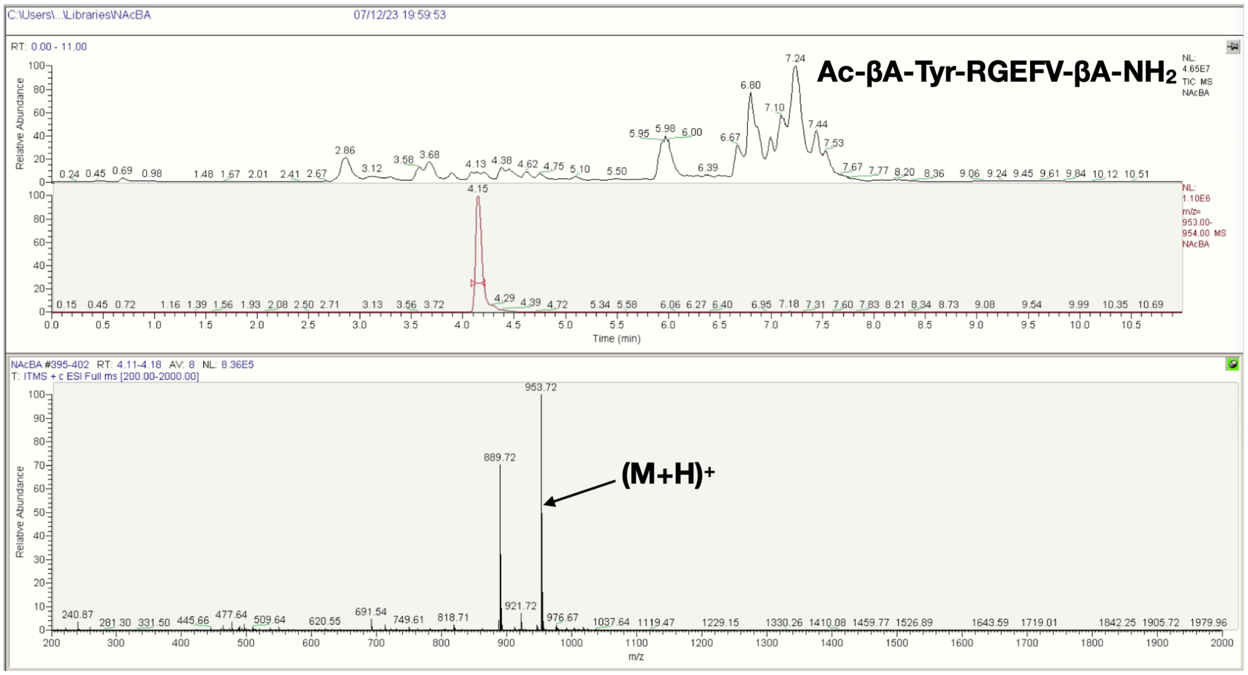

R

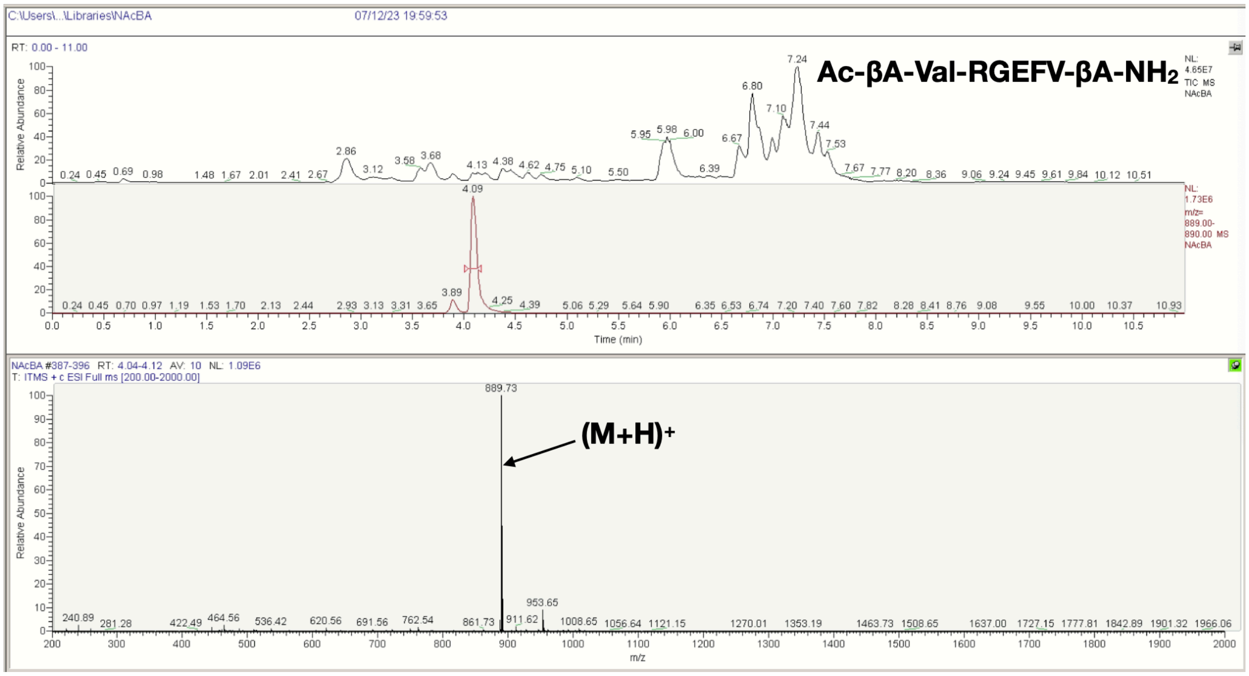

Figure S13. LCMS spectra of the Ac-βA-X-RGEFV-βA-NH_2 libraries, where X = a) Ala, b) Arg, c) Asn, d) Asp, e) Gln, f) Glu, g) Gly, h) His, i) Ile/Leu, j) Lys, k) Met, l) Phe, m) Pro, n) Ser, o) Thr, p) Trp, q) Tyr, r) Val.

A

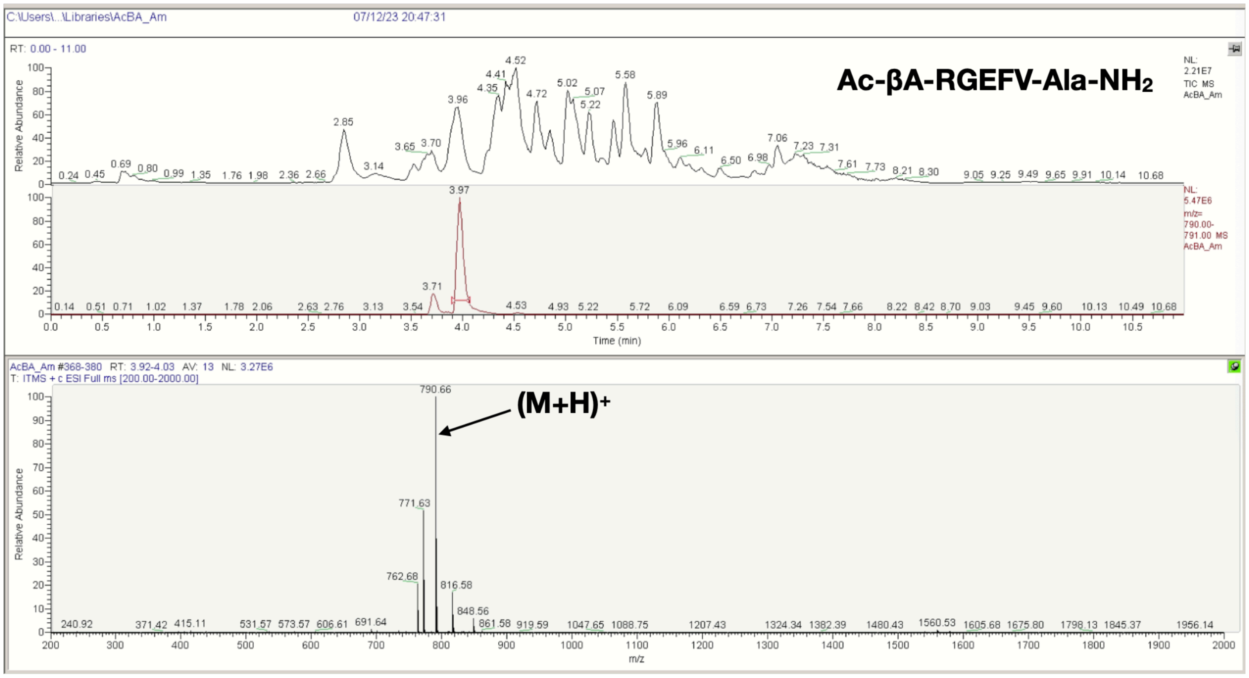
C:\Users\...\Libraries\AcBA_Am
07/12/23 20:47:31
RT: 0.00 - 11.00
Ac-βA-RGEFV-Ala-NH2
NL: 2.21E7 TIC MS AcBA_Am
NL: 5.47E6 m/z= 790.00-791.00 MS AcBA_Am
Relative Abundance
Time (min)
AcBA_Am #368-380 RT: 3.92-4.03 AV: 13 NL: 3.27E6
T: ITMS + c ESI Full ms [200.00-2000.00]
790.66
(M+H)+
771.63
762.68
816.58
848.56
m/z

B

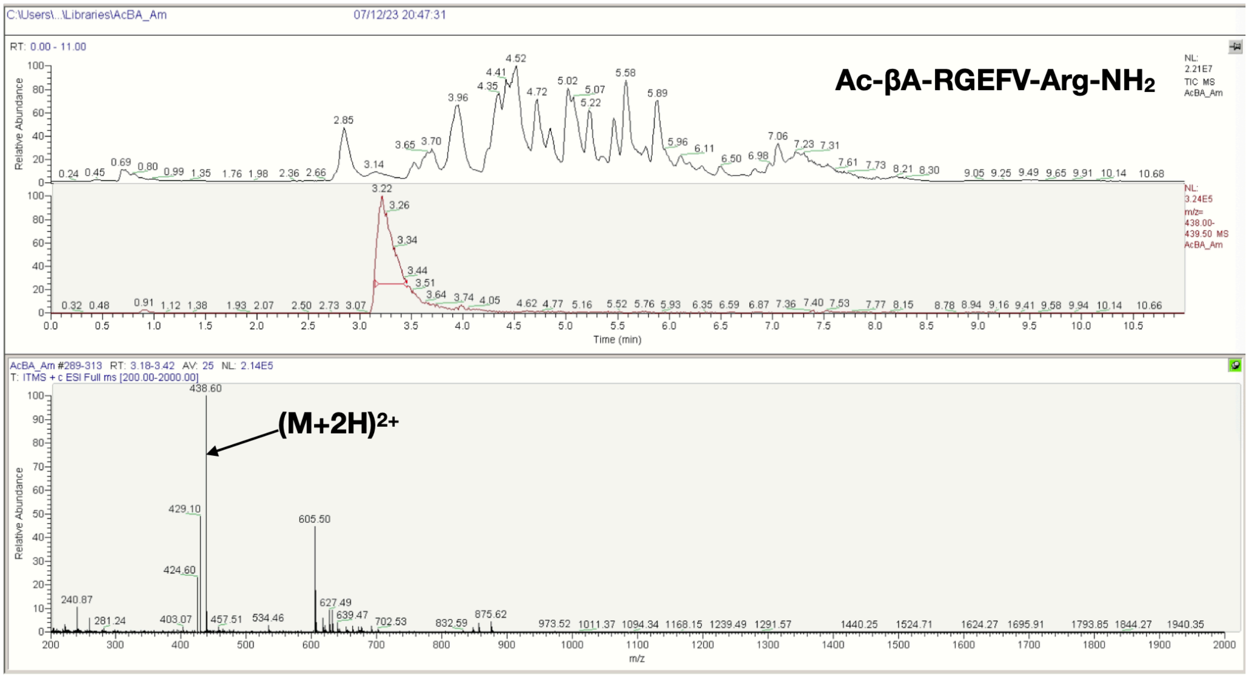
C:\Users\...\Libraries\AcBA_Am
07/12/23 20:47:31
RT: 0.00 - 11.00
Ac-βA-RGEFV-Arg-NH2
NL: 2.21E7 TIC MS AcBA_Am
NL: 3.24E5 m/z= 438.00-439.50 MS AcBA_Am
Relative Abundance
Time (min)
AcBA_Am #289-313 RT: 3.18-3.42 AV: 25 NL: 2.14E5
T: ITMS + c ESI Full ms [200.00-2000.00]
438.60
(M+2H)2+
429.10
424.60
605.50
m/z

C

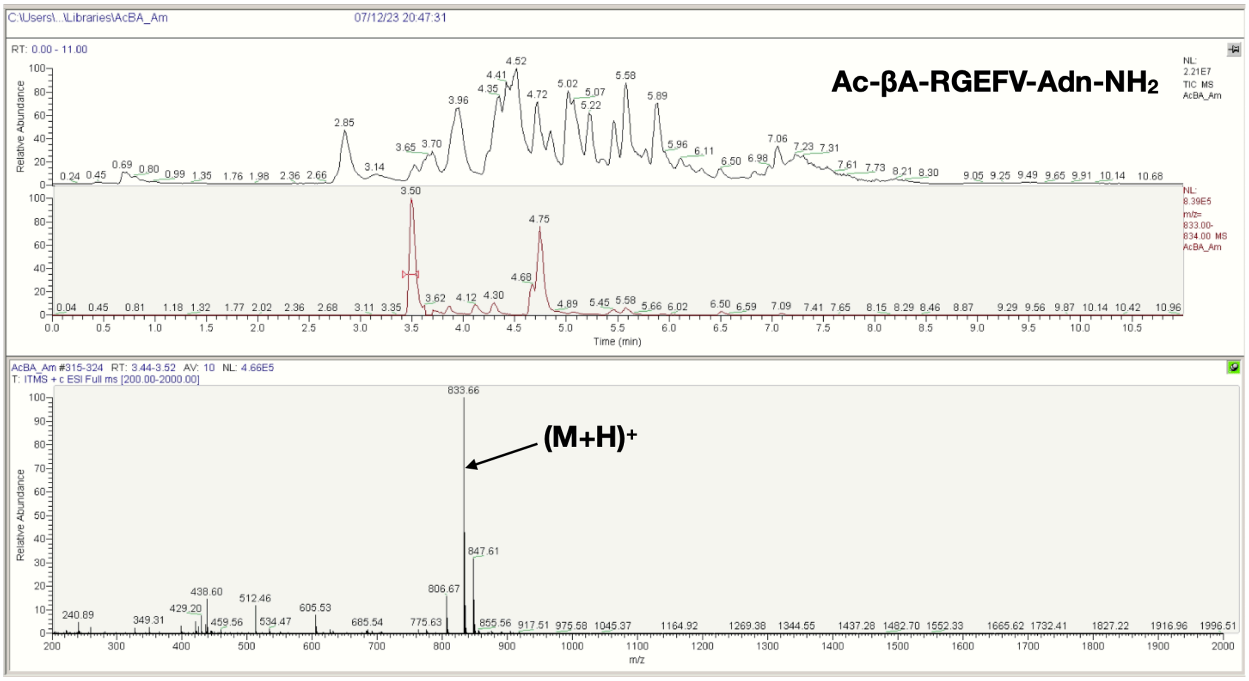
C:\Users\...\Libraries\AcBA_Am
07/12/23 20:47:31
Ac-βA-RGEFV-Adn-NH2
RT: 0.00 - 11.00
Relative Abundance
Time (min)
AcBA_Am #315-324 RT: 3.44-3.52 AV: 10 NL: 4.66E5
T: ITMS + c ESI Full ms [200.00-2000.00]
833.66
(M+H)+
847.61
806.67
m/z

D

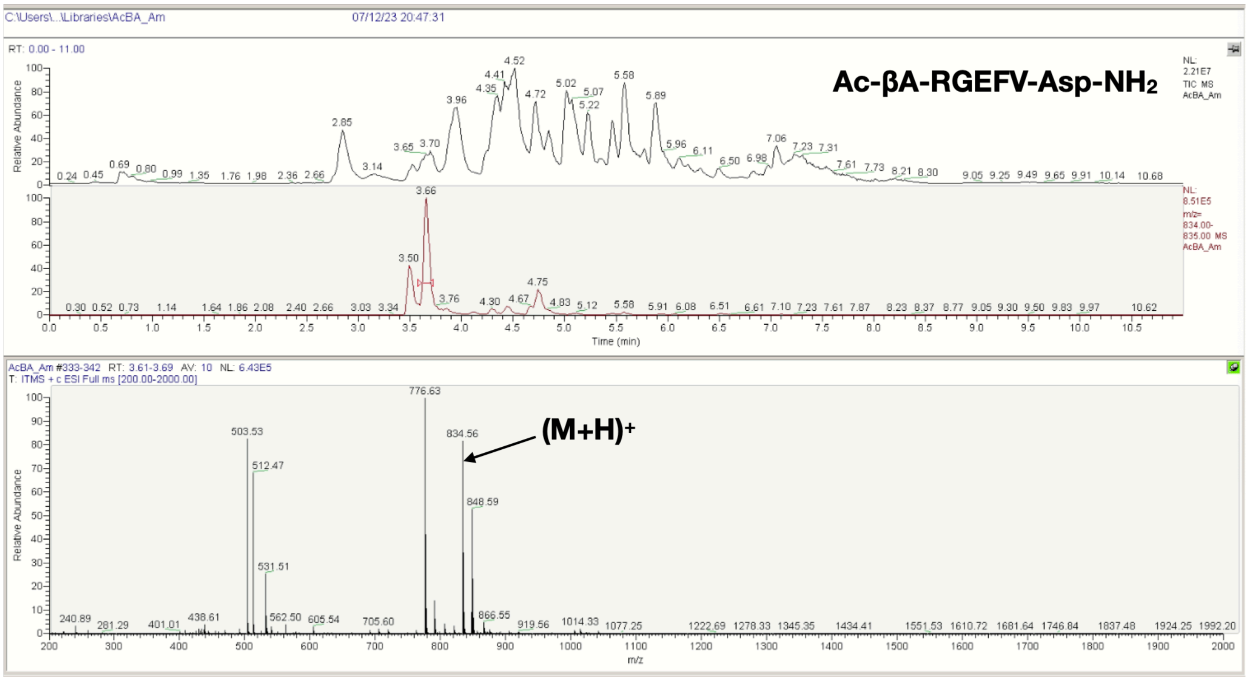
C:\Users\...\Libraries\AcBA_Am
07/12/23 20:47:31
Ac-βA-RGEFV-Asp-NH2
RT: 0.00 - 11.00
Relative Abundance
Time (min)
AcBA_Am #333-342 RT: 3.61-3.69 AV: 10 NL: 6.43E5
T: ITMS + c ESI Full ms [200.00-2000.00]
776.63
834.56
(M+H)+
848.59
503.53
512.47
m/z

E

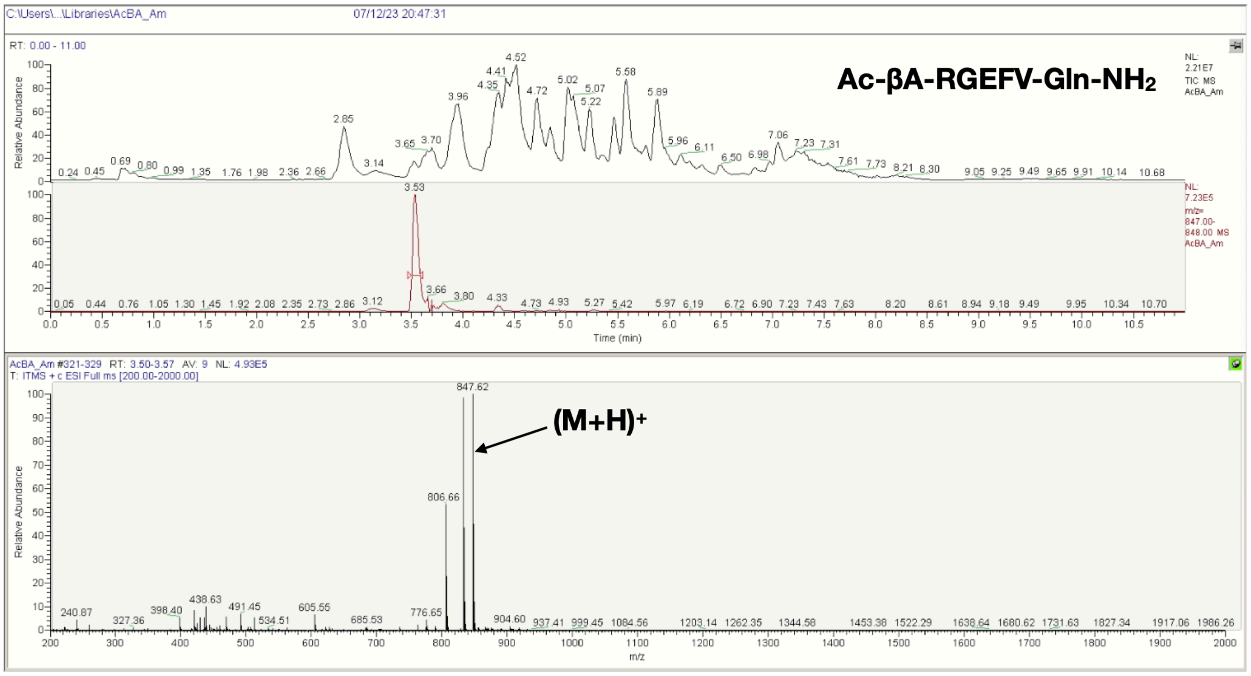
C:\Users\...\Libraries\AcBA_Am
07/12/23 20:47:31
RT: 0.00 - 11.00
Ac-βA-RGEFV-Gln-NH2
NL: 2.21E7 TIC MS AcBA_Am
NL: 7.23E5 m/z= 847.00-848.00 MS AcBA_Am
Relative Abundance
Time (min)
AcBA_Am #321-329 RT: 3.50-3.57 AV: 9 NL: 4.93E5
T: ITMS + c ESI Full ms [200.00-2000.00]
847.62
806.66
(M+H)+
m/z

F

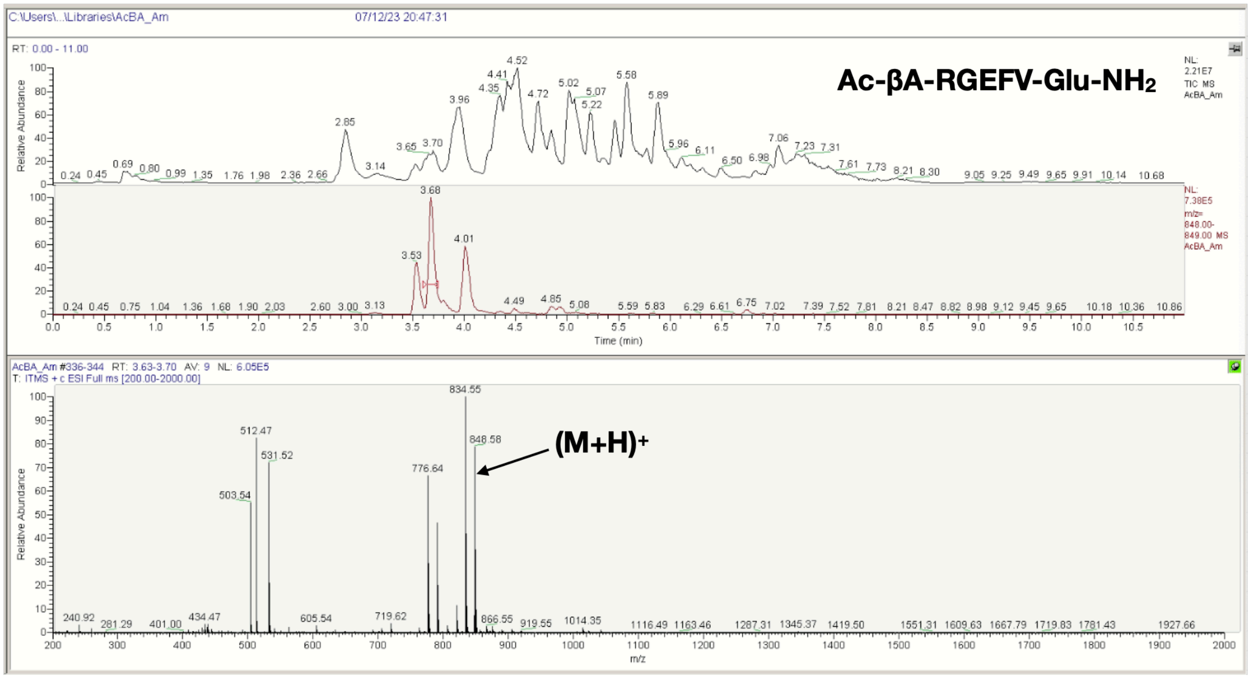
C:\Users\...\Libraries\AcBA_Am
07/12/23 20:47:31
RT: 0.00 - 11.00
Ac-βA-RGEFV-Glu-NH2
NL: 2.21E7 TIC MS AcBA_Am
NL: 7.38E5 m/z= 848.00-849.00 MS AcBA_Am
Relative Abundance
Time (min)
AcBA_Am #336-344 RT: 3.63-3.70 AV: 9 NL: 6.05E5
T: ITMS + c ESI Full ms [200.00-2000.00]
834.55
848.58
512.47
531.52
503.54
776.64
(M+H)+
m/z

G

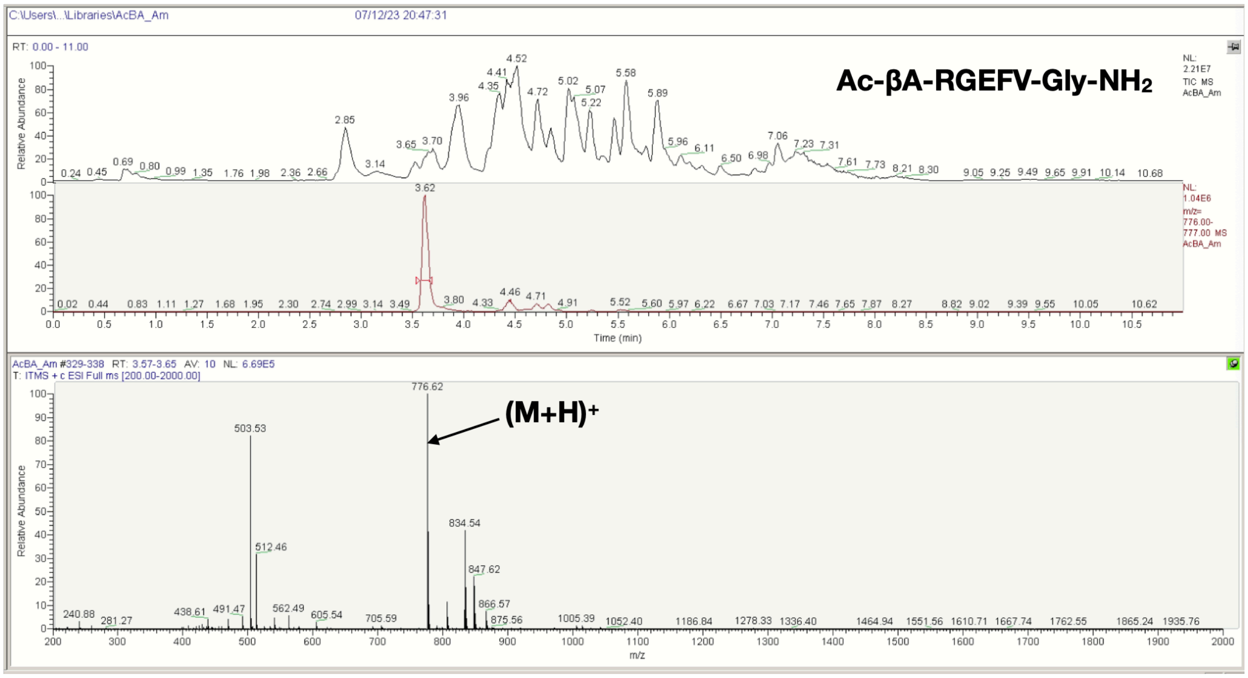

Ac-βA-RGEFV-Gly-NH2
(M+H)+
Relative Abundance
Time (min)
m/z

H

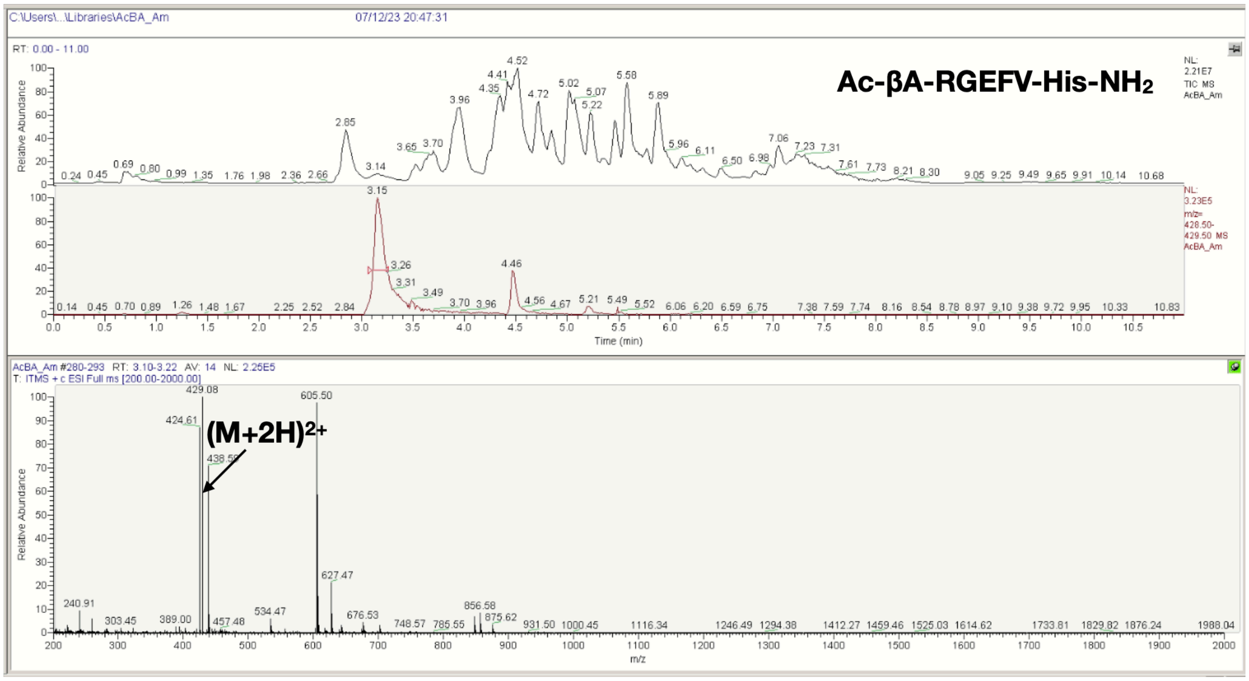

Ac-βA-RGEFV-His-NH2
(M+2H)2+
Relative Abundance
Time (min)
m/z

I

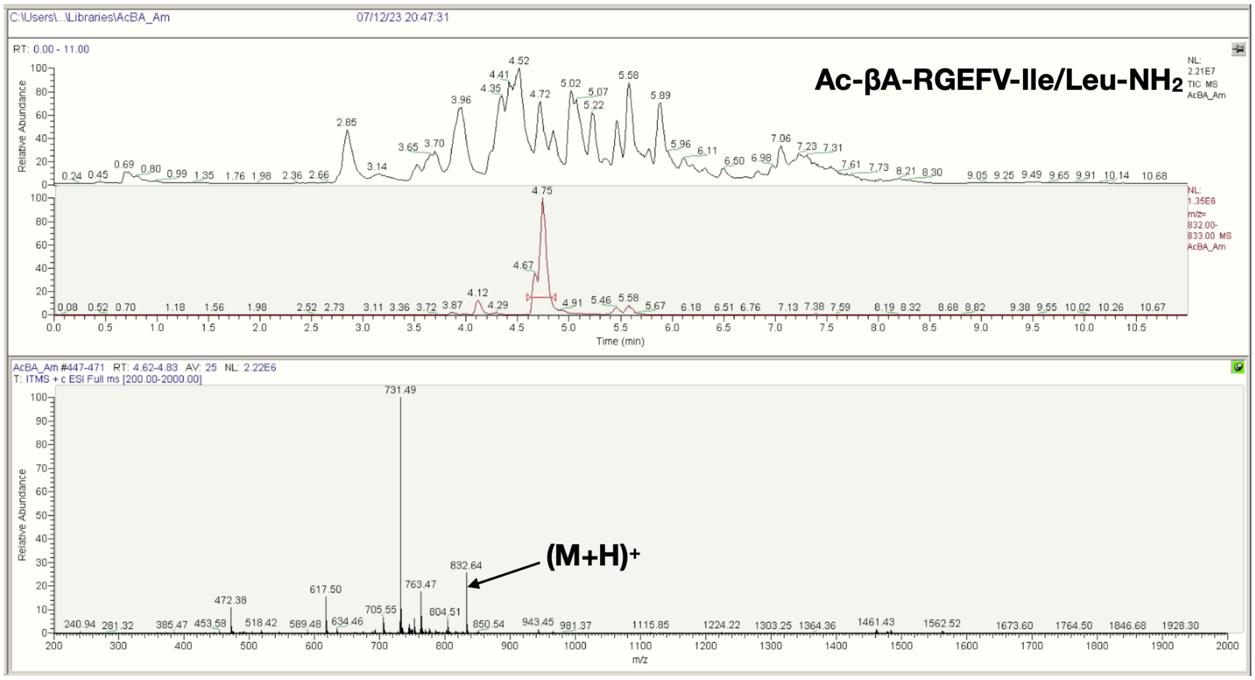
C:\Users\...\Libraries\AcBA_Am
07/12/23 20:47:31
RT: 0.00 - 11.00
Ac-βA-RGEFV-Ile/Leu-NH2
NL: 2.21E7 TIC MS AcBA_Am
NL: 1.35E6 m/z= 832.00-833.00 MS AcBA_Am
Relative Abundance
Time (min)
AcBA_Am #447-471 RT: 4.62-4.83 AV: 25 NL: 2.22E6
T: ITMS + c ESI Full ms [200.00-2000.00]
731.49
832.64
(M+H)+
m/z

J

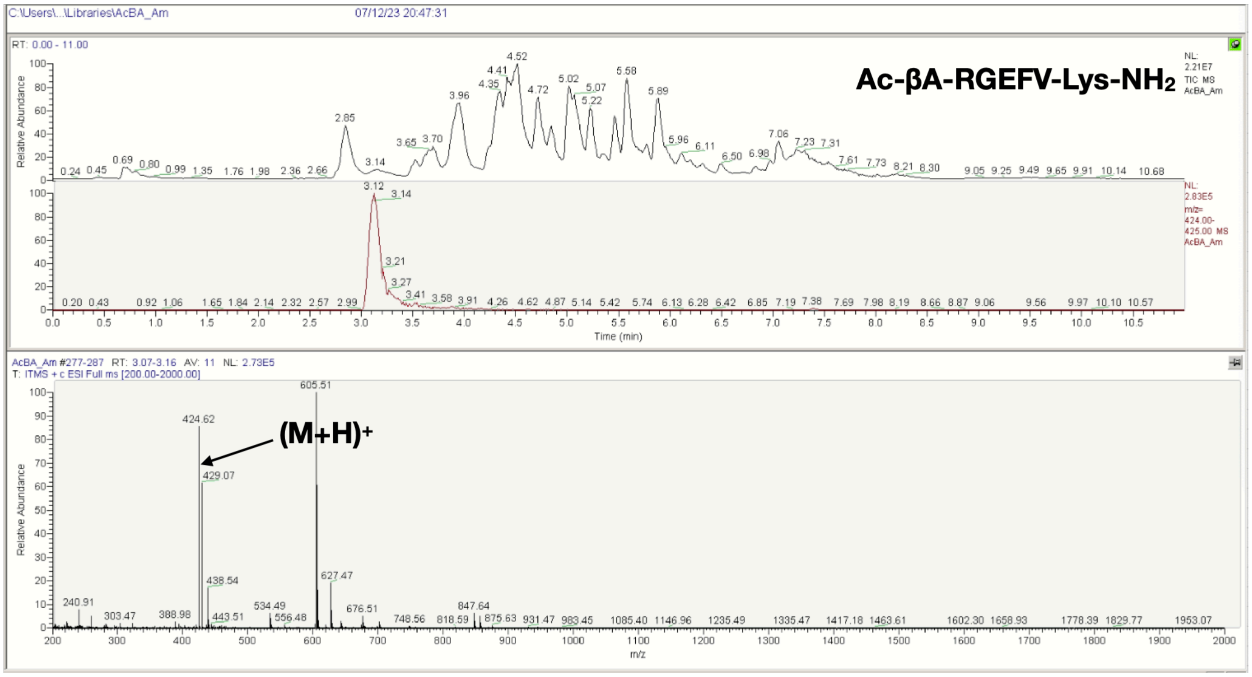
C:\Users\...\Libraries\AcBA_Am
07/12/23 20:47:31
RT: 0.00 - 11.00
Ac-βA-RGEFV-Lys-NH2
NL: 2.21E7 TIC MS AcBA_Am
NL: 2.83E5 m/z= 424.00-425.00 MS AcBA_Am
Relative Abundance
Time (min)
AcBA_Am #277-287 RT: 3.07-3.16 AV: 11 NL: 2.73E5
T: ITMS + c ESI Full ms [200.00-2000.00]
605.51
424.62
(M+H)+
m/z

K

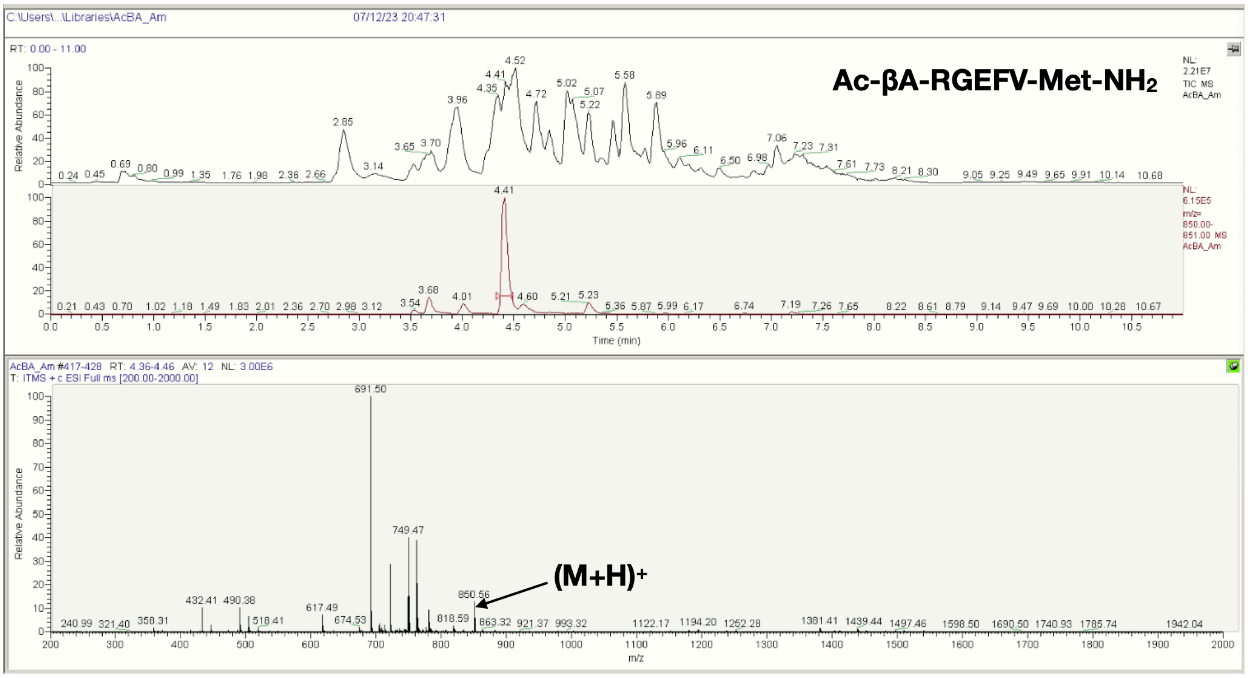

L

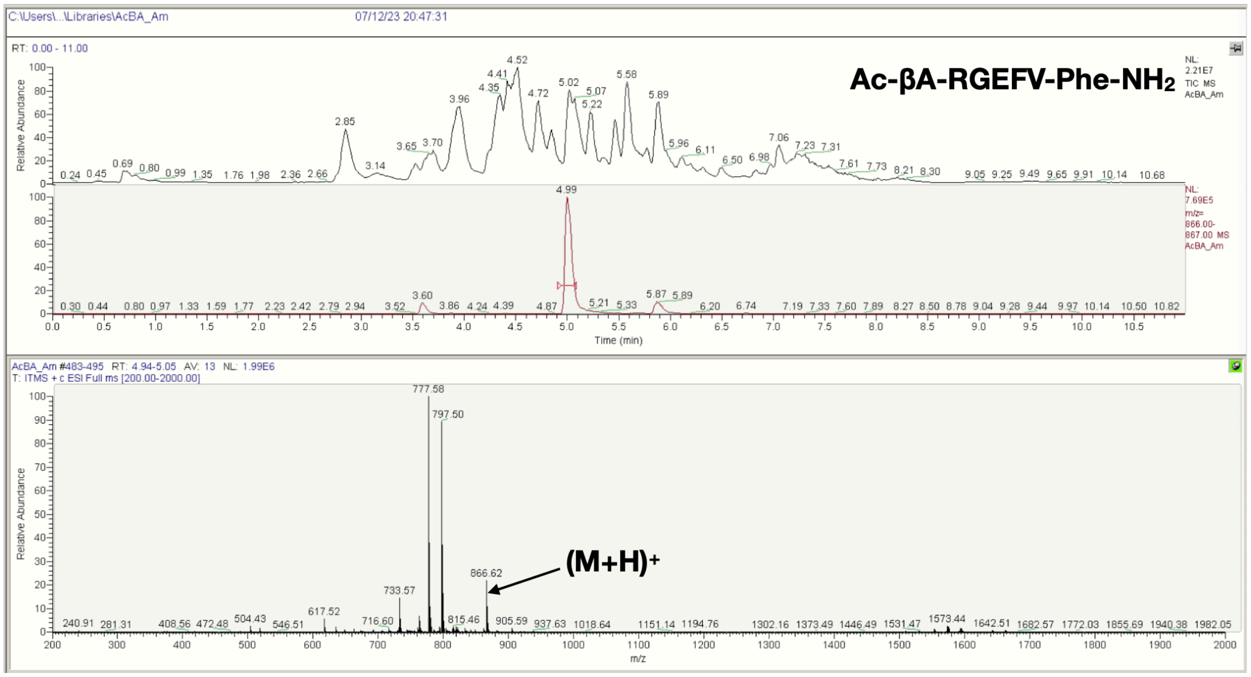

M

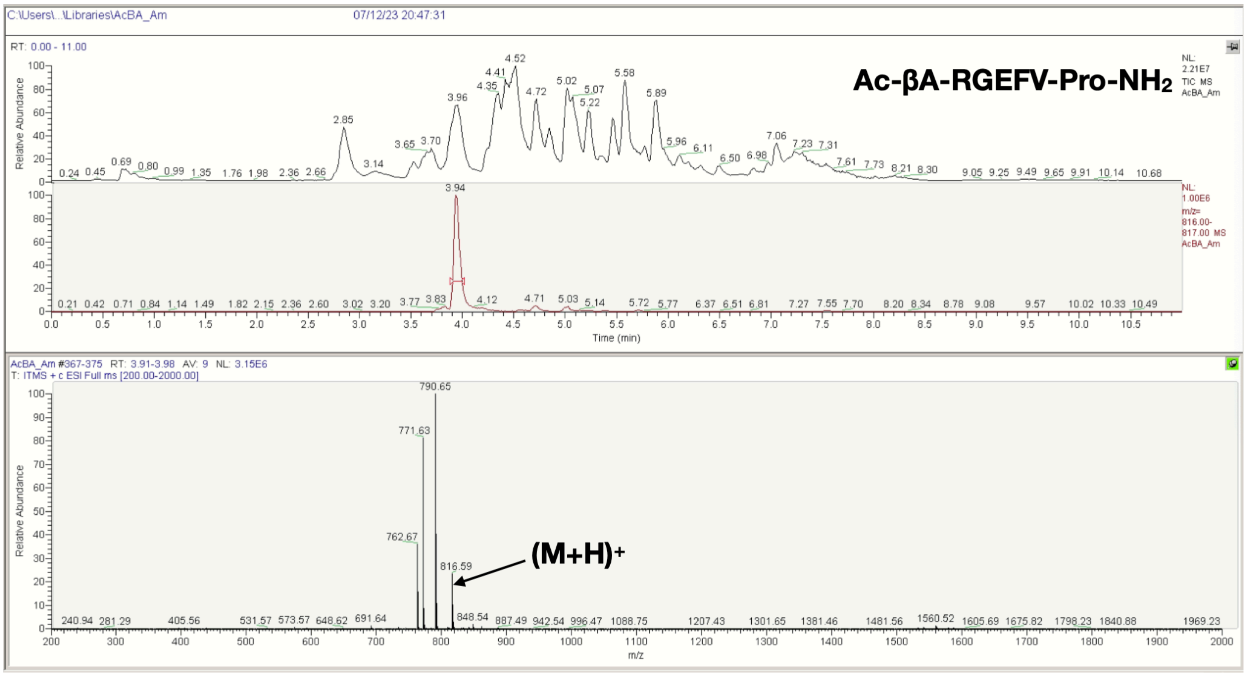
C:\Users\...\Libraries\AcBA_Am
07/12/23 20:47:31
RT: 0.00 - 11.00
Ac-βA-RGEFV-Pro-NH2
NL: 2.21E7 TIC MS AcBA_Am
NL: 1.00E6 m/z= 816.00-817.00 MS AcBA_Am
Relative Abundance
Time (min)
AcBA_Am #367-375 RT: 3.91-3.98 AV: 9 NL: 3.15E6
T: ITMS + c ESI Full ms [200.00-2000.00]
790.65
771.63
762.67
816.59
(M+H)+
m/z

N

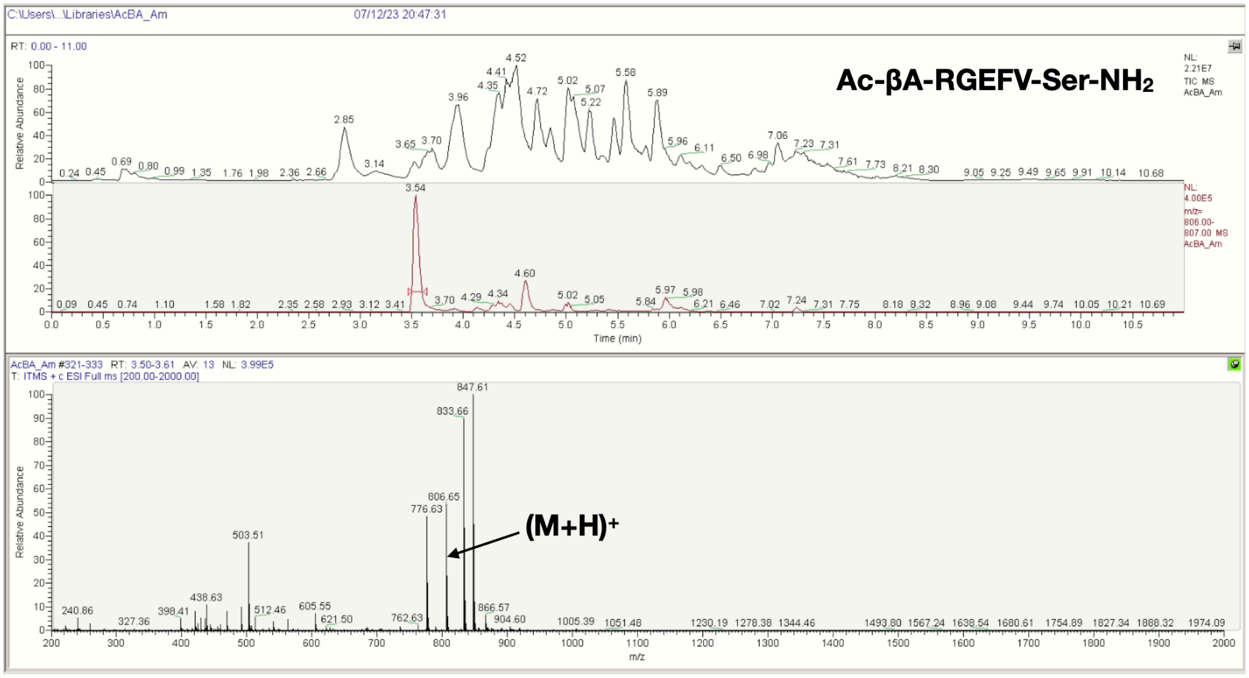
C:\Users\...\Libraries\AcBA_Am
07/12/23 20:47:31
RT: 0.00 - 11.00
Ac-βA-RGEFV-Ser-NH2
NL: 2.21E7 TIC MS AcBA_Am
NL: 4.00E5 m/z= 806.00-807.00 MS AcBA_Am
Relative Abundance
Time (min)
AcBA_Am #321-333 RT: 3.50-3.61 AV: 13 NL: 3.99E5
T: ITMS + c ESI Full ms [200.00-2000.00]
847.61
833.66
806.65
776.63
503.51
(M+H)+
m/z

O

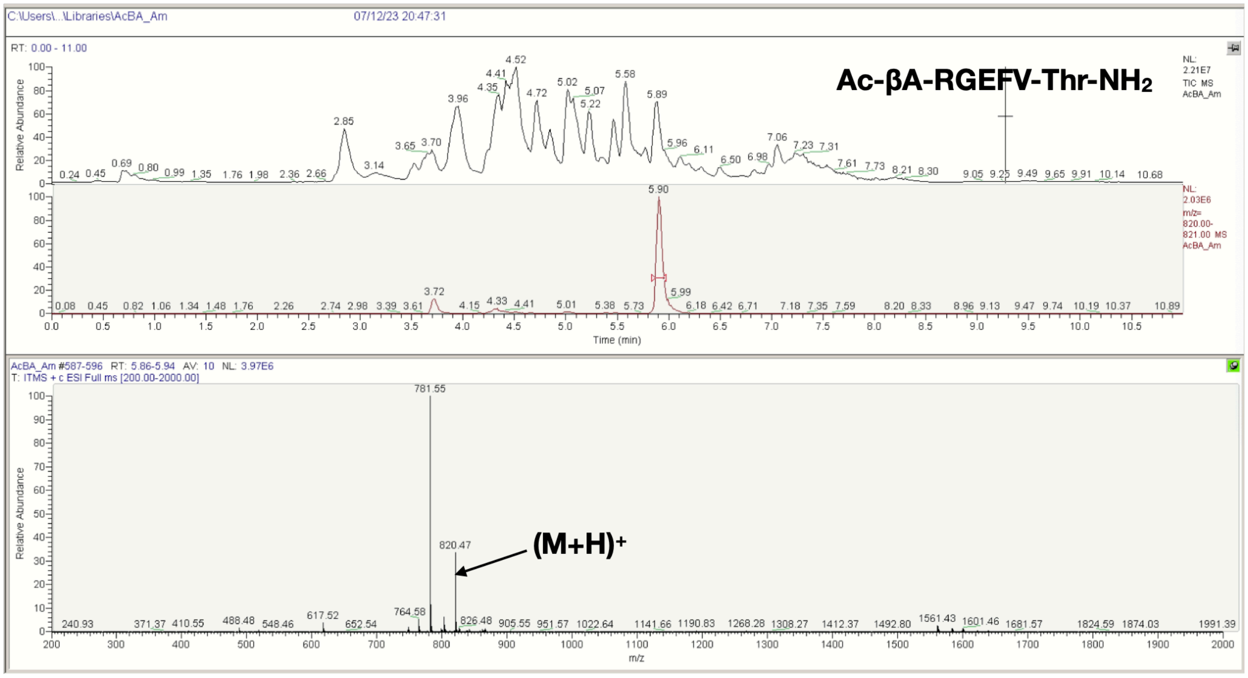

C:\Users\...\Libraries\AcBA_Am
07/12/23 20:47:31
RT: 0.00 - 11.00
Ac-βA-RGEFV-Thr-NH2
Relative Abundance
Time (min)
AcBA_Am #587-596 RT: 5.86-5.94 AV: 10 NL: 3.97E6
T: ITMS + c ESI Full ms [200.00-2000.00]
781.55
820.47
(M+H)+
m/z

P

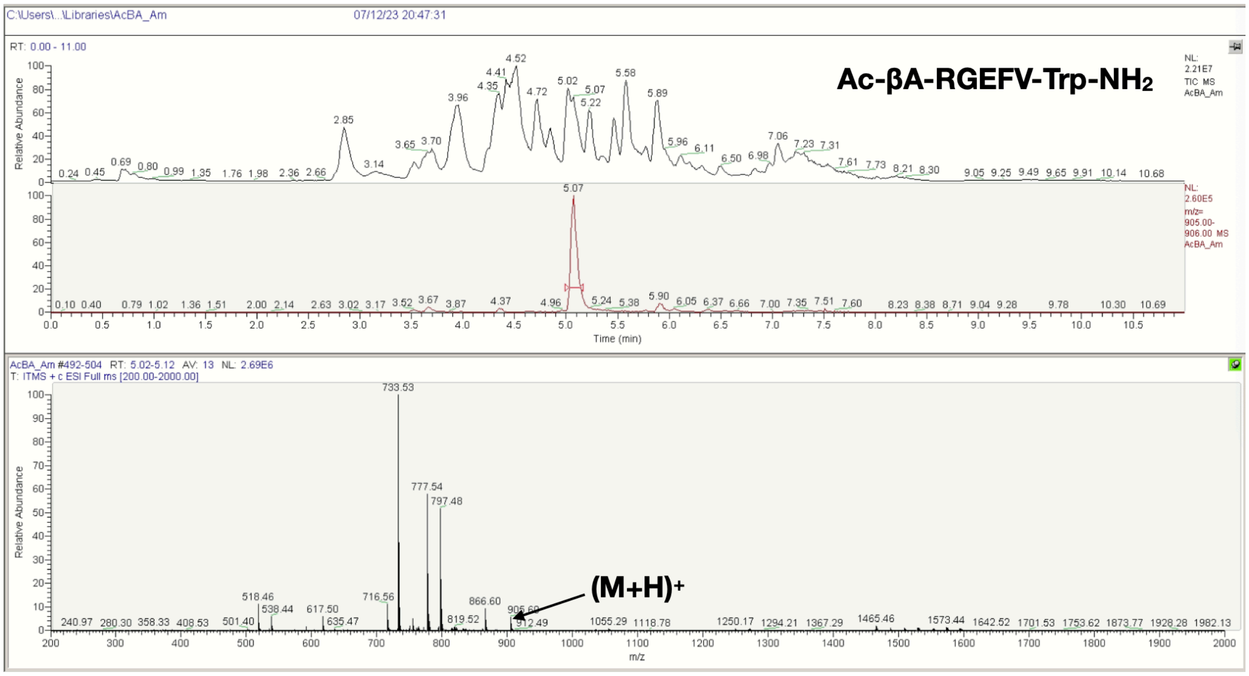

C:\Users\...\Libraries\AcBA_Am
07/12/23 20:47:31
RT: 0.00 - 11.00
Ac-βA-RGEFV-Trp-NH2
Relative Abundance
Time (min)
AcBA_Am #492-504 RT: 5.02-5.12 AV: 13 NL: 2.69E6
T: ITMS + c ESI Full ms [200.00-2000.00]
733.53
777.54
797.48
(M+H)+
m/z

Q

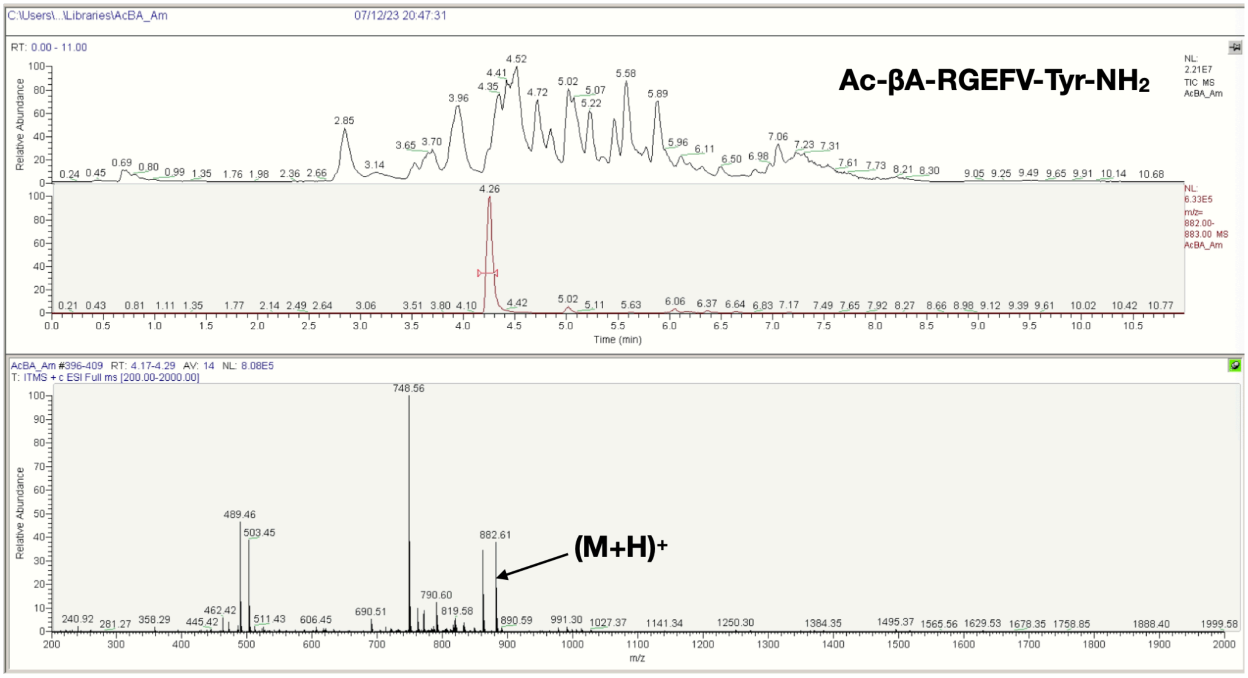

R

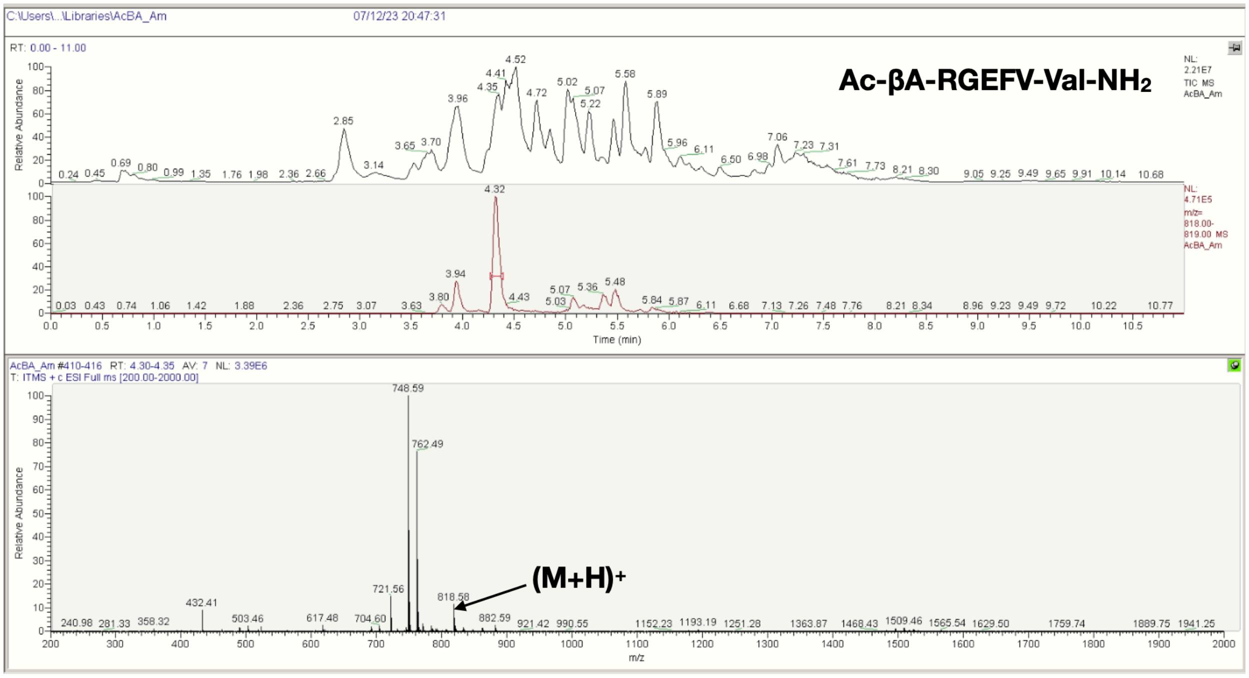

Figure S14. LCMS spectra of the Ac-βA-RGEFV-X-NH_2 libraries, where X = a) Ala, b) Arg, c) Asn, d) Asp, e) Gln, f) Glu, g) Gly, h) His, i) Ile/Leu, j) Lys, k) Met, l) Phe, m) Pro, n) Ser, o) Thr, p) Trp, q) Tyr, r) Val.

A

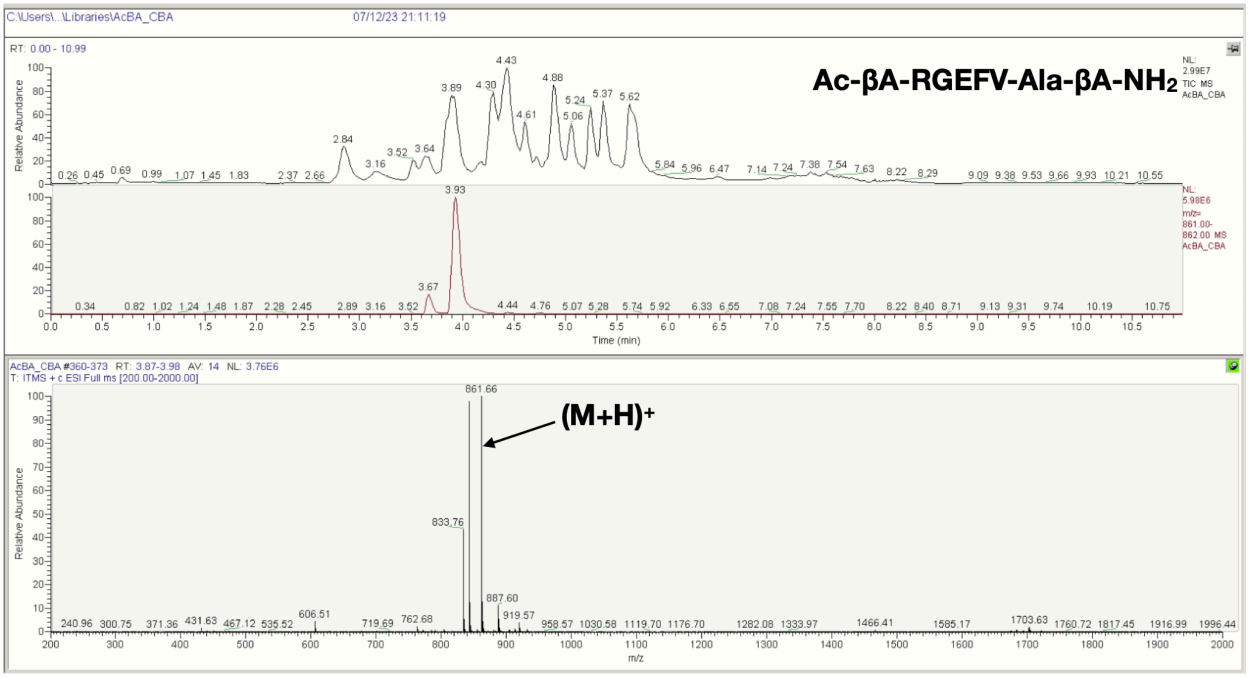
C:\Users\...\Libraries\AcBA_CBA
07/12/23 21:11:19
Ac-βA-RGEFV-Ala-βA-NH2
Relative Abundance
Time (min)
AcBA_CBA #360-373 RT: 3.87-3.98 AV: 14 NL: 3.76E6
T: ITMS + c ESI Full ms [200.00-2000.00]
(M+H)+
861.66
833.76
887.60
m/z

B

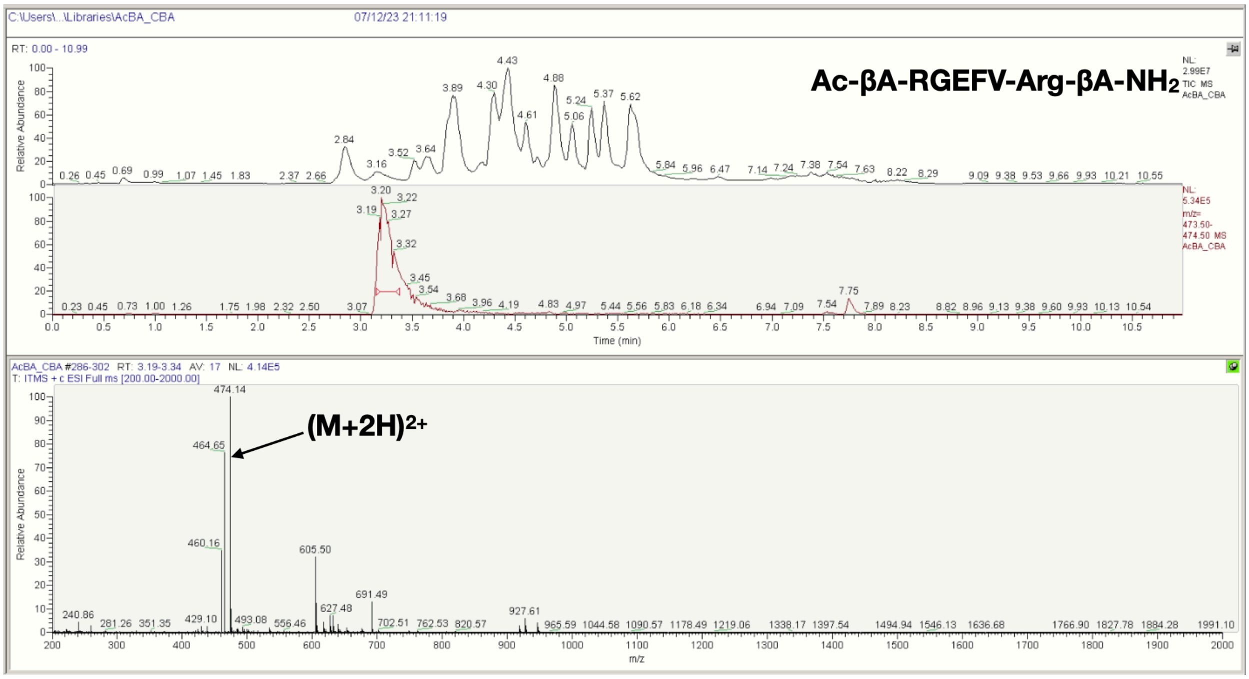
C:\Users\...\Libraries\AcBA_CBA
07/12/23 21:11:19
Ac-βA-RGEFV-Arg-βA-NH2
Relative Abundance
Time (min)
AcBA_CBA #286-302 RT: 3.19-3.34 AV: 17 NL: 4.14E5
T: ITMS + c ESI Full ms [200.00-2000.00]
(M+2H)2+
474.14
464.65
460.16
605.50
691.49
m/z

C

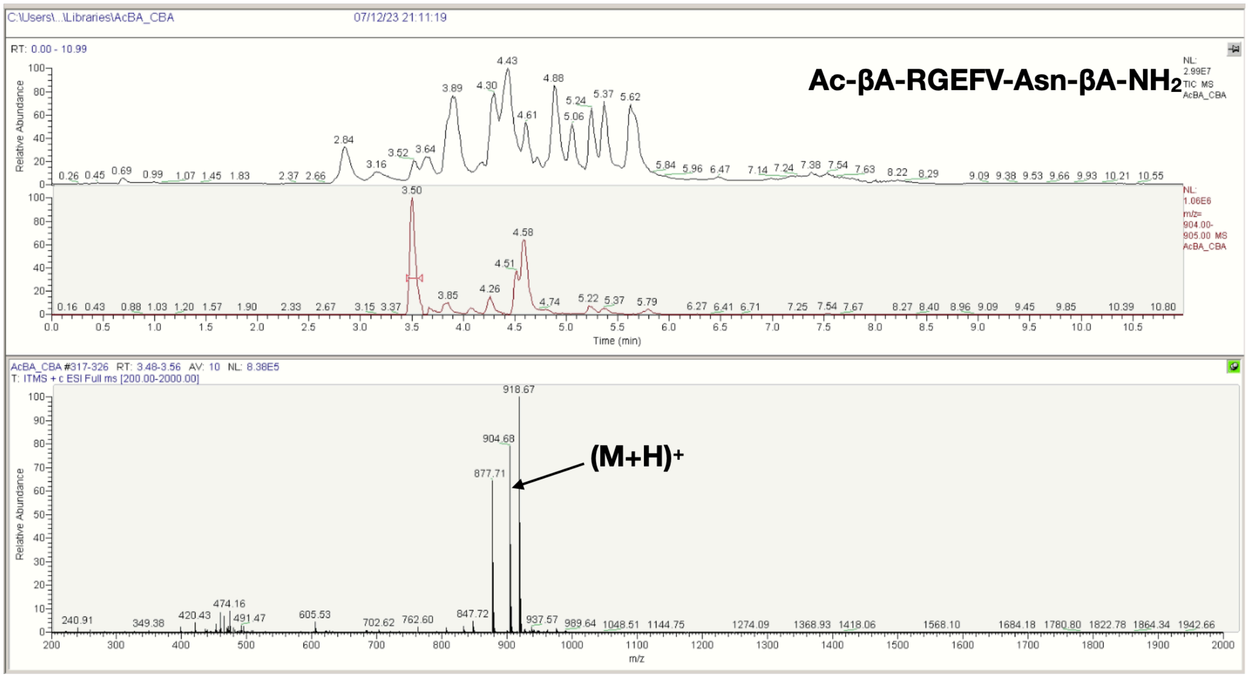
C:\Users\..\Libraries\AcBA_CBA 07/12/23 21:11:19
RT: 0.00 - 10.99
Ac-βA-RGEFV-Asn-βA-NH2
NL: 2.99E7 TIC MS AcBA_CBA
NL: 1.06E6 m/z= 904.00-905.00 MS AcBA_CBA
Relative Abundance
Time (min)
AcBA_CBA #317-326 RT: 3.48-3.56 AV: 10 NL: 8.38E5
T: ITMS + c ESI Full ms [200.00-2000.00]
918.67
904.68
877.71
(M+H)+
m/z

D

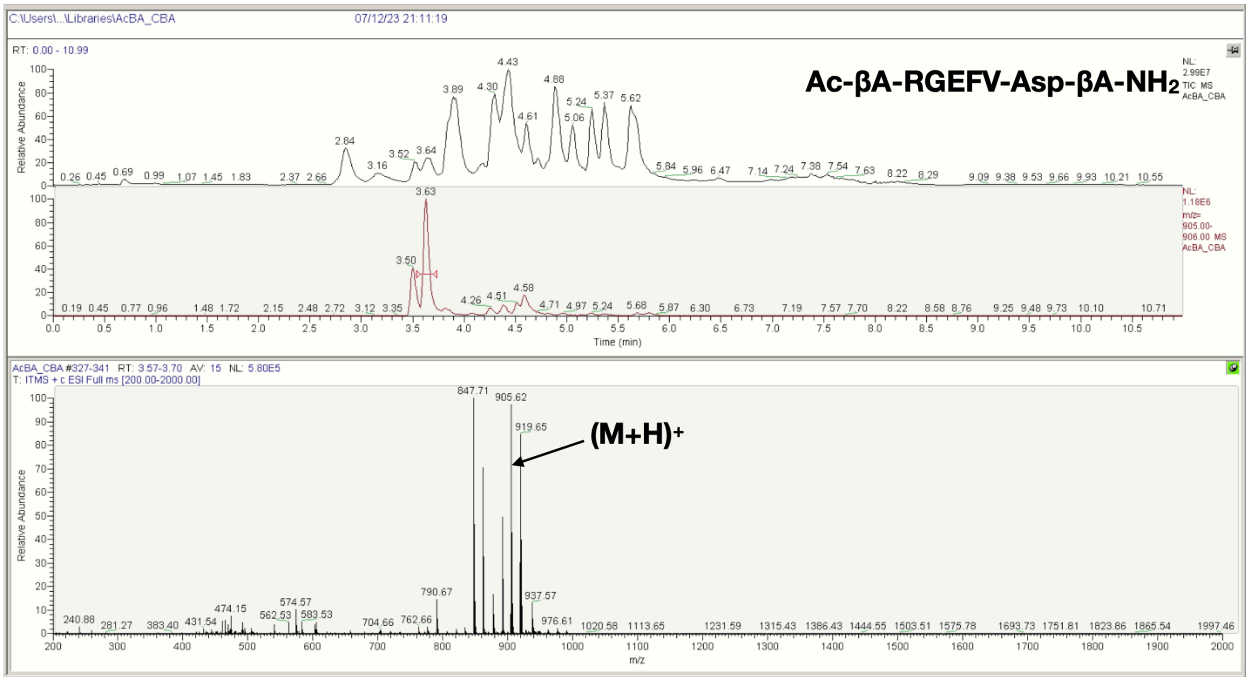
C:\Users\..\Libraries\AcBA_CBA 07/12/23 21:11:19
RT: 0.00 - 10.99
Ac-βA-RGEFV-Asp-βA-NH2
NL: 2.99E7 TIC MS AcBA_CBA
NL: 1.18E6 m/z= 905.00-906.00 MS AcBA_CBA
Relative Abundance
Time (min)
AcBA_CBA #327-341 RT: 3.57-3.70 AV: 15 NL: 5.80E5
T: ITMS + c ESI Full ms [200.00-2000.00]
847.71
905.62
919.65
(M+H)+
m/z

E

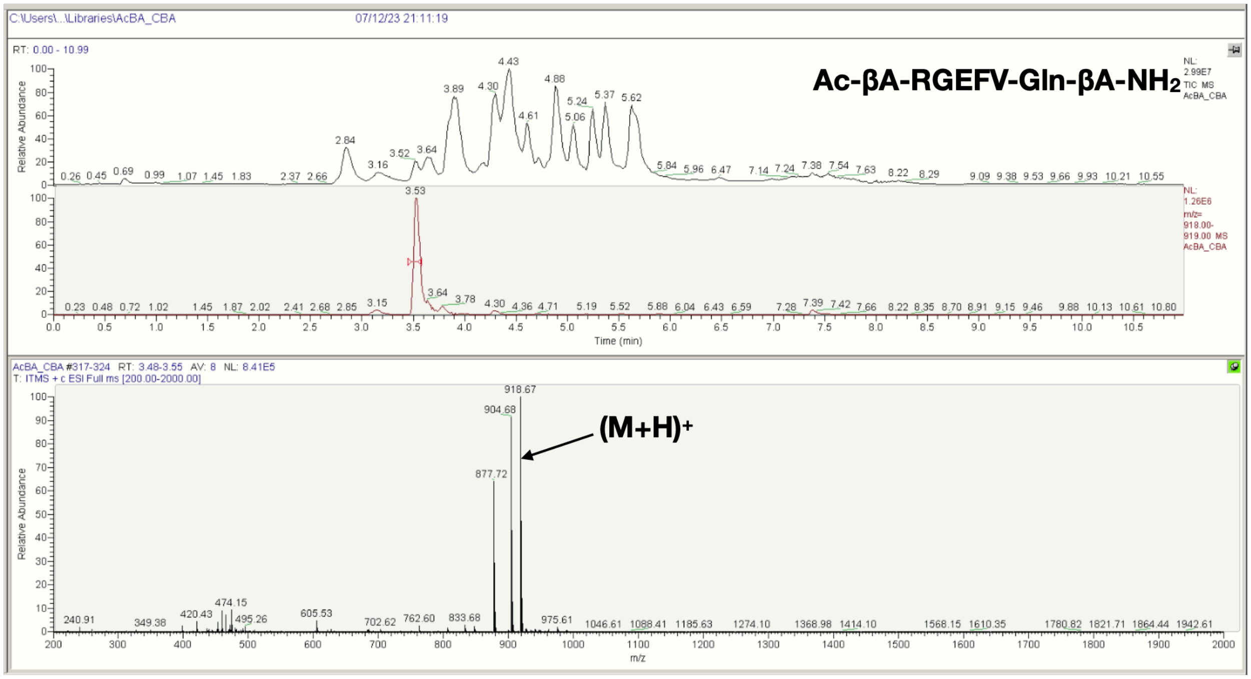

Ac-βA-RGEFV-Gln-βA-NH2
(M+H)+
918.67
904.68
877.72
Relative Abundance
Time (min)
m/z

F

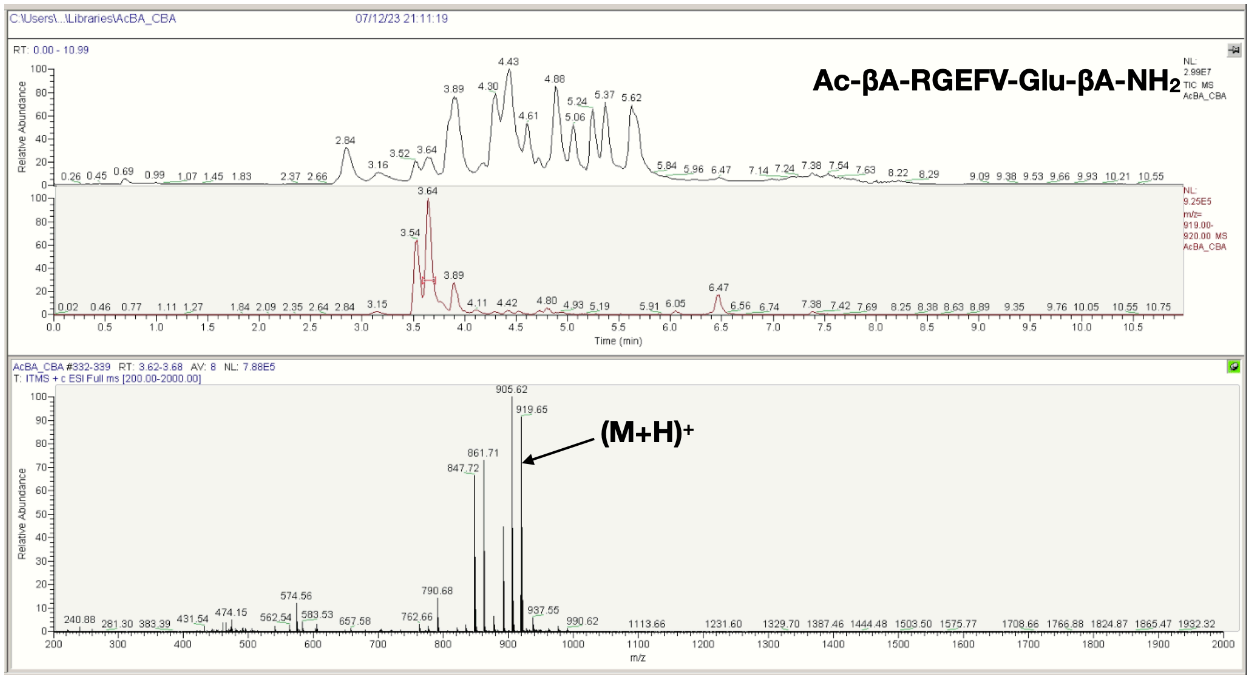

Ac-βA-RGEFV-Glu-βA-NH2
(M+H)+
905.62
919.65
861.71
847.72
Relative Abundance
Time (min)
m/z

G

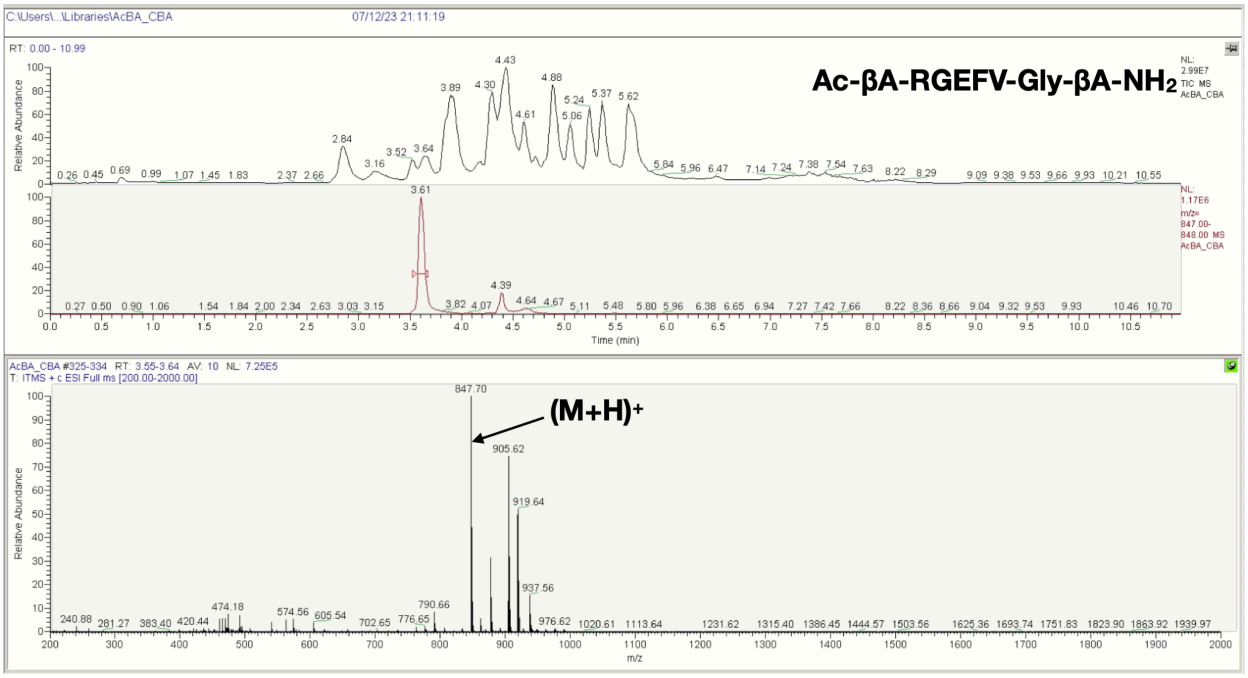
C:\Users\...\Libraries\AcBA_CBA
07/12/23 21:11:19
RT: 0.00 - 10.99
Ac-βA-RGEFV-Gly-βA-NH2
NL: 2.99E7 TIC MS AcBA_CBA
NL: 1.17E6 m/z= 847.00-848.00 MS AcBA_CBA
Relative Abundance
Time (min)
AcBA_CBA #325-334 RT: 3.55-3.64 AV: 10 NL: 7.25E5
T: ITMS + c ESI Full ms [200.00-2000.00]
847.70
(M+H)+
905.62
919.64
937.56
m/z

H

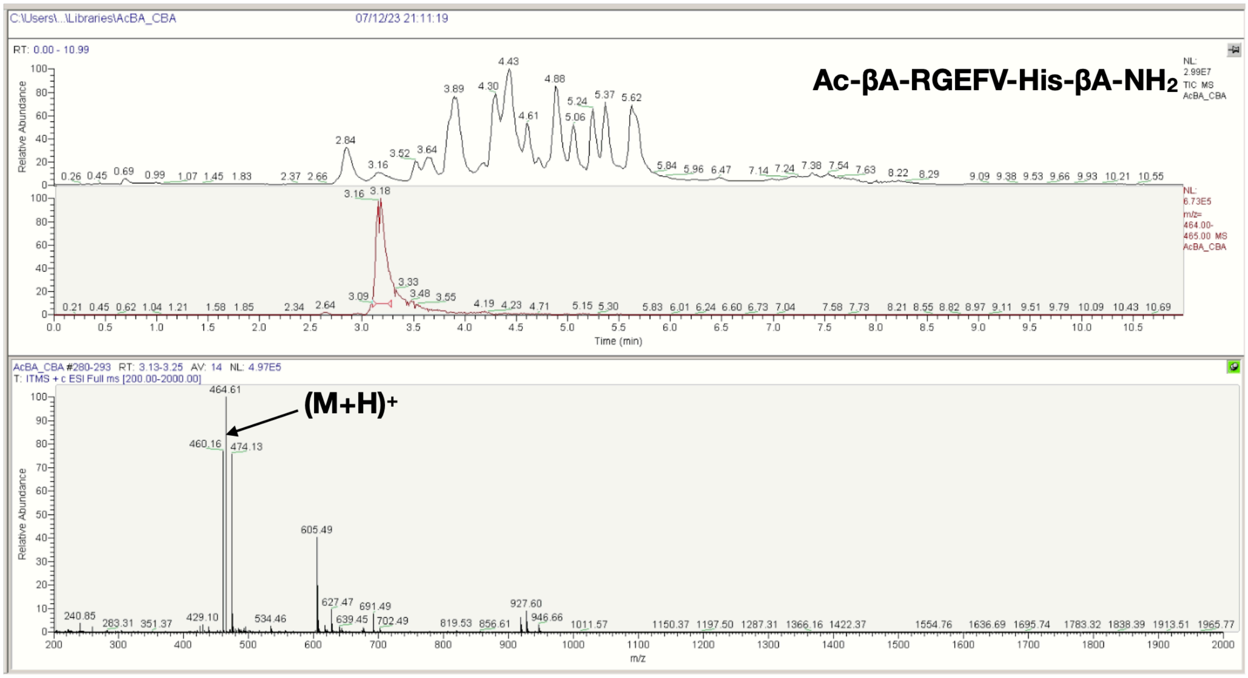
C:\Users\...\Libraries\AcBA_CBA
07/12/23 21:11:19
RT: 0.00 - 10.99
Ac-βA-RGEFV-His-βA-NH2
NL: 2.99E7 TIC MS AcBA_CBA
NL: 6.73E5 m/z= 464.00-465.00 MS AcBA_CBA
Relative Abundance
Time (min)
AcBA_CBA #280-293 RT: 3.13-3.25 AV: 14 NL: 4.97E5
T: ITMS + c ESI Full ms [200.00-2000.00]
464.61
(M+H)+
460.16
474.13
605.49
m/z

I

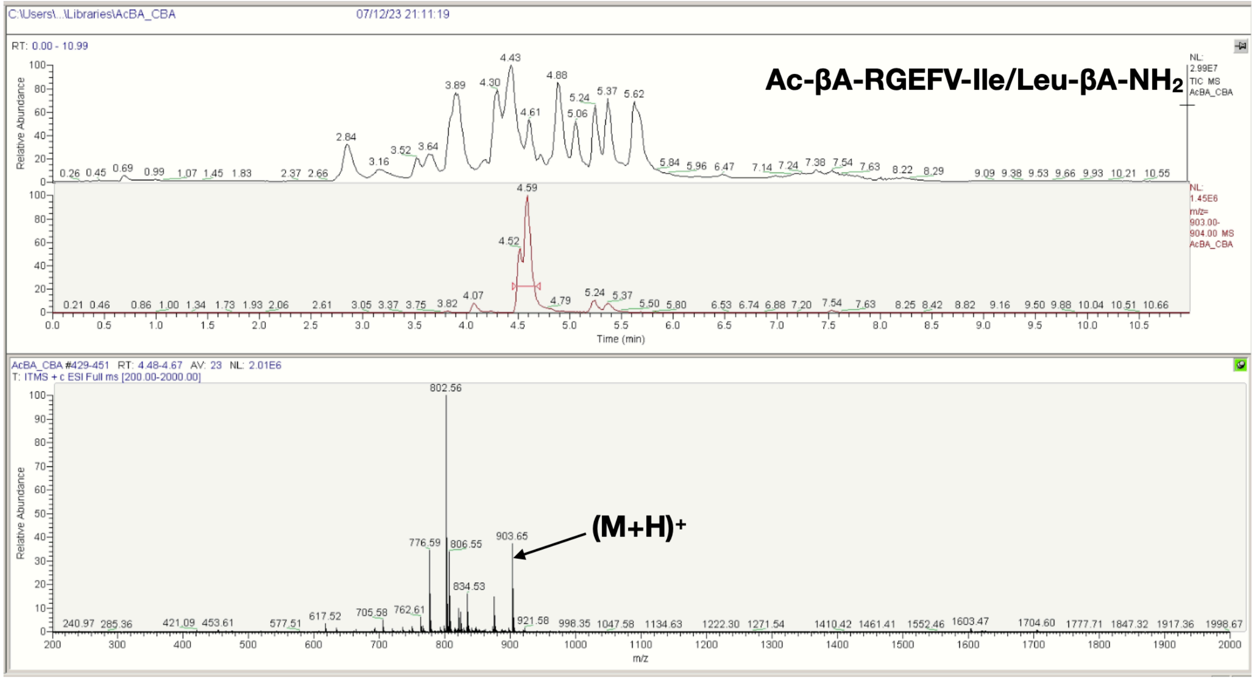
C:\Users\...\Libraries\AcBA_CBA
07/12/23 21:11:19
RT: 0.00 - 10.99
Ac-βA-RGEFV-Ile/Leu-βA-NH2
NL: 2.99E7 TIC MS AcBA_CBA
NL: 1.45E6 m/z= 903.00-904.00 MS AcBA_CBA
Time (min)
Relative Abundance
AcBA_CBA #429-451 RT: 4.48-4.67 AV: 23 NL: 2.01E6
T: ITMS + c ESI Full ms [200.00-2000.00]
802.56
903.65
(M+H)+
m/z

J

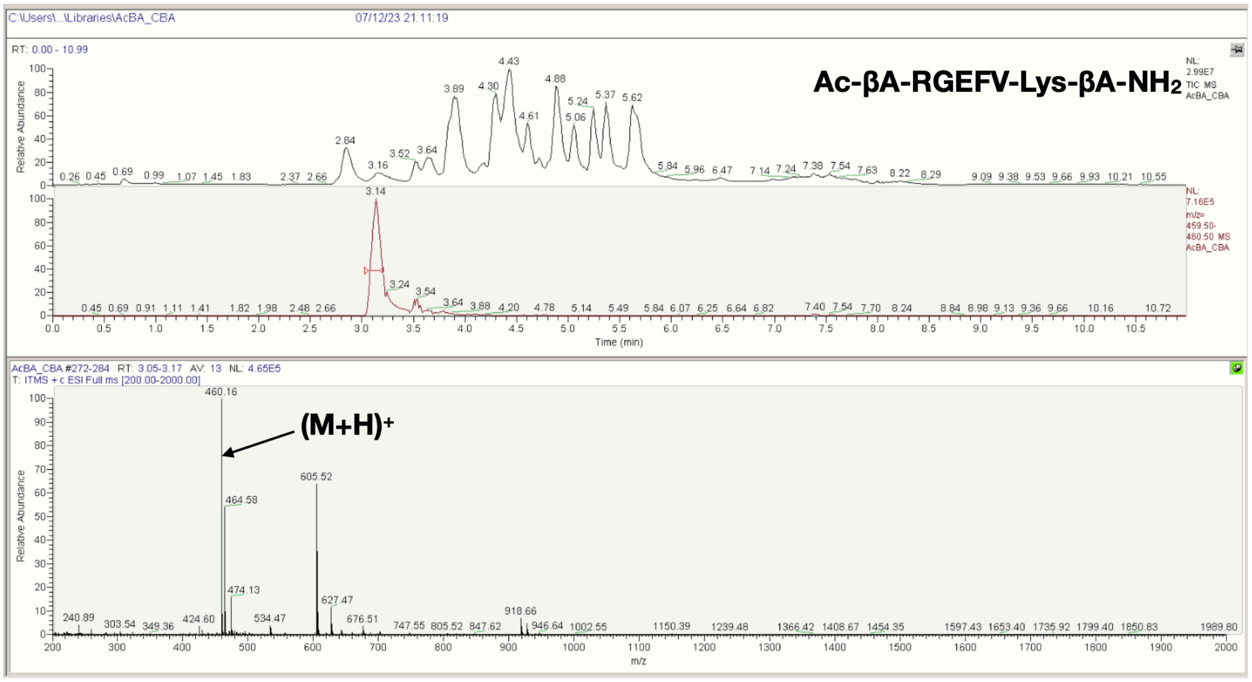
C:\Users\...\Libraries\AcBA_CBA
07/12/23 21:11:19
RT: 0.00 - 10.99
Ac-βA-RGEFV-Lys-βA-NH2
NL: 2.99E7 TIC MS AcBA_CBA
NL: 7.16E5 m/z= 459.50-460.50 MS AcBA_CBA
Time (min)
Relative Abundance
AcBA_CBA #272-284 RT: 3.05-3.17 AV: 13 NL: 4.65E5
T: ITMS + c ESI Full ms [200.00-2000.00]
460.16
(M+H)+
605.52
m/z

K

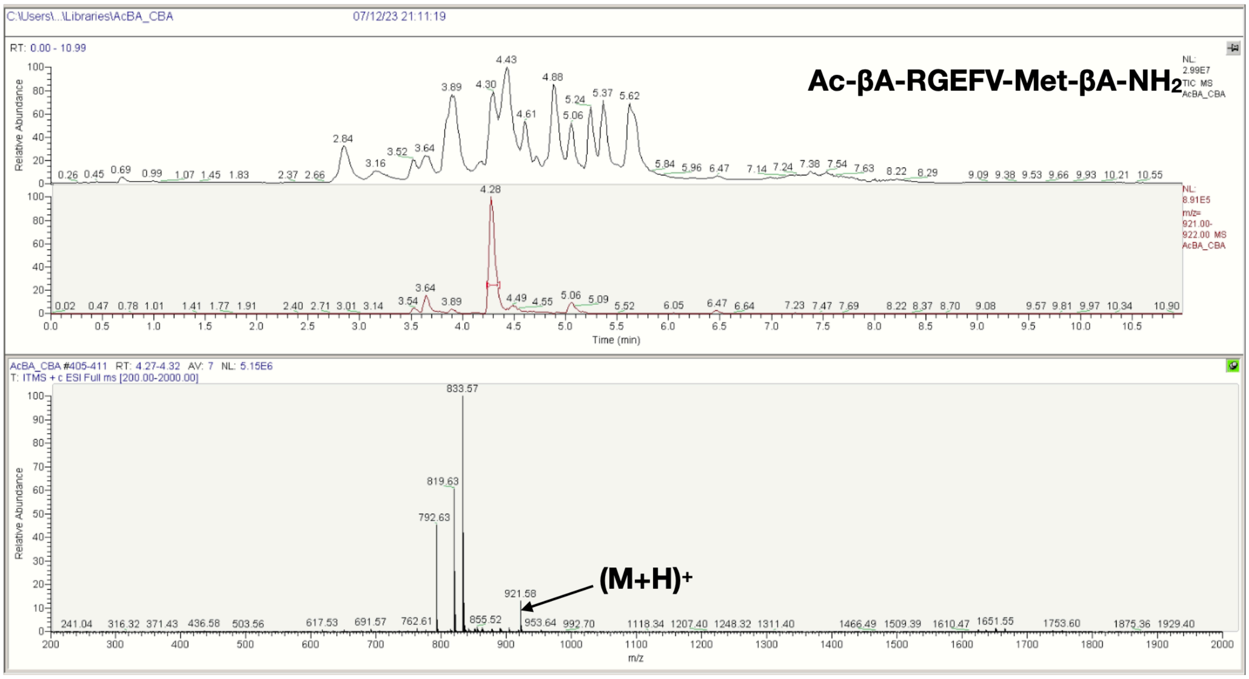
C:\Users\...\Libraries\AcBA_CBA
07/12/23 21:11:19
Ac-βA-RGEFV-Met-βA-NH2
Relative Abundance
Time (min)
AcBA_CBA #405-411 RT: 4.27-4.32 AV: 7 NL: 5.15E6
T: ITMS + c ESI Full ms [200.00-2000.00]
833.57
819.63
792.63
921.58
(M+H)+
m/z

L

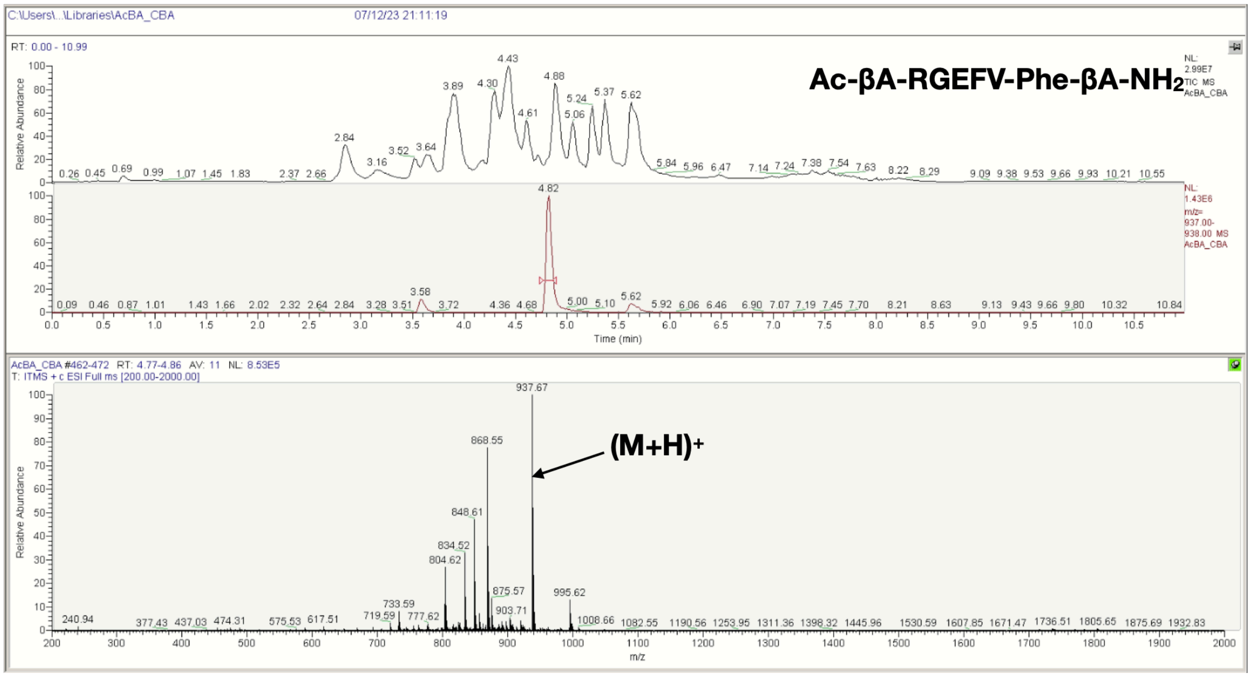
C:\Users\...\Libraries\AcBA_CBA
07/12/23 21:11:19
Ac-βA-RGEFV-Phe-βA-NH2
Relative Abundance
Time (min)
AcBA_CBA #462-472 RT: 4.77-4.86 AV: 11 NL: 8.53E5
T: ITMS + c ESI Full ms [200.00-2000.00]
937.67
868.55
848.61
834.52
804.62
(M+H)+
m/z

M

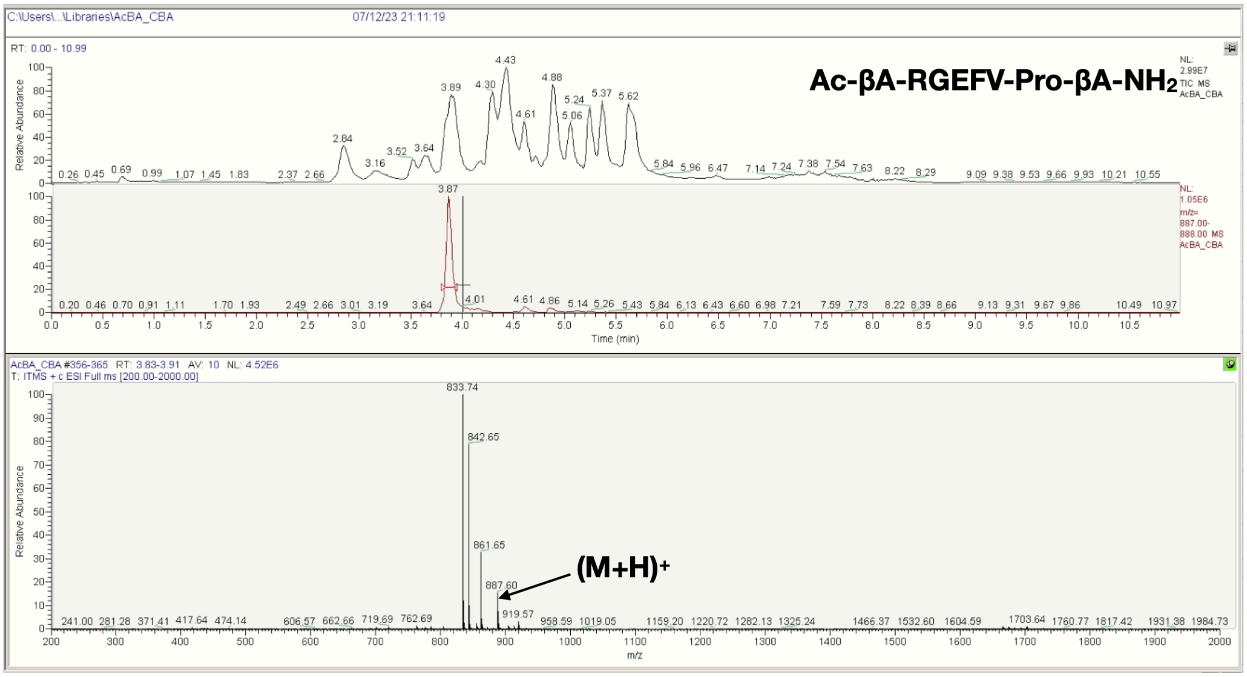

N

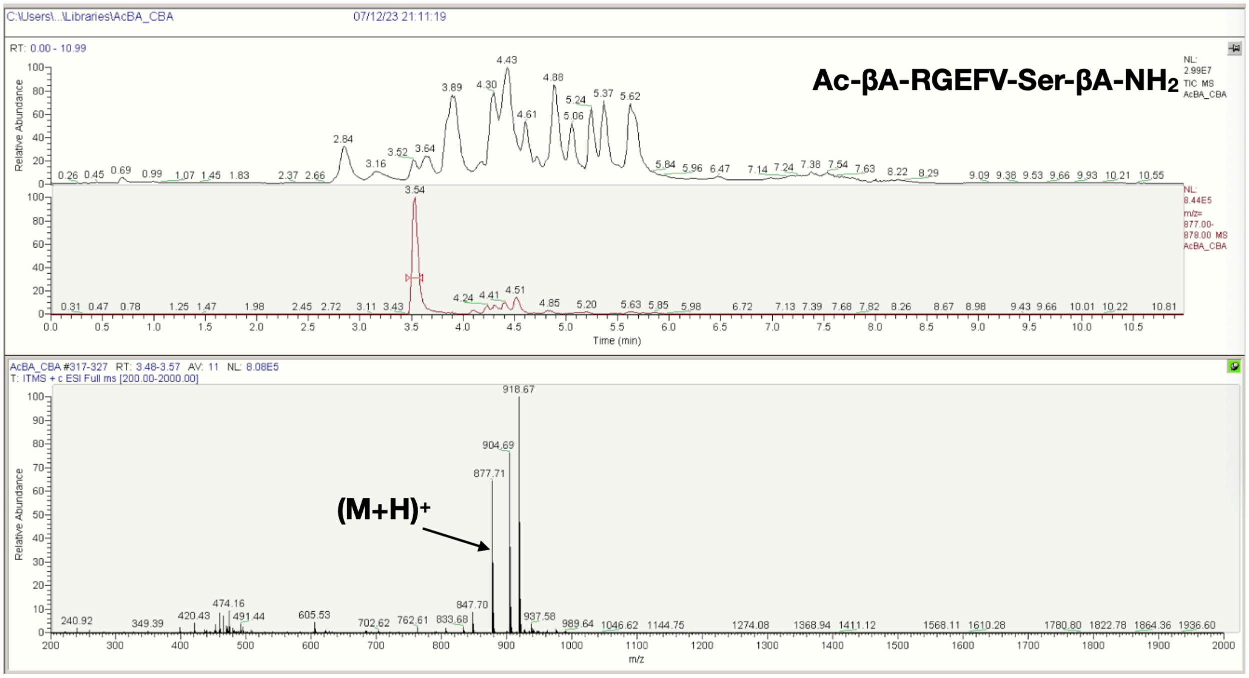

O

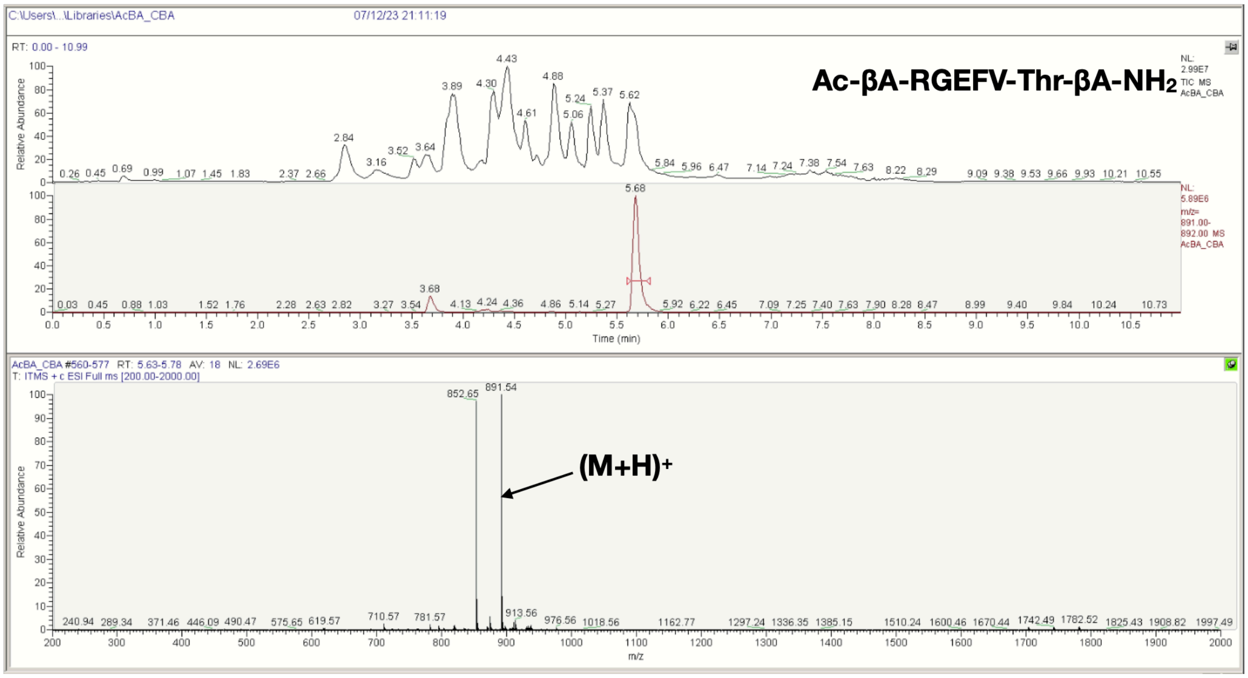
C:\Users\...\Libraries\AcBA_CBA
07/12/23 21:11:19
Ac-βA-RGEFV-Thr-βA-NH2
Relative Abundance
Time (min)
AcBA_CBA #560-577 RT: 5.63-5.78 AV: 18 NL: 2.69E6
T: ITMS + c ESI Full ms [200.00-2000.00]
852.65
891.54
(M+H)+
m/z

P

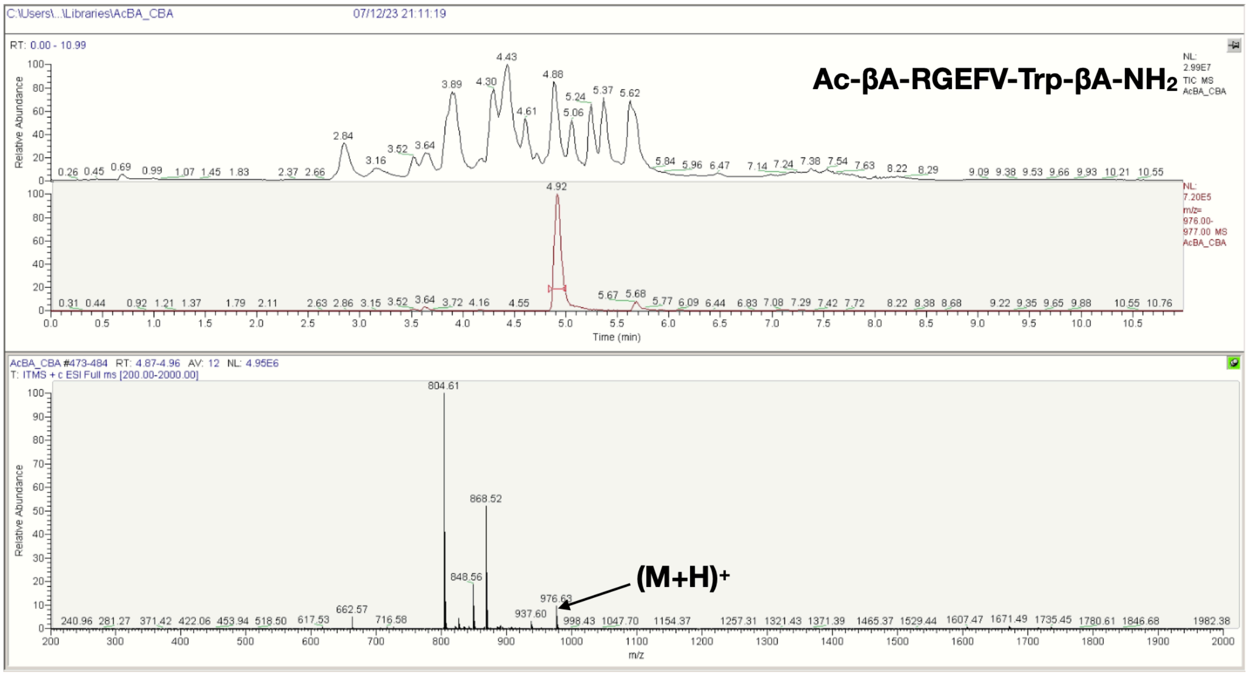
C:\Users\...\Libraries\AcBA_CBA
07/12/23 21:11:19
Ac-βA-RGEFV-Trp-βA-NH2
Relative Abundance
Time (min)
AcBA_CBA #473-484 RT: 4.87-4.96 AV: 12 NL: 4.95E6
T: ITMS + c ESI Full ms [200.00-2000.00]
804.61
868.52
848.56
976.63
(M+H)+
m/z

Q

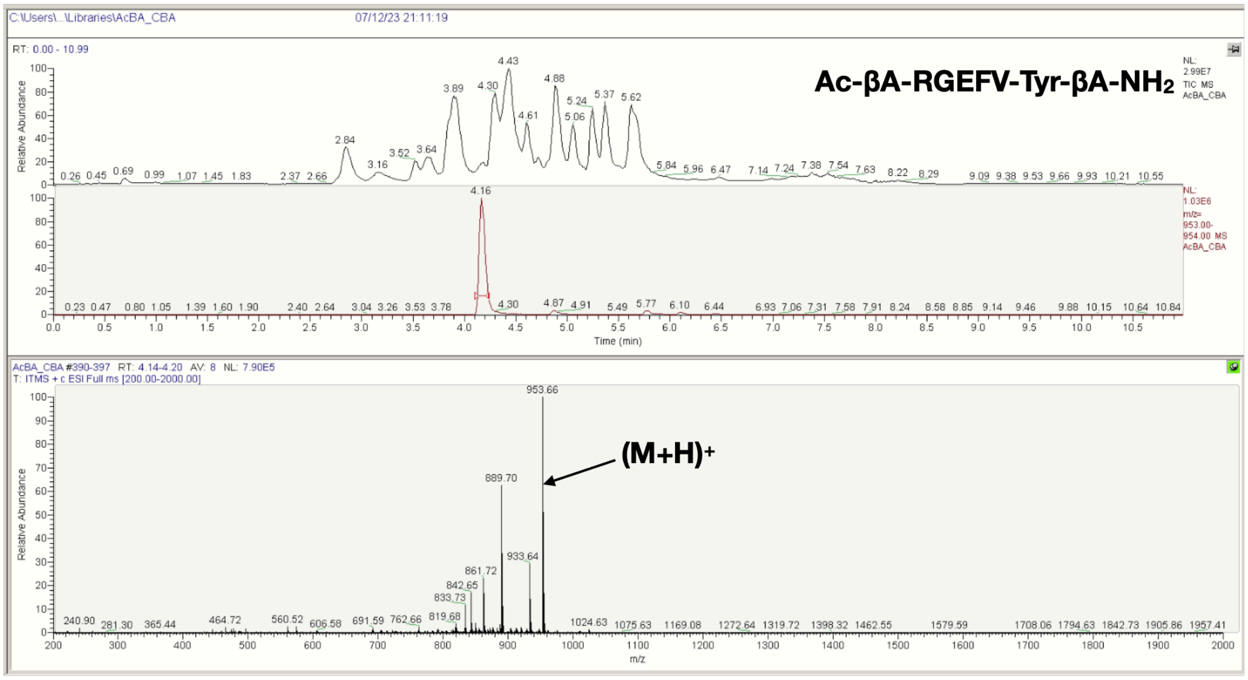

R

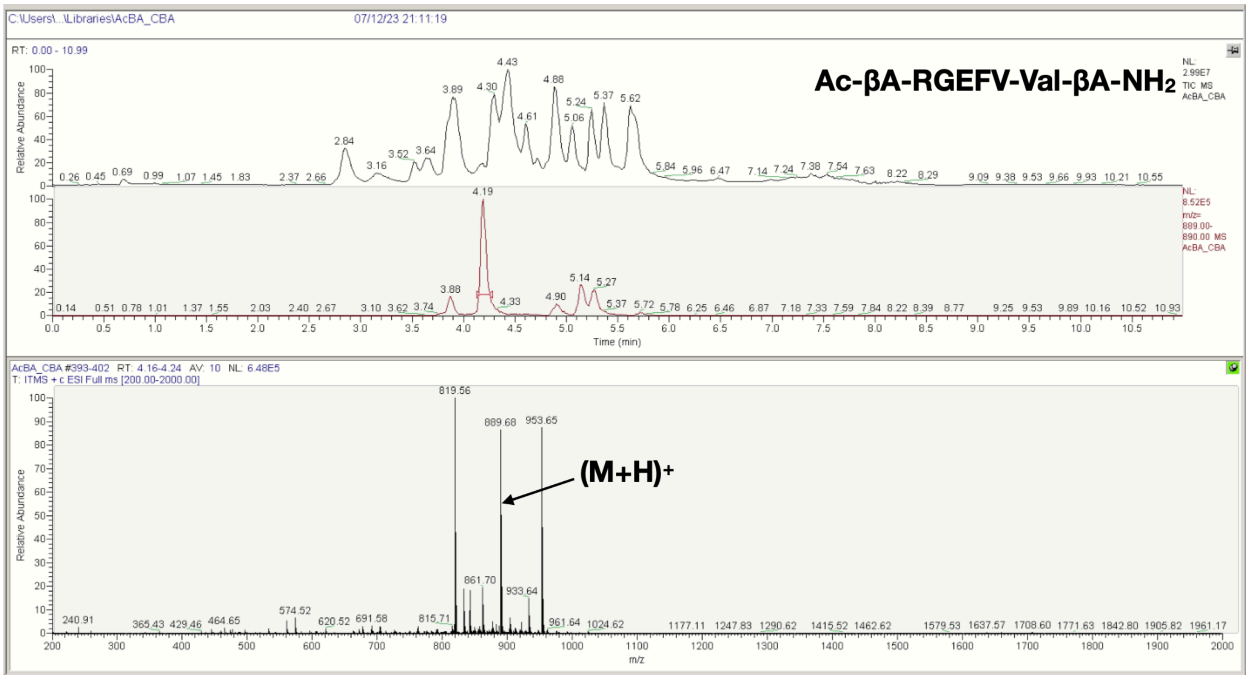

Figure S15. LCMS spectra of the Ac-βA-RGEFV-X-βA-NH_2 libraries, where X = a) Ala, b) Arg, c) Asn, d) Asp, e) Gln, f) Glu, g) Gly, h) His, i) Ile/Leu, j) Lys, k) Met, l) Phe, m) Pro, n) Ser, o) Thr, p) Trp, q) Tyr, r) Val.

A

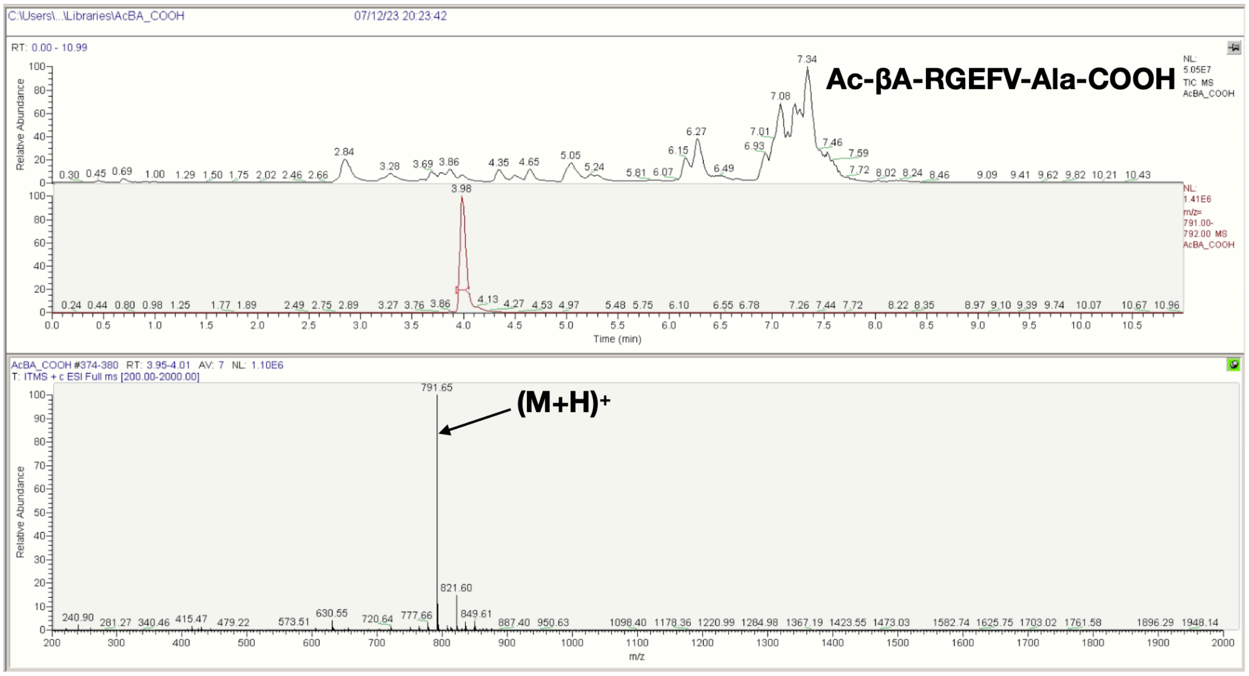
C:\Users\...\Libraries\AcBA_COOH
07/12/23 20:23:42
Ac-βA-RGEFV-Ala-COOH
AcBA_COOH #374-380 RT: 3.95-4.01 AV: 7 NL: 1.10E6
T: ITMS + c ESI Full ms [200.00-2000.00]
791.65
(M+H)+
Relative Abundance
Time (min)
m/z

B

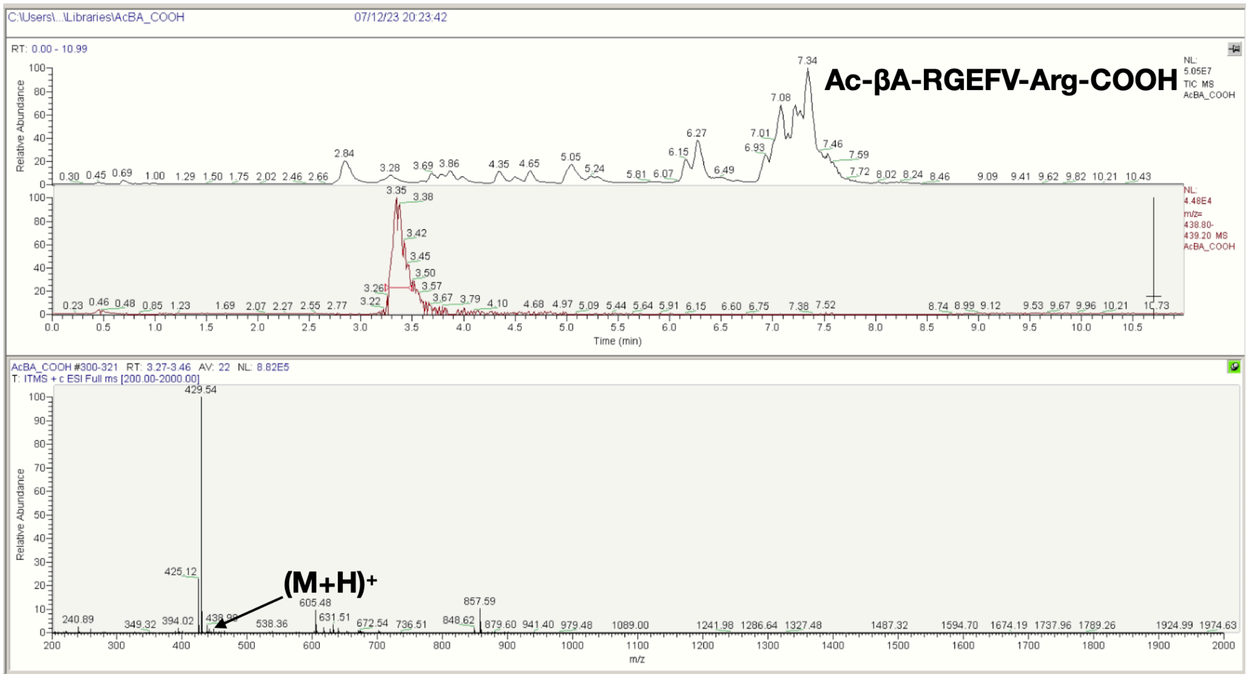
C:\Users\...\Libraries\AcBA_COOH
07/12/23 20:23:42
Ac-βA-RGEFV-Arg-COOH
AcBA_COOH #300-321 RT: 3.27-3.46 AV: 22 NL: 8.82E5
T: ITMS + c ESI Full ms [200.00-2000.00]
429.54
(M+H)+
Relative Abundance
Time (min)
m/z

C

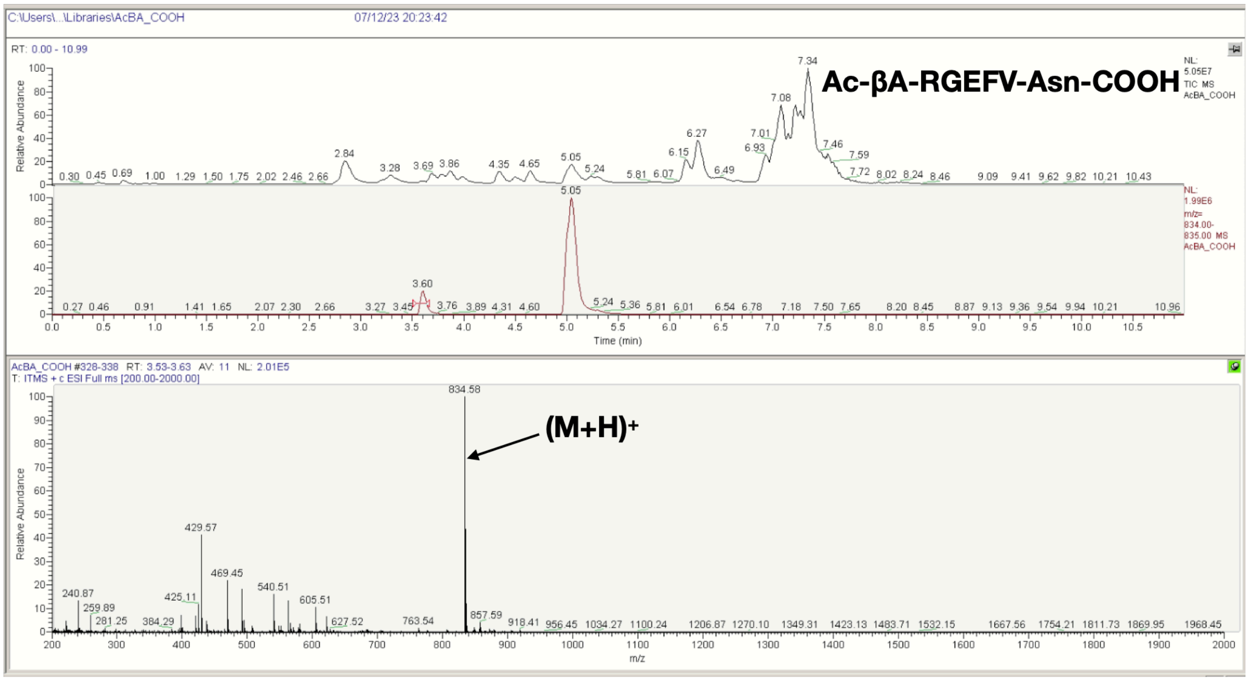

C:\Users\...\Libraries\AcBA_COOH
07/12/23 20:23:42
RT: 0.00 - 10.99
Ac-βA-RGEFV-Asn-COOH
NL: 5.05E7 TIC MS AcBA_COOH
NL: 1.99E6 m/z= 834.00-835.00 MS AcBA_COOH
Relative Abundance
Time (min)
AcBA_COOH #328-338 RT: 3.53-3.63 AV: 11 NL: 2.01E5
T: ITMS + c ESI Full ms [200.00-2000.00]
834.58
(M+H)+
m/z

D

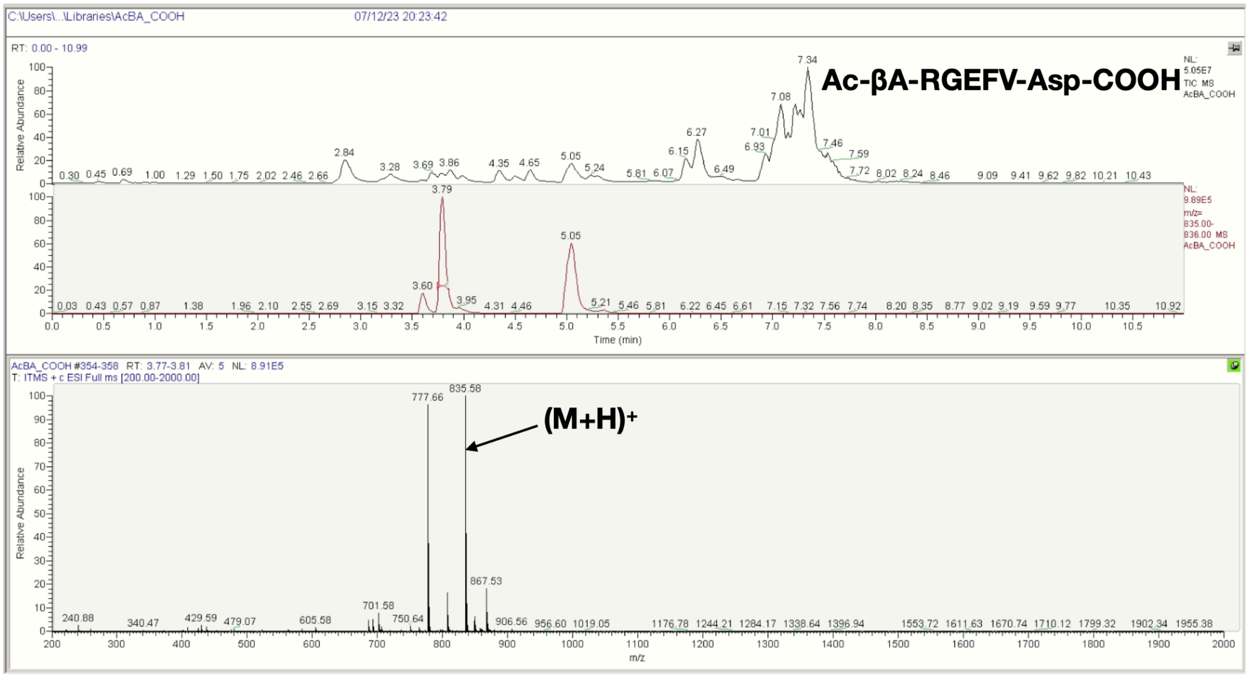

C:\Users\...\Libraries\AcBA_COOH
07/12/23 20:23:42
RT: 0.00 - 10.99
Ac-βA-RGEFV-Asp-COOH
NL: 5.05E7 TIC MS AcBA_COOH
NL: 9.89E5 m/z= 835.00-836.00 MS AcBA_COOH
Relative Abundance
Time (min)
AcBA_COOH #354-358 RT: 3.77-3.81 AV: 5 NL: 8.91E5
T: ITMS + c ESI Full ms [200.00-2000.00]
777.66
835.58
(M+H)+
m/z

E

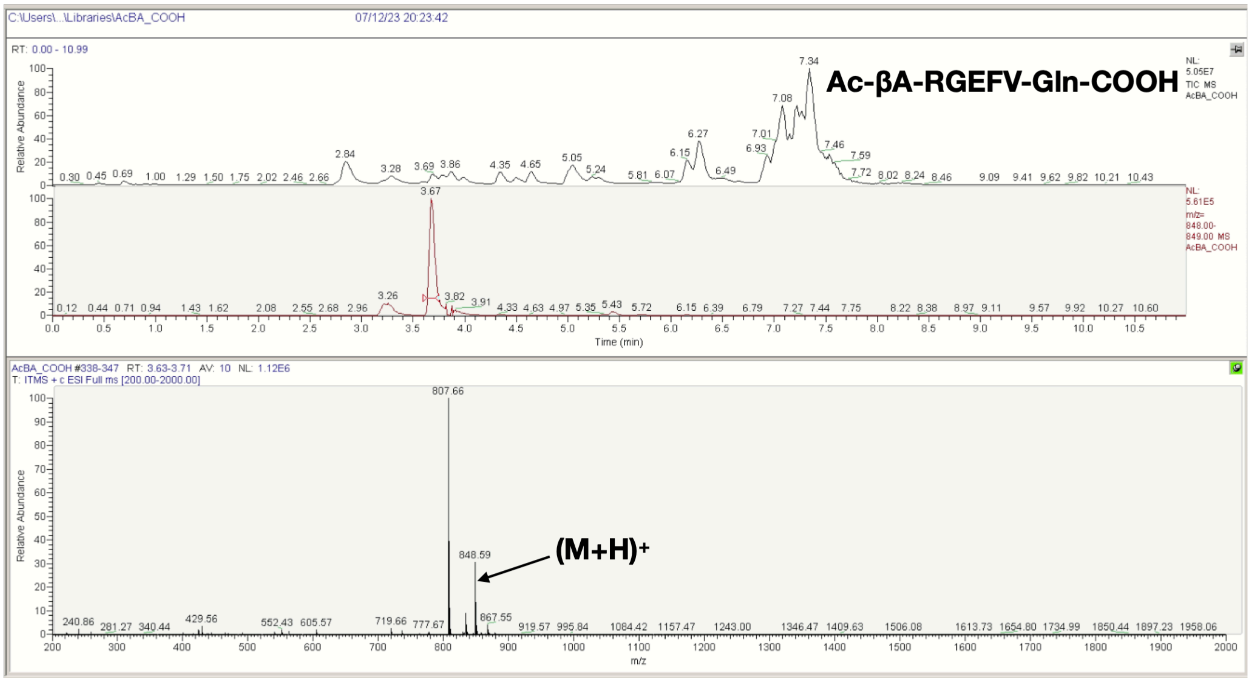

F

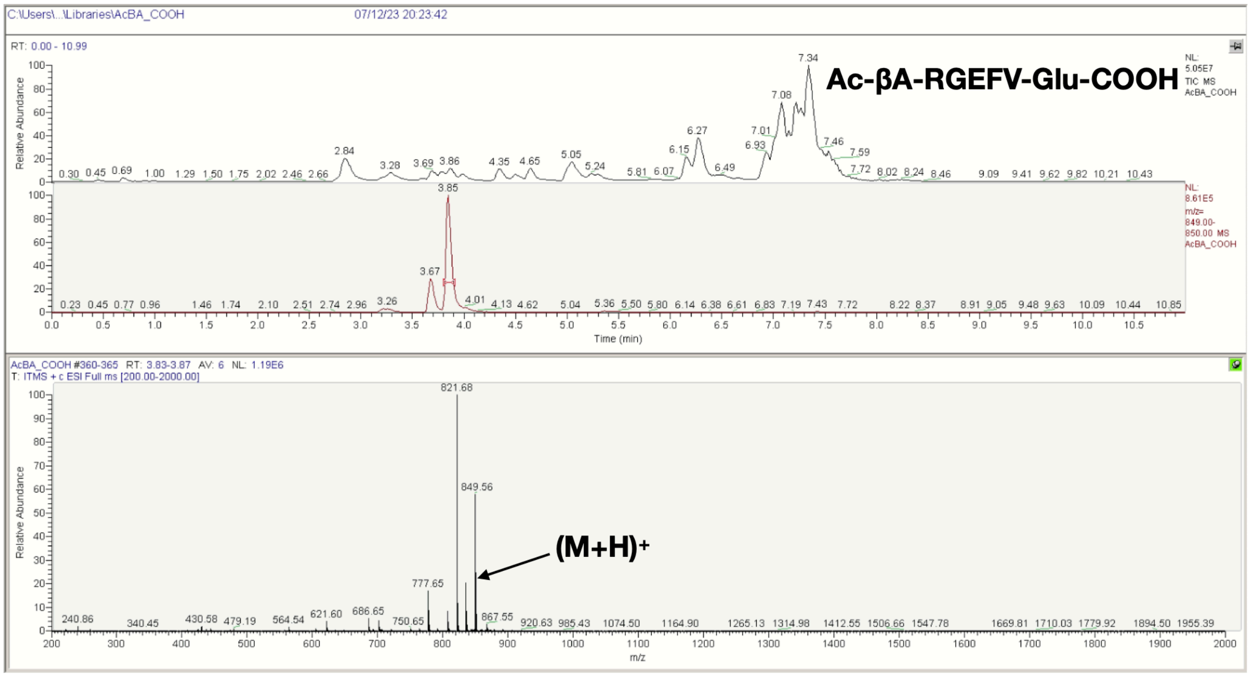

G

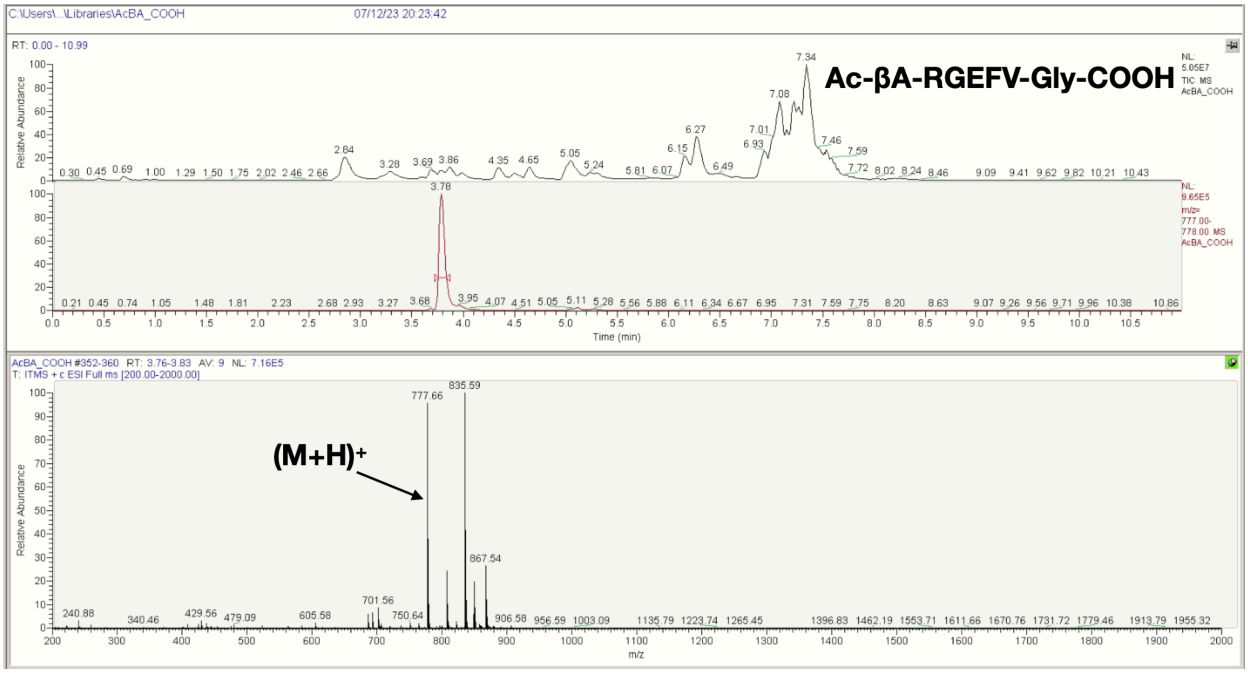
Ac-βA-RGEFV-Gly-COOH
(M+H)+
777.66
835.59
867.54

H

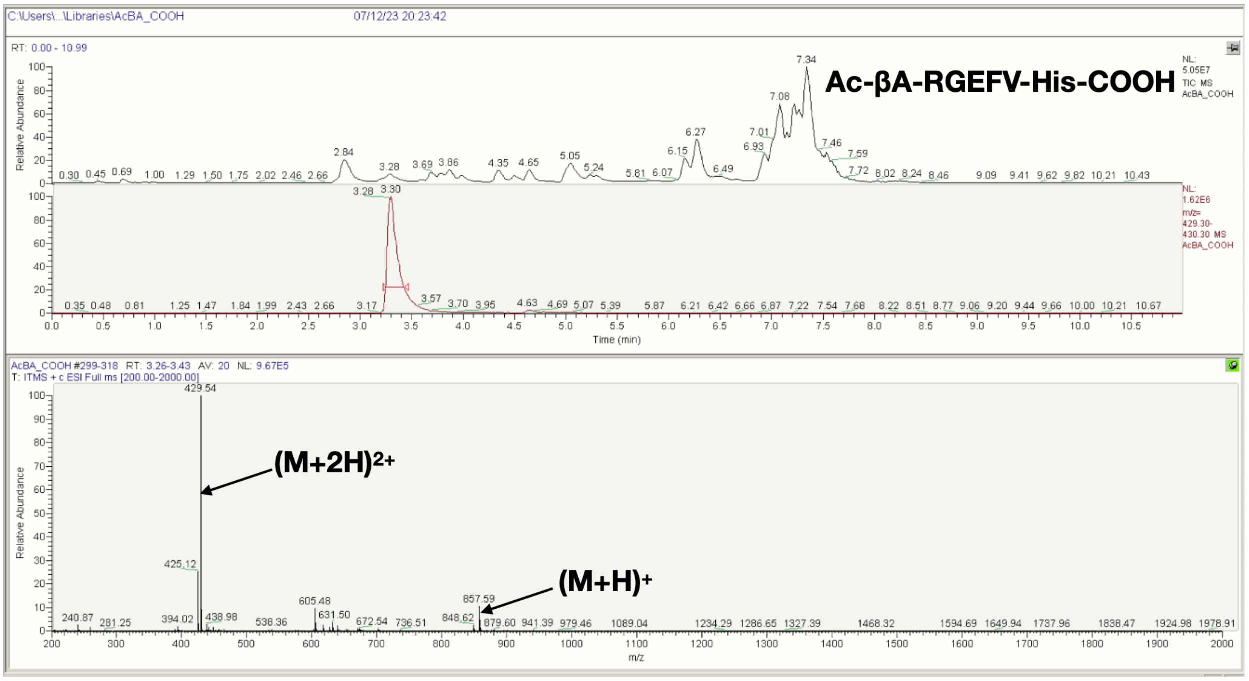
Ac-βA-RGEFV-His-COOH
(M+2H)2+
(M+H)+
429.54
857.59

I

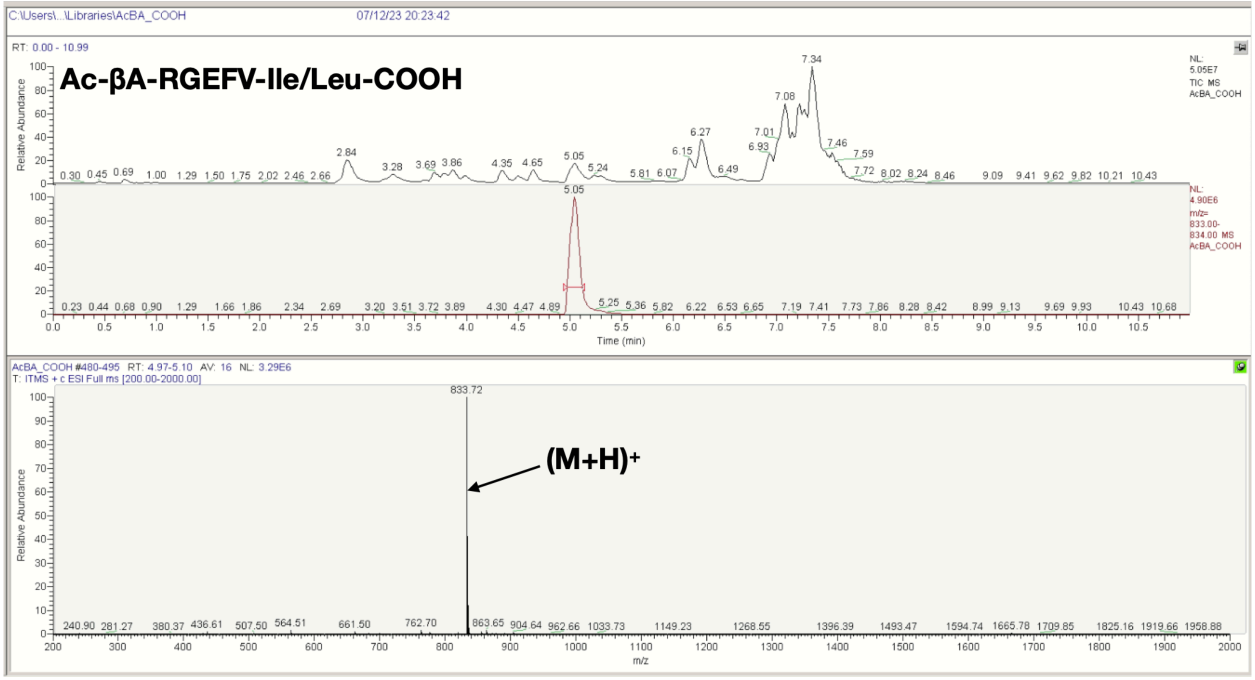
C:\Users\...\Libraries\AcBA_COOH
07/12/23 20:23:42
RT: 0.00 - 10.99
Ac-βA-RGEFV-Ile/Leu-COOH
NL: 5.05E7 TIC MS AcBA_COOH
NL: 4.90E6 m/z= 833.00-834.00 MS AcBA_COOH
Relative Abundance
Time (min)
AcBA_COOH #480-495 RT: 4.97-5.10 AV: 16 NL: 3.29E6
T: ITMS + c ESI Full ms [200.00-2000.00]
833.72
(M+H)+
m/z

J

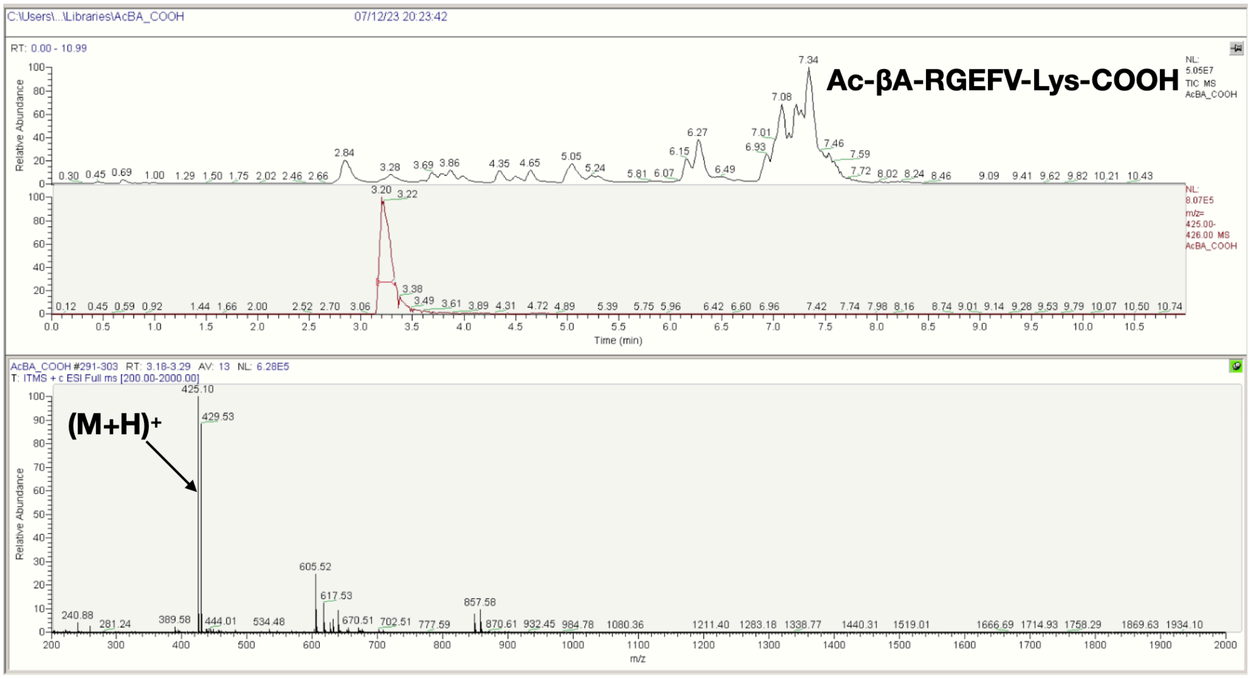
C:\Users\...\Libraries\AcBA_COOH
07/12/23 20:23:42
RT: 0.00 - 10.99
Ac-βA-RGEFV-Lys-COOH
NL: 5.05E7 TIC MS AcBA_COOH
NL: 8.07E5 m/z= 425.00-426.00 MS AcBA_COOH
Relative Abundance
Time (min)
AcBA_COOH #291-303 RT: 3.18-3.29 AV: 13 NL: 6.28E5
T: ITMS + c ESI Full ms [200.00-2000.00]
425.10
429.53
(M+H)+
605.52
617.53
857.58
m/z

K

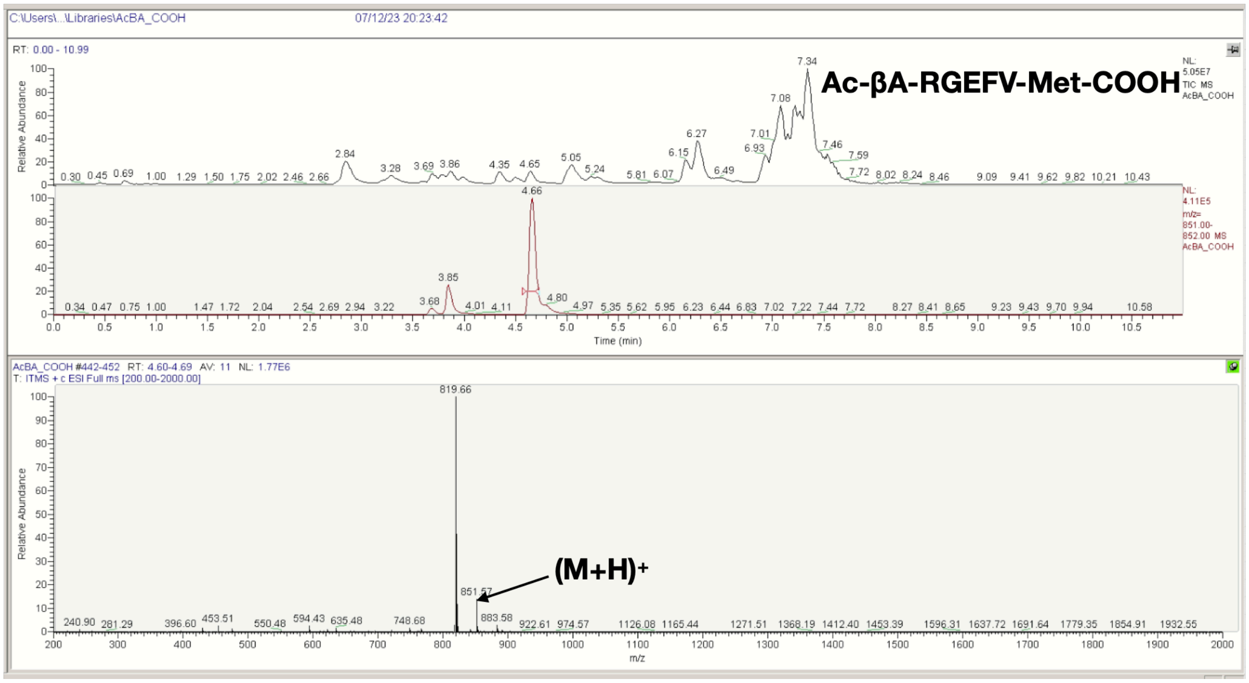

C:\Users\...\Libraries\AcBA_COOH
07/12/23 20:23:42
RT: 0.00 - 10.99
Ac-βA-RGEFV-Met-COOH
Relative Abundance
Time (min)
AcBA_COOH #442-452 RT: 4.60-4.69 AV: 11 NL: 1.77E6
T: ITMS + c ESI Full ms [200.00-2000.00]
819.66
851.67
(M+H)+
m/z

L

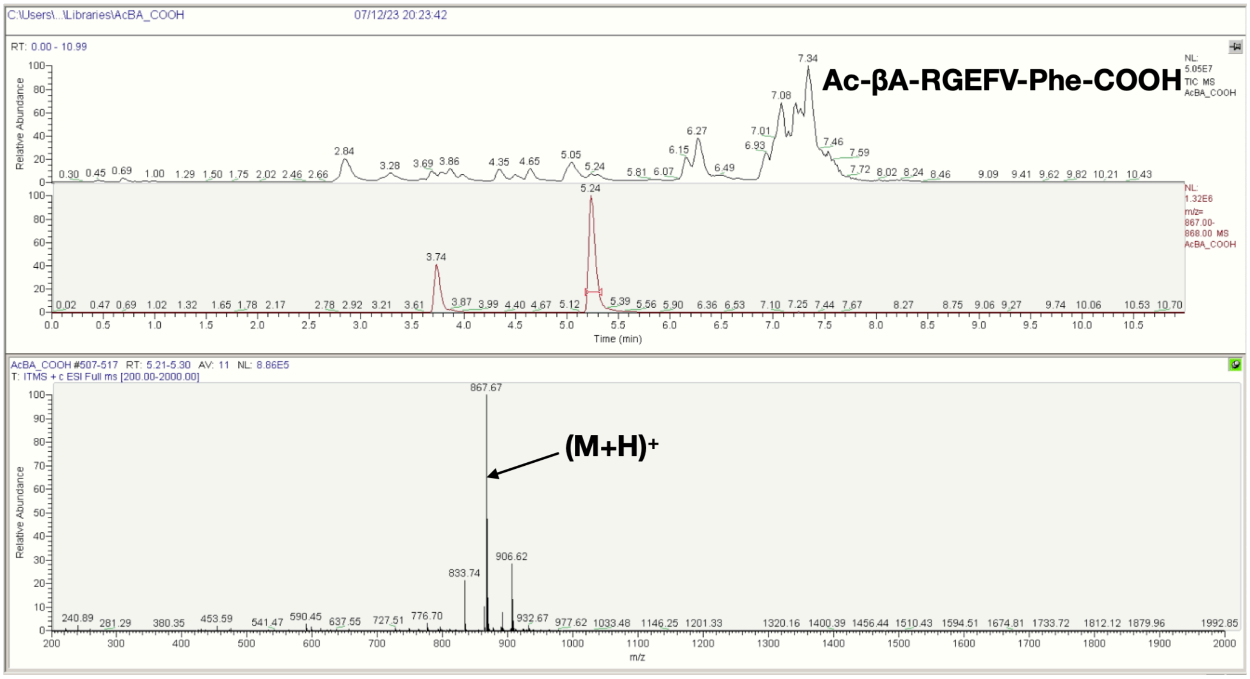

C:\Users\...\Libraries\AcBA_COOH
07/12/23 20:23:42
RT: 0.00 - 10.99
Ac-βA-RGEFV-Phe-COOH
Relative Abundance
Time (min)
AcBA_COOH #507-517 RT: 5.21-5.30 AV: 11 NL: 8.86E5
T: ITMS + c ESI Full ms [200.00-2000.00]
867.67
(M+H)+
833.74
906.62
m/z

M

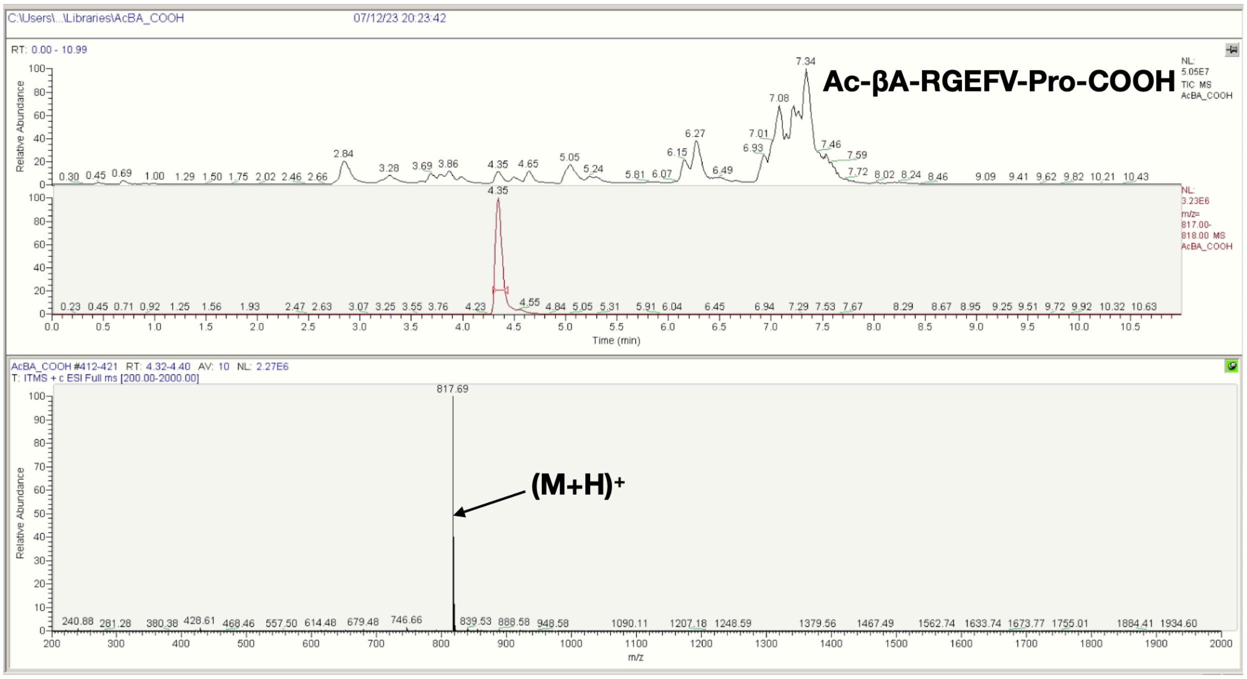

N

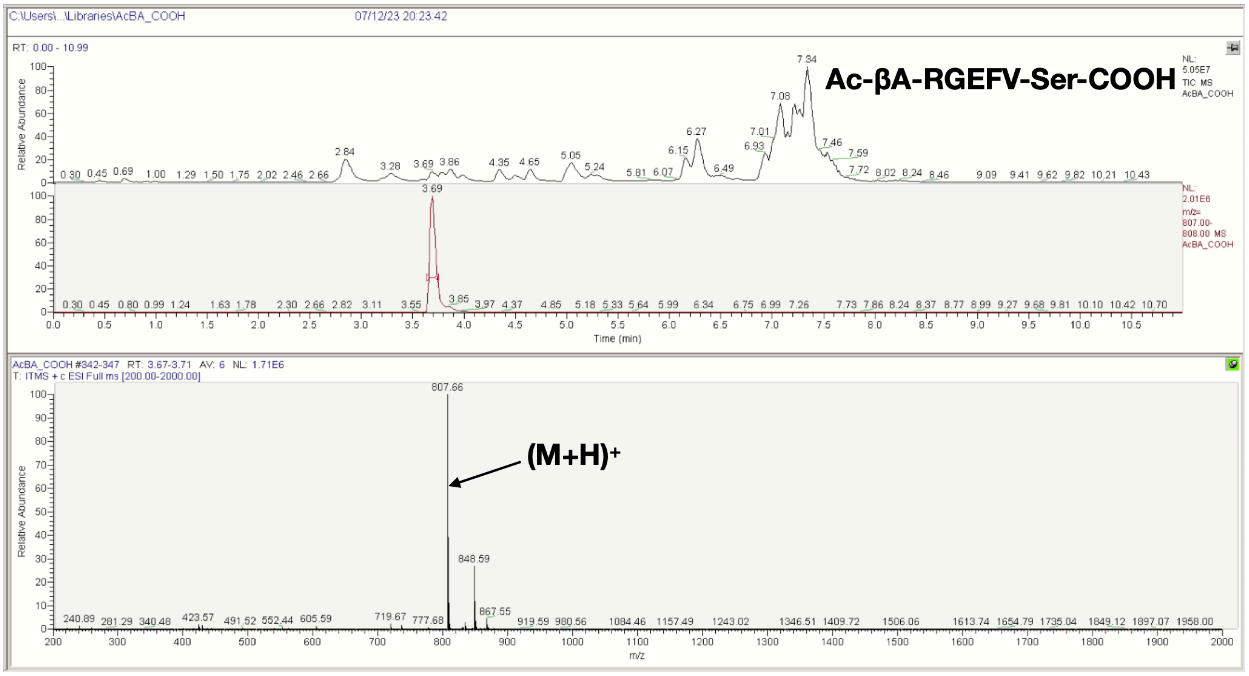

O

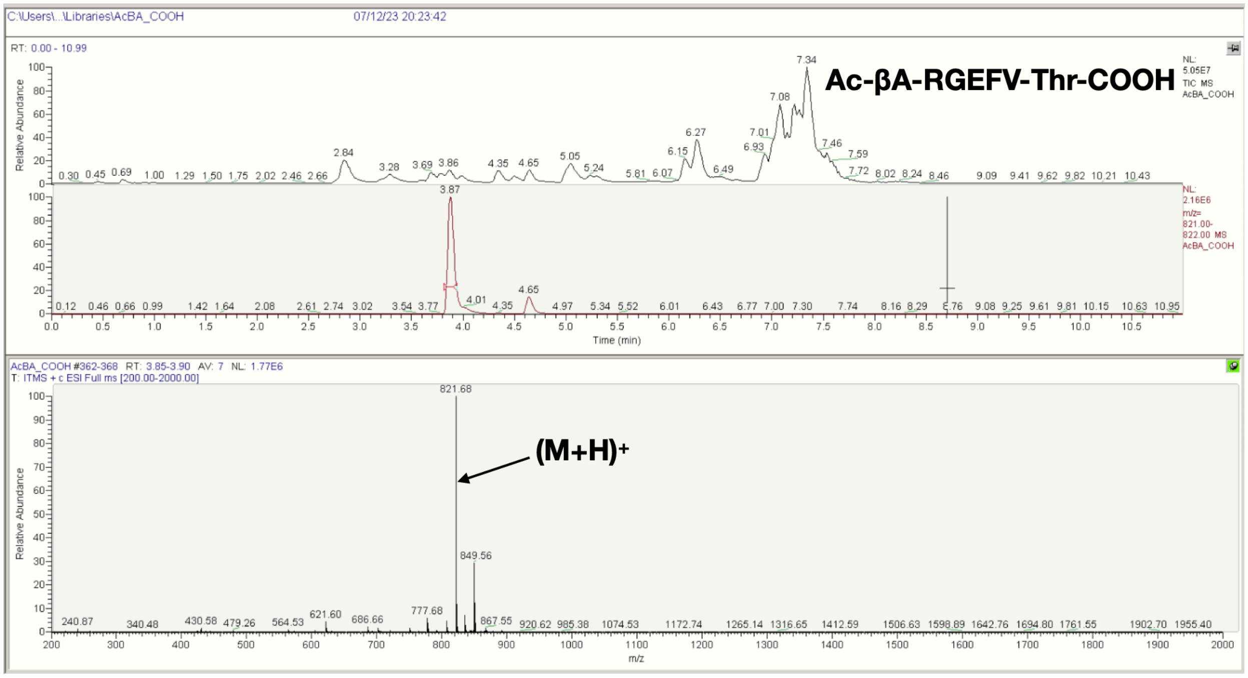
C:\Users\...\Libraries\AcBA_COOH
07/12/23 20:23:42
RT: 0.00 - 10.99
Ac-βA-RGEFV-Thr-COOH
NL: 5.05E7 TIC MS AcBA_COOH
NL: 2.16E6 m/z= 821.00-822.00 MS AcBA_COOH
Relative Abundance
Time (min)
AcBA_COOH #362-368 RT: 3.85-3.90 AV: 7 NL: 1.77E6
T: ITMS + c ESI Full ms [200.00-2000.00]
821.68
849.56
(M+H)+
m/z

P

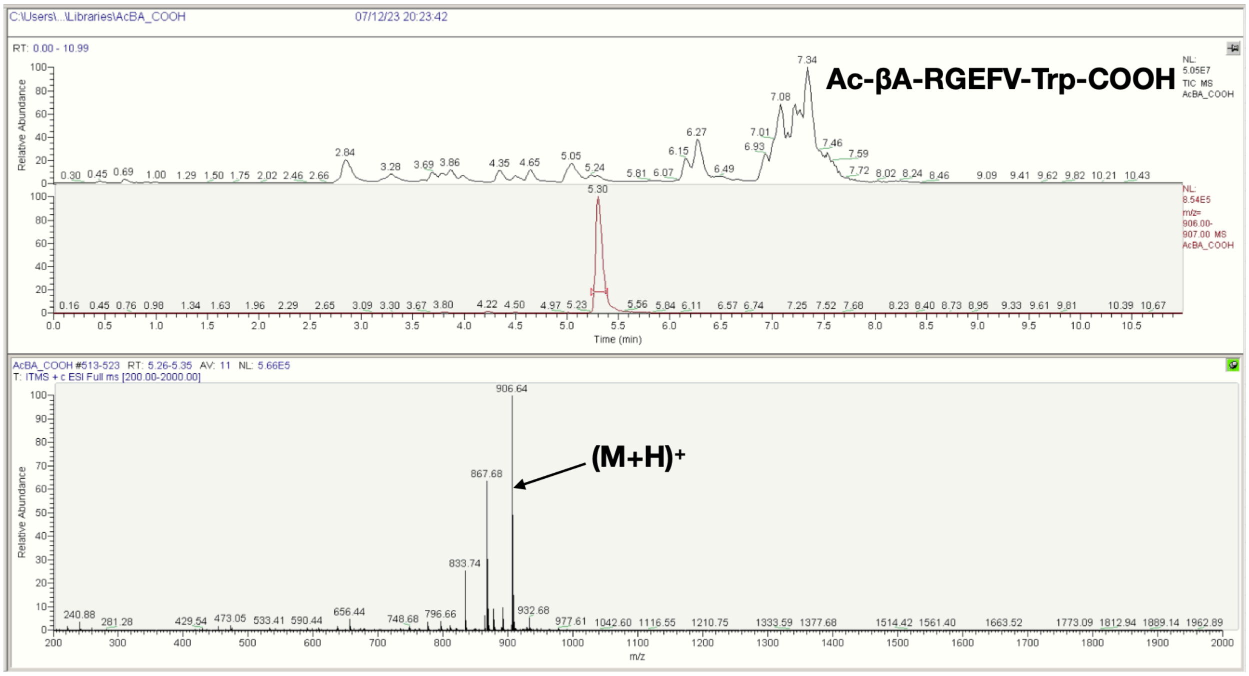
C:\Users\...\Libraries\AcBA_COOH
07/12/23 20:23:42
RT: 0.00 - 10.99
Ac-βA-RGEFV-Trp-COOH
NL: 5.05E7 TIC MS AcBA_COOH
NL: 8.54E5 m/z= 906.00-907.00 MS AcBA_COOH
Relative Abundance
Time (min)
AcBA_COOH #513-523 RT: 5.26-5.35 AV: 11 NL: 5.66E5
T: ITMS + c ESI Full ms [200.00-2000.00]
906.64
867.68
833.74
(M+H)+
m/z

Q

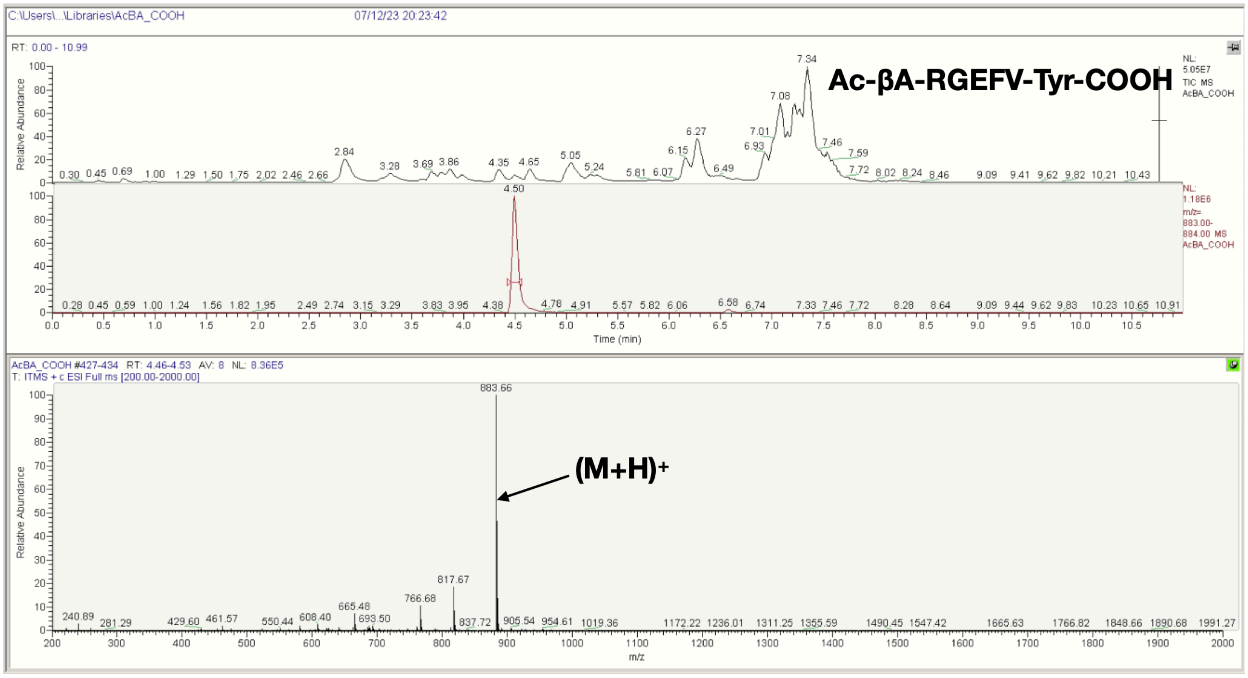

R

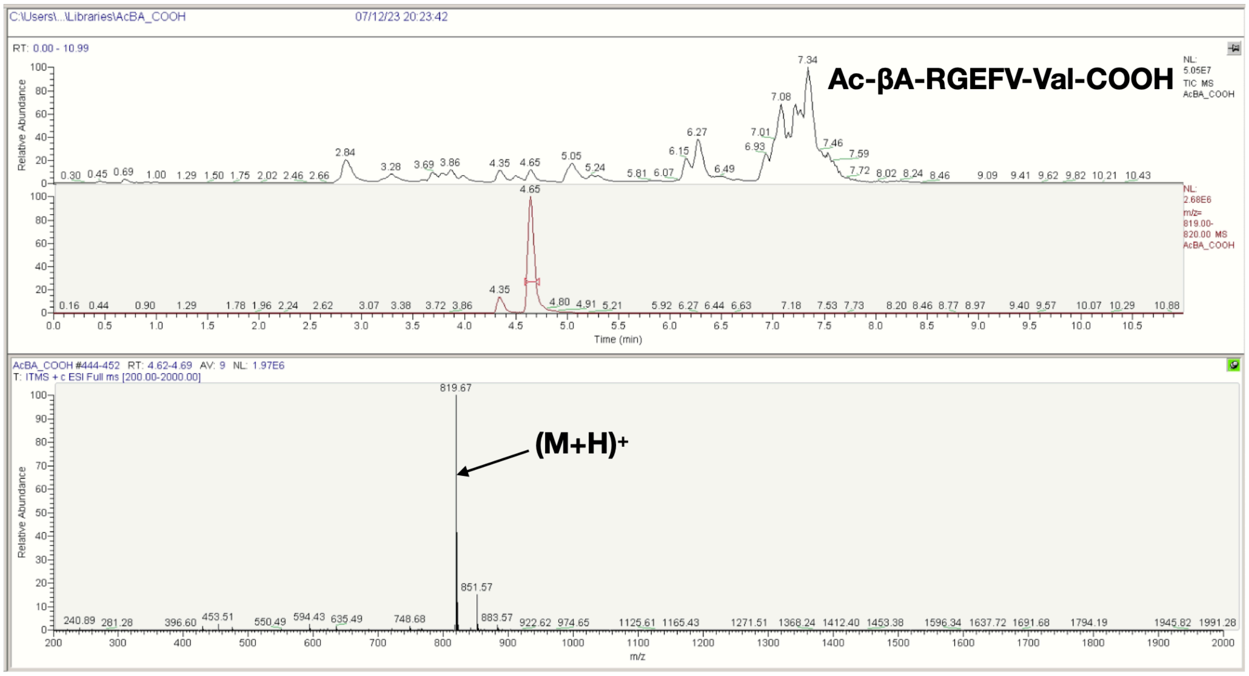

Figure S16. LCMS spectra of the Ac-βA-RGEFV-X-COOH libraries, where X = a) Ala, b) Arg, c) Asn, d) Asp, e) Gln, f) Glu, g) Gly, h) His, i) Ile/Leu, j) Lys, k) Met, l) Phe, m) Pro, n) Ser, o) Thr, p) Trp, q) Tyr, r) Val.

A

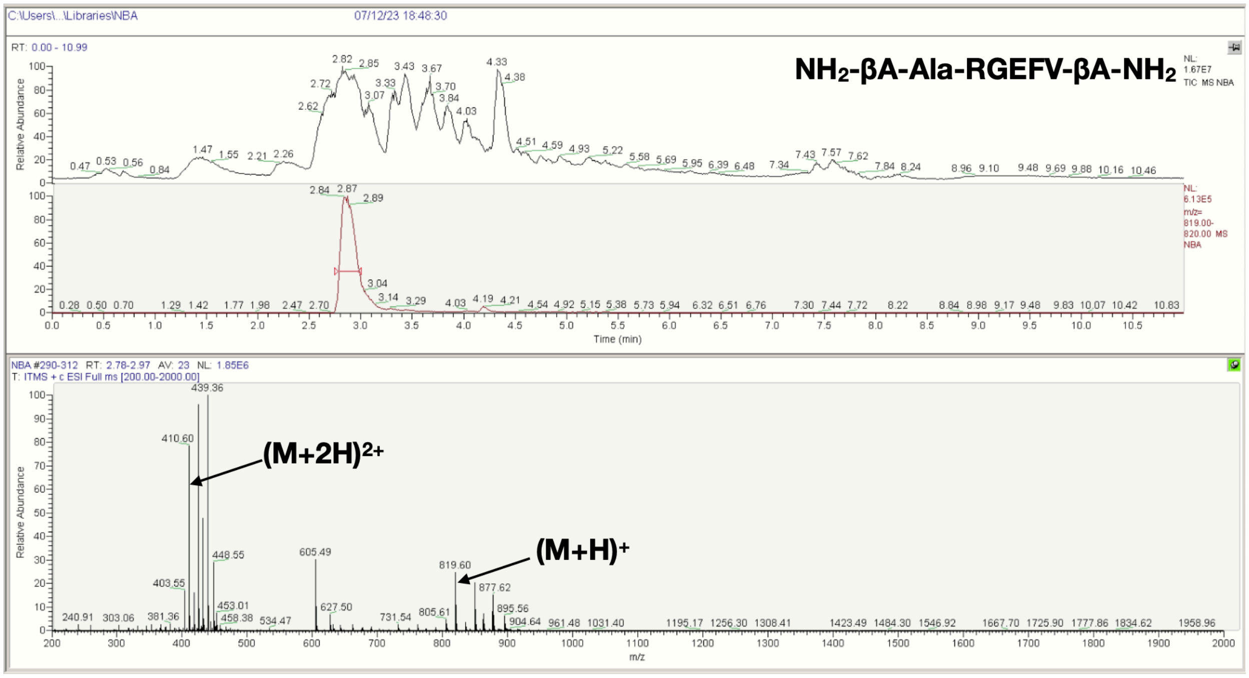
C:\Users\...\Libraries\NBA
07/12/23 18:48:30
NH2-βA-Ala-RGEFV-βA-NH2
Relative Abundance
Time (min)
NBA #290-312 RT: 2.78-2.97 AV: 23 NL: 1.85E6
T: ITMS + c ESI Full ms [200.00-2000.00]
(M+2H)2+
(M+H)+
m/z

B

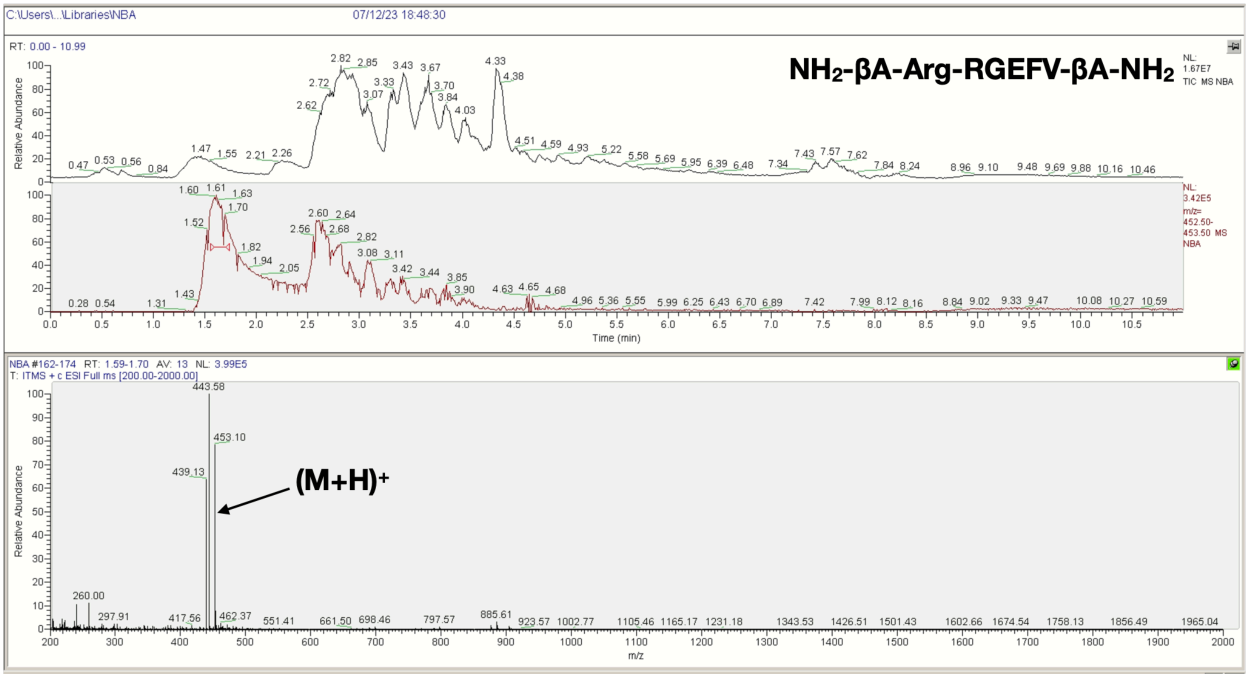
C:\Users\...\Libraries\NBA
07/12/23 18:48:30
NH2-βA-Arg-RGEFV-βA-NH2
Relative Abundance
Time (min)
NBA #162-174 RT: 1.59-1.70 AV: 13 NL: 3.99E5
T: ITMS + c ESI Full ms [200.00-2000.00]
(M+H)+
m/z

C

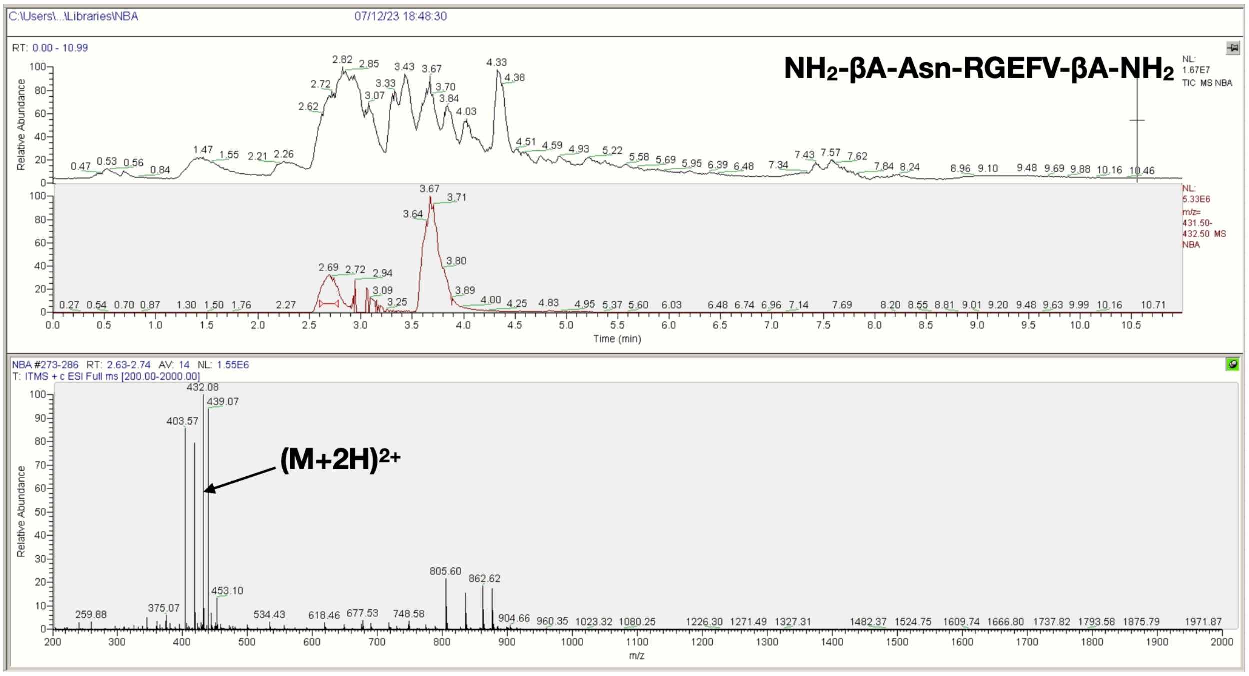
C:\Users\...\Libraries\NBA
07/12/23 18:48:30
RT: 0.00 - 10.99
NH2-βA-Asn-RGEFV-βA-NH2
NL: 1.67E7 TIC MS NBA
NL: 5.33E6 m/z= 431.50-432.50 MS NBA
Relative Abundance
Time (min)
NBA #273-286 RT: 2.63-2.74 AV: 14 NL: 1.55E6
T: ITMS + c ESI Full ms [200.00-2000.00]
432.08
439.07
403.57
(M+2H)2+
805.60
862.62
453.10
m/z

D

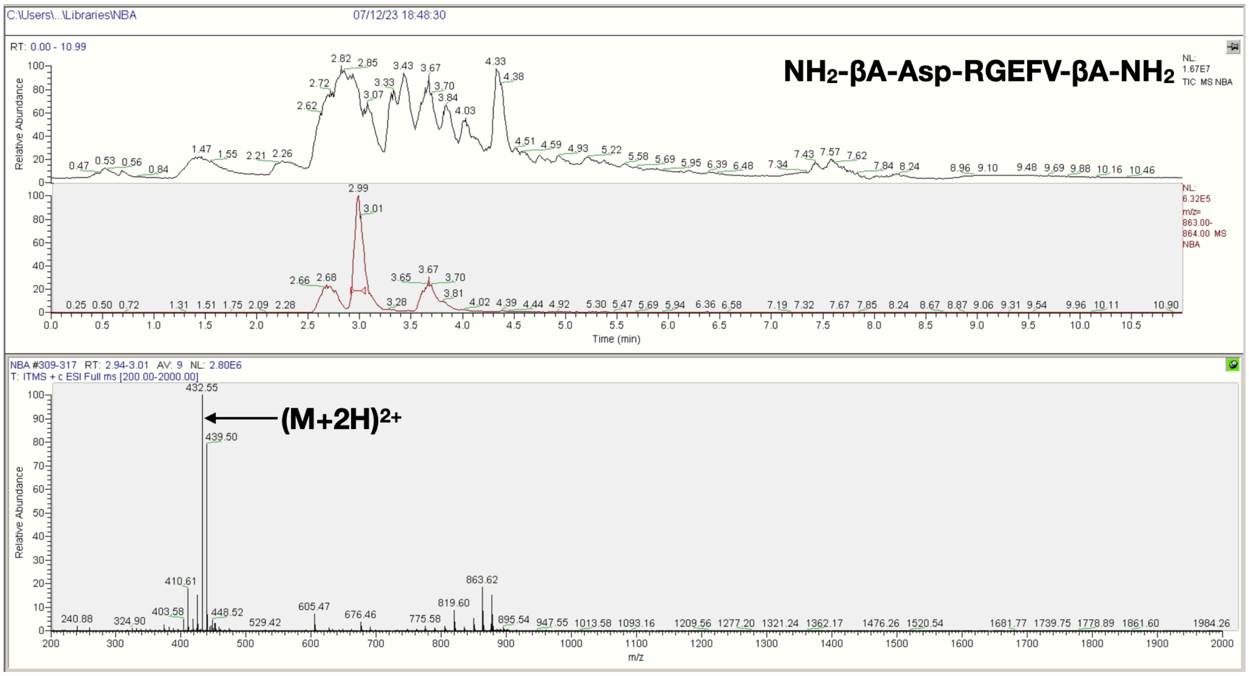
C:\Users\...\Libraries\NBA
07/12/23 18:48:30
RT: 0.00 - 10.99
NH2-βA-Asp-RGEFV-βA-NH2
NL: 1.67E7 TIC MS NBA
NL: 6.32E5 m/z= 863.00-864.00 MS NBA
Relative Abundance
Time (min)
NBA #309-317 RT: 2.94-3.01 AV: 9 NL: 2.80E6
T: ITMS + c ESI Full ms [200.00-2000.00]
432.55
(M+2H)2+
439.50
410.61
863.62
819.60
m/z

E

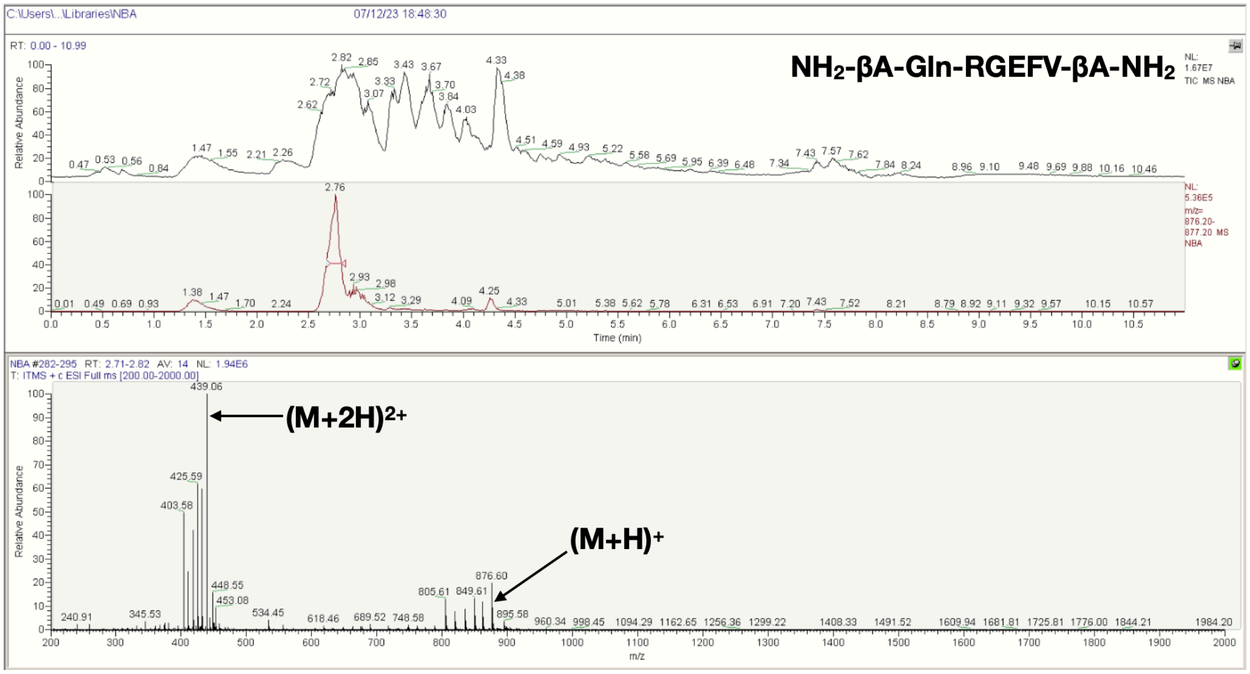
C:\Users\...\Libraries\NBA
07/12/23 18:48:30
NH2-βA-Gln-RGEFV-βA-NH2
Relative Abundance
Time (min)
NBA #282-295 RT: 2.71-2.82 AV: 14 NL: 1.94E6
T: ITMS + c ESI Full ms [200.00-2000.00]
439.06
(M+2H)2+
(M+H)+
876.60
m/z

F

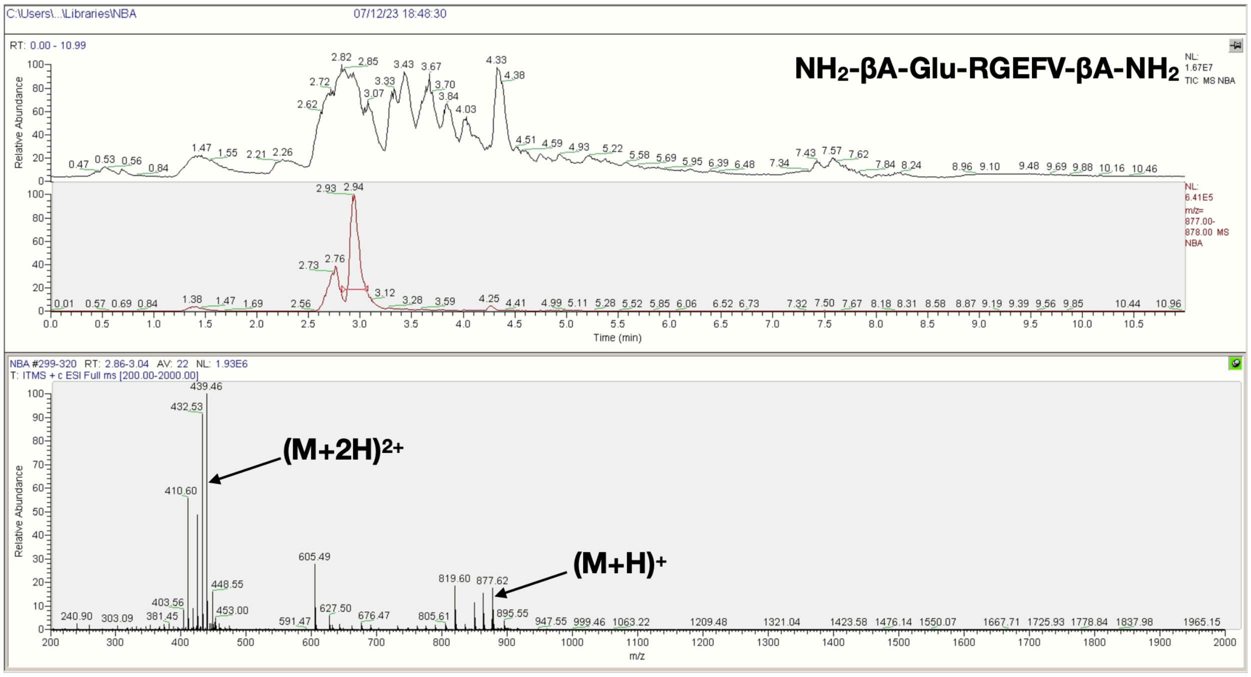
C:\Users\...\Libraries\NBA
07/12/23 18:48:30
NH2-βA-Glu-RGEFV-βA-NH2
Relative Abundance
Time (min)
NBA #299-320 RT: 2.86-3.04 AV: 22 NL: 1.93E6
T: ITMS + c ESI Full ms [200.00-2000.00]
439.46
(M+2H)2+
(M+H)+
877.62
m/z

G

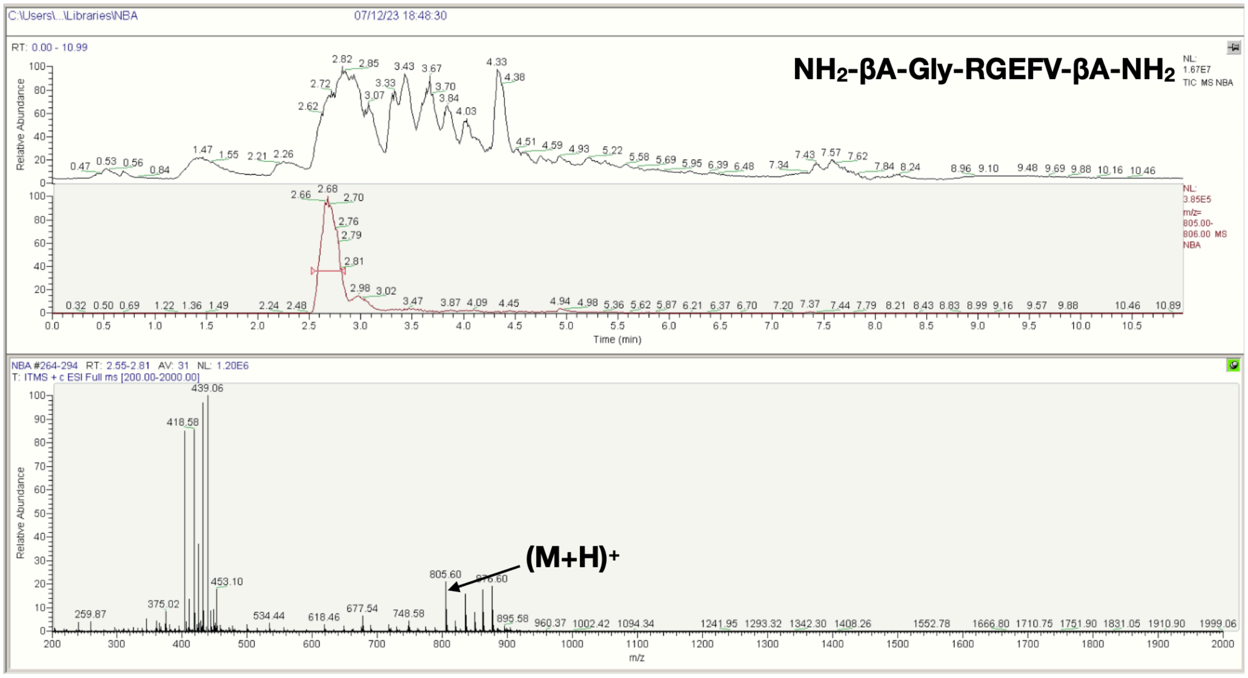
NH2-βA-Gly-RGEFV-βA-NH2
(M+H)+
805.60
418.58
439.06
453.10

H

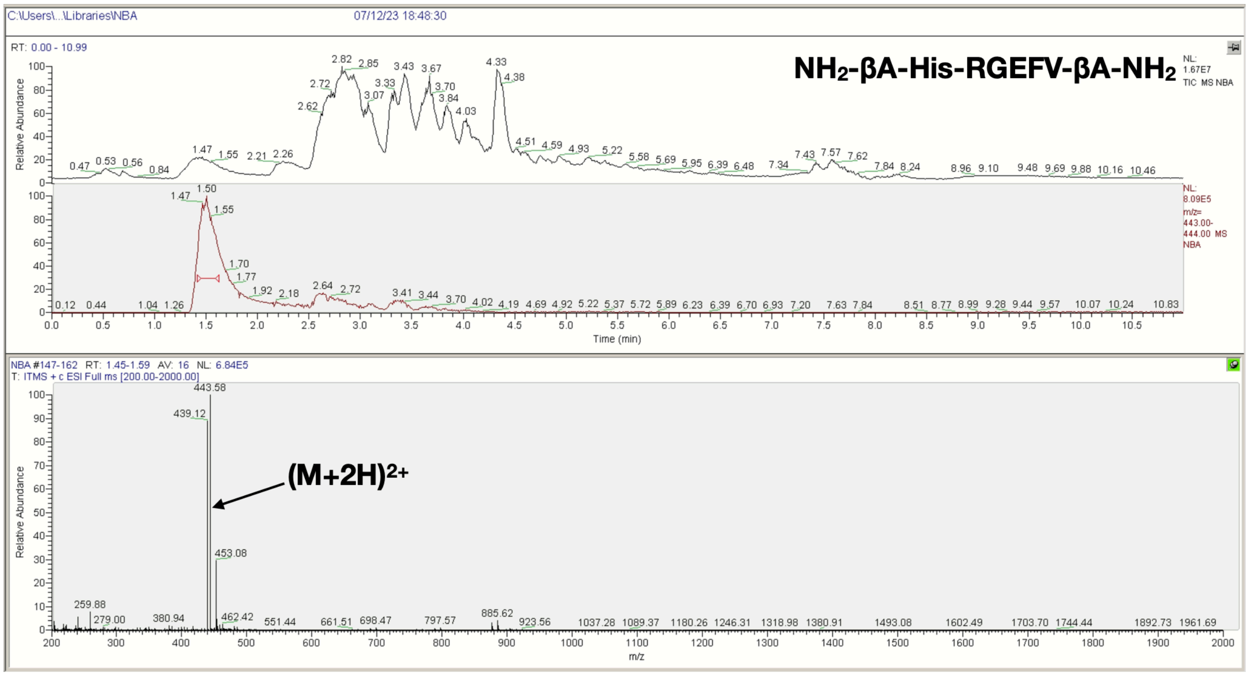
NH2-βA-His-RGEFV-βA-NH2
(M+2H)2+
443.58
439.12
453.08

I

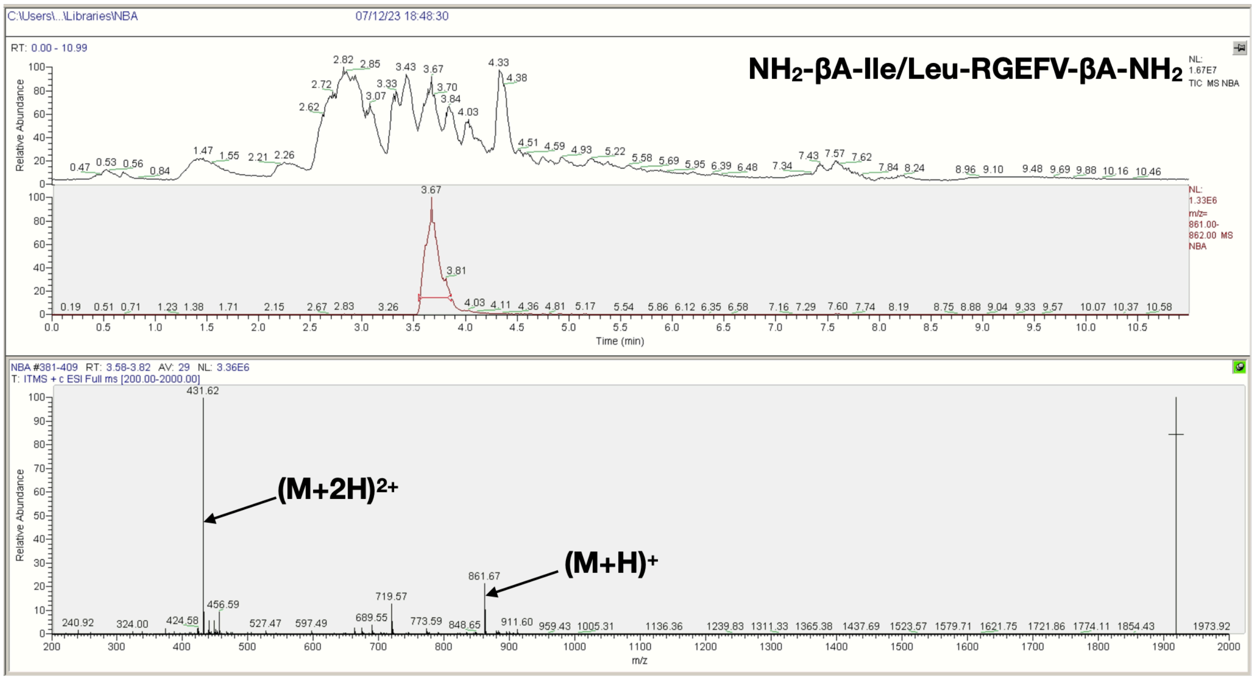
C:\Users\...\Libraries\NBA
07/12/23 18:48:30
RT: 0.00 - 10.99
NH2-βA-Ile/Leu-RGEFV-βA-NH2
NL: 1.67E7 TIC MS NBA
NL: 1.33E6 m/z= 861.00-862.00 MS NBA
Relative Abundance
Time (min)
NBA #381-409 RT: 3.58-3.82 AV: 29 NL: 3.36E6
T: ITMS + c ESI Full ms [200.00-2000.00]
431.62
(M+2H)2+
861.67
(M+H)+
m/z

J

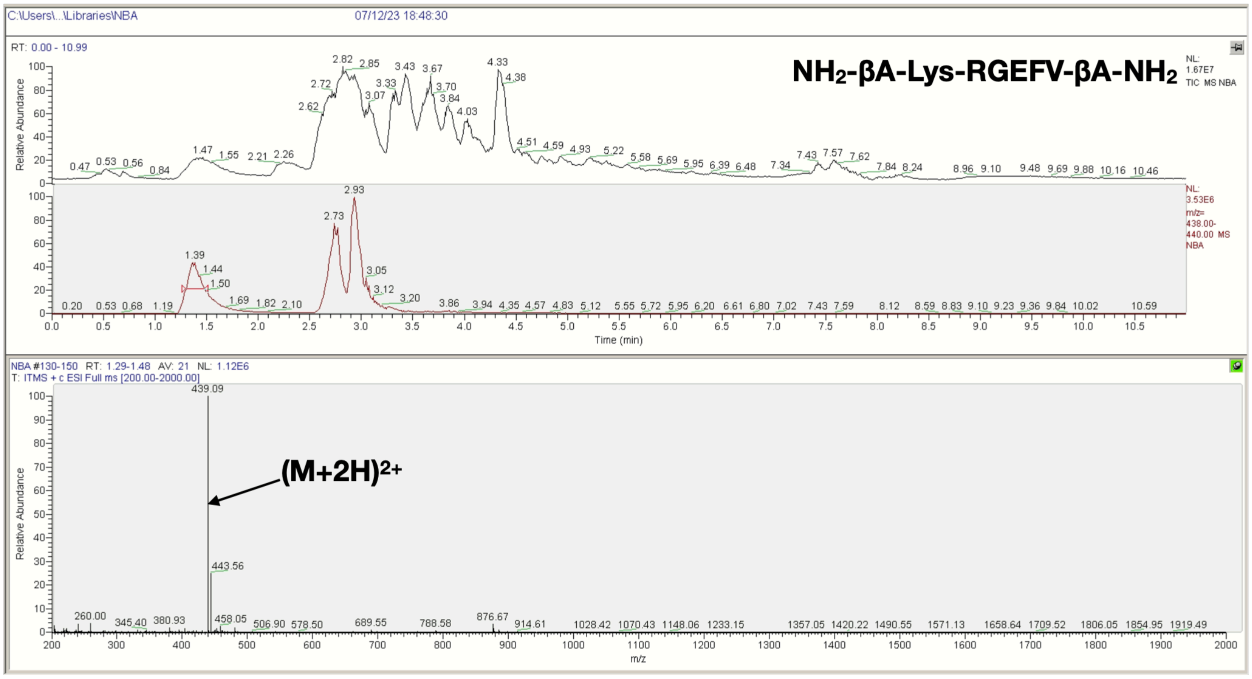
C:\Users\...\Libraries\NBA
07/12/23 18:48:30
RT: 0.00 - 10.99
NH2-βA-Lys-RGEFV-βA-NH2
NL: 1.67E7 TIC MS NBA
NL: 3.53E6 m/z= 438.00-440.00 MS NBA
Relative Abundance
Time (min)
NBA #130-150 RT: 1.29-1.48 AV: 21 NL: 1.12E6
T: ITMS + c ESI Full ms [200.00-2000.00]
439.09
(M+2H)2+
443.56
m/z

K

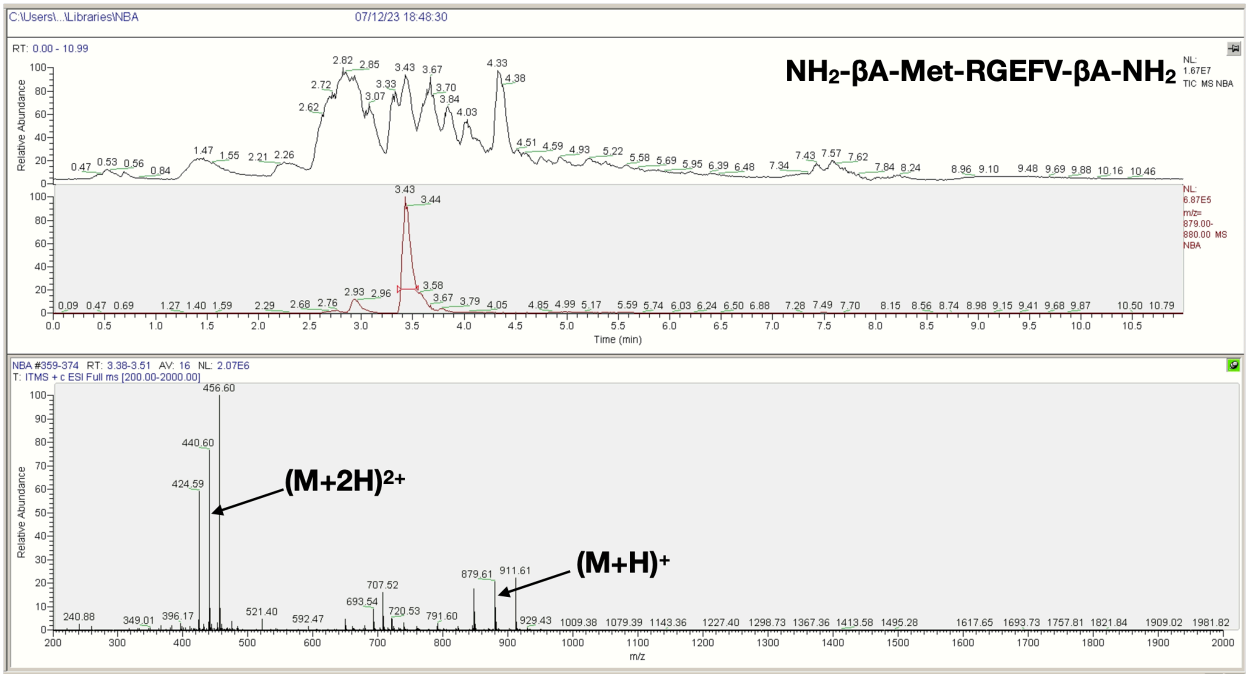
NH2-βA-Met-RGEFV-βA-NH2
(M+2H)2+
(M+H)+
Relative Abundance
Time (min)
m/z

L

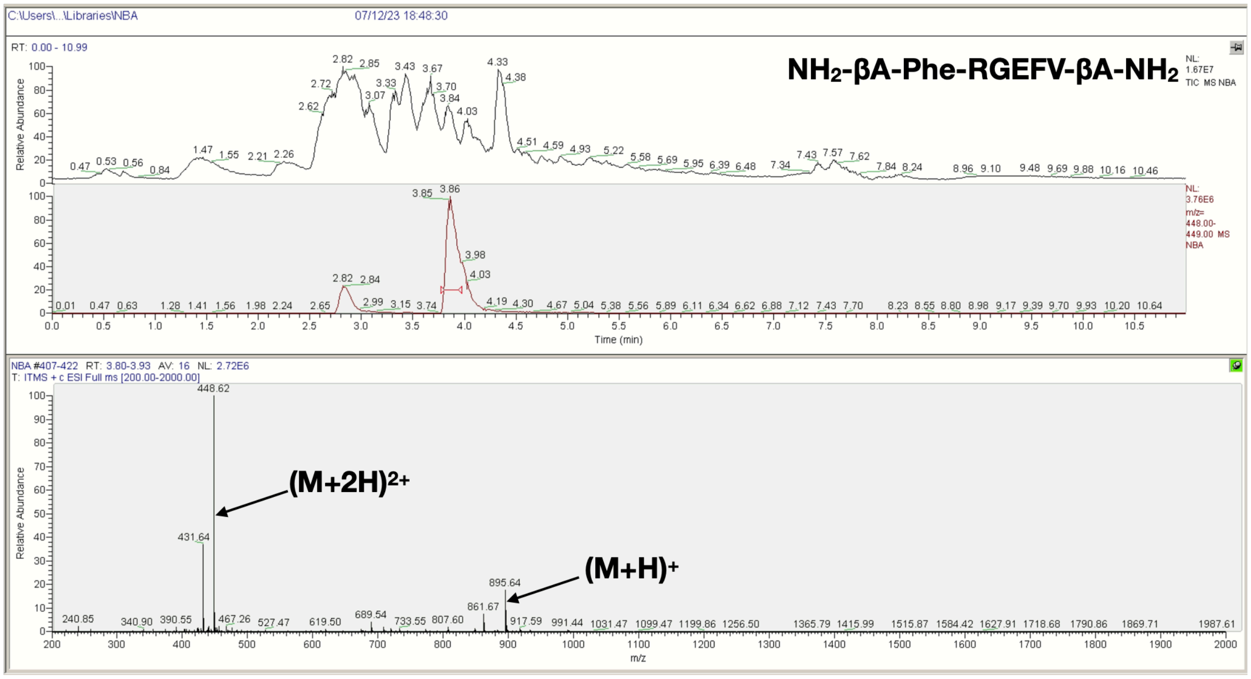
NH2-βA-Phe-RGEFV-βA-NH2
(M+2H)2+
(M+H)+
Relative Abundance
Time (min)
m/z

M

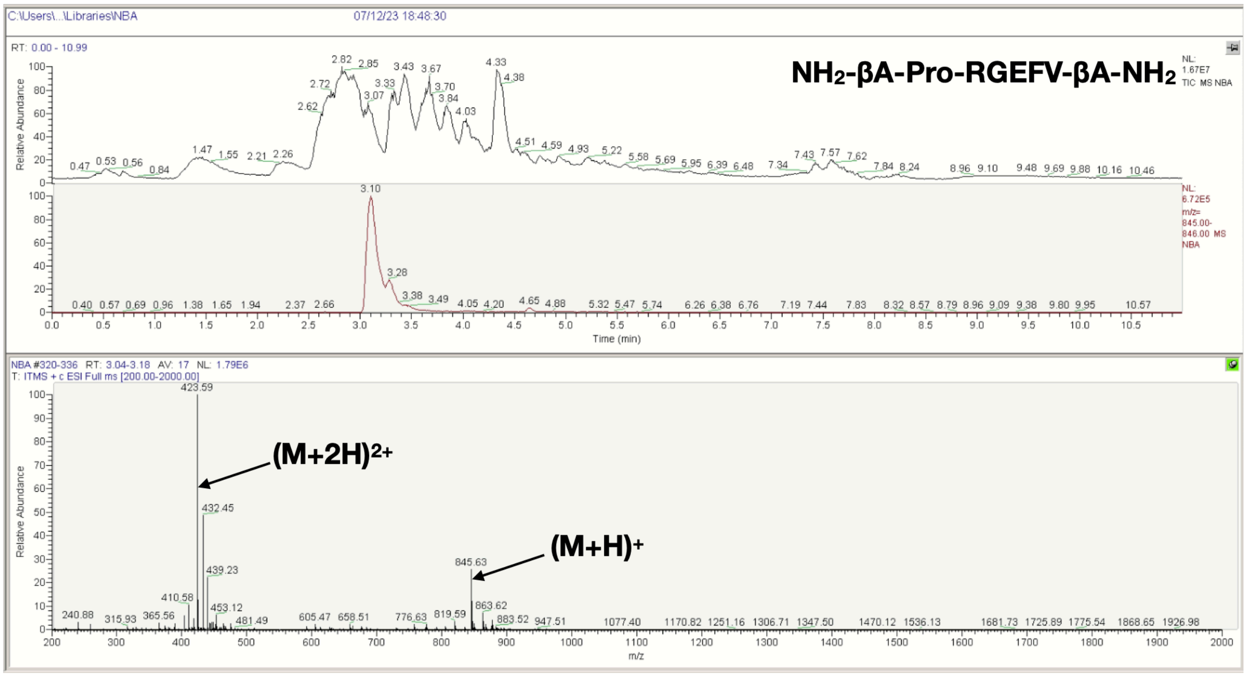
NH2-βA-Pro-RGEFV-βA-NH2
(M+2H)2+
(M+H)+
423.59
845.63

N

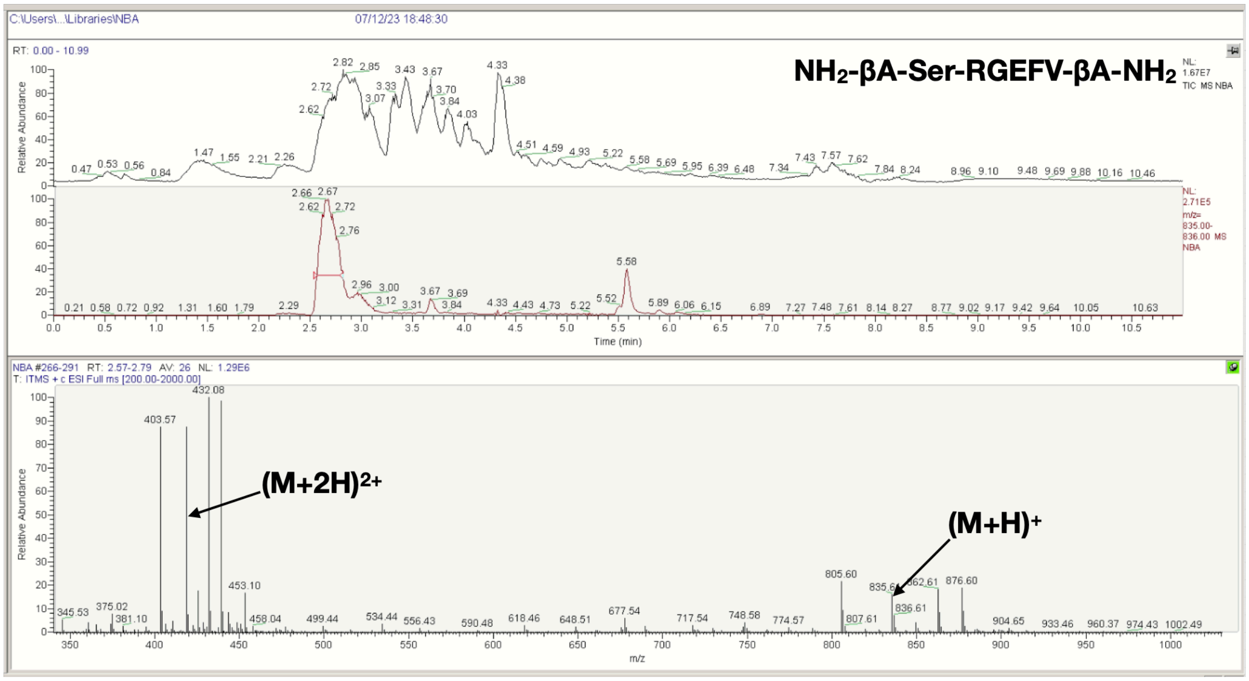
NH2-βA-Ser-RGEFV-βA-NH2
(M+2H)2+
(M+H)+
432.08
403.57

O

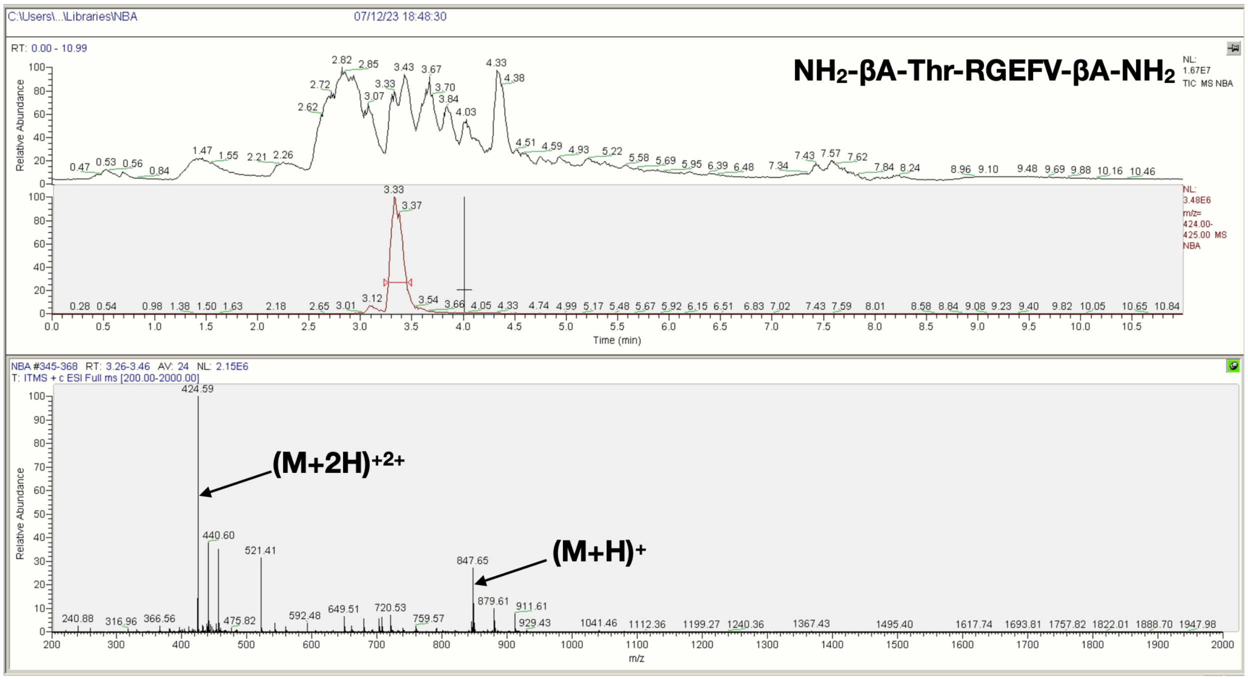

C:\Users\...\Libraries\NBA
07/12/23 18:48:30
RT: 0.00 - 10.99
NH2-βA-Thr-RGEFV-βA-NH2
NL: 1.67E7 TIC MS NBA
NL: 3.48E6 m/z= 424.00-425.00 MS NBA
Relative Abundance
Time (min)
NBA #345-368 RT: 3.26-3.46 AV: 24 NL: 2.15E6
T: ITMS + c ESI Full ms [200.00-2000.00]
424.59
(M+2H)+2+
440.60
521.41
847.65
(M+H)+
879.61
911.61
m/z

P

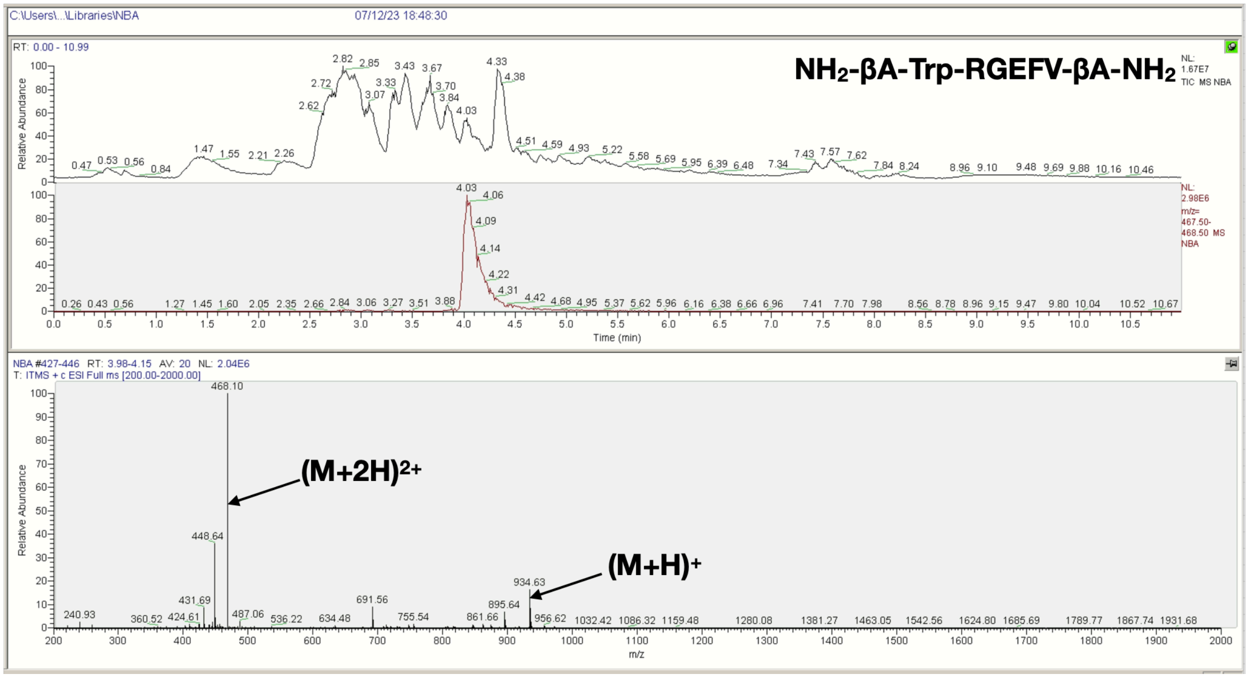

C:\Users\...\Libraries\NBA
07/12/23 18:48:30
RT: 0.00 - 10.99
NH2-βA-Trp-RGEFV-βA-NH2
NL: 1.67E7 TIC MS NBA
NL: 2.98E6 m/z= 467.50-468.50 MS NBA
Relative Abundance
Time (min)
NBA #427-446 RT: 3.98-4.15 AV: 20 NL: 2.04E6
T: ITMS + c ESI Full ms [200.00-2000.00]
468.10
(M+2H)2+
448.64
934.63
(M+H)+
895.64
691.56
m/z

Q

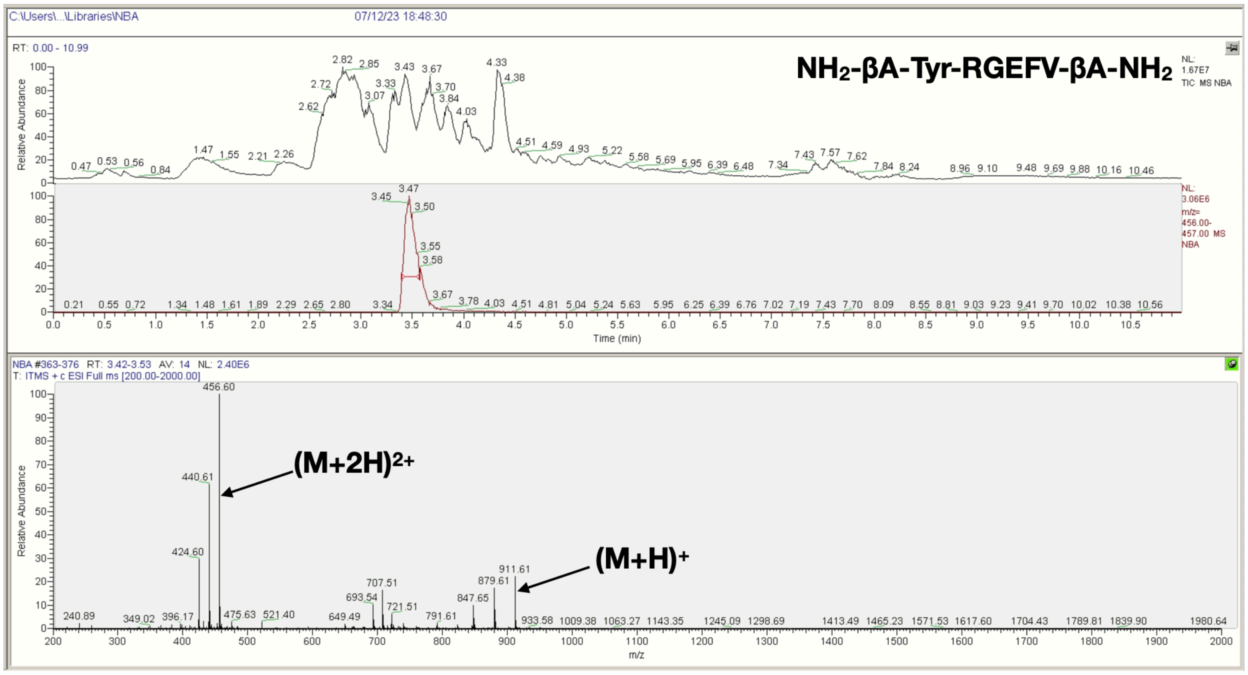

R

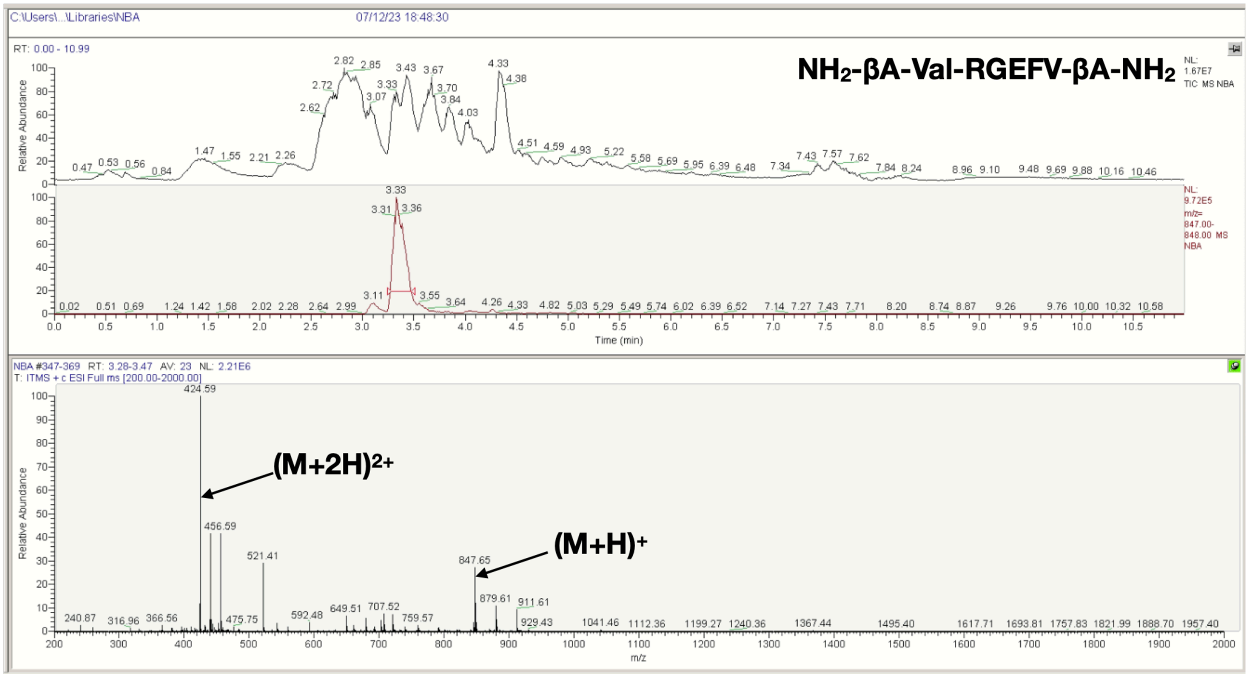

Figure S17. LCMS spectra of the NH_2-βA-X-RGEFV-βA-NH_2 libraries, where X = a) Ala, b) Arg, c) Asn, d) Asp, e) Gln, f) Glu, g) Gly, h) His, i) Ile/Leu, j) Lys, k) Met, l) Phe, m) Pro, n) Ser, o) Thr, p) Trp, q) Tyr, r) Val.

A

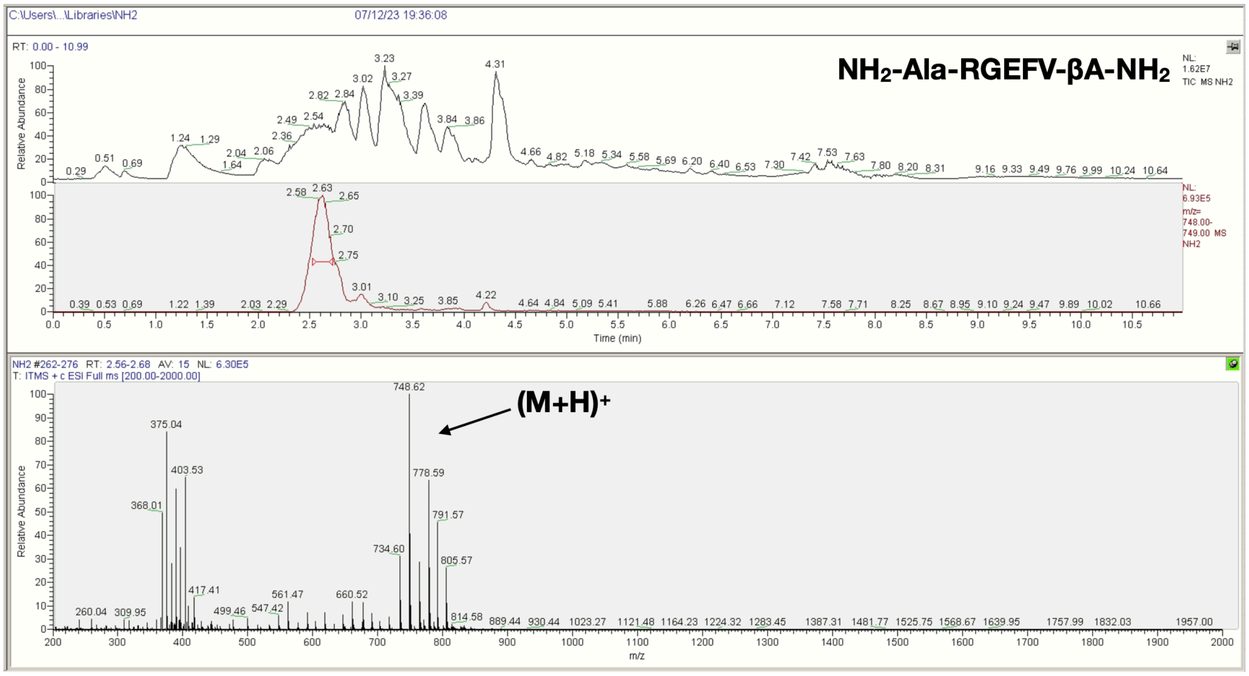

B

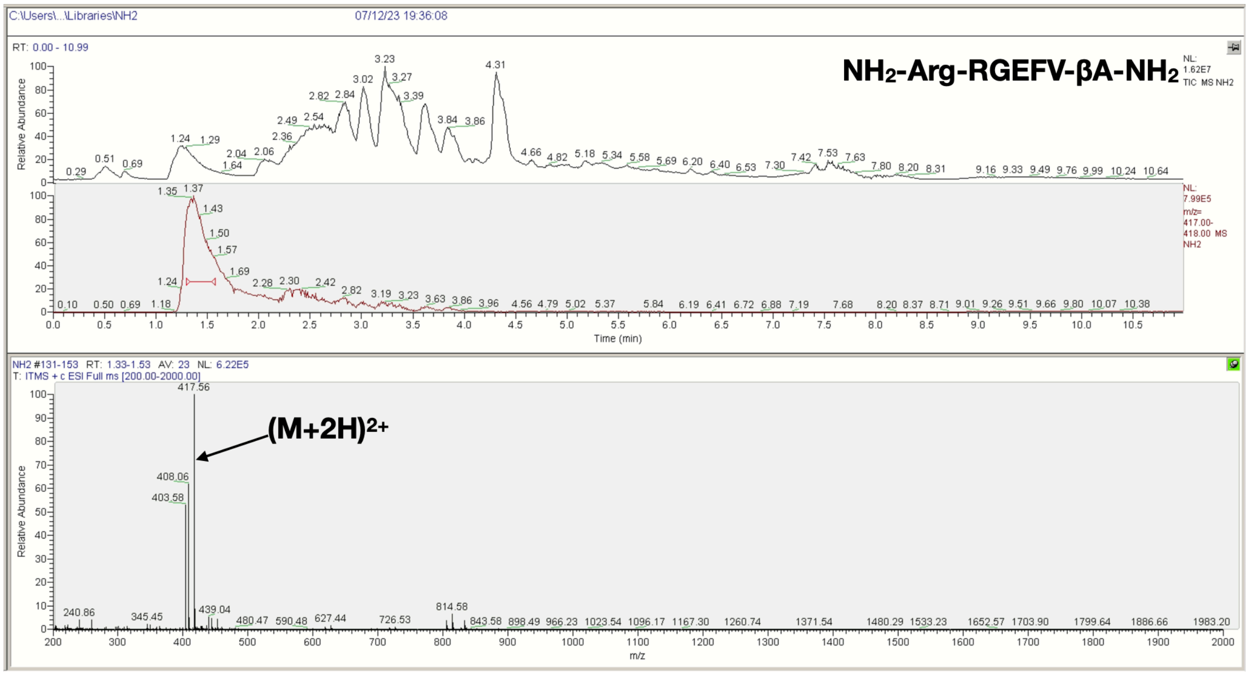

C

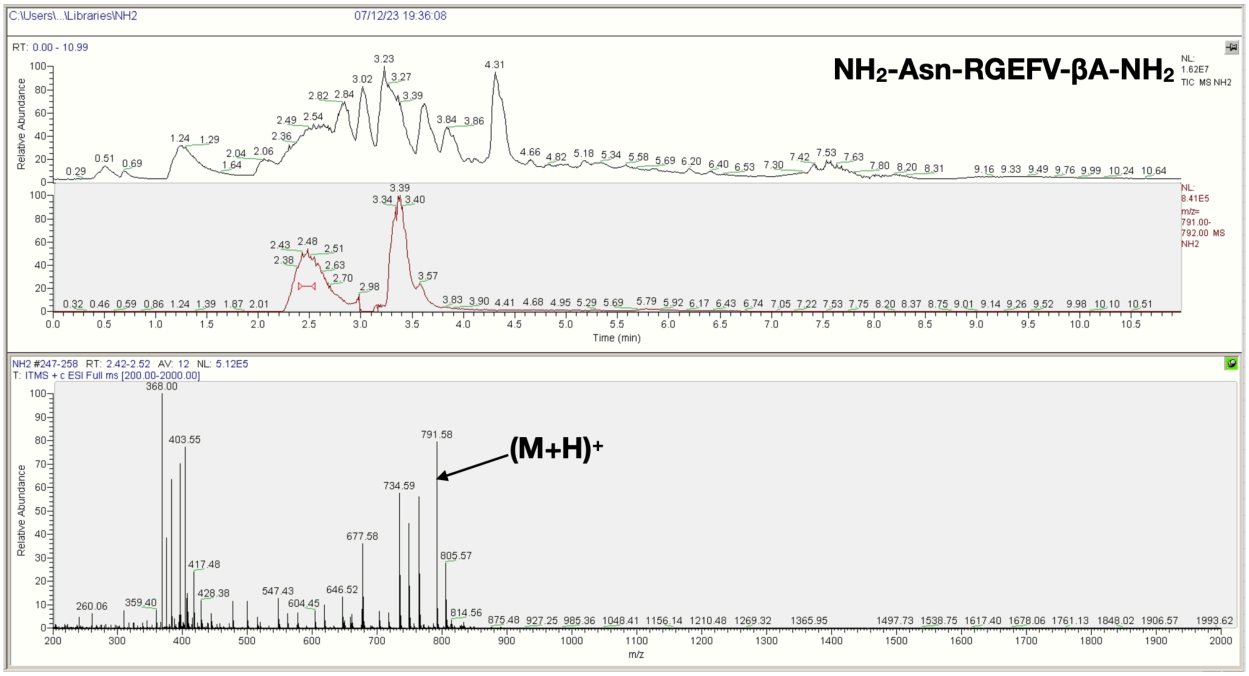
C:\Users\...\Libraries\NH2
07/12/23 19:36:08
RT: 0.00 - 10.99
NH2-Asn-RGEFV-βA-NH2
NL: 1.62E7 TIC MS NH2
NL: 8.41E5 m/z= 791.00-792.00 MS NH2
Relative Abundance
Time (min)
NH2 #247-258 RT: 2.42-2.52 AV: 12 NL: 5.12E5
T: ITMS + c ESI Full ms [200.00-2000.00]
(M+H)+
791.58
m/z

D

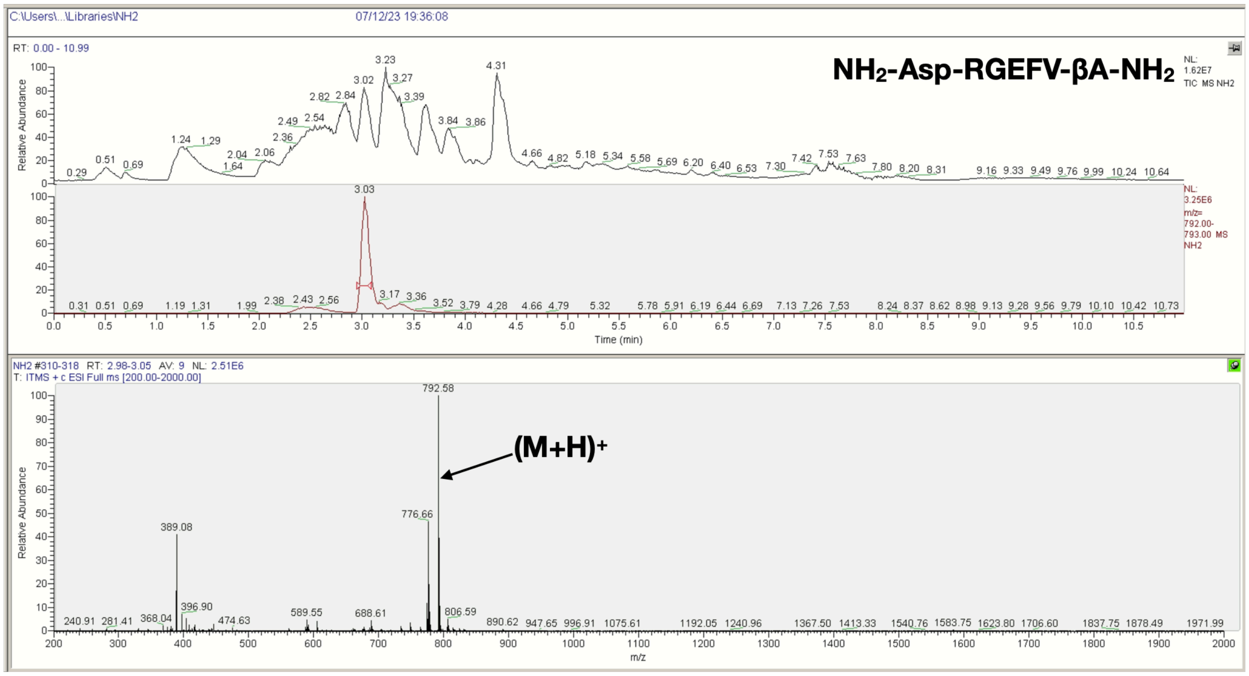
C:\Users\...\Libraries\NH2
07/12/23 19:36:08
RT: 0.00 - 10.99
NH2-Asp-RGEFV-βA-NH2
NL: 1.62E7 TIC MS NH2
NL: 3.25E6 m/z= 792.00-793.00 MS NH2
Relative Abundance
Time (min)
NH2 #310-318 RT: 2.98-3.05 AV: 9 NL: 2.51E6
T: ITMS + c ESI Full ms [200.00-2000.00]
(M+H)+
792.58
m/z

E

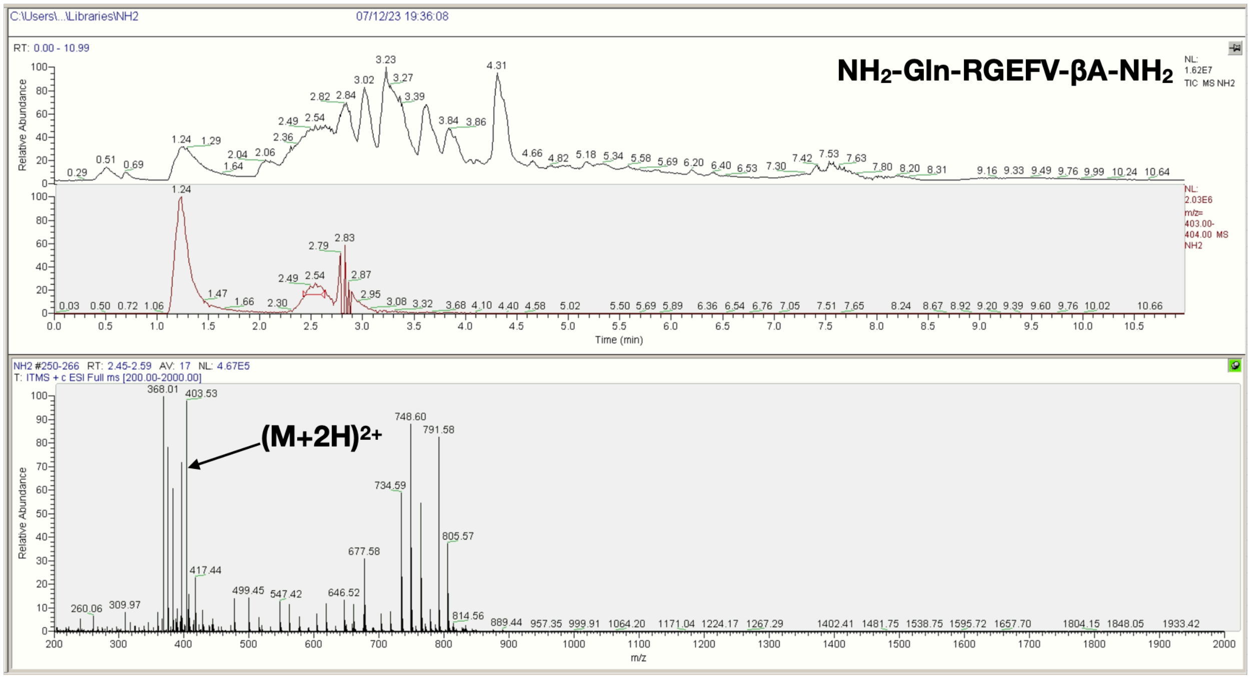
C:\Users\...\Libraries\NH2
07/12/23 19:36:08
RT: 0.00 - 10.99
NH2-Gln-RGEFV-βA-NH2
NL: 1.62E7 TIC MS NH2
NL: 2.03E6 m/z= 403.00-404.00 MS NH2
Relative Abundance
Time (min)
NH2 #250-266 RT: 2.45-2.59 AV: 17 NL: 4.67E5
T: ITMS + c ESI Full ms [200.00-2000.00]
(M+2H)2+
m/z

F

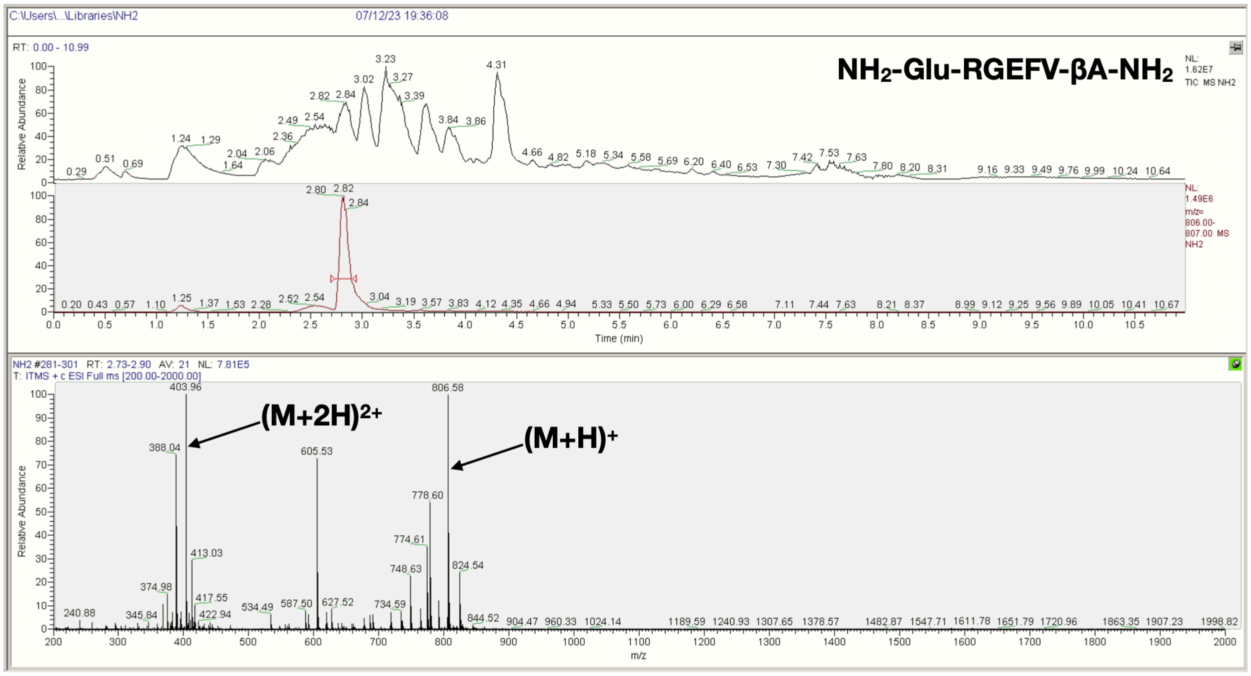
C:\Users\...\Libraries\NH2
07/12/23 19:36:08
RT: 0.00 - 10.99
NH2-Glu-RGEFV-βA-NH2
NL: 1.62E7 TIC MS NH2
NL: 1.49E6 m/z= 806.00-807.00 MS NH2
Relative Abundance
Time (min)
NH2 #281-301 RT: 2.73-2.90 AV: 21 NL: 7.81E5
T: ITMS + c ESI Full ms [200.00-2000.00]
(M+2H)2+
(M+H)+
m/z

G

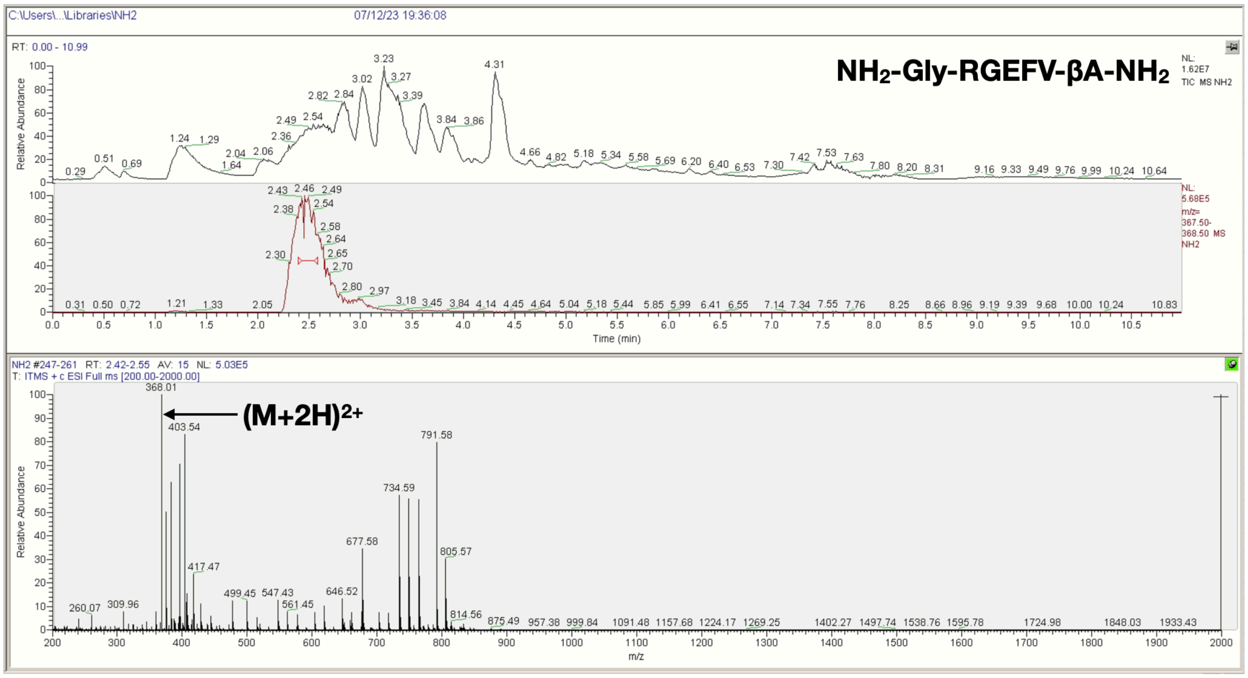

C:\Users\...\Libraries\NH2
07/12/23 19:36:08
RT: 0.00 - 10.99
NH2-Gly-RGEFV-βA-NH2
NL: 1.62E7 TIC MS NH2
NL: 5.68E5 m/z= 367.50-368.50 MS NH2
Relative Abundance
Time (min)
NH2 #247-261 RT: 2.42-2.55 AV: 15 NL: 5.03E5
T: ITMS + c ESI Full ms [200.00-2000.00]
368.01
(M+2H)2+
403.54
791.58
734.59
677.58
805.57
417.47
m/z

H

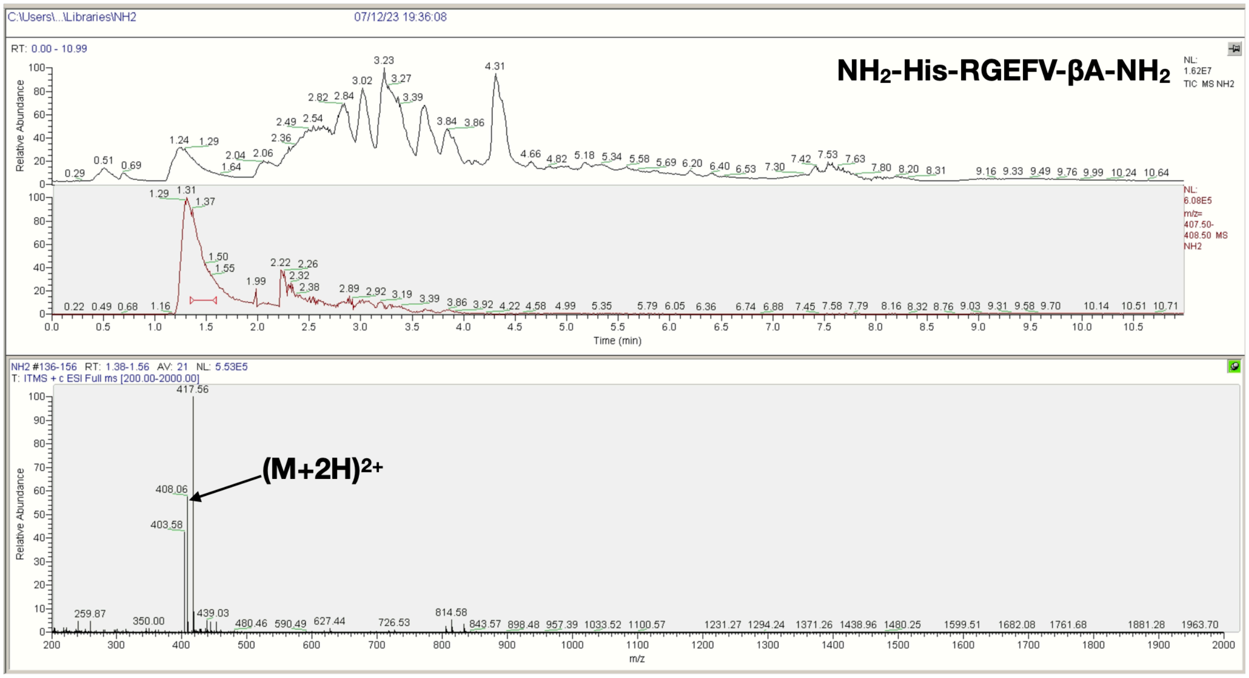

C:\Users\...\Libraries\NH2
07/12/23 19:36:08
RT: 0.00 - 10.99
NH2-His-RGEFV-βA-NH2
NL: 1.62E7 TIC MS NH2
NL: 6.08E5 m/z= 407.50-408.50 MS NH2
Relative Abundance
Time (min)
NH2 #136-156 RT: 1.38-1.56 AV: 21 NL: 5.53E5
T: ITMS + c ESI Full ms [200.00-2000.00]
417.56
(M+2H)2+
408.06
403.58
m/z

I

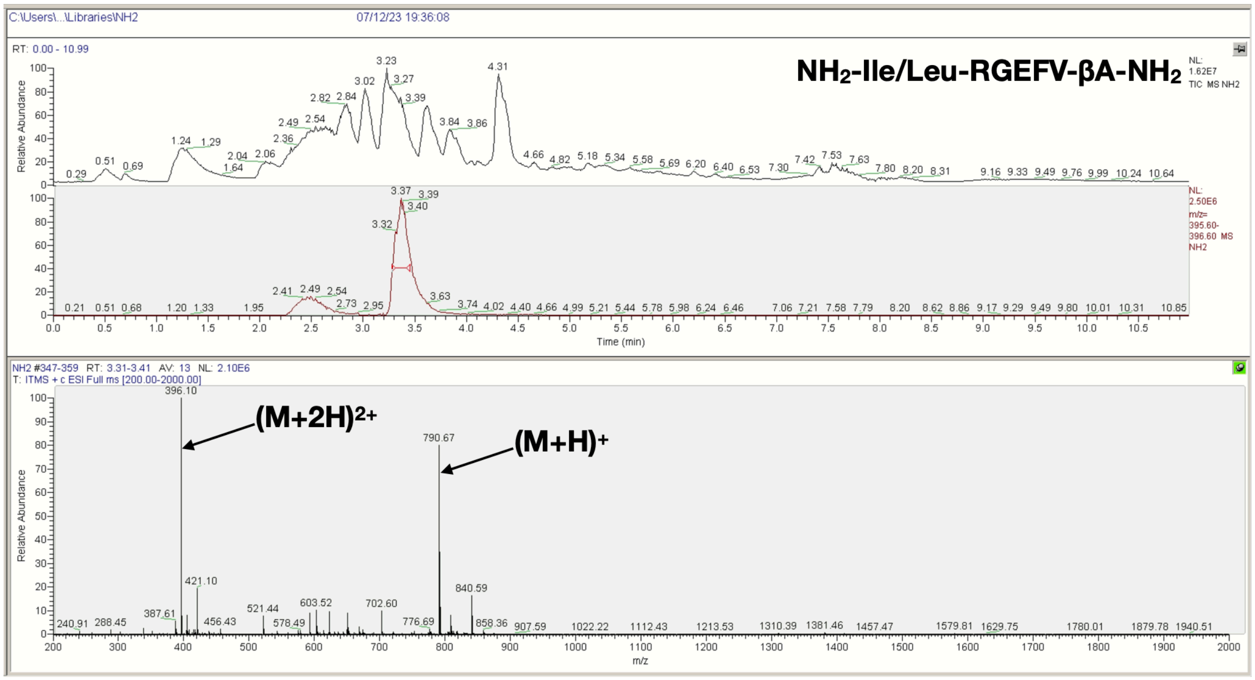
C:\Users\...\Libraries\NH2
07/12/23 19:36:08
NH2-Ile/Leu-RGEFV-βA-NH2
(M+2H)2+
(M+H)+
396.10
790.67
Relative Abundance
Time (min)
m/z

J

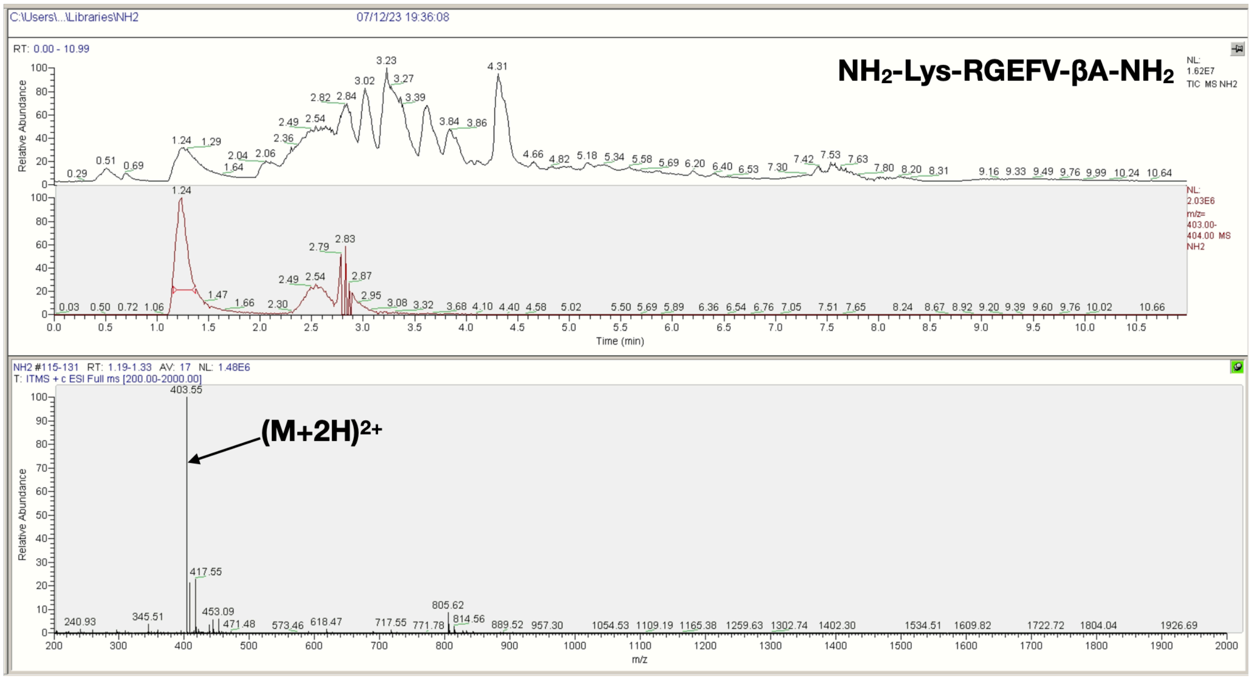
C:\Users\...\Libraries\NH2
07/12/23 19:36:08
NH2-Lys-RGEFV-βA-NH2
(M+2H)2+
403.55
Relative Abundance
Time (min)
m/z

K

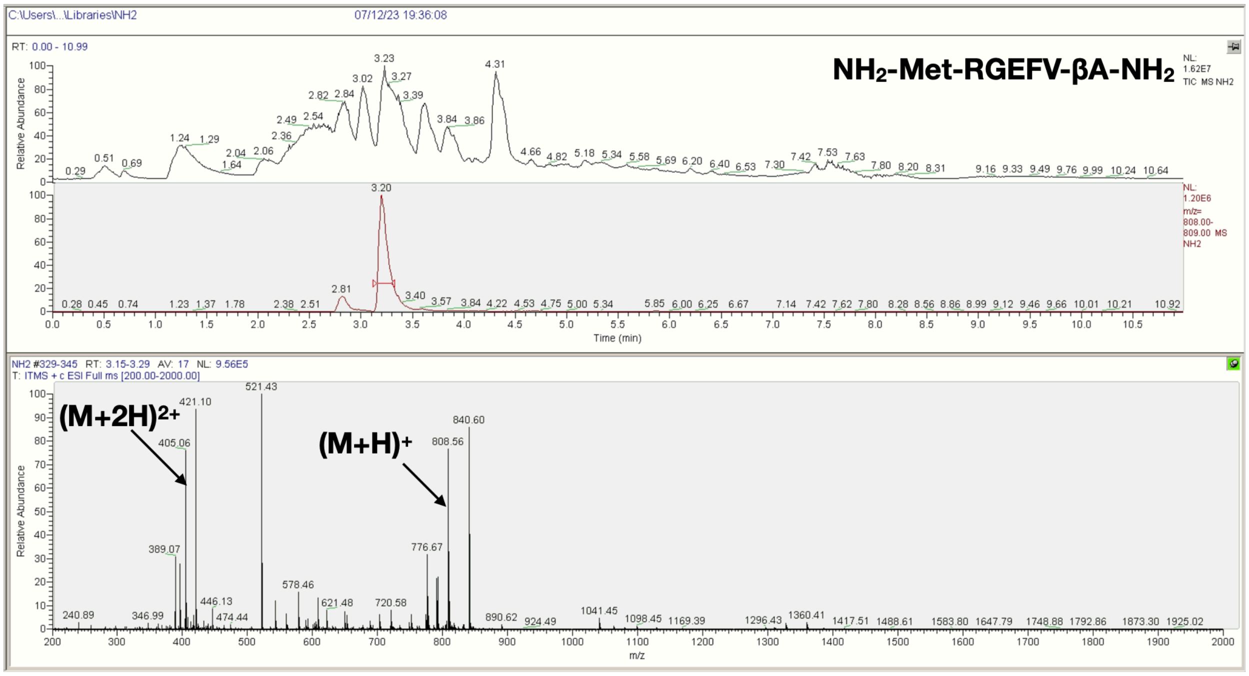
C:\Users\...\Libraries\NH2
07/12/23 19:36:08
RT: 0.00 - 10.99
NH2-Met-RGEFV-βA-NH2
NL: 1.62E7 TIC MS NH2
NL: 1.20E6 m/z= 808.00-809.00 MS NH2
Relative Abundance
Time (min)
NH2 #329-345 RT: 3.15-3.29 AV: 17 NL: 9.56E5
T: ITMS + c ESI Full ms [200.00-2000.00]
(M+2H)2+
(M+H)+
421.10
405.06
521.43
840.60
808.56
776.67
389.07
m/z

L

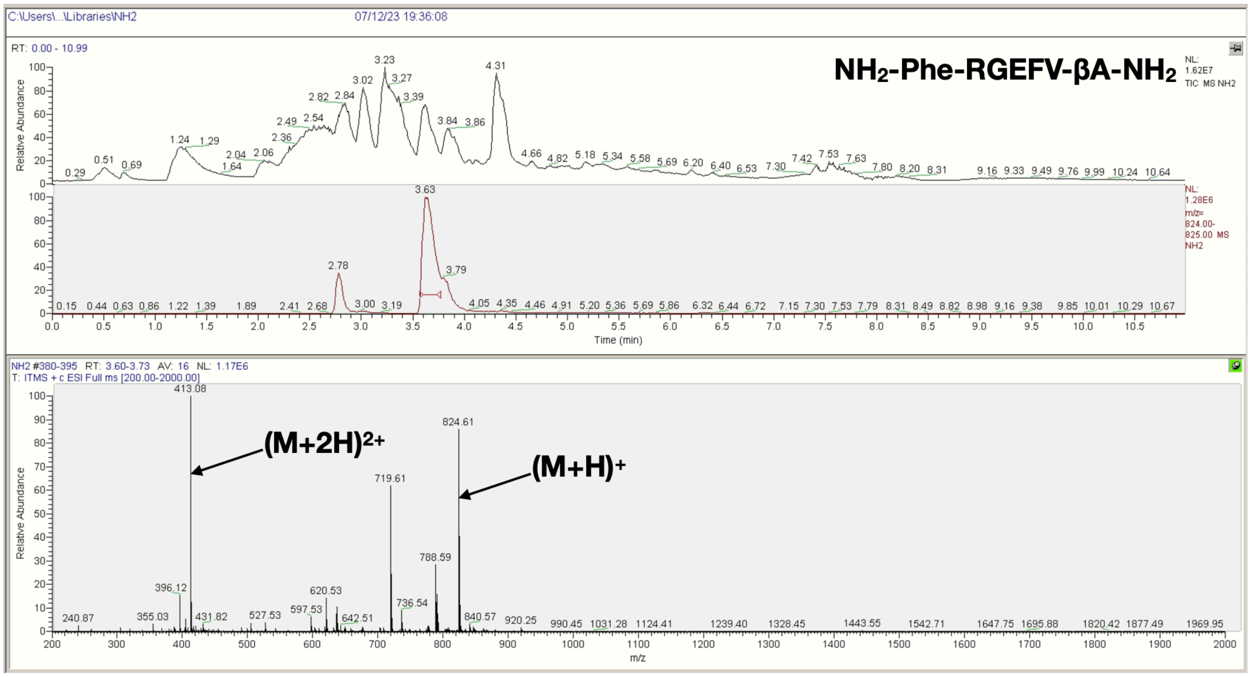
C:\Users\...\Libraries\NH2
07/12/23 19:36:08
RT: 0.00 - 10.99
NH2-Phe-RGEFV-βA-NH2
NL: 1.62E7 TIC MS NH2
NL: 1.28E6 m/z= 824.00-825.00 MS NH2
Relative Abundance
Time (min)
NH2 #380-395 RT: 3.60-3.73 AV: 16 NL: 1.17E6
T: ITMS + c ESI Full ms [200.00-2000.00]
(M+2H)2+
(M+H)+
413.08
824.61
719.61
788.59
m/z

M

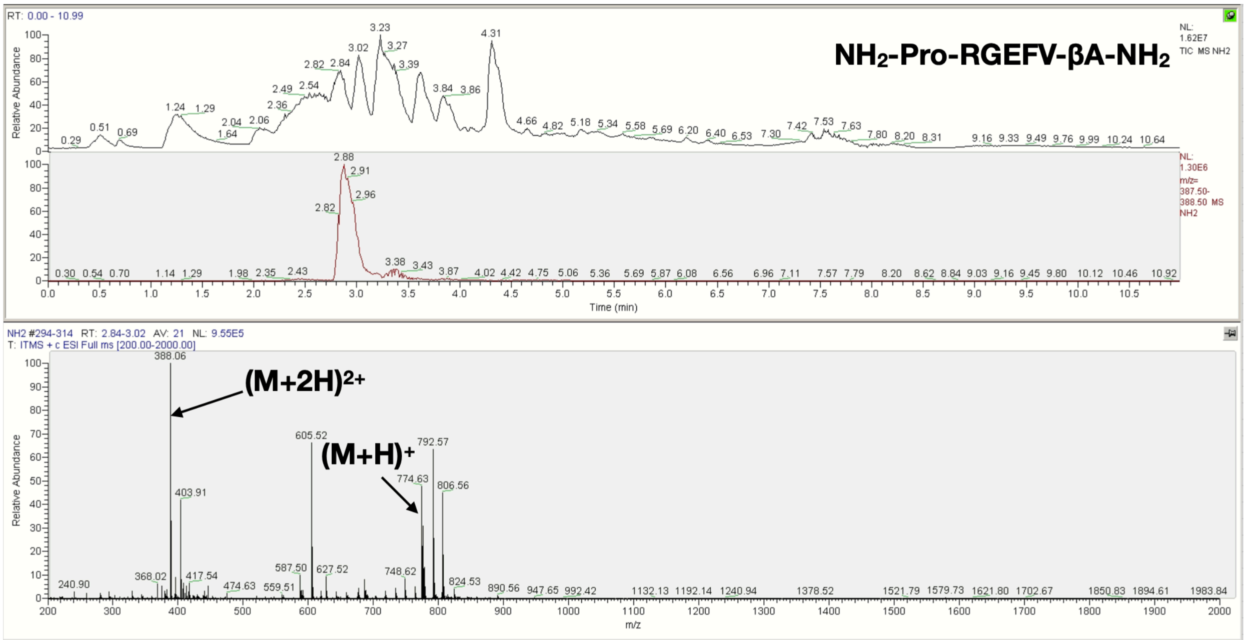
NH2-Pro-RGEFV-βA-NH2
RT: 0.00 - 10.99
Relative Abundance
Time (min)
NH2 #294-314 RT: 2.84-3.02 AV: 21 NL: 9.55E5
T: ITMS + c ESI Full ms [200.00-2000.00]
(M+2H)2+
(M+H)+
388.06
605.52
792.57
774.63
806.56
403.91
m/z

N

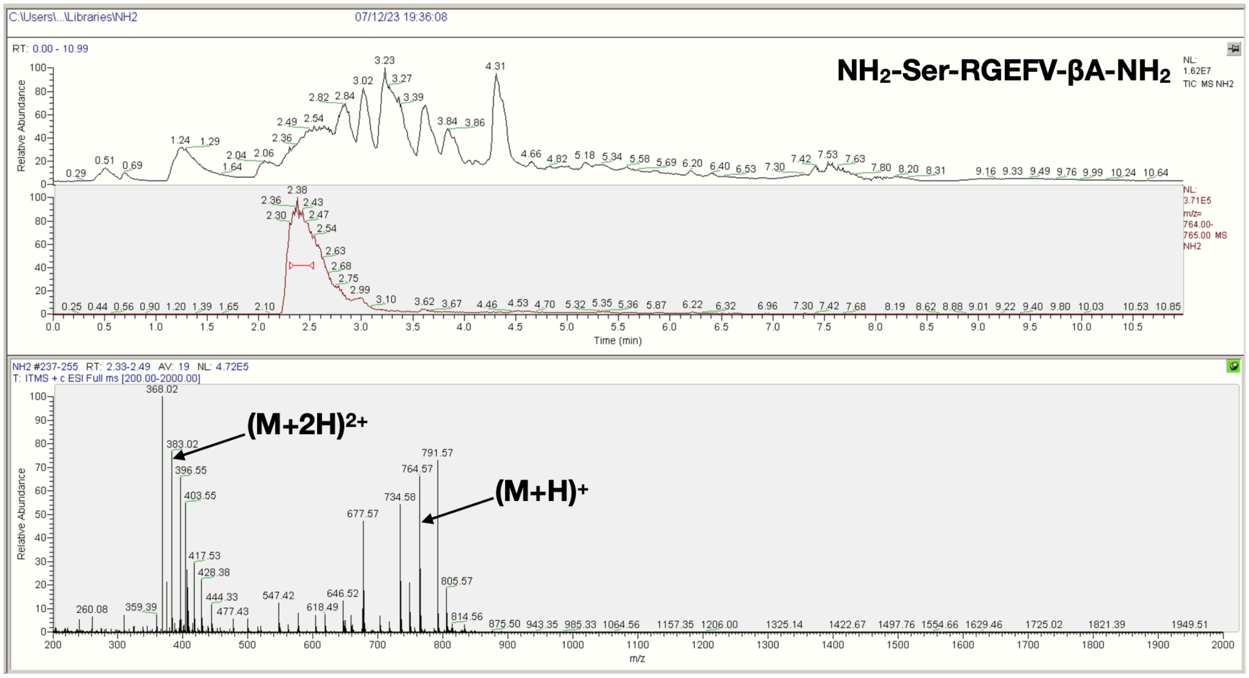
C:\Users\...\Libraries\NH2
07/12/23 19:36:08
NH2-Ser-RGEFV-βA-NH2
RT: 0.00 - 10.99
Relative Abundance
Time (min)
NH2 #237-255 RT: 2.33-2.49 AV: 19 NL: 4.72E5
T: ITMS + c ESI Full ms [200.00-2000.00]
(M+2H)2+
(M+H)+
368.02
383.02
791.57
764.57
734.58
677.57
m/z

O

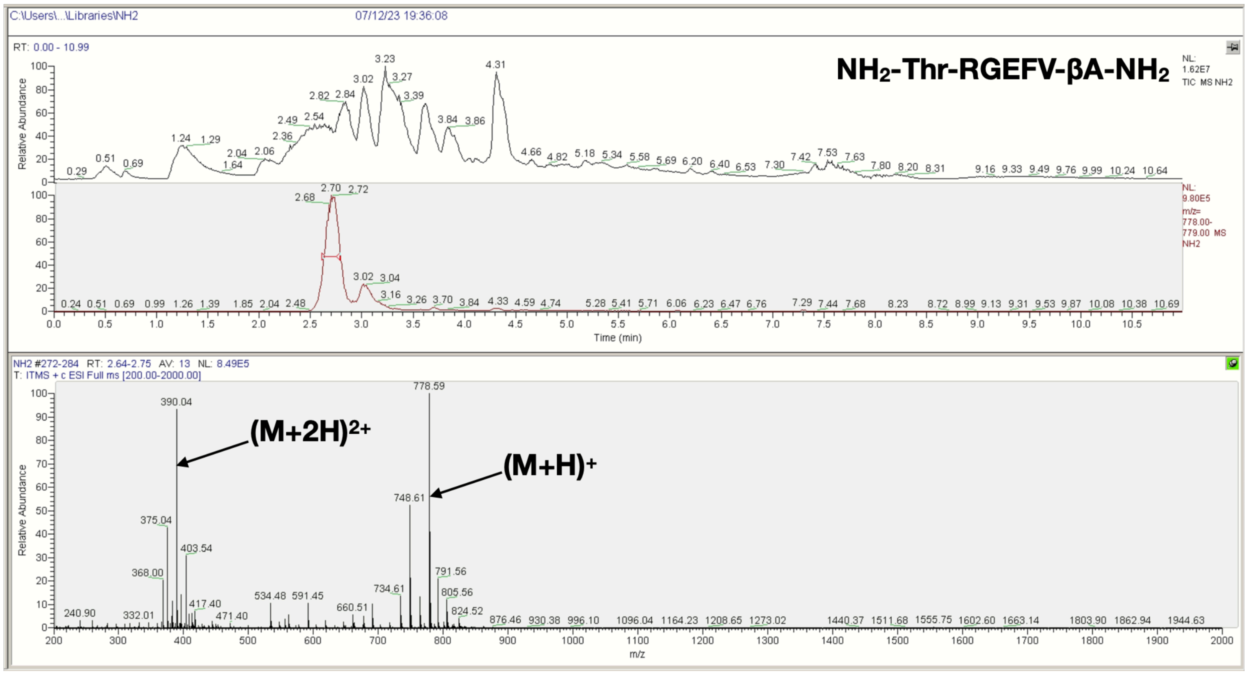

P

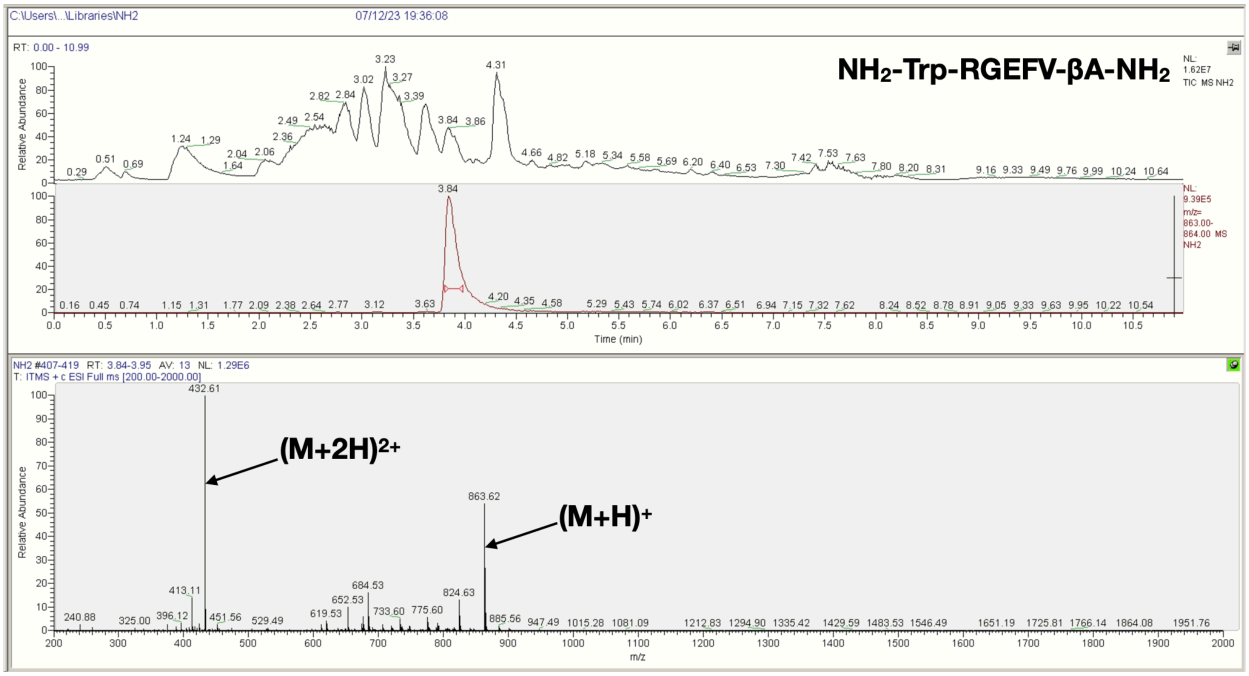

Q

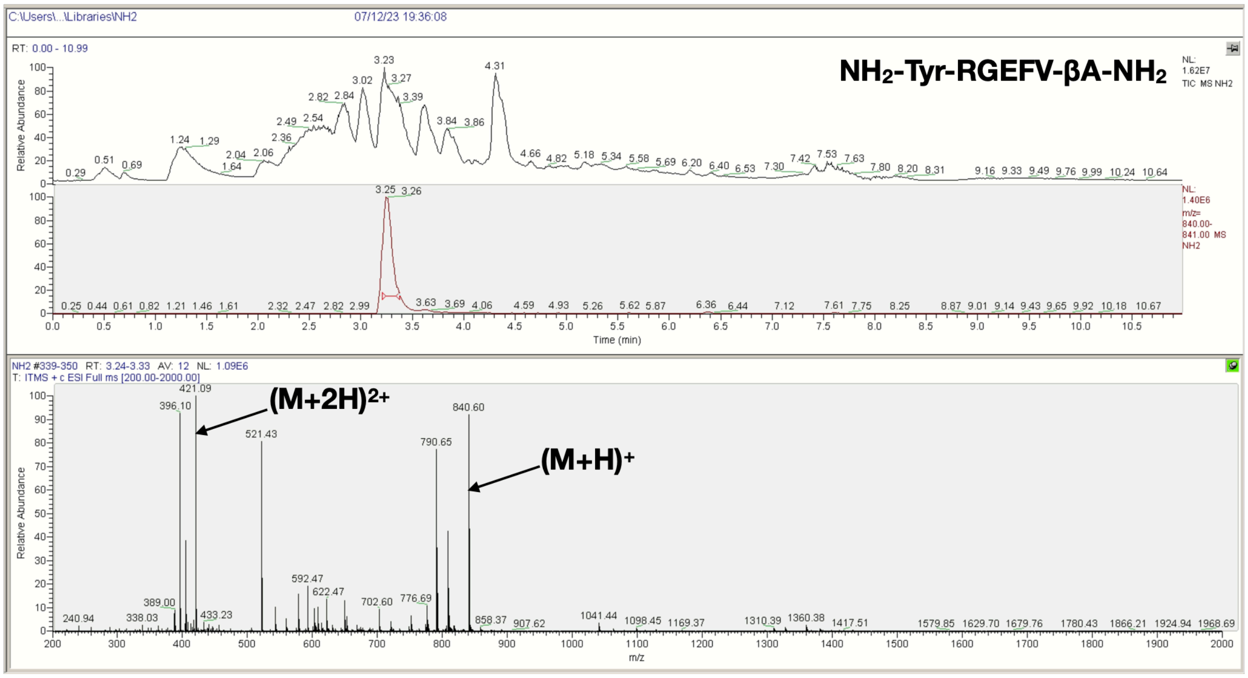

R

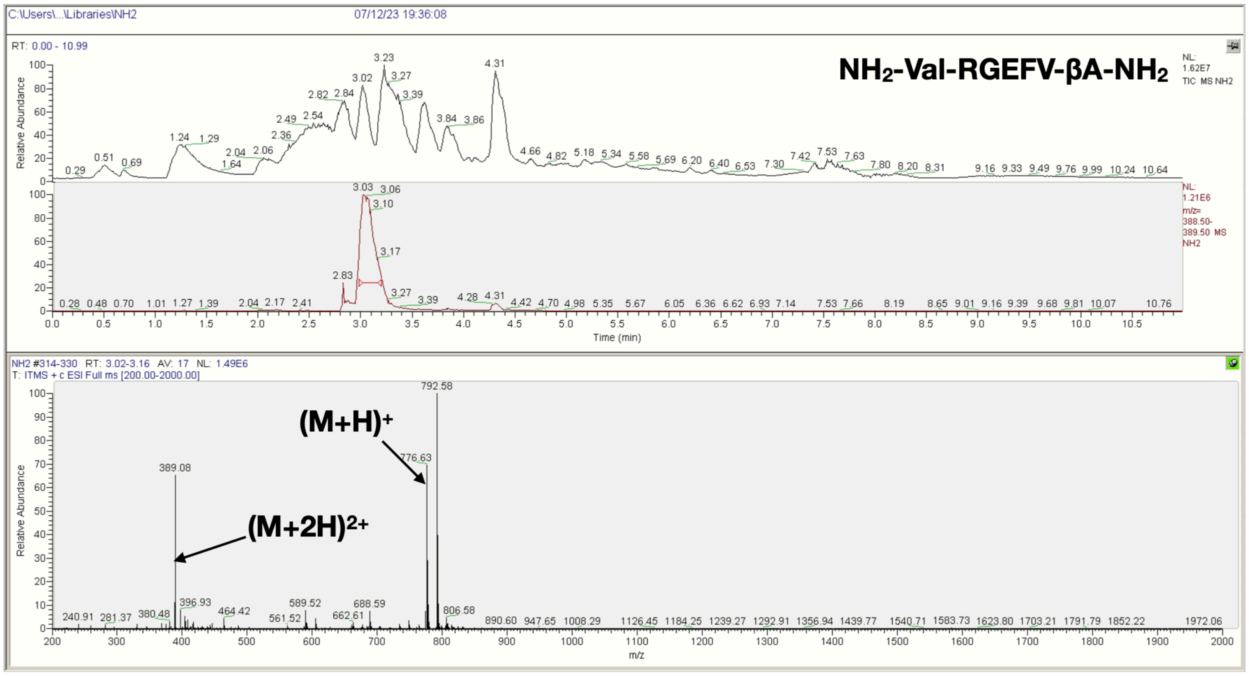

Figure S18. LCMS spectra of the NH_2-X-RGEFV-βA-NH_2 libraries, where X = a) Ala, b) Arg, c) Asn, d) Asp, e) Gln, f) Glu, g) Gly, h) His, i) Ile/Leu, j) Lys, k) Met, l) Phe, m) Pro, n) Ser, o) Thr, p) Trp, q) Tyr, r) Val.

A Ac-βA-Gly-RGEFV-βA-Am

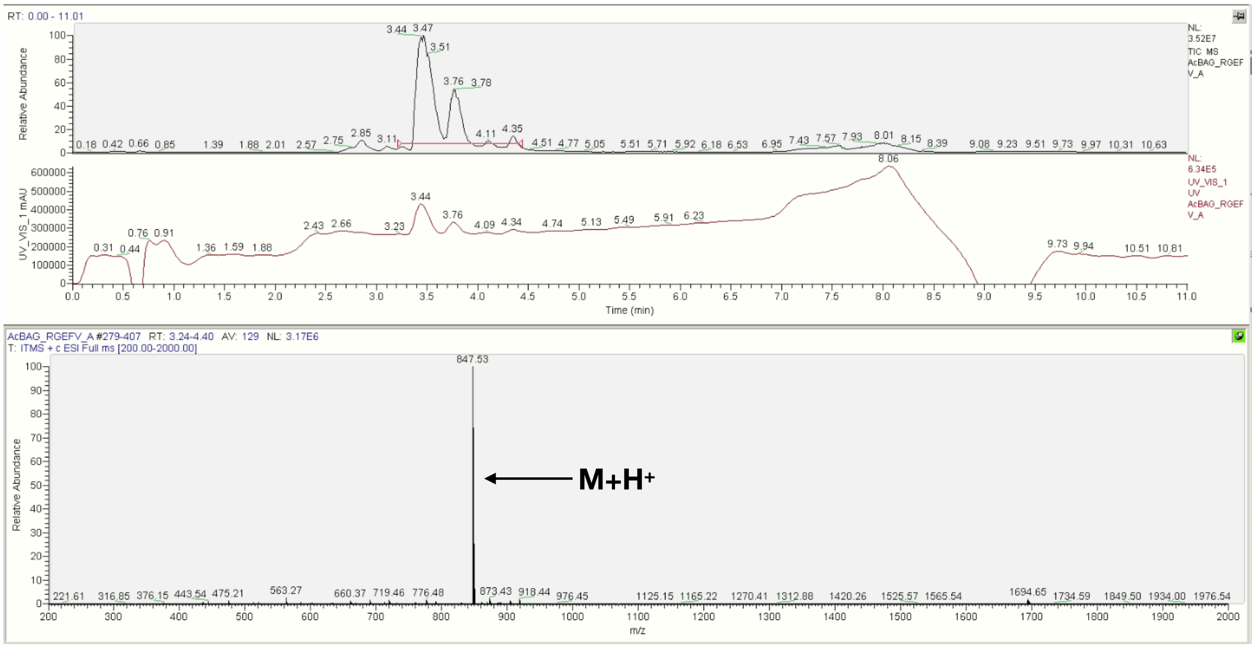

B Ac-Gly-RGEFV-βA-Am

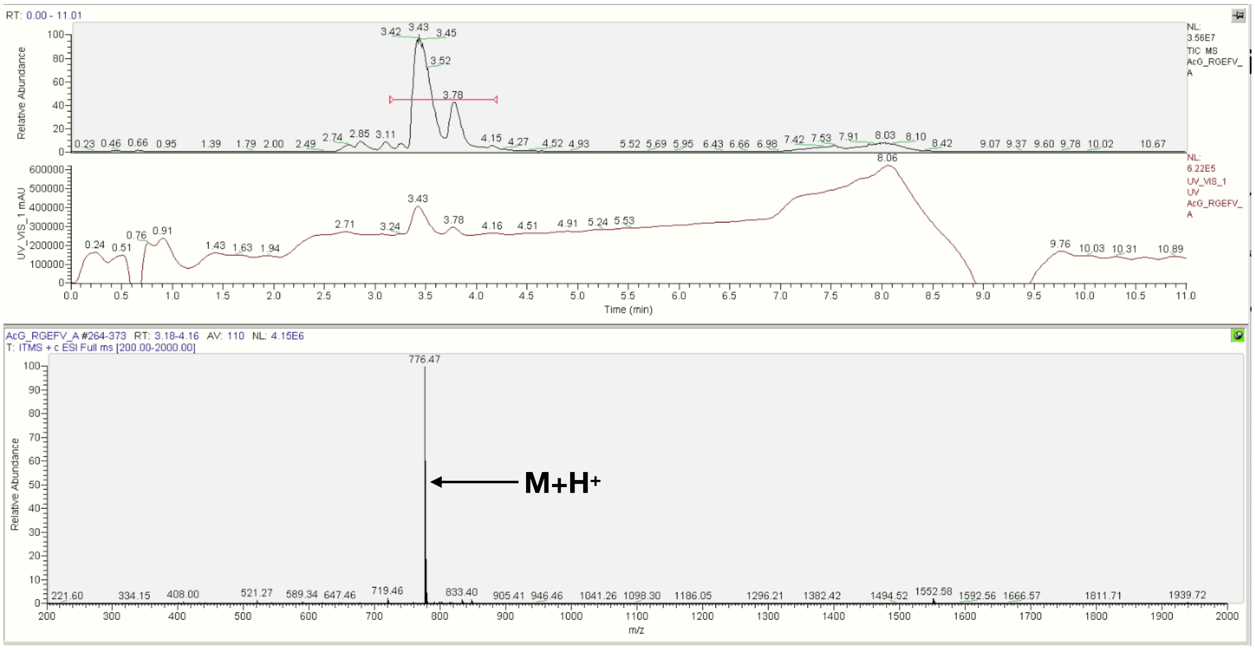

C NH2-βA-Gly-RGEFV-βA-Am

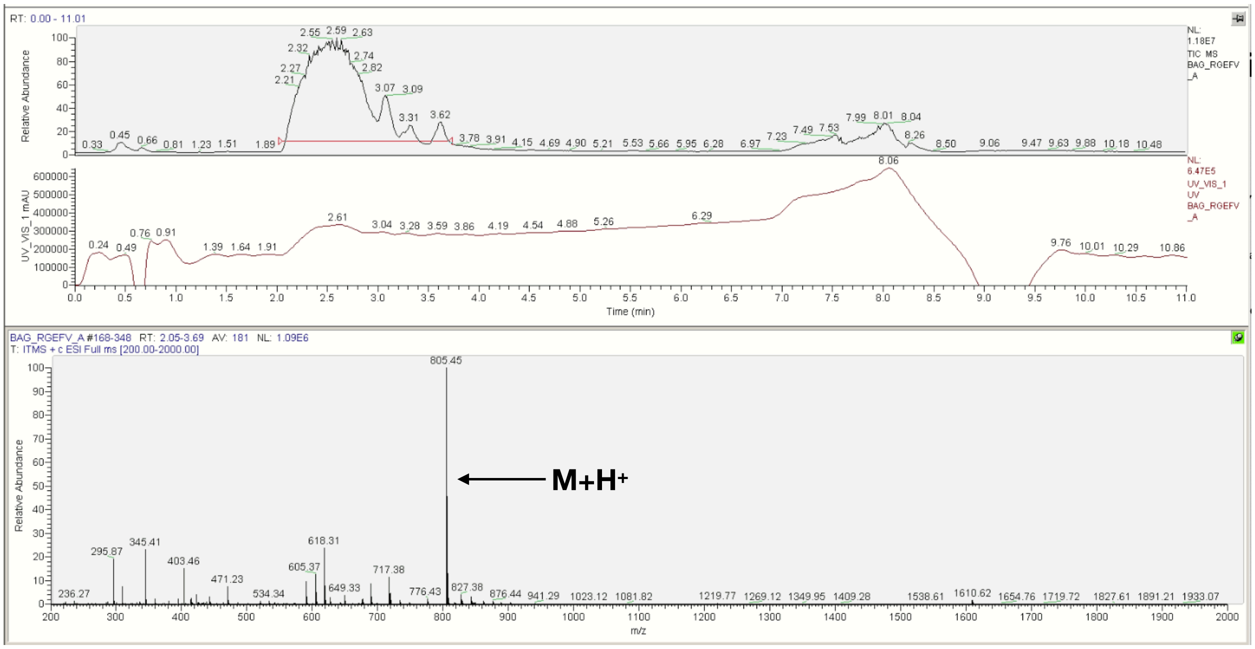

D NH2-Gly-RGEFV-βA-Am

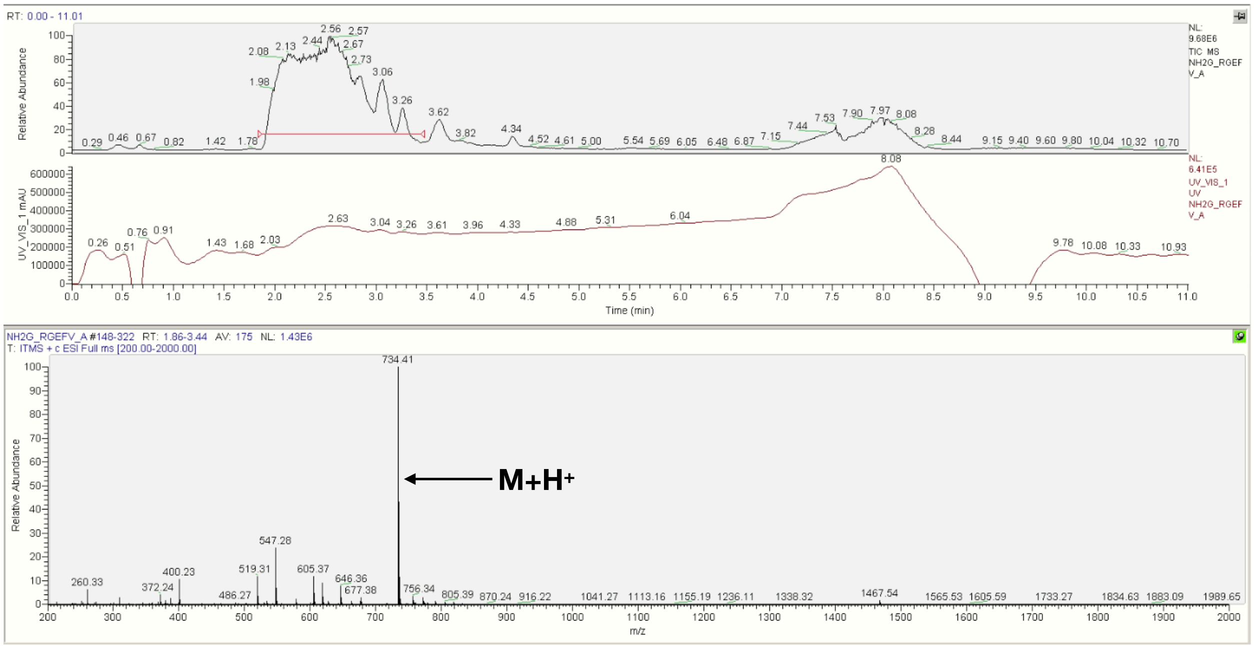

E Ac-βA-RGEFV-Gly-βA-Am

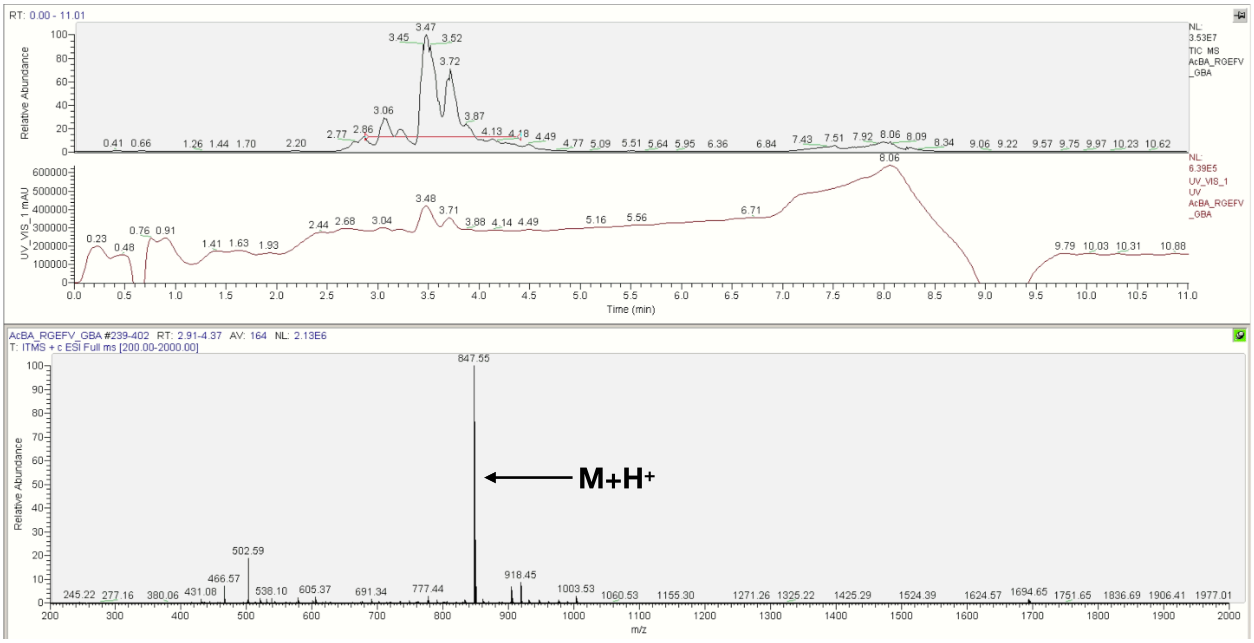

F Ac-βA-RGEFV-Gly-Am

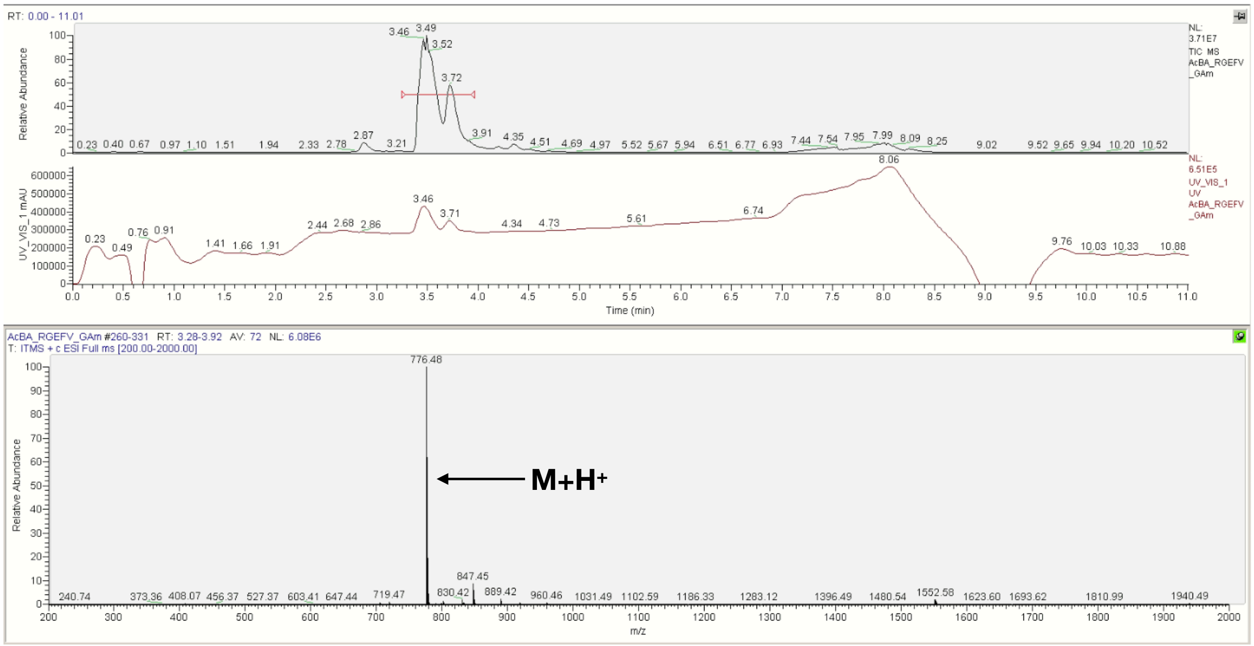

G Ac-βA-RGEFV-Gly-COOH

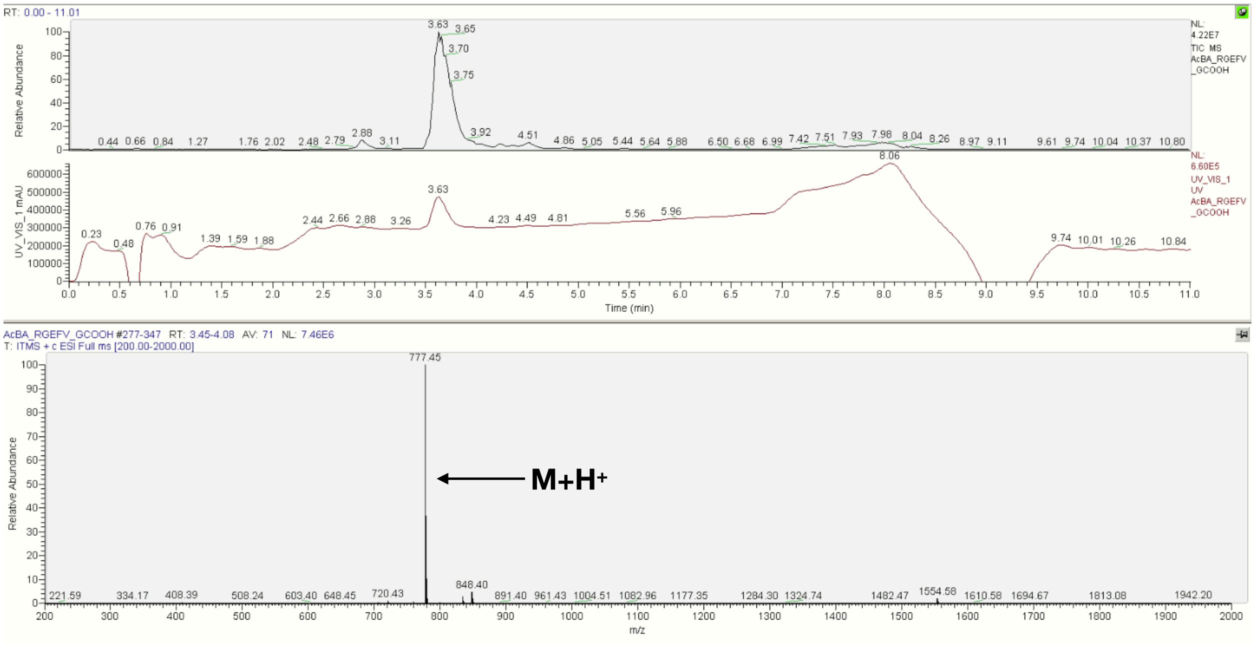

Figure S19. LCMS spectra of the peptides used for the concentration studies in which a glycine was placed on the N-terminus for N-terminal libraries, and the C-terminus for C-terminal libraries. a) Ac-βA, b) Ac, c) N-βA, d) NH_2, e) C-βA, f) Am, g) COOH.

A Ac-βA-Gly-LIAANK-βA-Am

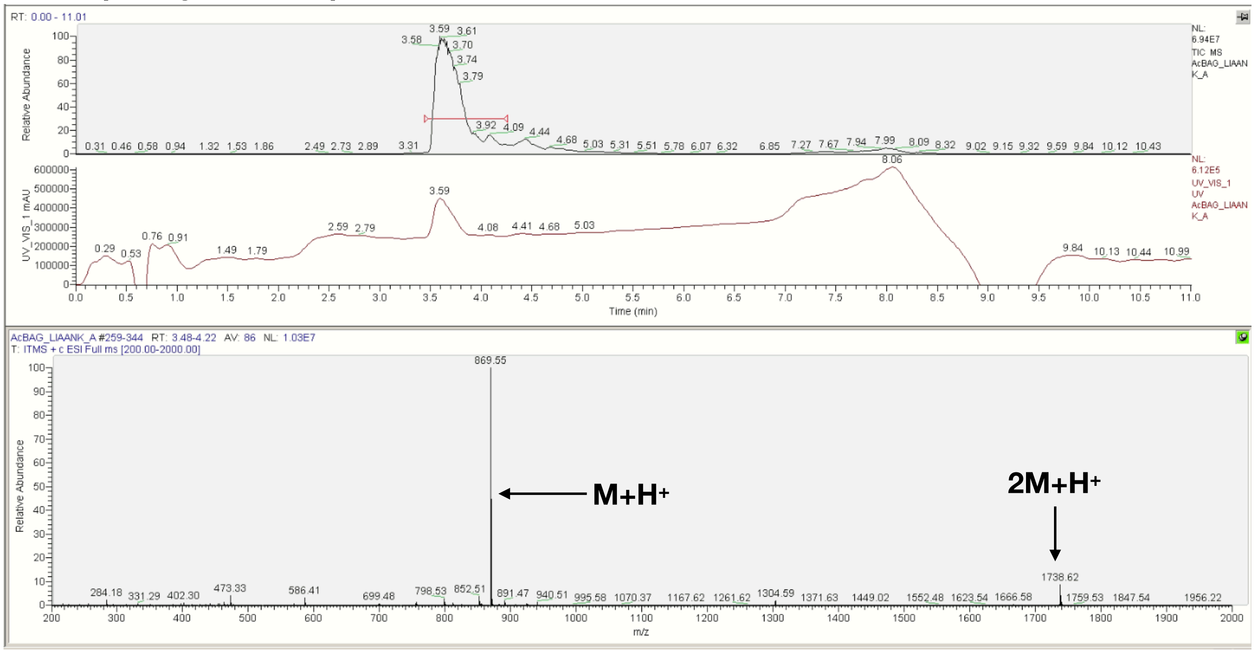

B Ac-Gly-LIAANK-βA-Am

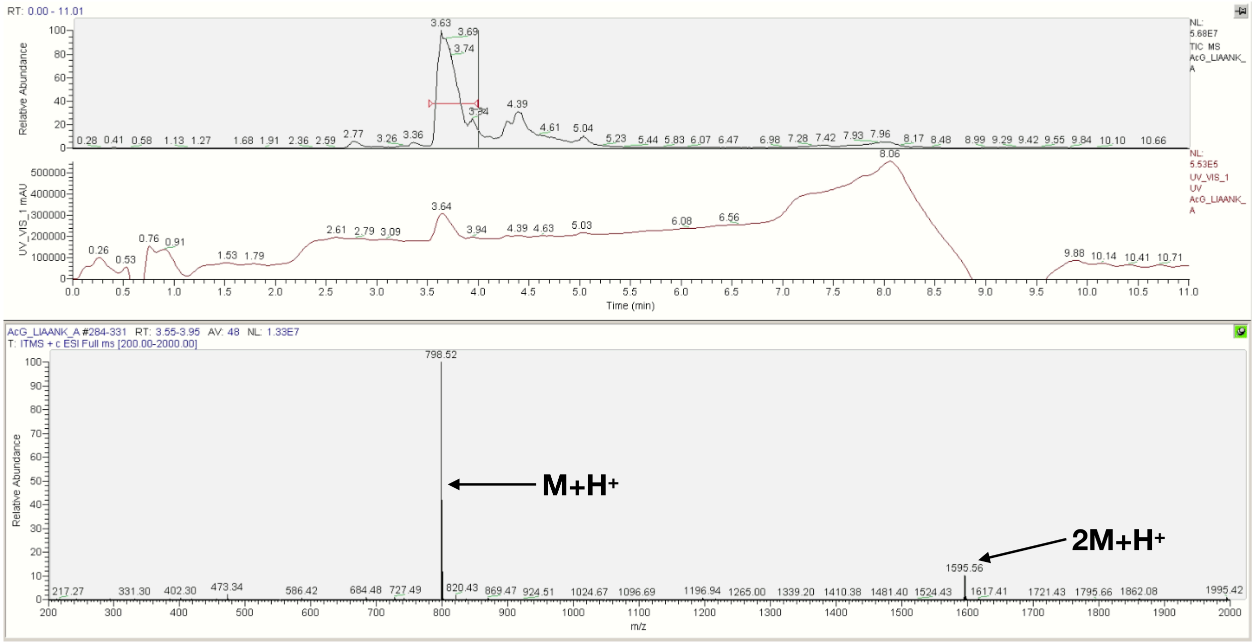

C NH_2-βA-Gly-LIAANK-βA-Am

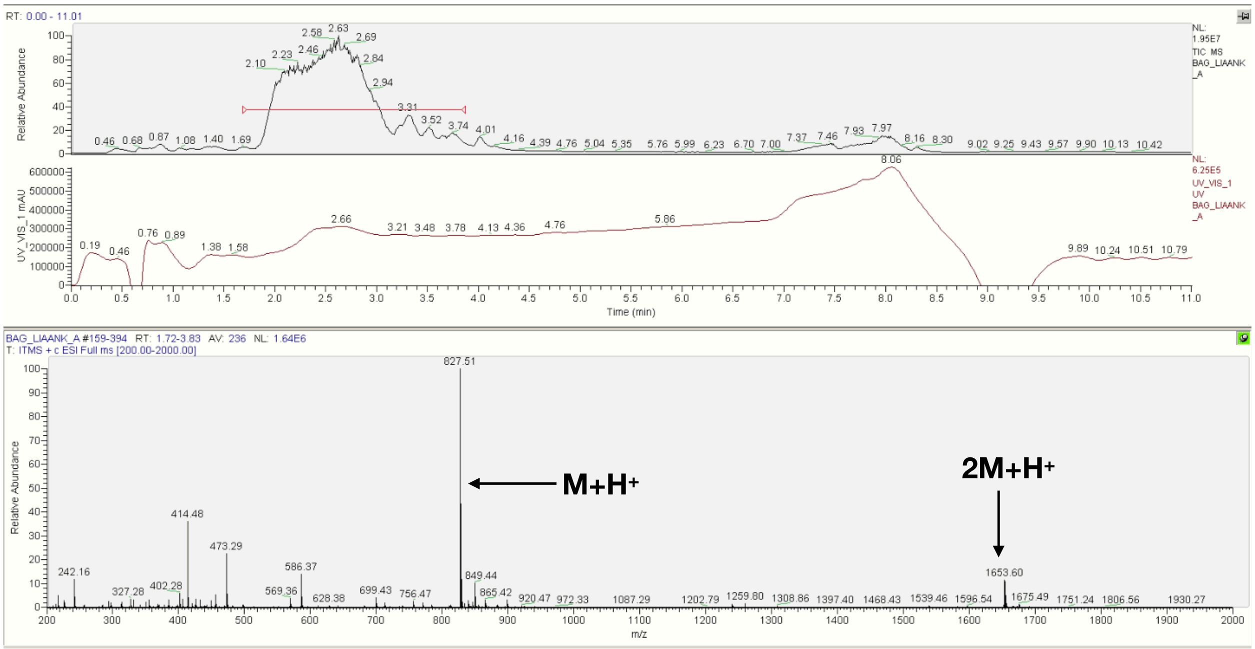

D NH$_2$-Gly-LIAANK-βA-Am

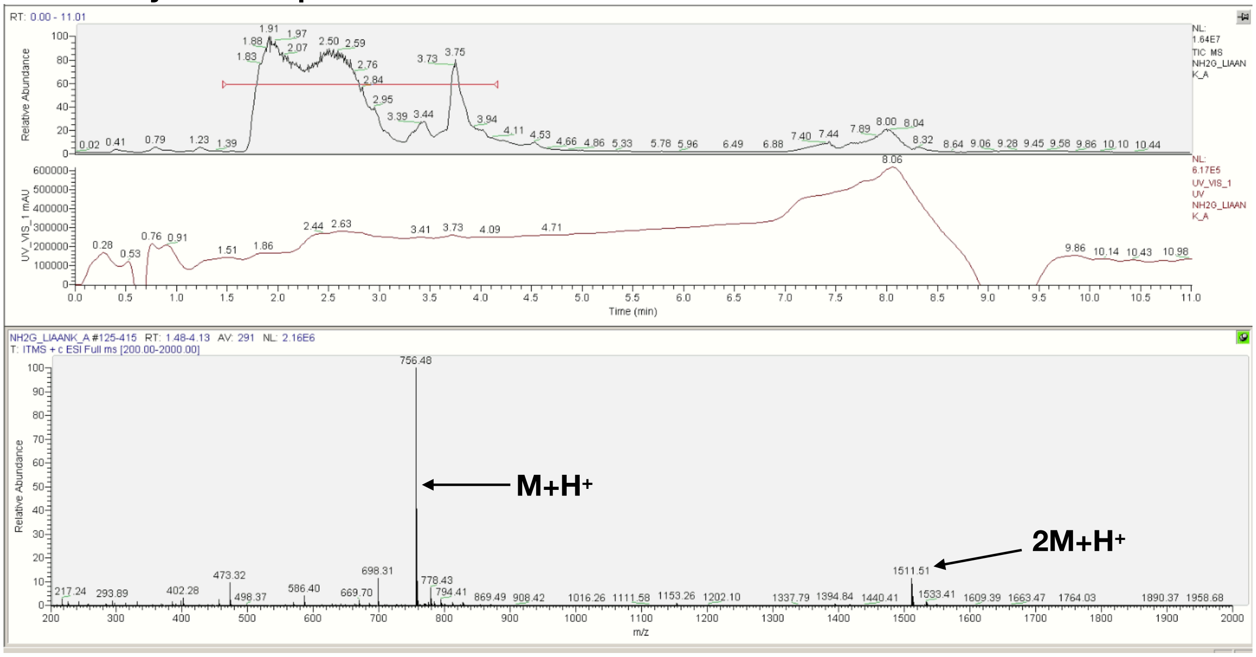

E Ac-βA-LIAANK-Gly-βA-Am

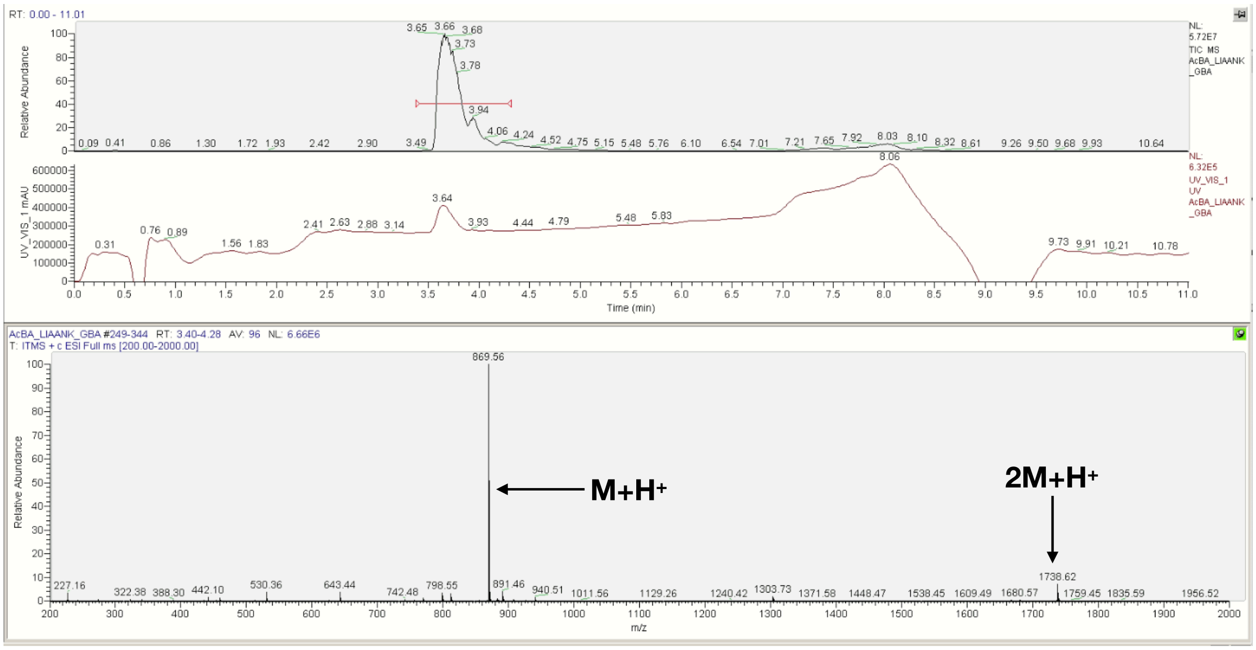

F Ac-βA-LIAANK-Gly-Am

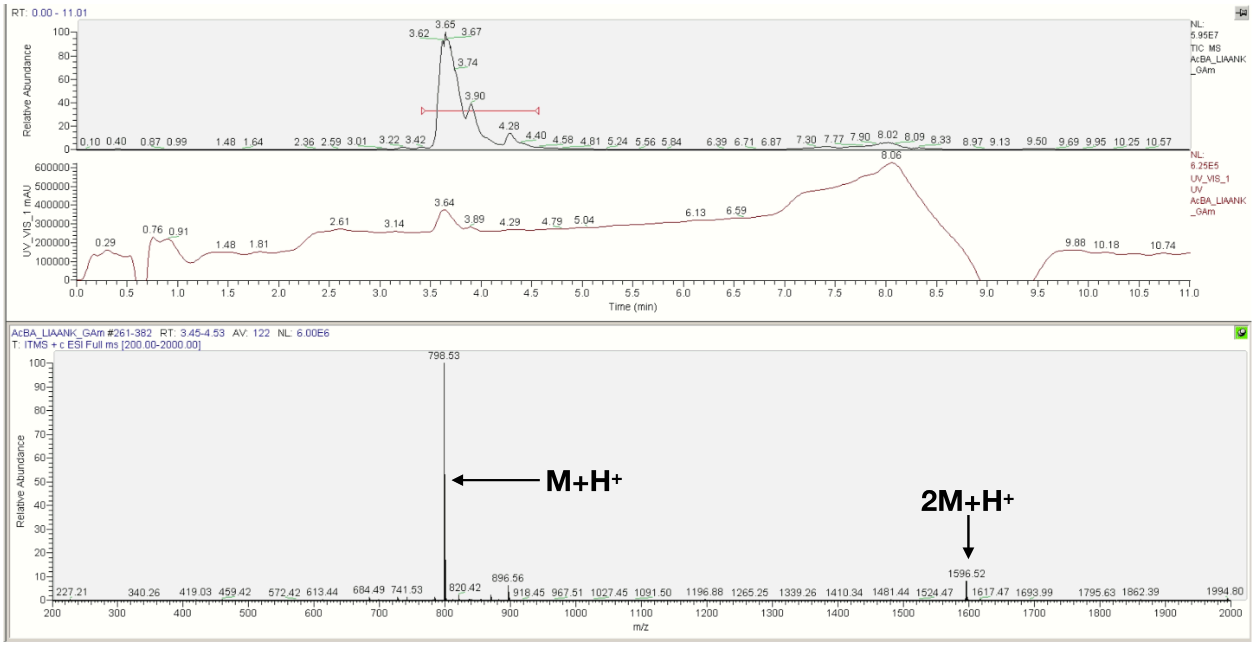

G Ac-βA-LIAANK-Gly-COOH

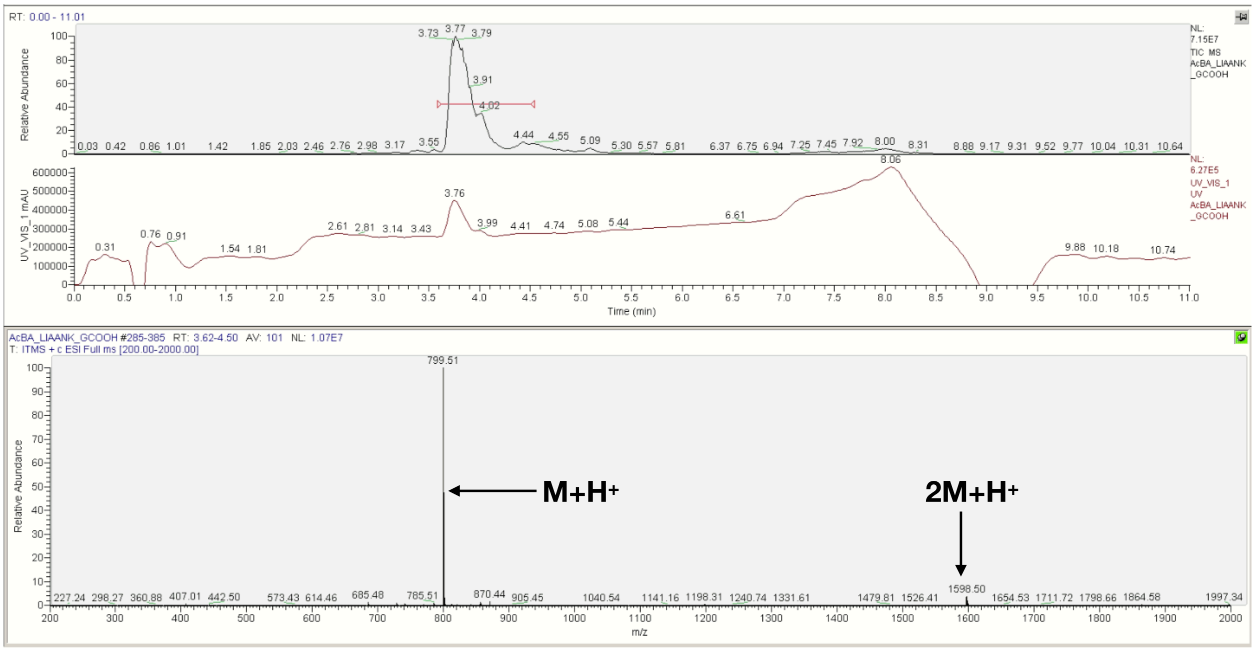

Figure S20. LCMS spectra of the LIAANK peptides in which a glycine was placed on the N-terminus for N-terminal libraries, and the C-terminus for C-terminal libraries. a) Ac-βA, b) Ac, c) N-βA, d) NH_2, e) C-βA, f) Am, g) COOH.

A Ac-βA-Gly-IVKVA-βA-Am

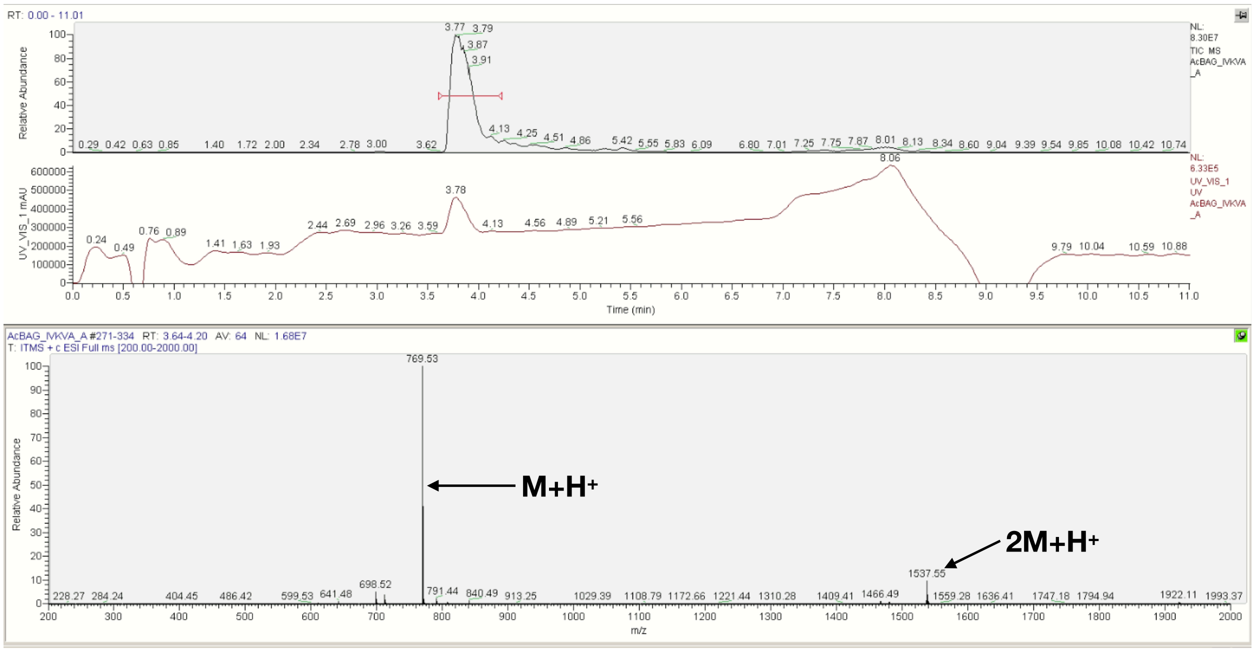

B Ac-Gly-IVKVA-βA-Am

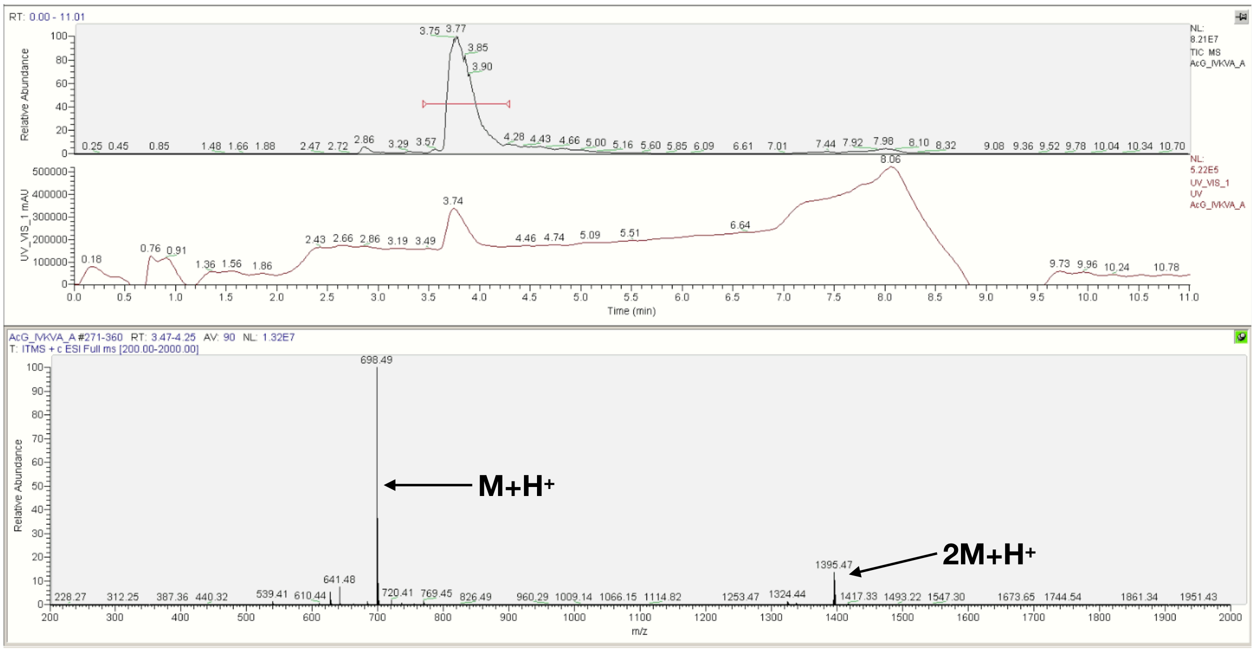

C NH$_2$-βA-Gly-IVKVA-βA-Am

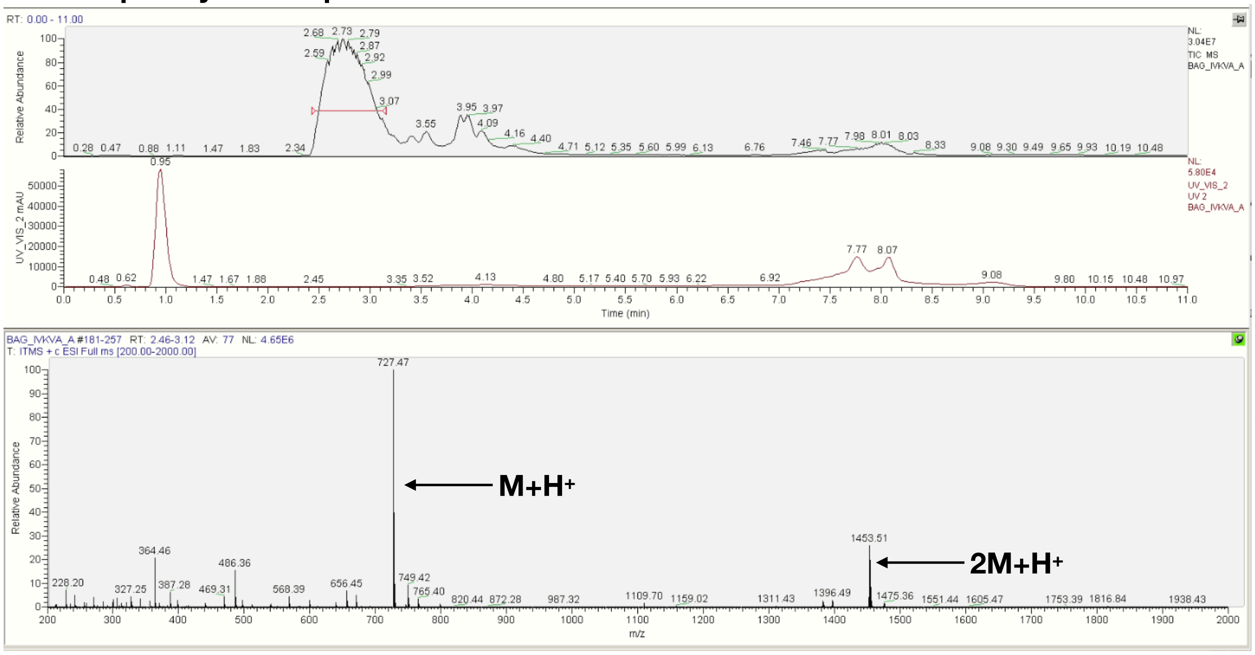

D NH$_2$-Gly-IVKVA-βA-Am

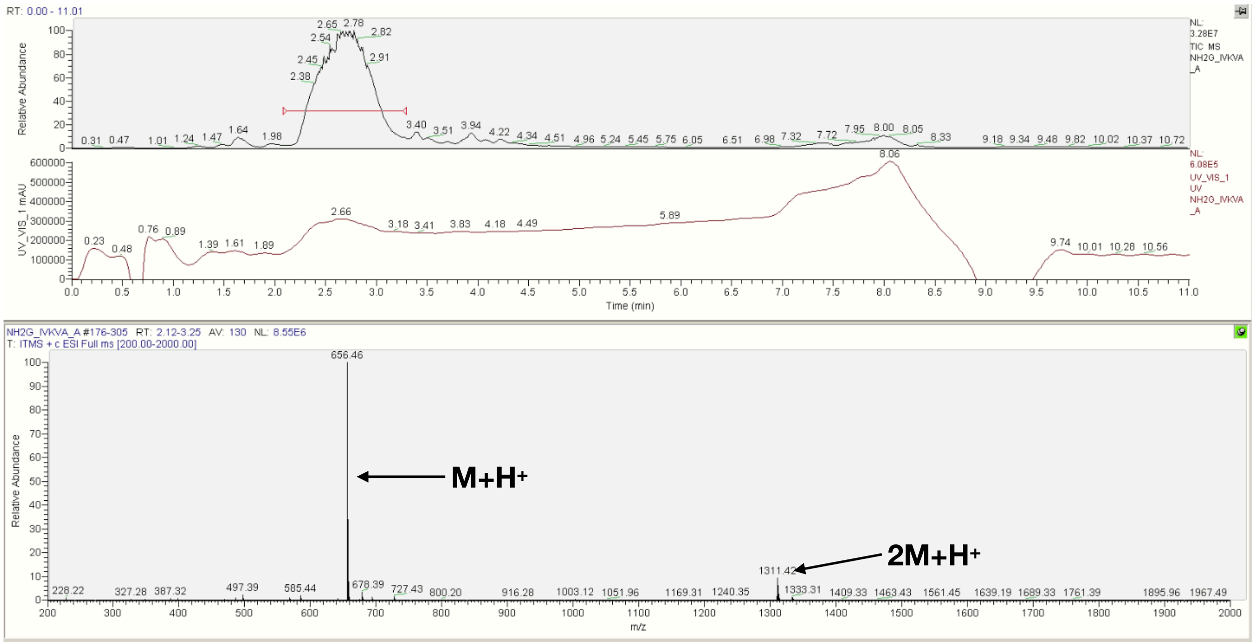

E Ac-βA-IVKVA-Gly-βA-Am

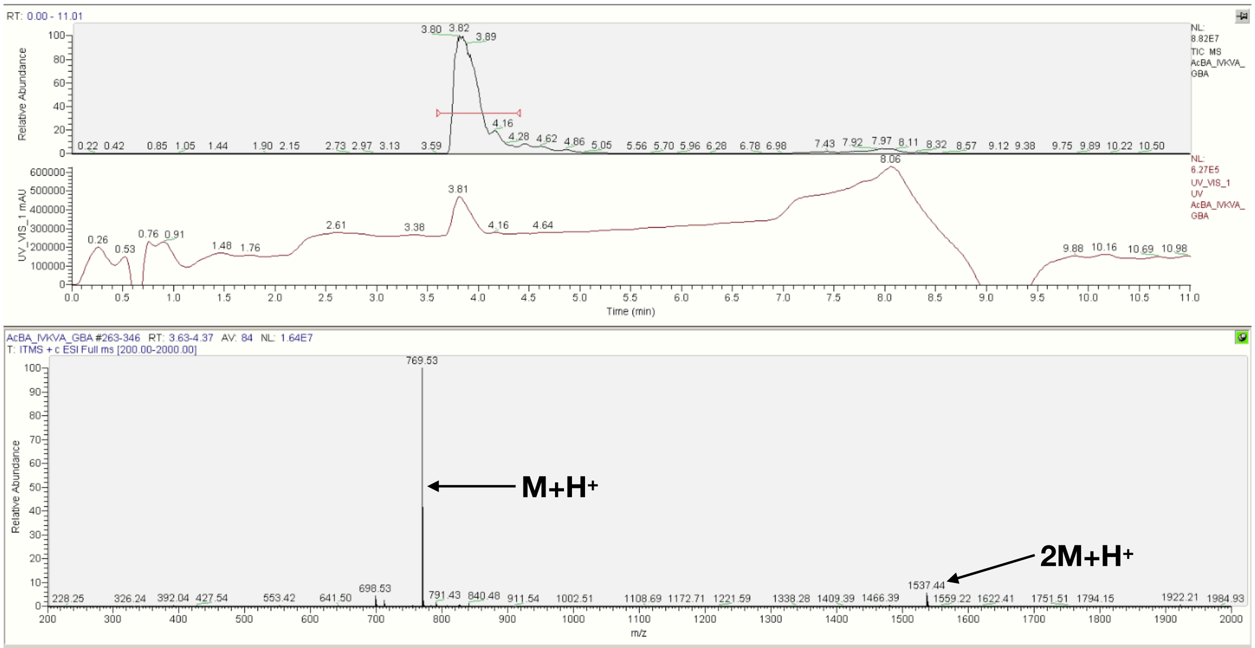

F Ac-βA-IVKVA-Gly-Am

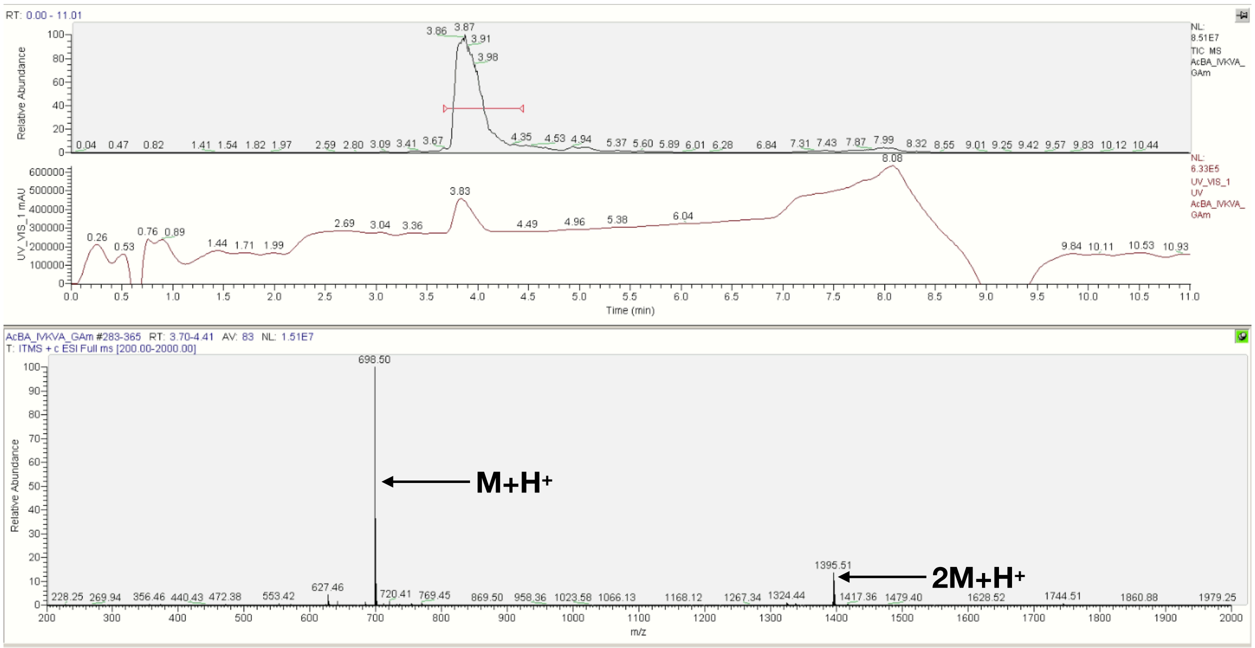

G Ac-βA-IVKVA-Gly-COOH

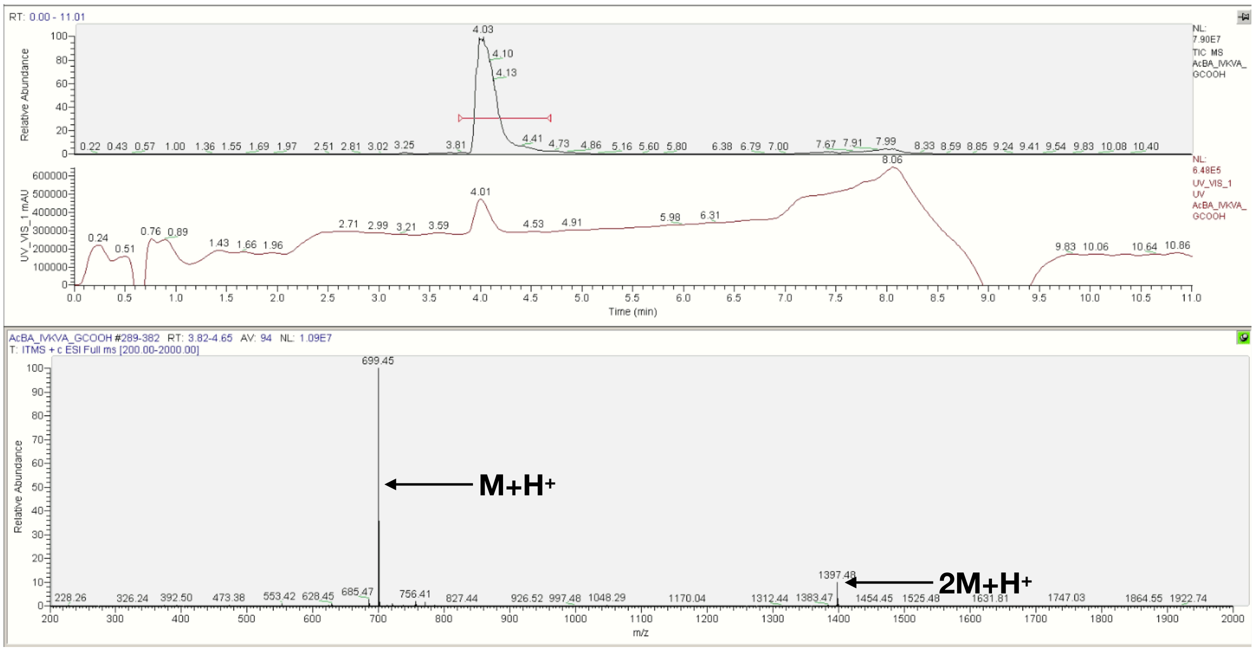

Figure S21. LCMS spectra of the IVKVA peptides in which a glycine was placed on the N-terminus for N-terminal libraries, and the C-terminus for C-terminal libraries. a) Ac-βA, b) Ac, c) N-βA, d) NH_2, e) C-βA, f) Am, g) COOH.

A

<u>NH_2-Lys(N_3)-RGEFV-βA-Am</u>

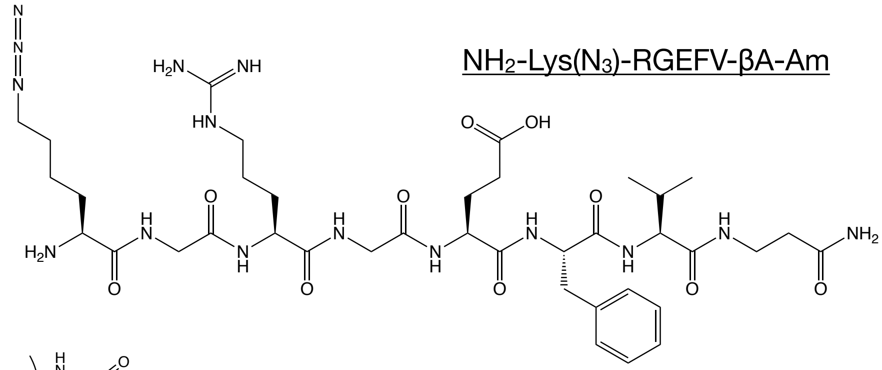

<u>NH_2-Lys(PEG)-RGEFV-βA-Am</u>

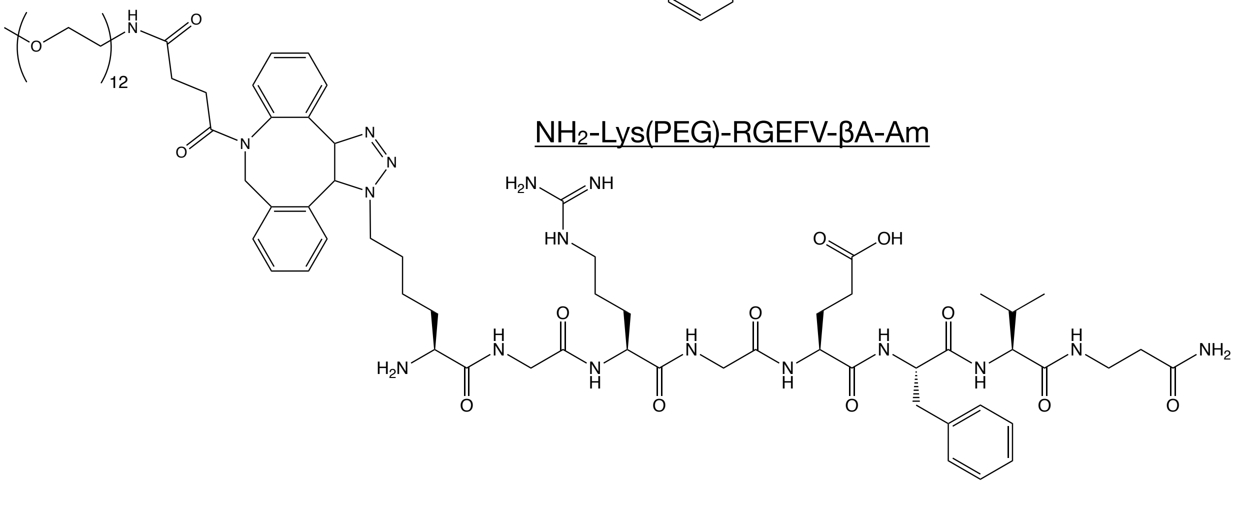

B Ac-βA-Lys(N_3)-RGEFV-βA-Am

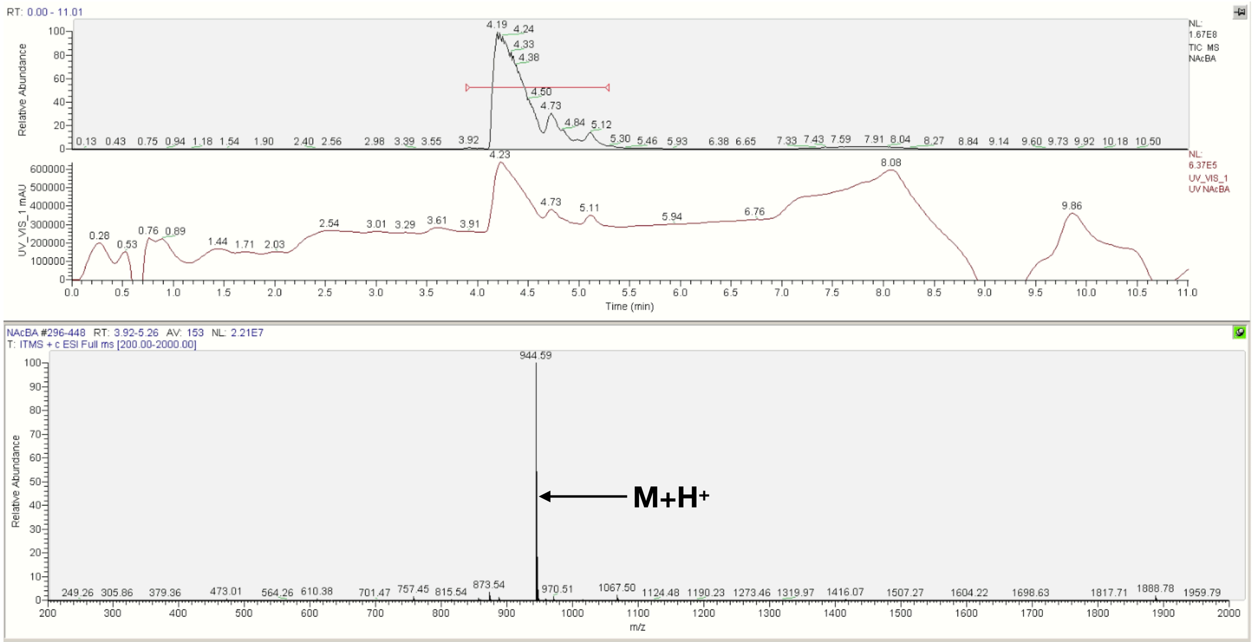

C Ac-Lys(N_3)-RGEFV-βA-Am

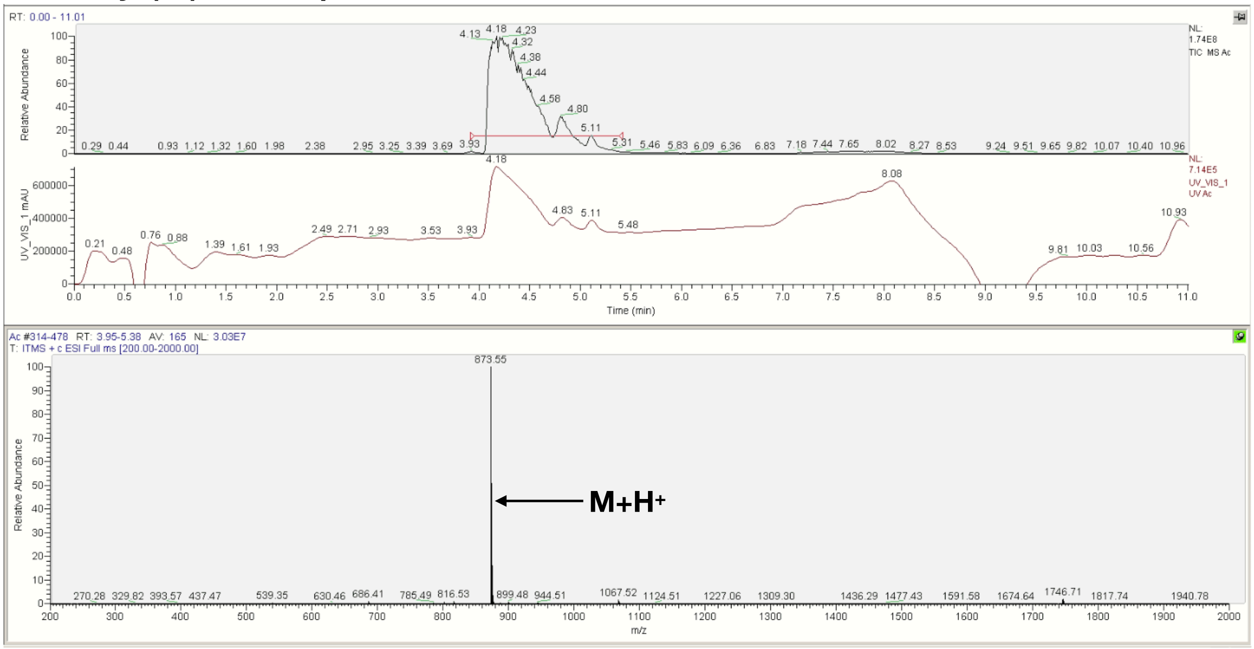

D NH$_2$-βA-Lys(N$_3$)-RGEFV-βA-Am

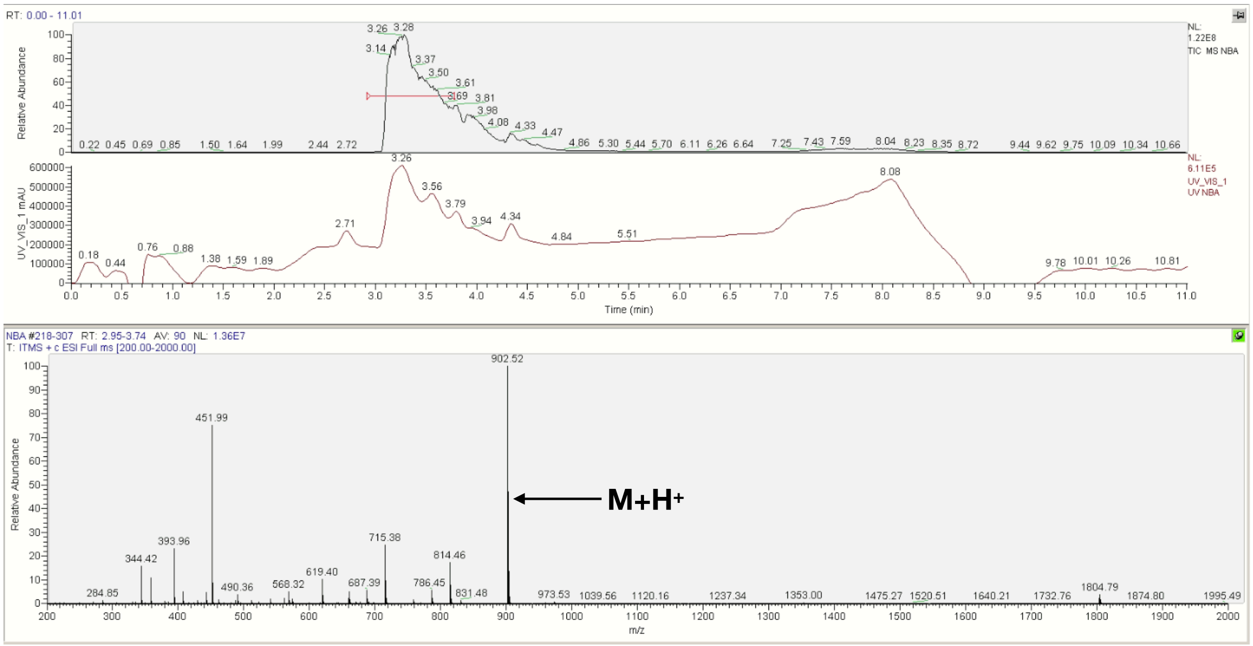

E NH$_2$-Lys(N$_3$)-RGEFV-βA-Am

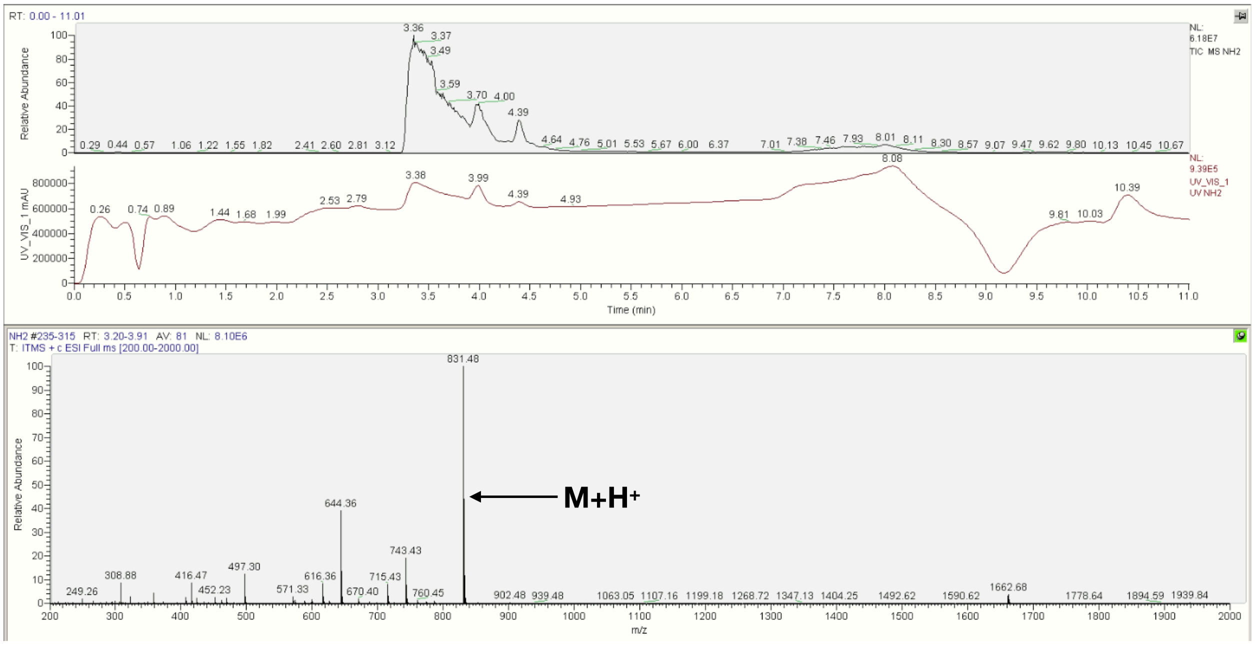

F Ac-βA-RGEFV-Lys(N_3)-βA-Am

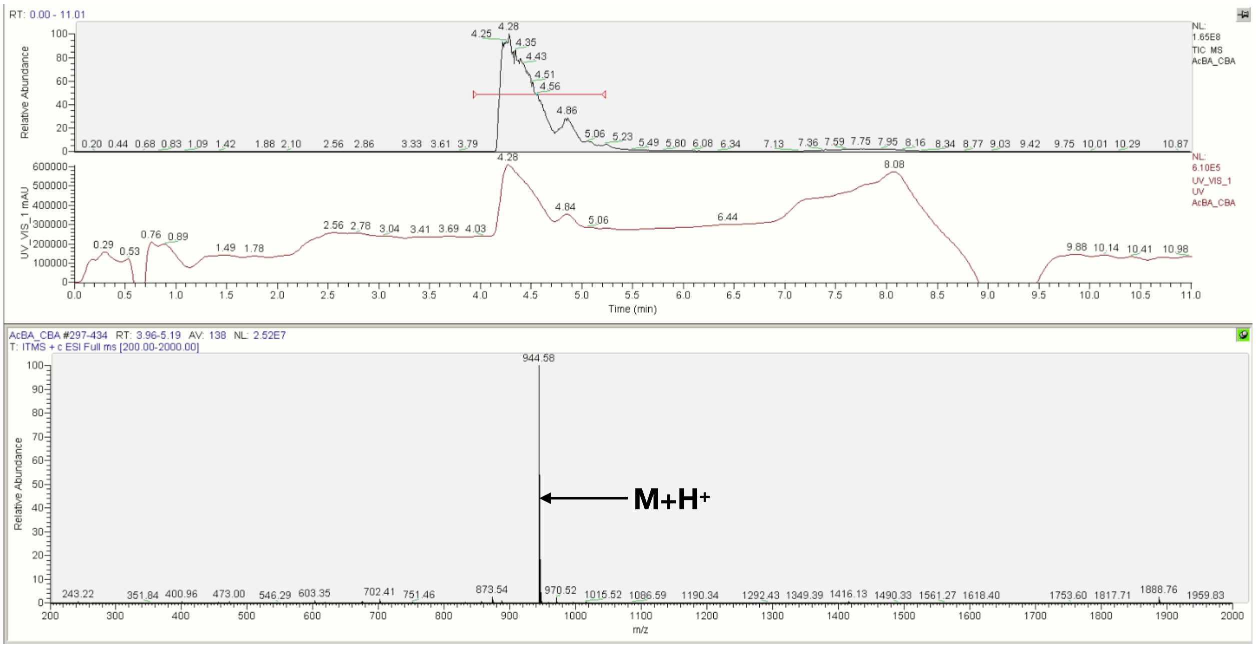

G Ac-βA-RGEFV-Lys(N_3)-Am

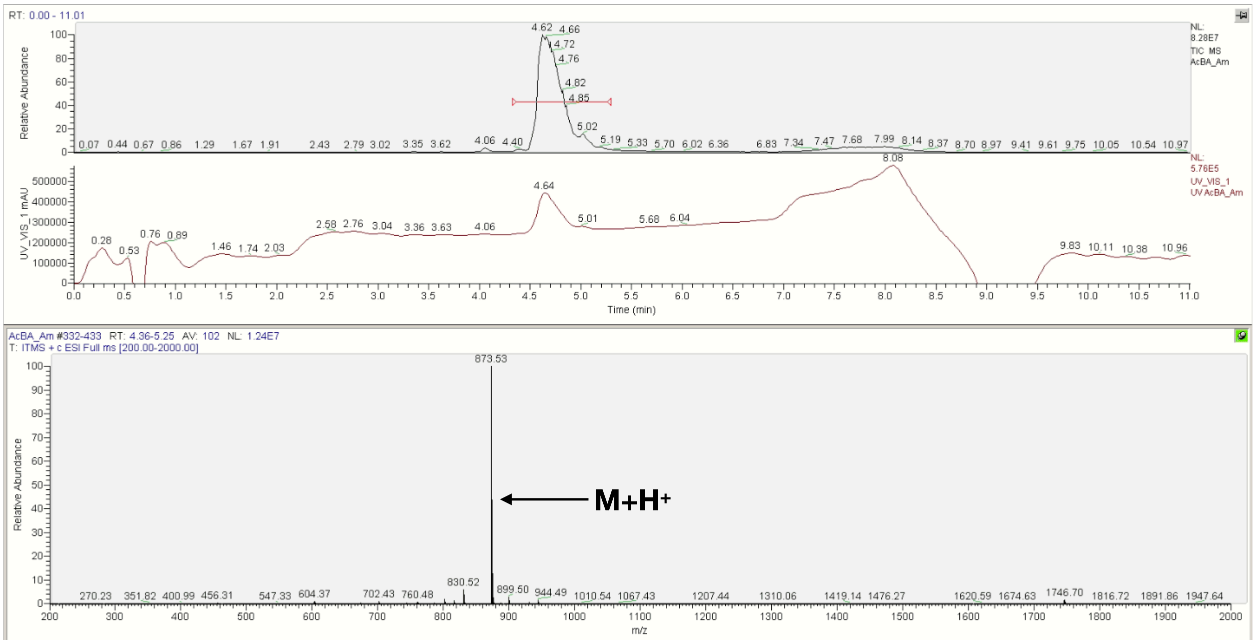

H Ac-βA-RGEFV-Lys(N_3)-COOH

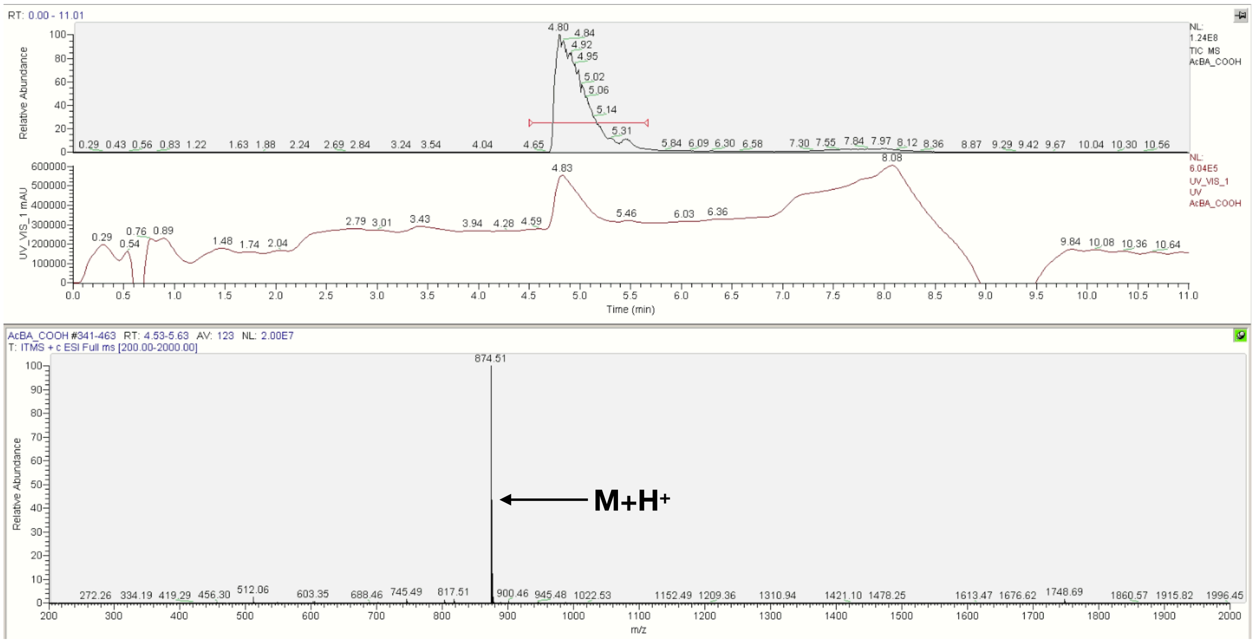

I Ac-βA-Lys(PEG)-RGEFV-βA-Am

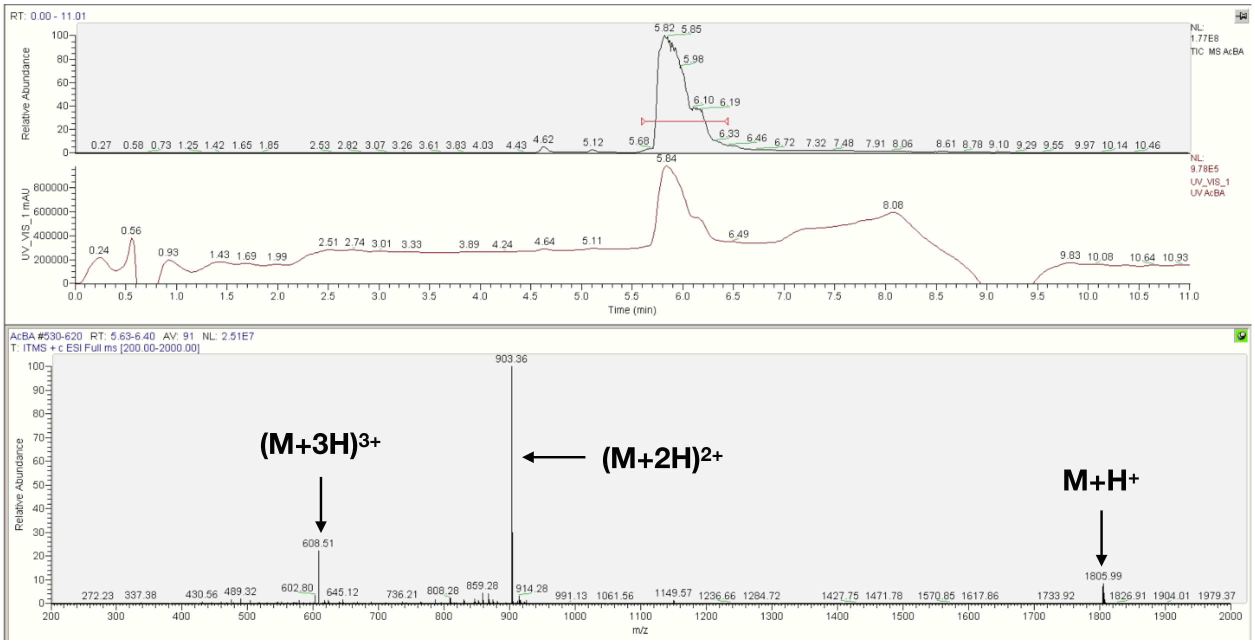

J Ac-Lys(PEG)-RGEFV-βA-Am

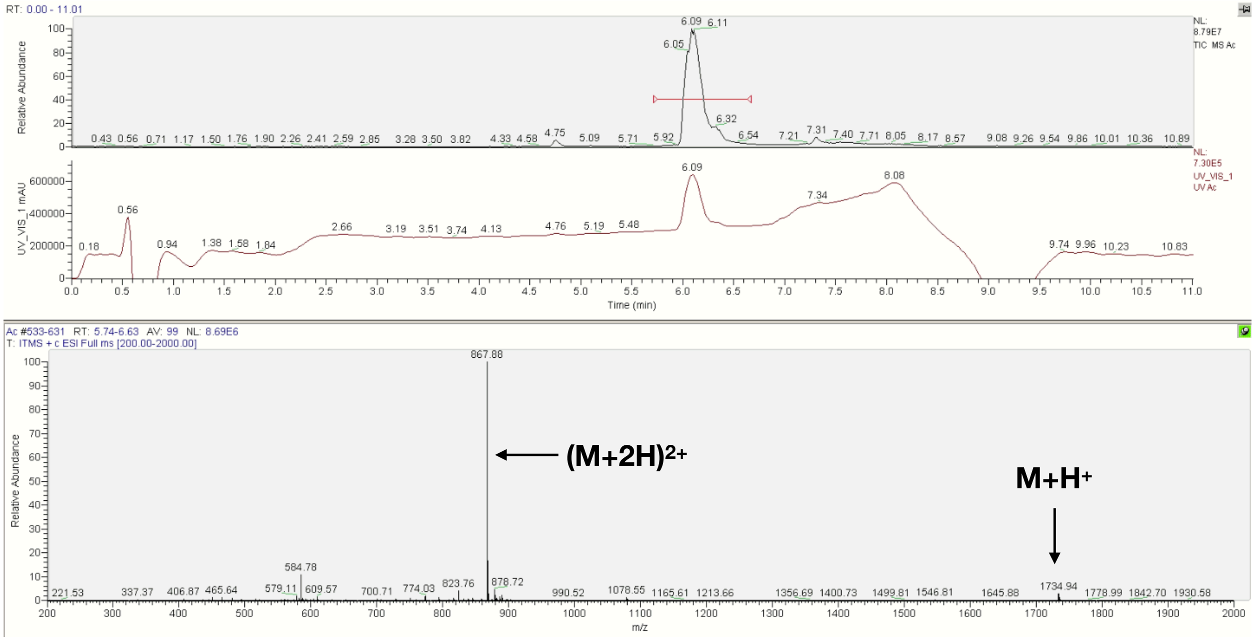

K NH_2-βA-Lys(PEG)-RGEFV-βA-Am

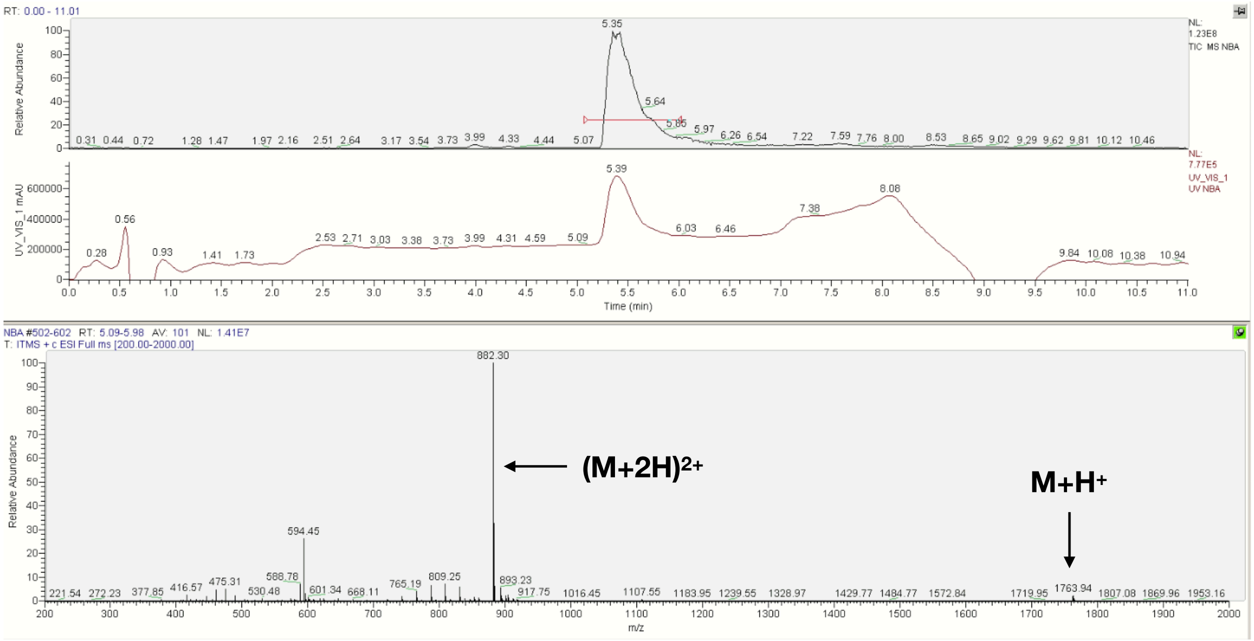

L NH$_2$-Lys(PEG)-RGEFV-βA-Am

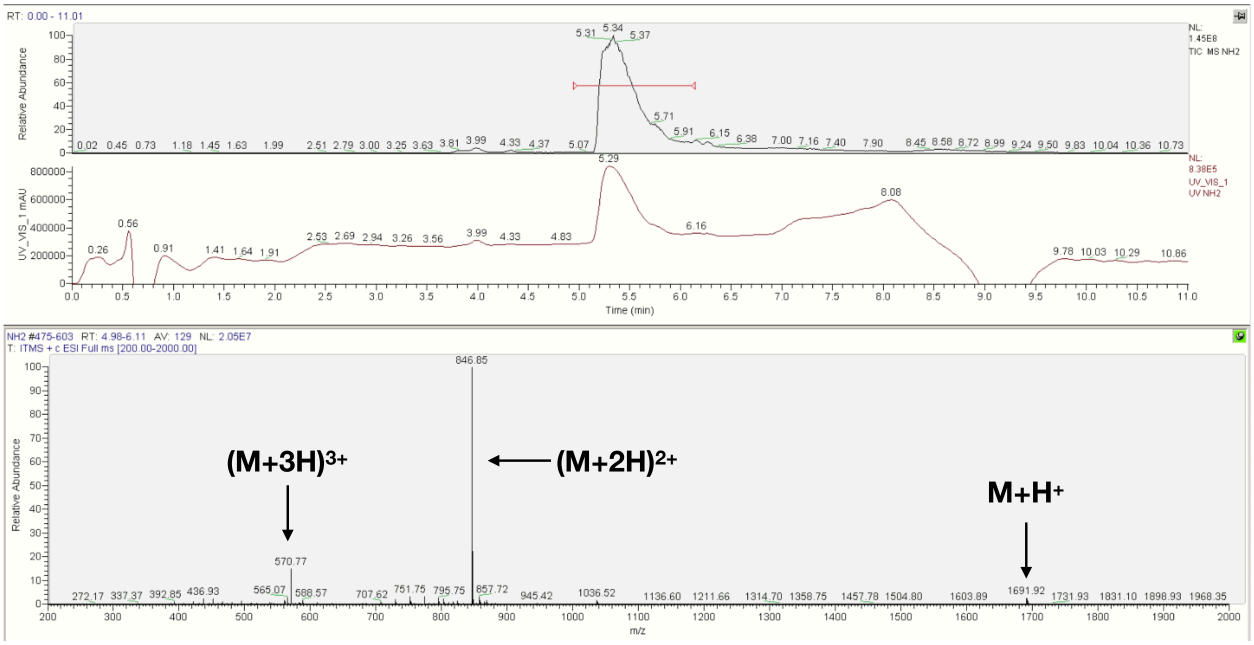

M Ac-βA-RGEFV-Lys(PEG)-βA-Am

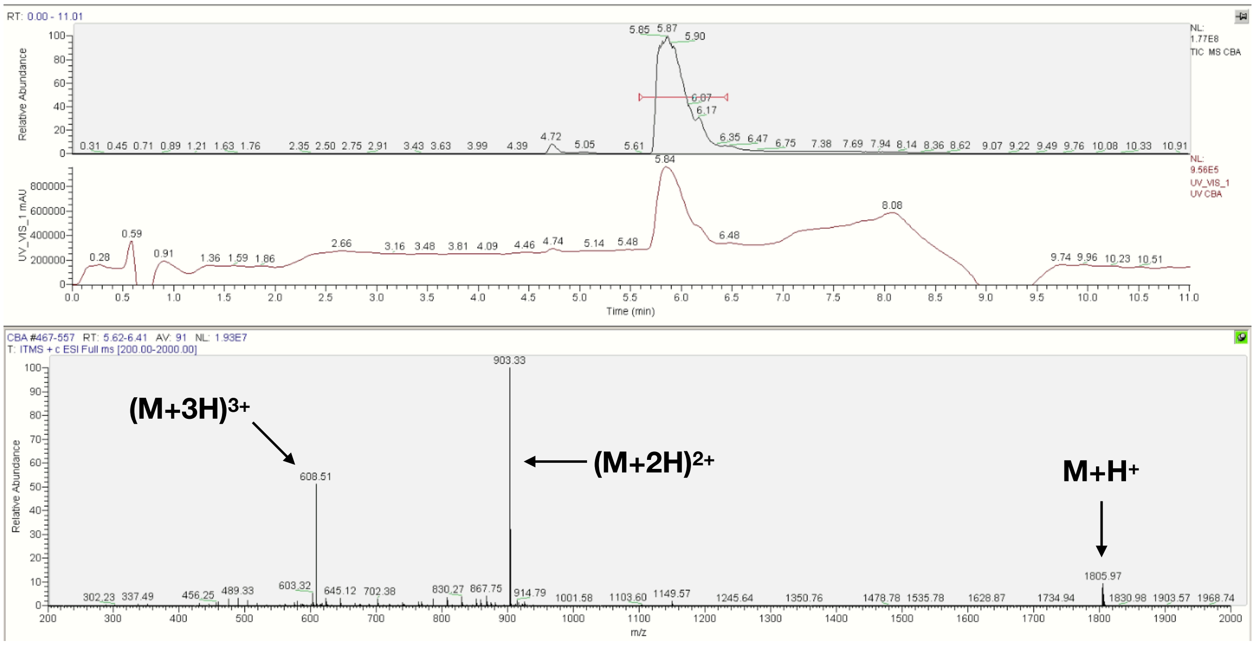

N Ac-βA-RGEFV-Lys(PEG)-Am

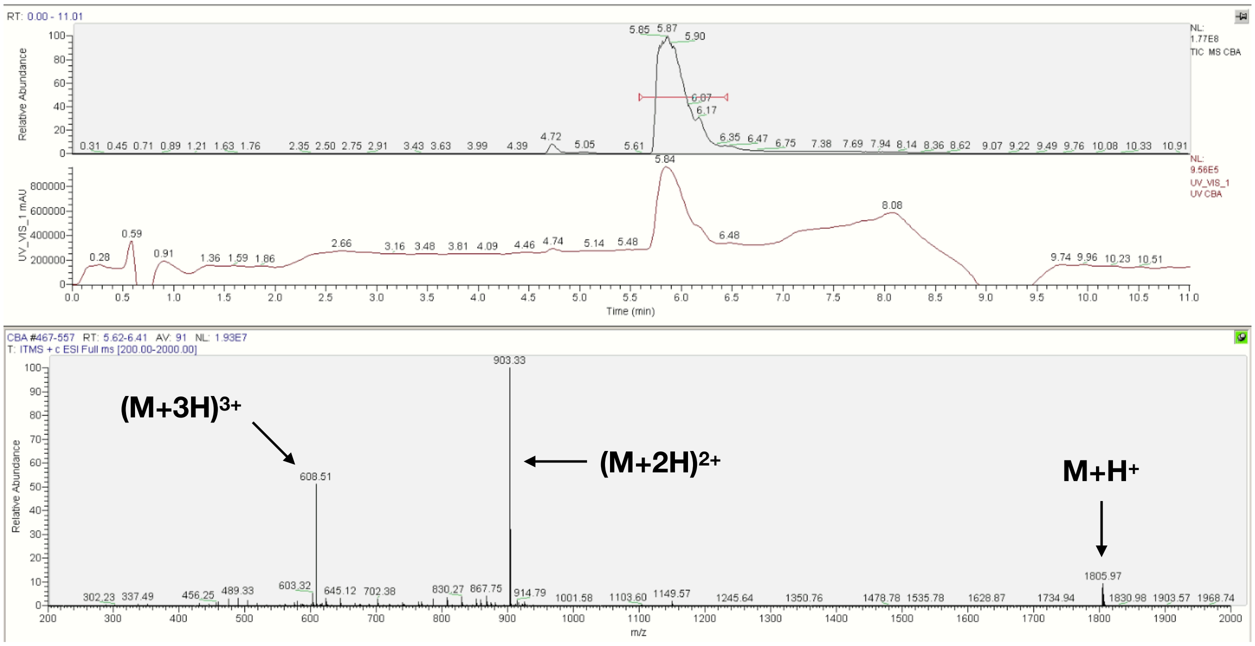

O Ac-βA-RGEFV-Lys(PEG)-COOH

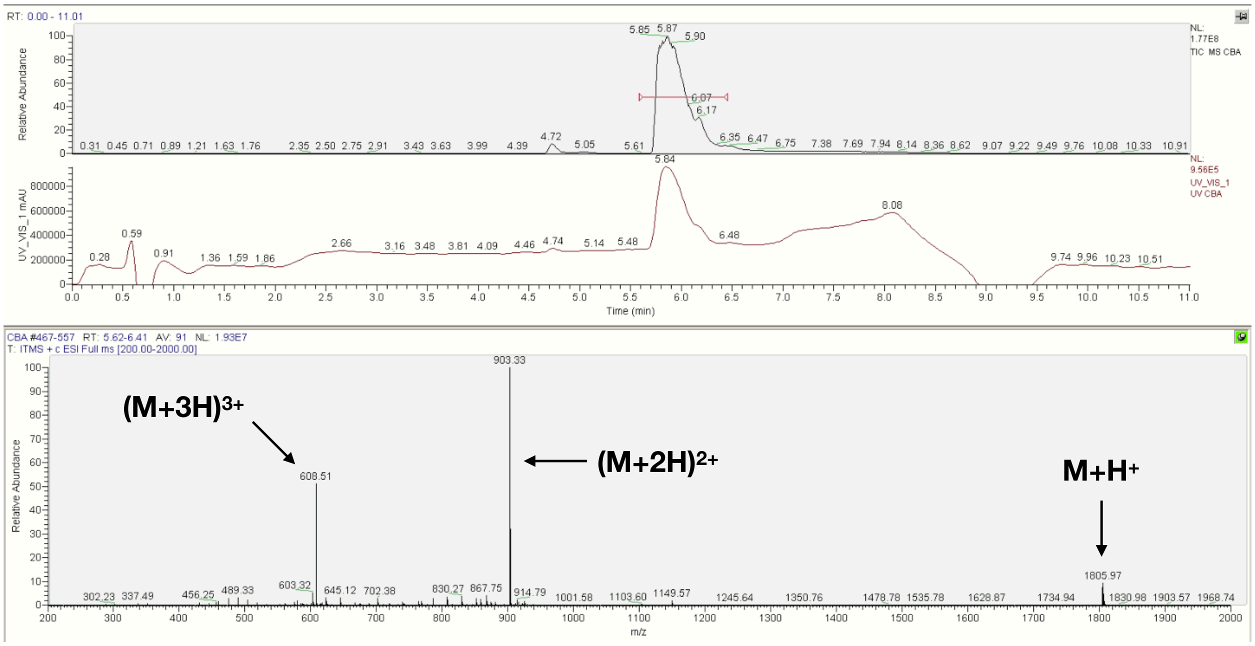

Figure S22. LCMS spectra of the Azide and PEG modified peptides in which a glycine was placed on the N-terminus for N-terminal libraries, and the C-terminus for C-terminal libraries. a) The structure of azide and PEG peptides. The azide peptides contained the following functionalizations: b) Ac-βA, c) Ac, d) N-βA, e) NH_2, f) C-βA, g) Am, h) COOH. The PEG-modified peptides contained the following functionalizations: i) Ac-βA, j) Ac, k) N-βA, l) NH_2, m) C-βA, n) Am, o) COOH.

A N_3-KGPQGIWGQLys(N_3)-NH_2 (PanMMP)

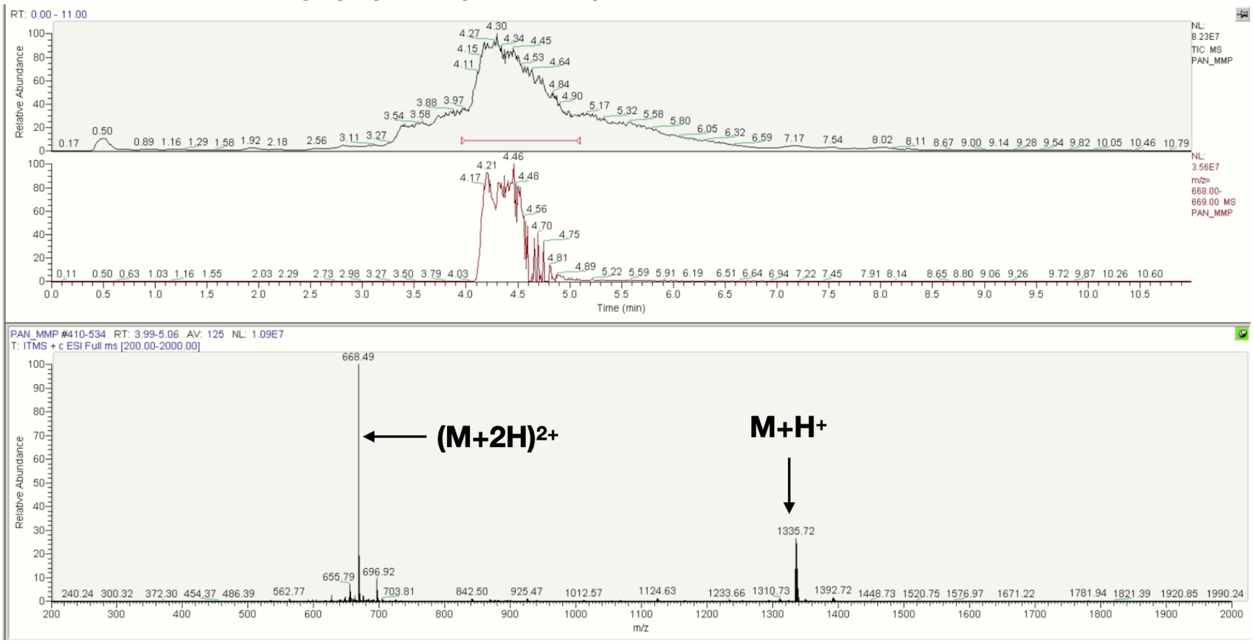

B NH_2-GRGDS-Lys(N_3)-NH_2

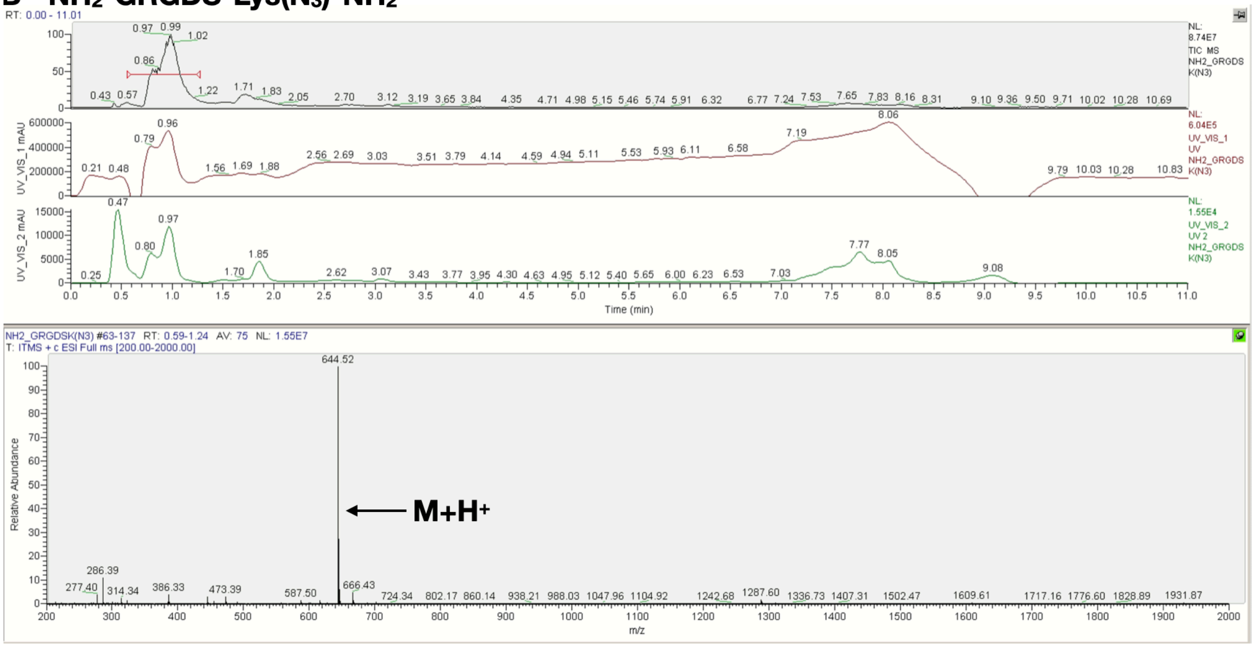

C Ac-βA-GRGDS-Lys(N_3)-NH_2

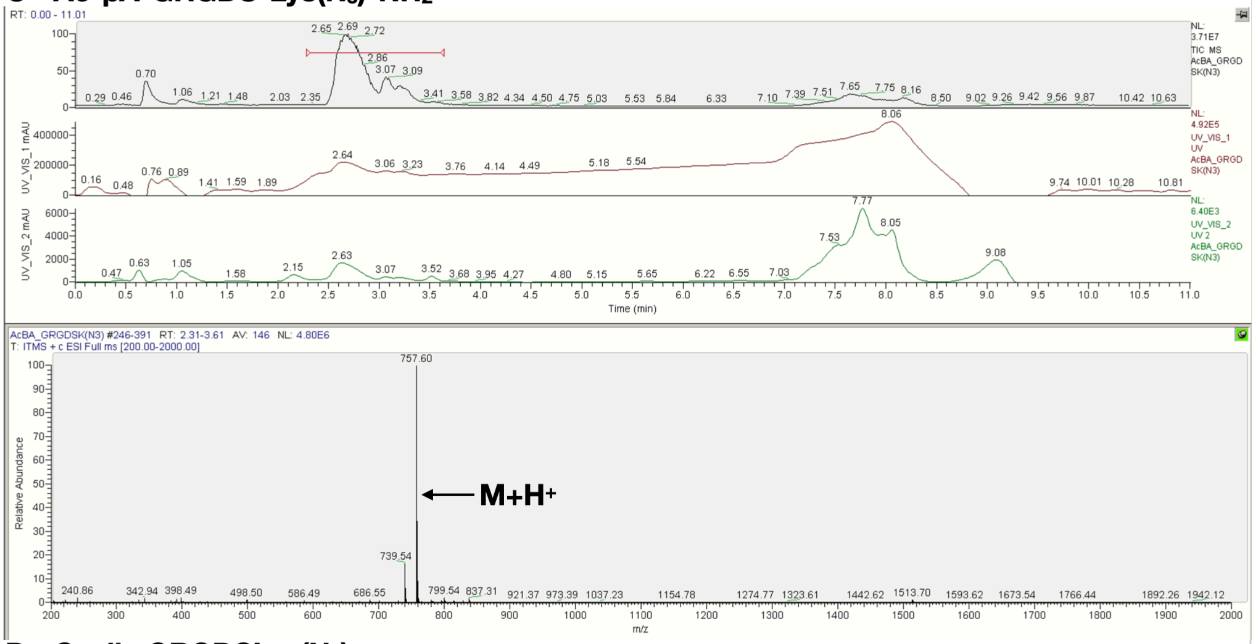

D Cyclic GRGDSLys(N_3)

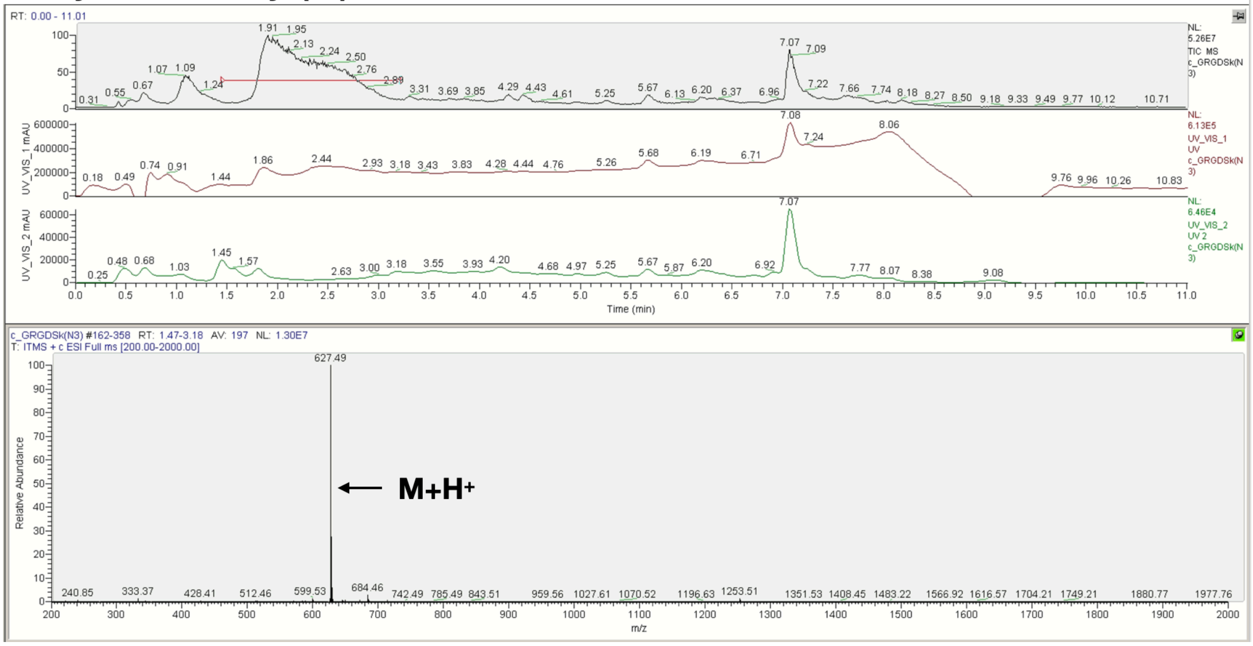

E NH$_2$-βFβAβAβAβAβA-NH$_2$

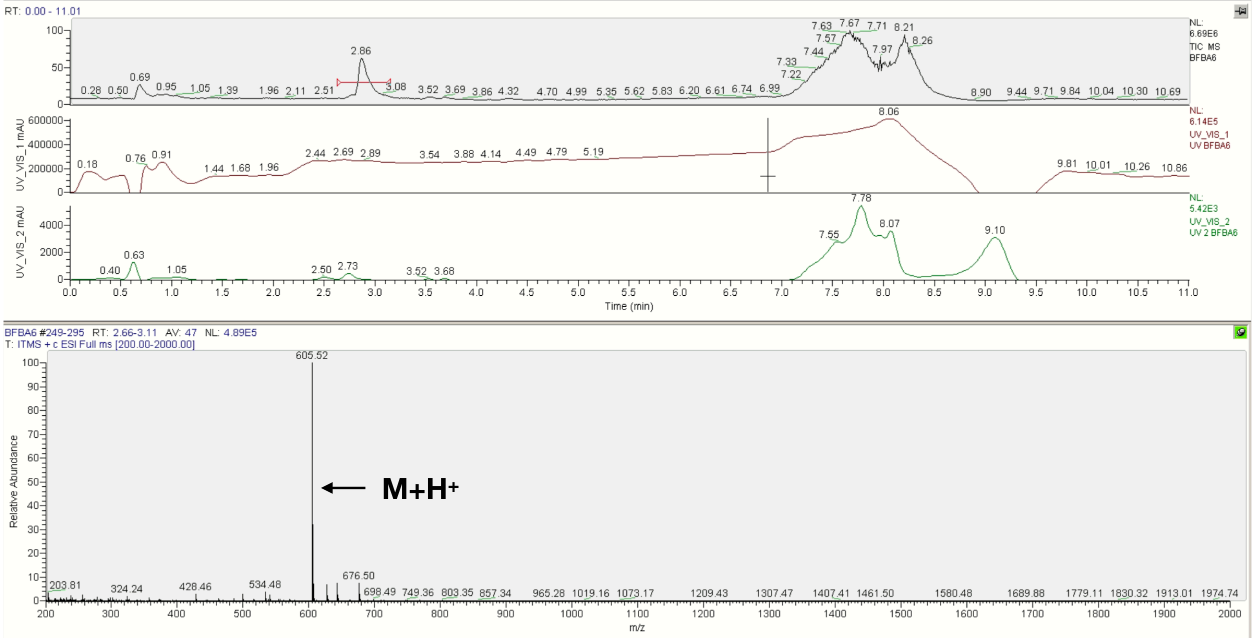

Figure S23. LCMS spectra of the peptides used for cell culture: a) N3-KGPQGIWGQK-Lys(N$_3$)-NH$_2$. Note that the N-terminus of the peptide contained an azide-acetic acid moiety. b) NH$_2$-GRGDS-Lys(N$_3$)-NH$_2$, c) Ac-βA-GRGDS-Lys(N$_3$)-NH$_2$, d) cyclic GRGDS-Lys(N$_3$), e) non-proteolytically degradable NH$_2$-βFβAβAβAβAβA-NH$_2$ internal standard used in all peptide degradation studies.

Statistical Analysis

A **Data for soluble peptides incubated with cells on tissue culture plastic.**

	Df	Sum Sq	Mean Sq	F value	Pr(>F)
celltype	2	279.3	139.65	3125.07	<2e-16 ***
donor	7	12.4	1.77	39.57	<2e-16 ***
experiment	7	22.7	3.24	72.56	<2e-16 ***
endgroup	6	376.8	62.79	1405.17	<2e-16 ***
amino_acid	17	36.4	2.14	47.91	<2e-16 ***
timepoint	5	607.1	121.43	2717.35	<2e-16 ***
residuals	22621	1010.9			

Fit: aov(formula = ave ~ cell + donor + experiment + endgroup + amino_acid + timepoint)

Cell Type

	diff	lwr	upr	p adj
hUVEC-hMSC	0.18174843	0.17324958	0.19024728	0
Macrophage-hMSC	0.26609418	0.25814551	0.27404286	0
Macrophage-hUVEC	0.08434575	0.07639741	0.09229409	0

Endgroup - Averaged over all time points

	diff	lwr	upr	p adj
Am-CBA	-0.035178958	-0.05066638	-0.01969154	0.0000000
COOH-CBA	-0.147212545	-0.16269996	-0.13172512	0.0000000
AcBA-CBA	-0.004391847	-0.01988046	0.01109677	0.9812038
Ac-CBA	-0.036389911	-0.05188451	-0.02089531	0.0000000
NBA-CBA	-0.093876888	-0.10936431	-0.07838947	0.0000000
NH2-CBA	-0.389396425	-0.40489103	-0.37390182	0.0000000
COOH-Am	-0.112033586	-0.12751981	-0.09654736	0.0000000
AcBA-Am	0.030787111	0.01529969	0.04627453	0.0000000
Ac-Am	-0.001210953	-0.01670436	0.01428245	0.9999876
NBA-Am	-0.058697929	-0.07418415	-0.04321170	0.0000000
NH2-Am	-0.354217467	-0.36971087	-0.33872406	0.0000000
AcBA-COOH	0.142820698	0.12733328	0.15830812	0.0000000
Ac-COOH	0.110822633	0.09532923	0.12631604	0.0000000
NBA-COOH	0.053335657	0.03784943	0.06882188	0.0000000
NH2-COOH	-0.242183880	-0.25767729	-0.22669047	0.0000000
Ac-AcBA	-0.031998064	-0.04749267	-0.01650346	0.0000000
NBA-AcBA	-0.089485041	-0.10497246	-0.07399762	0.0000000
NH2-AcBA	-0.385004578	-0.40049918	-0.36950998	0.0000000
NBA-Ac	-0.057486976	-0.07298038	-0.04199357	0.0000000
NH2-Ac	-0.353006513	-0.36850710	-0.33750593	0.0000000
NH2-NBA	-0.295519537	-0.31101294	-0.28002613	0.0000000

Amino Acid

	diff	lwr	upr	p adj
D-A	0.0911418367	0.0617564292	0.1205272442	0.0000000
E-A	0.0674525839	0.0380671764	0.0968379914	0.0000000
F-A	-0.0285623966	-0.0579478041	0.0008230109	0.0680295
G-A	0.0074636879	-0.0219217196	0.0368490954	0.9999903
H-A	-0.0193680806	-0.0487534881	0.0100173269	0.6837704
I_L-A	0.0150238223	-0.0143615852	0.0444092299	0.9485997
K-A	-0.0251598995	-0.0545453070	0.0042255080	0.2061101
M-A	-0.0071542074	-0.0365454494	0.0222370346	0.9999948
N-A	0.0307876787	0.0014022712	0.0601730862	0.0286257
P-A	0.0493901460	0.0200047385	0.0787755535	0.0000005
Q-A	-0.0006024072	-0.0299878147	0.0287830003	1.0000000
R-A	-0.0697643361	-0.0991497436	-0.0403789286	0.0000000
S-A	-0.0104474036	-0.0398328111	0.0189380039	0.9990181
T-A	0.0102079392	-0.0191774683	0.0395933467	0.9992686
V-A	0.0375140807	0.0080876607	0.0669405006	0.0012033
W-A	-0.0644909884	-0.0939115249	-0.0350704518	0.0000000
Y-A	-0.0120895025	-0.0414749100	0.0172959050	0.9943445
E-D	-0.0236892528	-0.0530746603	0.0056961547	0.3045992
F-D	-0.1197042334	-0.1490896409	-0.0903188259	0.0000000
G-D	-0.0836781489	-0.1130635564	-0.0542927414	0.0000000
H-D	-0.1105099174	-0.1398953249	-0.0811245099	0.0000000
I_L-D	-0.0761180144	-0.1055034219	-0.0467326069	0.0000000
K-D	-0.1163017363	-0.1456871438	-0.0869163288	0.0000000
M-D	-0.0982960442	-0.1276872861	-0.0689048022	0.0000000
N-D	-0.0603541581	-0.0897395656	-0.0309687506	0.0000000
P-D	-0.0417516908	-0.0711370983	-0.0123662833	0.0001051
Q-D	-0.0917442439	-0.1211296514	-0.0623588364	0.0000000
R-D	-0.1609061728	-0.1902915803	-0.1315207653	0.0000000
S-D	-0.1015892404	-0.1309746479	-0.0722038329	0.0000000
T-D	-0.0809338976	-0.1103193051	-0.0515484901	0.0000000
V-D	-0.0536277561	-0.0830541760	-0.0242013361	0.0000000
W-D	-0.1556328251	-0.1850533616	-0.1262122886	0.0000000
Y-D	-0.1032313392	-0.1326167467	-0.0738459317	0.0000000
F-E	-0.0960149805	-0.1254003880	-0.0666295730	0.0000000
G-E	-0.0599888960	-0.0893743035	-0.0306034885	0.0000000
H-E	-0.0868206645	-0.1162060720	-0.0574352570	0.0000000
I_L-E	-0.0524287616	-0.0818141691	-0.0230433541	0.0000000
K-E	-0.0926124834	-0.1219978909	-0.0632270759	0.0000000
M-E	-0.0746067913	-0.1039980333	-0.0452155493	0.0000000
N-E	-0.0366649053	-0.0660503128	-0.0072794978	0.0018310
P-E	-0.0180624379	-0.0474478454	0.0113229696	0.7898131
Q-E	-0.0680549911	-0.0974403986	-0.0386695836	0.0000000
R-E	-0.1372169200	-0.1666023275	-0.1078315125	0.0000000
S-E	-0.0778999875	-0.1072853950	-0.0485145800	0.0000000
T-E	-0.0572446447	-0.0866300522	-0.0278592372	0.0000000
V-E	-0.0299385032	-0.0593649232	-0.0005120833	0.0409923
W-E	-0.1319435723	-0.1613641088	-0.1025230357	0.0000000
Y-E	-0.0795420864	-0.1089274939	-0.0501566789	0.0000000
G-F	0.0360260845	0.0066406770	0.0654114920	0.0025440
H-F	0.0091943160	-0.0201910915	0.0385797235	0.9998155
I_L-F	0.0435862190	0.0142008115	0.0729716265	0.0000338
K-F	0.0034024971	-0.0259829104	0.0327879046	1.0000000

M-F	0.0214081892	-0.0079830528	0.0507994312	0.4981737
N-F	0.0593500753	0.0299646678	0.0887354828	0.0000000
P-F	0.0779525426	0.0485671351	0.1073379501	0.0000000
Q-F	0.0279599894	-0.0014254181	0.0573453970	0.0844257
R-F	-0.0412019394	-0.0705873469	-0.0118165319	0.0001460
S-F	0.0181149930	-0.0112704145	0.0475004005	0.7859067
T-F	0.0387703358	0.0093849283	0.0681557433	0.0005903
V-F	0.0660764773	0.0366500574	0.0955028973	0.0000000
W-F	-0.0359285917	-0.0653491282	-0.0065080552	0.0027321
Y-F	0.0164728942	-0.0129125133	0.0458583017	0.8898527
H-G	-0.0268317685	-0.0562171760	0.0025536390	0.1237204
I_L-G	0.0075601345	-0.0218252730	0.0369455420	0.9999883
K-G	-0.0326235874	-0.0620089949	-0.0032381799	0.0129911
M-G	-0.0146178953	-0.0440091373	0.0147733467	0.9599599
N-G	0.0233239908	-0.0060614167	0.0527093983	0.3326789
P-G	0.0419264581	0.0125410506	0.0713118656	0.0000946
Q-G	-0.0080660950	-0.0374515025	0.0213193125	0.9999702
R-G	-0.0772280239	-0.1066134314	-0.0478426164	0.0000000
S-G	-0.0179110915	-0.0472964990	0.0114743160	0.8008656
T-G	0.0027442513	-0.0266411562	0.0321296588	1.0000000
V-G	0.0300503928	0.0006239729	0.0594768128	0.0392224
W-G	-0.0719546762	-0.1013752127	-0.0425341397	0.0000000
Y-G	-0.0195531903	-0.0489385978	0.0098322172	0.6674980
I_L-H	0.0343919030	0.0050064955	0.0637773105	0.0057134
K-H	-0.0057918189	-0.0351772264	0.0235935886	0.9999998
M-H	0.0122138732	-0.0171773688	0.0416051152	0.9936667
N-H	0.0501557593	0.0207703518	0.0795411668	0.0000002
P-H	0.0687582266	0.0393728191	0.0981436341	0.0000000
Q-H	0.0187656735	-0.0106197340	0.0481510810	0.7348440
R-H	-0.0503962554	-0.0797816629	-0.0210108479	0.0000001
S-H	0.0089206770	-0.0204647305	0.0383060845	0.9998777
T-H	0.0295760198	0.0001906123	0.0589614273	0.0464617
V-H	0.0568821613	0.0274557414	0.0863085813	0.0000000
W-H	-0.0451229077	-0.0745434442	-0.0157023712	0.0000130
Y-H	0.0072785782	-0.0221068293	0.0366639857	0.9999933
K-I_L	-0.0401837219	-0.0695691294	-0.0107983144	0.0002651
M-I_L	-0.0221780298	-0.0515692717	0.0072132122	0.4289220
N-I_L	0.0157638563	-0.0136215512	0.0451492638	0.9223924
P-I_L	0.0343663236	0.0049809161	0.0637517311	0.0057841
Q-I_L	-0.0156262295	-0.0450116370	0.0137591780	0.9278480
R-I_L	-0.0847881584	-0.1141735659	-0.0554027509	0.0000000
S-I_L	-0.0254712260	-0.0548566335	0.0039141815	0.1884060
T-I_L	-0.0048158832	-0.0342012907	0.0245695243	1.0000000
V-I_L	0.0224902583	-0.0069361616	0.0519166783	0.4040892
W-I_L	-0.0795148107	-0.1089353472	-0.0500942742	0.0000000
Y-I_L	-0.0271133248	-0.0564987323	0.0022720827	0.1127786
M-K	0.0180056921	-0.0113855499	0.0473969341	0.7942535
N-K	0.0559475782	0.0265621707	0.0853329857	0.0000000
P-K	0.0745500455	0.0451646380	0.1039354530	0.0000000
Q-K	0.0245574924	-0.0048279151	0.0539428999	0.2435191
R-K	-0.0446044365	-0.0739898440	-0.0152190290	0.0000176
S-K	0.0147124959	-0.0146729116	0.0440979034	0.9574387
T-K	0.0353678387	0.0059824312	0.0647532462	0.0035440
V-K	0.0626739802	0.0332475603	0.0921004002	0.0000000

W-K	-0.0393310888	-0.0687516253	-0.0099105523	0.0004429
Y-K	0.0130703971	-0.0163150104	0.0424558046	0.9867309
N-M	0.0379418861	0.0085506441	0.0673331280	0.0009334
P-M	0.0565443534	0.0271531114	0.0859355954	0.0000000
Q-M	0.0065518002	-0.0228394418	0.0359430422	0.9999986
R-M	-0.0626101287	-0.0920013707	-0.0332188867	0.0000000
S-M	-0.0032931962	-0.0326844382	0.0260980458	1.0000000
T-M	0.0173621466	-0.0120290954	0.0467533886	0.8385896
V-M	0.0446682881	0.0152360418	0.0741005344	0.0000177
W-M	-0.0573367809	-0.0867631450	-0.0279104169	0.0000000
Y-M	-0.0049352951	-0.0343265371	0.0244559469	1.0000000
P-N	0.0186024673	-0.0107829402	0.0479878748	0.7480944
Q-N	-0.0313900858	-0.0607754933	-0.0020046783	0.0222507
R-N	-0.1005520147	-0.1299374222	-0.0711666072	0.0000000
S-N	-0.0412350823	-0.0706204898	-0.0118496748	0.0001432
T-N	-0.0205797395	-0.0499651470	0.0088056680	0.5741915
V-N	0.0067264020	-0.0227000179	0.0361528220	0.9999980
W-N	-0.0952786670	-0.1246992035	-0.0658581305	0.0000000
Y-N	-0.0428771811	-0.0722625886	-0.0134917736	0.0000528
Q-P	-0.0499925532	-0.0793779607	-0.0206071457	0.0000002
R-P	-0.1191544821	-0.1485398896	-0.0897690746	0.0000000
S-P	-0.0598375496	-0.0892229571	-0.0304521421	0.0000000
T-P	-0.0391822068	-0.0685676143	-0.0097967993	0.0004690
V-P	-0.0118760653	-0.0413024852	0.0175503547	0.9954584
W-P	-0.1138811343	-0.1433016708	-0.0844605978	0.0000000
Y-P	-0.0614796485	-0.0908650560	-0.0320942410	0.0000000
R-Q	-0.0691619289	-0.0985473364	-0.0397765214	0.0000000
S-Q	-0.0098449964	-0.0392304039	0.0195404111	0.9995420
T-Q	0.0108103464	-0.0185750611	0.0401957539	0.9984965
V-Q	0.0381164879	0.0086900679	0.0675429078	0.0008703
W-Q	-0.0638885812	-0.0933091177	-0.0344680447	0.0000000
Y-Q	-0.0114870953	-0.0408725028	0.0178983122	0.9968695
S-R	0.0593169325	0.0299315250	0.0887023400	0.0000000
T-R	0.0799722752	0.0505868677	0.1093576827	0.0000000
V-R	0.1072784168	0.0778519968	0.1367048367	0.0000000
W-R	0.0052733477	-0.0241471888	0.0346938842	1.0000000
Y-R	0.0576748336	0.0282894261	0.0870602411	0.0000000
T-S	0.0206553428	-0.0087300647	0.0500407503	0.5672063
V-S	0.0479614843	0.0185350644	0.0773879043	0.0000017
W-S	-0.0540435847	-0.0834641212	-0.0246230482	0.0000000
Y-S	-0.0016420988	-0.0310275063	0.0277433087	1.0000000
V-T	0.0273061415	-0.0021202784	0.0567325615	0.1070972
W-T	-0.0746989275	-0.1041194640	-0.0452783910	0.0000000
Y-T	-0.0222974416	-0.0516828491	0.0070879659	0.4180775
W-V	-0.1020050690	-0.1314665691	-0.0725435690	0.0000000
Y-V	-0.0496035832	-0.0790300031	-0.0201771632	0.0000004
Y-W	0.0524014859	0.0229809494	0.0818220224	0.0000000

Donor

	diff	lwr	upr	p adj
3088202-3087423	-0.009686784	-0.0295486217	1.017505e-02	0.8747639
3091412-3087423.	-0.040182618	-0.0600444554	-2.032078e-02	0.0000000
310264-3087423	-0.027216718	-0.0470917219	-7.341713e-03	0.0006227
310268-3087423	-0.019910449	-0.0397744766	-4.642087e-05	0.0488963
310280-3087423	-0.055138247	-0.0750000843	-3.527641e-02	0.0000000
4608-3087423	-0.045885753	-0.0657607575	-2.601075e-02	0.0000000
8119-3087423	-0.025544000	-0.0454058378	-5.682163e-03	0.0019297
8478-3087423	-0.030891304	-0.0507531412	-1.102947e-02	0.0000378
THP1-3087423	-0.086540213	-0.1064042410	-6.667619e-02	0.0000000
3091412-3088202.	-0.030495834	-0.0503576714	-1.063400e-02	0.0000520
310264-3088202	-0.017529934	-0.0374049379	2.345071e-03	0.1394945
310268-3088202	-0.010223665	-0.0300876925	9.640363e-03	0.8344493
310280-3088202.	-0.045451463	-0.0653133002	-2.558962e-02	0.0000000
4608-3088202	-0.036198969	-0.0560739735	-1.632396e-02	0.0000001
8119-3088202	-0.015857216	-0.0357190537	4.004622e-03	0.2543606
8478-3088202	-0.021204519	-0.0410663571	-1.342682e-03	0.0254446
THP1-3088202	-0.076853429	-0.0967174569	-5.698940e-02	0.0000000
310264-3091412	0.012965900	-0.0069091041	3.284090e-02	0.5531567
310268-3091412	0.020272169	0.0004081412	4.013620e-02	0.0409855
310280-3091412	-0.014955629	-0.0348174665	4.906209e-03	0.3363452
4608-3091412	-0.005703135	-0.0255781397	1.417187e-02	0.9962700
8119-3091412	0.014638618	-0.0052232200	3.450046e-02	0.3680951
8478-3091412	0.009291314	-0.0105705234	2.915315e-02	0.9003185
	diff	lwr	upr	p adj
THP1-3091412	-0.046357595	-0.0662216232	-2.649357e-02	0.0000000
310268-310264	0.007306269	-0.0125709241	2.718346e-02	0.9777526
310280-310264	-0.027921529	-0.0477965333	-8.046525e-03	0.0003758
4608-310264	-0.018669036	-0.0385571978	1.219127e-03	0.0873723
8119-310264	0.001672718	-0.0182022868	2.154772e-02	0.9999999
8478-310264	-0.003674586	-0.0235495901	1.620042e-02	0.9998916
THP1-310264	-0.059323495	-0.0792006885	-3.944630e-02	0.0000000
310280-310268	-0.035227798	-0.0550918258	-1.536377e-02	0.0000007
4608-310268	-0.025975305	-0.0458524975	-6.098112e-03	0.0014675
8119-310268	-0.005633551	-0.0254975793	1.423048e-02	0.9965883
8478-310268	-0.010980855	-0.0308448827	8.883173e-03	0.7671006
THP1-310268	-0.066629764	-0.0864959822	-4.676355e-02	0.0000000
4608-310280	0.009252493	-0.0106225109	2.912750e-02	0.9029945
8119-310280	0.029594246	0.0097324088	4.945608e-02	0.0001057
8478-310280	0.024246943	0.0043851055	4.410878e-02	0.0044310
THP1-310280	-0.031401966	-0.0512659943	-1.153794e-02	0.0000250
8119-4608	0.020341753	0.0004667488	4.021676e-02	0.0398183
8478-4608	0.014994450	-0.0048805545	3.486945e-02	0.3335221
THP1-4608	-0.040654460	-0.0605316529	-2.077727e-02	0.0000000
8478-8119	-0.005347303	-0.0252091410	1.451453e-02	0.9977135
THP1-8119	-0.060996213	-0.0808602408	-4.113219e-02	0.0000000
THP1-8478	-0.055648910	-0.0755129374	-3.578488e-02	0.0000000

B **Soluble peptides with cells on tissue culture plastic, 48 hour time point only**

48 hours	Df	Sum Sq	Mean Sq	F value	Pr(>F)
celltype	2	279.3	139.65	1952.84	<2e-16 ***
donor	7	12.4	1.77	24.73	<2e-16 ***
experiment	7	22.7	3.24	45.34	<2e-16 ***
endgroup	6	376.8	62.79	878.08	<2e-16 ***
amino_acid	17	36.4	2.14	29.93	<2e-16 ***
residuals	22626	1618	0.07		

Tukey multiple comparisons of means
95% family-wise confidence level

Fit: aov(formula = ave ~ cell + donor + experiment + endgroup + amino_acid)

**Cell type**	diff	lwr	upr	p adj
hUVEC-hMSC	0.18174843	0.17099723	0.19249964	0
Mac-hMSC	0.26609418	0.25603896	0.27614940	0
Mac-hUVEC	0.08434575	0.07429095	0.09440055	0

**Endgroup**	diff	lwr	upr	p adj
Am-CBA	-0.035178958	-0.05477083	-0.01558708	0.0000023
COOH-CBA	-0.147212545	-0.16680442	-0.12762067	0.0000000
AcBA-CBA	-0.004391847	-0.02398523	0.01520154	0.9946058
Ac-CBA	-0.036389911	-0.05599087	-0.01678895	0.0000007
NBA-CBA	-0.093876888	-0.11346876	-0.07428501	0.0000000
NH2-CBA	-0.389396425	-0.40899738	-0.36979547	0.0000000
COOH-Am	-0.112033586	-0.13162395	-0.09244322	0.0000000
AcBA-Am	0.030787111	0.01119524	0.05037899	0.0000737
Ac-Am	-0.001210953	-0.02081040	0.01838849	0.9999969
NBA-Am	-0.058697929	-0.07828829	-0.03910757	0.0000000
NH2-Am	-0.354217467	-0.37381691	-0.33461802	0.0000000
AcBA-COOH	0.142820698	0.12322882	0.16241257	0.0000000
Ac-COOH	0.110822633	0.09122319	0.13042208	0.0000000
NBA-COOH	0.053335657	0.03374529	0.07292602	0.0000000
NH2-COOH	-0.242183880	-0.26178333	-0.22258443	0.0000000
Ac-AcBA	-0.031998064	-0.05159902	-0.01239711	0.0000305
NBA-AcBA	-0.089485041	-0.10907691	-0.06989317	0.0000000
NH2-AcBA	-0.385004578	-0.40460554	-0.36540362	0.0000000
NBA-Ac	-0.057486976	-0.07708642	-0.03788753	0.0000000
NH2-Ac	-0.353006513	-0.37261504	-0.33339799	0.0000000
NH2-NBA	-0.295519537	-0.31511898	-0.27592009	0.0000000

Amino acid

	diff	lwr	upr	p adj
D-A	0.0911418367	0.0539687514	0.1283149221	0.0000000
E-A	0.0674525839	0.0302794985	0.1046256693	0.0000000
F-A	-0.0285623966	-0.0657354820	0.0086106888	0.3938383
G-A	0.0074636879	-0.0297093975	0.0446367732	0.9999997
H-A	-0.0193680806	-0.0565411660	0.0178050047	0.9393452
I_L-A	0.0150238223	-0.0221492630	0.0521969077	0.9953836
K-A	-0.0251598995	-0.0623329849	0.0120131859	0.6374478
M-A	-0.0071542074	-0.0443346735	0.0300262587	0.9999999
N-A	0.0307876787	-0.0063854067	0.0679607640	0.2581939
P-A	0.0493901460	0.0122170606	0.0865632314	0.0005071
Q-A	-0.0006024072	-0.0377754926	0.0365706782	1.0000000
R-A	-0.0697643361	-0.1069374215	-0.0325912507	0.0000000
S-A	-0.0104474036	-0.0476204890	0.0267256818	0.9999583
T-A	0.0102079392	-0.0269651462	0.0473810246	0.9999700
V-A	0.0375140807	0.0002891138	0.0747390476	0.0457894
W-A	-0.0644909884	-0.1017085126	-0.0272734641	0.0000000
Y-A	-0.0120895025	-0.0492625879	0.0250835829	0.9996903
E-D	-0.0236892528	-0.0608623382	0.0134838325	0.7380570
F-D	-0.1197042334	-0.1568773188	-0.0825311480	0.0000000
G-D	-0.0836781489	-0.1208512343	-0.0465050635	0.0000000
H-D	-0.1105099174	-0.1476830028	-0.0733368320	0.0000000
I_L-D	-0.0761180144	-0.1132910998	-0.0389449290	0.0000000
K-D	-0.1163017363	-0.1534748217	-0.0791286509	0.0000000
M-D	-0.0982960442	-0.1354765103	-0.0611155780	0.0000000
N-D	-0.0603541581	-0.0975272435	-0.0231810727	0.0000020
P-D	-0.0417516908	-0.0789247762	-0.0045786054	0.0109361
Q-D	-0.0917442439	-0.1289173293	-0.0545711585	0.0000000
R-D	-0.1609061728	-0.1980792582	-0.1237330874	0.0000000
S-D	-0.1015892404	-0.1387623258	-0.0644161550	0.0000000
T-D	-0.0809338976	-0.1181069830	-0.0437608122	0.0000000
V-D	-0.0536277561	-0.0908527230	-0.0164027892	0.0000738
W-D	-0.1556328251	-0.1928503493	-0.1184153009	0.0000000
Y-D	-0.1032313392	-0.1404044246	-0.0660582538	0.0000000
F-E	-0.0960149805	-0.1331880659	-0.0588418952	0.0000000
G-E	-0.0599888960	-0.0971619814	-0.0228158107	0.0000025
H-E	-0.0868206645	-0.1239937499	-0.0496475792	0.0000000
I_L-E	-0.0524287616	-0.0896018469	-0.0152556762	0.0001263
K-E	-0.0926124834	-0.1297855688	-0.0554393981	0.0000000
M-E	-0.0746067913	-0.1117872574	-0.0374263252	0.0000000
N-E	-0.0366649053	-0.0738379906	0.0005081801	0.0582158
P-E	-0.0180624379	-0.0552355233	0.0191106475	0.9678116
Q-E	-0.0680549911	-0.1052280765	-0.0308819057	0.0000000
R-E	-0.1372169200	-0.1743900054	-0.1000438346	0.0000000
S-E	-0.0778999875	-0.1150730729	-0.0407269021	0.0000000
T-E	-0.0572446447	-0.0944177301	-0.0200715594	0.0000115
V-E	-0.0299385032	-0.0671634701	0.0072864637	0.3088003
W-E	-0.1319435723	-0.1691610965	-0.0947260480	0.0000000
Y-E	-0.0795420864	-0.1167151718	-0.0423690010	0.0000000
G-F	0.0360260845	-0.0011470009	0.0731991699	0.0701352
H-F	0.0091943160	-0.0279787694	0.0463674014	0.9999934
I_L-F	0.0435862190	0.0064131336	0.0807593044	0.0055425
K-F	0.0034024971	-0.0337705883	0.0405755825	1.0000000

M-F	0.0214081892	-0.0157722769	0.0585886554	0.8654509
N-F	0.0593500753	0.0221769899	0.0965231607	0.0000036
P-F	0.0779525426	0.0407794572	0.1151256280	0.0000000
Q-F	0.0279599894	-0.0092130959	0.0651330748	0.4352011
R-F	-0.0412019394	-0.0783750248	-0.0040288541	0.0133051
S-F	0.0181149930	-0.0190580924	0.0552880784	0.9669115
T-F	0.0387703358	0.0015972504	0.0759434212	0.0303156
V-F	0.0660764773	0.0288515104	0.1033014442	0.0000000
W-F	-0.0359285917	-0.0731461160	0.0012889325	0.0730109
Y-F	0.0164728942	-0.0207001912	0.0536459796	0.9872413
H-G	-0.0268317685	-0.0640048539	0.0103413169	0.5159264
I_L-G	0.0075601345	-0.0296129509	0.0447332199	0.9999997
K-G	-0.0326235874	-0.0697966728	0.0045494980	0.1714720
M-G	-0.0146178953	-0.0517983614	0.0225625709	0.9966506
N-G	0.0233239908	-0.0138490946	0.0604970762	0.7611846
P-G	0.0419264581	0.0047533727	0.0790995435	0.0102676
Q-G	-0.0080660950	-0.0452391804	0.0291069903	0.9999991
R-G	-0.0772280239	-0.1144011093	-0.0400549386	0.0000000
S-G	-0.0179110915	-0.0550841769	0.0192619939	0.9702995
T-G	0.0027442513	-0.0344288341	0.0399173367	1.0000000
V-G	0.0300503928	-0.0071745741	0.0672753597	0.3021633
W-G	-0.0719546762	-0.1091722005	-0.0347371520	0.0000000
Y-G	-0.0195531903	-0.0567262757	0.0176198951	0.9341904
I_L-H	0.0343919030	-0.0027811824	0.0715649884	0.1100495
K-H	-0.0057918189	-0.0429649043	0.0313812665	1.0000000
M-H	0.0122138732	-0.0249665929	0.0493943394	0.9996463
N-H	0.0501557593	0.0129826739	0.0873288447	0.0003604
P-H	0.0687582266	0.0315851412	0.1059313120	0.0000000
Q-H	0.0187656735	-0.0184074119	0.0559387588	0.9541257
R-H	-0.0503962554	-0.0875693408	-0.0132231701	0.0003233
S-H	0.0089206770	-0.0282524084	0.0460937624	0.9999958
T-H	0.0295760198	-0.0075970656	0.0667491052	0.3282941
V-H	0.0568821613	0.0196571944	0.0941071282	0.0000145
W-H	-0.0451229077	-0.0823404320	-0.0079053835	0.0031128
Y-H	0.0072785782	-0.0298945072	0.0444516636	0.9999998
K-I_L	-0.0401837219	-0.0773568073	-0.0030106365	0.0189500
M-I_L	-0.0221780298	-0.0593584959	0.0150024364	0.8274584
N-I_L	0.0157638563	-0.0214092291	0.0529369417	0.9920654
P-I_L	0.0343663236	-0.0028067618	0.0715394090	0.1107945
Q-I_L	-0.0156262295	-0.0527993149	0.0215468559	0.9927993
R-I_L	-0.0847881584	-0.1219612438	-0.0476150730	0.0000000
S-I_L	-0.0254712260	-0.0626443114	0.0117018594	0.6150779
T-I_L	-0.0048158832	-0.0419889686	0.0323572022	1.0000000
V-I_L	0.0224902583	-0.0147347086	0.0597152252	0.8120325
W-I_L	-0.0795148107	-0.1167323349	-0.0422972865	0.0000000
Y-I_L	-0.0271133248	-0.0642864102	0.0100597606	0.4955013
M-K	0.0180056921	-0.0191747740	0.0551861583	0.9688215
N-K	0.0559475782	0.0187744928	0.0931206636	0.0000225
P-K	0.0745500455	0.0373769601	0.1117231309	0.0000000
Q-K	0.0245574924	-0.0126155930	0.0617305777	0.6798651
R-K	-0.0446044365	-0.0817775219	-0.0074313512	0.0037393
S-K	0.0147124959	-0.0224605895	0.0518855813	0.9963790
T-K	0.0353678387	-0.0018052467	0.0725409241	0.0844742
V-K	0.0626739802	0.0254490133	0.0998989471	0.0000004

W-K	-0.0393310888	-0.0765486131	-0.0021135646	0.0256295
Y-K	0.0130703971	-0.0241026883	0.0502434825	0.9991467
N-M	0.0379418861	0.0007614199	0.0751223522	0.0395549
P-M	0.0565443534	0.0193638873	0.0937248195	0.0000166
Q-M	0.0065518002	-0.0306286659	0.0437322663	1.0000000
R-M	-0.0626101287	-0.0997905948	-0.0254296626	0.0000004
S-M	-0.0032931962	-0.0404736624	0.0338872699	1.0000000
T-M	0.0173621466	-0.0198183196	0.0545426127	0.9781475
V-M	0.0446682881	0.0074359507	0.0819006254	0.0037499
W-M	-0.0573367809	-0.0945616771	-0.0201118848	0.0000114
Y-M	-0.0049352951	-0.0421157612	0.0322451711	1.0000000
P-N	0.0186024673	-0.0185706181	0.0557755527	0.9576289
Q-N	-0.0313900858	-0.0685631712	0.0057829996	0.2270870
R-N	-0.1005520147	-0.1377251001	-0.0633789293	0.0000000
S-N	-0.0412350823	-0.0784081677	-0.0040619969	0.0131501
T-N	-0.0205797395	-0.0577528249	0.0165933459	0.9000136
V-N	0.0067264020	-0.0304985649	0.0439513689	0.9999999
W-N	-0.0952786670	-0.1324961912	-0.0580611428	0.0000000
Y-N	-0.0428771811	-0.0800502665	-0.0057040957	0.0072404
Q-P	-0.0499925532	-0.0871656386	-0.0128194678	0.0003878
R-P	-0.1191544821	-0.1563275675	-0.0819813967	0.0000000
S-P	-0.0598375496	-0.0970106350	-0.0226644642	0.0000027
T-P	-0.0391822068	-0.0763552922	-0.0020091214	0.0265046
V-P	-0.0118760653	-0.0491010322	0.0253489016	0.9997602
W-P	-0.1138811343	-0.1510986586	-0.0766636101	0.0000000
Y-P	-0.0614796485	-0.0986527338	-0.0243065631	0.0000010
R-Q	-0.0691619289	-0.1063350143	-0.0319888435	0.0000000
S-Q	-0.0098449964	-0.0470180818	0.0273280889	0.9999822
T-Q	0.0108103464	-0.0263627390	0.0479834317	0.9999325
V-Q	0.0381164879	0.0008915210	0.0753414548	0.0379863
W-Q	-0.0638885812	-0.1011061054	-0.0266710569	0.0000001
Y-Q	-0.0114870953	-0.0486601807	0.0256859901	0.9998442
S-R	0.0593169325	0.0221438471	0.0964900178	0.0000037
T-R	0.0799722752	0.0427991899	0.1171453606	0.0000000
V-R	0.1072784168	0.0700534499	0.1445033837	0.0000000
W-R	0.0052733477	-0.0319441765	0.0424908720	1.0000000
Y-R	0.0576748336	0.0205017482	0.0948479190	0.0000091
T-S	0.0206553428	-0.0165177426	0.0578284282	0.8971104
V-S	0.0479614843	0.0107365174	0.0851864512	0.0009712
W-S	-0.0540435847	-0.0912611090	-0.0168260605	0.0000600
Y-S	-0.0016420988	-0.0388151842	0.0355309866	1.0000000
V-T	0.0273061415	-0.0099188254	0.0645311084	0.4843365
W-T	-0.0746989275	-0.1119164518	-0.0374814033	0.0000000
Y-T	-0.0222974416	-0.0594705270	0.0148756438	0.8208499
W-V	-0.1020050690	-0.1392744129	-0.0647357252	0.0000000
Y-V	-0.0496035832	-0.0868285501	-0.0123786163	0.0004757
Y-W	0.0524014859	0.0151839616	0.0896190101	0.0001318

Donor	diff	lwr	upr	p adj
3088202-3087423	-0.009686784	-0.0348123771	0.0154388090	0.9694090
3091412-3087423	-0.040182618	-0.0653082108	-0.0150570248	0.0000184
310264-3087423	-0.027216718	-0.0523589667	-0.0020744686	0.0217447
310268-3087423	-0.019910449	-0.0450388124	0.0052179149	0.2644817
310280-3087423	-0.055138247	-0.0802638397	-0.0300126536	0.0000000
4608-3087423	-0.045885753	-0.0710280023	-0.0207435042	0.0000001
8119-3087423	-0.025544000	-0.0506695932	-0.0004184071	0.0425781
8478-3087423	-0.030891304	-0.0560168965	-0.0057657105	0.0039781
THP1-3087423	-0.086540213	-0.1116685768	-0.0614118494	0.0000000
3091412-3088202	-0.030495834	-0.0556214267	-0.0053702407	0.0048322
310264-3088202	-0.017529934	-0.0426721826	0.0076123154	0.4524902
310268-3088202	-0.010223665	-0.0353520283	0.0149046990	0.9567040
310280-3088202	-0.045451463	-0.0705770556	-0.0203258696	0.0000002
4608-3088202	-0.036198969	-0.0613412182	-0.0110567202	0.0002255
8119-3088202	-0.015857216	-0.0409828091	0.0092683769	0.6012720
8478-3088202	-0.021204519	-0.0463301125	0.0039210736	0.1858617
THP1-3088202	-0.076853429	-0.1019817927	-0.0517250654	0.0000000
310264-3091412	0.012965900	-0.0121763489	0.0381081492	0.8328010
310268-3091412	0.020272169	-0.0048561946	0.0454005327	0.2407125
310280-3091412	-0.014955629	-0.0400812219	0.0101699642	0.6805488
4608-3091412	-0.005703135	-0.0308453845	0.0194391136	0.9994131
8119-3091412	0.014638618	-0.0104869754	0.0397642106	0.7073104
8478-3091412	0.009291314	-0.0158342788	0.0344169073	0.9768263
THP1-3091412	-0.046357595	-0.0714859590	-0.0212292317	0.0000000
310268-310264	0.007306269	-0.0178387489	0.0324512868	0.9958988
310280-310264	-0.027921529	-0.0530637780	-0.0027792800	0.0160568
4608-310264	-0.018669036	-0.0438279296	0.0064898584	0.3579557
8119-310264	0.001672718	-0.0234695315	0.0268149665	1.0000000
8478-310264	-0.003674586	-0.0288168349	0.0214676632	0.9999854
THP1-310264	-0.059323495	-0.0844685133	-0.0341784776	0.0000000
310280-310268	-0.035227798	-0.0603561616	-0.0100994343	0.0003921
4608-310268	-0.025975305	-0.0511203224	-0.0008302867	0.0362244
8119-310268	-0.005633551	-0.0307619151	0.0194948122	0.9994664
8478-310268	-0.010980855	-0.0361092185	0.0141475089	0.9327976
THP1-310268	-0.066629764	-0.0917608984	-0.0414986304	0.0000000
4608-310280	0.009252493	-0.0158897556	0.0343947424	0.9775736
8119-310280	0.029594246	0.0044686535	0.0547198395	0.0074457
8478-310280	0.024246943	-0.0008786499	0.0493725361	0.0691966
THP1-310280	-0.031401966	-0.0565303301	-0.0062736028	0.0030867
8119-4608	0.020341753	-0.0048004959	0.0454840021	0.2370098
8478-4608	0.014994450	-0.0101477993	0.0401366988	0.6780747
THP1-4608	-0.040654460	-0.0657994777	-0.0155094420	0.0000137
8478-8119	-0.005347303	-0.0304728964	0.0197782897	0.9996511
THP1-8119	-0.060996213	-0.0861245766	-0.0358678493	0.0000000
THP1-8478	-0.055648910	-0.0807772733	-0.0305205459	0.0000000

C Concentration study peptides at 48 hours

48 hours	Df	Sum Sq	Mean Sq	F value	Pr(>F)
celltype	2	7.184	3.592	97.36	<2e-16 ***
endgroup	6	21.549	3.591	97.34	<2e-16 ***
concentration	4	3.989	0.997	27.03	<2e-16 ***
residuals	302	11.142	0.037		

```
  Tukey multiple comparisons of means
    95% family-wise confidence level
Fit: aov(formula = ave ~ Cell + endgroup + concentration, data = concentration_48_stats)
```

Cell type

```
                          diff        lwr        upr     p adj
hUVEC-hMSC       0.02674739 -0.0356911 0.08918588 0.5717588
macrophage-hMSC  0.33290148  0.2704630 0.39533997 0.0000000
macrophage-hUVEC 0.30615409  0.2437156 0.36859258 0.0000000
```

Endgroup

```
                diff         lwr         upr     p adj
Ac_BA-Ac   -0.02869210 -0.14889144  0.09150724 0.9920278
Am-Ac      -0.30279864 -0.42299798 -0.18259929 0.0000000
CBA-Ac     -0.05351273 -0.17371207  0.06668661 0.8415104
COOH-Ac    -0.49469442 -0.61489376 -0.37449508 0.0000000
NBA-Ac     -0.36386417 -0.48406351 -0.24366483 0.0000000
NH2-Ac     -0.76234755 -0.88254689 -0.64214820 0.0000000
Am-Ac_BA   -0.27410654 -0.39430588 -0.15390719 0.0000000
CBA-Ac_BA  -0.02482063 -0.14501997  0.09537871 0.9963800
COOH-Ac_BA -0.46600232 -0.58620166 -0.34580298 0.0000000
NBA-Ac_BA  -0.33517207 -0.45537141 -0.21497273 0.0000000
NH2-Ac_BA  -0.73365544 -0.85385479 -0.61345610 0.0000000
CBA-Am      0.24928590  0.12908656  0.36948525 0.0000000
COOH-Am    -0.19189578 -0.31209513 -0.07169644 0.0000675
NBA-Am     -0.06106553 -0.18126487  0.05913381 0.7399153
NH2-Am     -0.45954891 -0.57974825 -0.33934957 0.0000000
COOH-CBA   -0.44118169 -0.56138103 -0.32098234 0.0000000
NBA-CBA    -0.31035144 -0.43055078 -0.19015209 0.0000000
NH2-CBA    -0.70883481 -0.82903416 -0.58863547 0.0000000
NBA-COOH    0.13083025  0.01063091  0.25102959 0.0229651
NH2-COOH   -0.26765313 -0.38785247 -0.14745378 0.0000000
NH2-NBA    -0.39848338 -0.51868272 -0.27828403 0.0000000
```

Concentration

```
                diff         lwr         upr     p adj
19.5-1250 -0.120196418 -0.21411722 -0.02627562 0.0046316
312-1250  -0.067417531 -0.16133833  0.02650327 0.2832901
5000-1250  0.186144359  0.09222356  0.28006516 0.0000011
78-1250   -0.110330119 -0.20425092 -0.01640932 0.0121507
312-19.5   0.052778887 -0.04114191  0.14669968 0.5358579
```

5000-19.5 0.306340777 0.21241998 0.40026157 0.0000000
78-19.5 0.009866299 -0.08405450 0.10378710 0.9984852
5000-312 0.253561889 0.15964109 0.34748269 0.0000000
78-312 -0.042912589 -0.13683339 0.05100821 0.7195936
78-5000 -0.296474478 -0.39039527 -0.20255368 0.0000000

D LIAANK peptides at 48 hours

48 hours	Df	Sum Sq	Mean Sq	F value	Pr(>F)
celltype	2	0.082	0.0409	2.13	0.129
endgroup	6	5.773	0.9621	50.11	<2e-16 ***
residuals	54	1.037	0.0192		

Tukey multiple comparisons of means
95% family-wise confidence level
Fit: aov(formula = ave ~ celltype + endgroup)

**Cell type**	diff	lwr	upr	p adj
Macrophage-hUVEC	-0.072990023	-0.1760419	0.03006182	0.2119027
hMSC-hUVEC.	-0.079463872	-0.1825157	0.02358797	0.1607616
hMSC-Macrophage	-0.006473849	-0.1095257	0.09657800	0.9874458

**Endgroup**	diff	lwr	upr	p adj
Ac-AcBA	0.07693082	-0.123081655	0.27694329	0.8994233
NBA-AcBA	-0.35614312	-0.556155600	-0.15613065	0.0000252
Am-AcBA	0.13334378	-0.066668695	0.33335625	0.4015930
CBA-AcBAG	0.19615704	-0.003855434	0.39616952	0.0579509
COOH-AcBA	-0.45166561	-0.651678083	-0.25165313	0.0000001
NH2-AcBA	-0.63354877	-0.833561246	-0.43353630	0.0000000
NBA-Ac	-0.43307394	-0.633086420	-0.23306147	0.0000003
Am-Ac	0.05641296	-0.143599515	0.25642543	0.9763913
CBA-Ac	0.11922622	-0.080786254	0.31923870	0.5373659
COOH-Ac	-0.52859643	-0.728608903	-0.32858395	0.0000000
NH2-Ac	-0.71047959	-0.910492066	-0.51046712	0.0000000
Am-NBA	0.48948691	0.289474430	0.68949938	0.0000000
CBA-NBA	0.55230017	0.352287691	0.75231264	0.0000000
COOH-NBA	-0.09552248	-0.295534958	0.10448999	0.7652009
NH2-NBA	-0.27740565	-0.477418121	-0.07739317	0.0015813
CBA-Am	0.06281326	-0.137199214	0.26282574	0.9599996
COOH-Am	-0.58500939	-0.785021863	-0.38499691	0.0000000
NH2G-Am	-0.76689255	-0.966905026	-0.56688008	0.0000000
COOH-CBA	-0.64782265	-0.847835124	-0.44781017	0.0000000
NH2-CBA	-0.82970581	-1.029718287	-0.62969334	0.0000000
NH2-COOH	-0.18188316	-0.381895638	0.01812931	0.0975390

E IVKVA peptides at 48 hours

48 hours	Df	Sum Sq	Mean Sq	F value	Pr(>F)
celltype	2	0.263	0.1314	2.027	0.141577
endgroup	6	1.853	0.3089	4.767	0.000578
residuals	54	3.499	0.0648		

Tukey multiple comparisons of means
95% family-wise confidence level

Fit: aov(formula = ave ~ celltype + endgroup)

**Celltype**	diff	lwr	upr	p adj
Macrophage-hUVEC	-0.01503481	-0.20436175	0.1742921	0.9800181
hMSC-hUVEC	0.12886108	-0.06046586	0.3181880	0.2377607
hMSC-Macrophage	0.14389589	-0.04543105	0.3332228	0.1690231

**Endgroup**	diff	lwr	upr	p adj
Ac-AcBA	0.30851472	-0.05894836	0.6759778073	0.1555552
NBA-AcBA	-0.25471927	-0.62218235	0.1127438195	0.3546125
Am-AcBA	-0.02456120	-0.39202429	0.3429018828	0.9999932
CBA-AcBA	0.09053903	-0.27692406	0.4580021099	0.9881916
COOH-AcBA	-0.18941059	-0.55687368	0.1780524904	0.6962825
NH2-AcBA	-0.05986569	-0.42732878	0.3075973939	0.9987708
NBA-Ac	-0.56323399	-0.93069707	-0.1957709032	0.0003595
Am-Ac	-0.33307592	-0.70053901	0.0343871602	0.0995625
CBA-Ac	-0.21797570	-0.58543878	0.1494873873	0.5431482
COOH-Ac	-0.49792532	-0.86538840	-0.1304622322	0.0021639
NH2-Ac	-0.36838041	-0.73584350	-0.0009173288	0.0490434
Am-NBA	0.23015806	-0.13730502	0.5976211480	0.4777630
CBA-NBA	0.34525829	-0.02220479	0.7127213751	0.0786424
COOH-NBA	0.06530867	-0.30215441	0.4327717556	0.9979950
NH2-NBA	0.19485357	-0.17260951	0.5623166590	0.6678880
CBA-Am	0.11510023	-0.25236286	0.4825633118	0.9604966
COOH-Am	-0.16484939	-0.53231248	0.2026136923	0.8130976
NH2-Am	-0.03530449	-0.40276757	0.3321585957	0.9999420
COOH-CBA	-0.27994962	-0.64741270	0.0875134652	0.2477438
NH2-CBA	-0.15040472	-0.51786780	0.2170583686	0.8695873
NH2-COOH	0.12954490	-0.23791818	0.4970079881	0.9314373

F **Soluble peptides with cells encapsulated in PEG hydrogels at 48 hours**

48 hours	Df	Sum Sq	Mean Sq	F value	Pr(>F)
celltype	2	8.19	4.095	125.42	<2e-16 ***
endgroup	6	74.10	12.350	378.28	<2e-16 ***
amino_acid	1081	5.87	0.345	10.58	<2e-16 ***
residuals	22626	35.29	0.033		

Tukey multiple comparisons of means
95% family-wise confidence level
Fit: aov(formula = ave ~ cell + endgroup + amino_acid)

**Cell type**	diff	lwr	upr	p adj
hUVEC-hMSC.	0.05360174	0.02261208	0.08459139	0.0001559
Macrophage-hMSC	0.20456374	0.17320883	0.23591865	0.0000000
Macrophage-hUVEC	0.15096200	0.11962755	0.18229646	0.0000000

**Endgroup**	diff	lwr	upr	p adj
AcBA-Ac	0.03708977	-0.02221344	0.09639299	0.5160796
Am-Ac	-0.04452478	-0.10392001	0.01487045	0.2885047
CBA-Ac	0.07091934	0.01161613	0.13022256	0.0078020
COOH-Ac	-0.38434272	-0.44641560	-0.32226984	0.0000000
NBA-Ac	-0.08609557	-0.14539878	-0.02679236	0.0003919
NH2-Ac	-0.68822281	-0.74752602	-0.62891960	0.0000000
Am-AcBA	-0.08161455	-0.14100978	-0.02221933	0.0010355
CBA-AcBA	0.03382957	-0.02547364	0.09313278	0.6262622
COOH-AcBA	-0.42143249	-0.48350537	-0.35935961	0.0000000
NBA-AcBA	-0.12318534	-0.18248856	-0.06388213	0.0000000
NH2-AcBA	-0.72531258	-0.78461579	-0.66600937	0.0000000
CBA-Am	0.11544412	0.05604890	0.17483935	0.0000003
COOH-Am	-0.33981794	-0.40197873	-0.27765714	0.0000000
NBA-Am	-0.04157079	-0.10096602	0.01782444	0.3729682
NH2-Am	-0.64369803	-0.70309326	-0.58430280	0.0000000
COOH-CBA	-0.45526206	-0.51733494	-0.39318918	0.0000000
NBA-CBA	-0.15701491	-0.21631813	-0.09771170	0.0000000
NH2-CBA	-0.75914215	-0.81844536	-0.69983894	0.0000000
NBA-COOH	0.29824715	0.23617427	0.36032003	0.0000000
NH2-COOH	-0.30388009	-0.36595297	-0.24180721	0.0000000
NH2-NBA	-0.60212724	-0.66143045	-0.54282403	0.0000000

Amino acid

	diff	lwr	upr	p adj
D-A	0.1757707421	0.062276920	0.289264564	0.0000112
E-A	0.1257644436	0.012270622	0.239258265	0.0135034
F-A	-0.0196719476	-0.133165769	0.093821874	1.0000000
G-A	0.0399516470	-0.073542175	0.153445469	0.9990828
H-A	-0.0762467186	-0.189740541	0.037247103	0.6465183
I/L-A	0.0768747060	-0.036619116	0.190368528	0.6318774
K-A	-0.0205584614	-0.134052283	0.092935360	0.9999999
M-A	0.0501176495	-0.063376172	0.163611471	0.9871761

N-A	0.0738796244	-0.039614197	0.187373446	0.7003139
P-A	0.1097088102	-0.003785012	0.223202632	0.0718473
Q-A	0.0332618800	-0.080231942	0.146755702	0.9999196
R-A	-0.0392564012	-0.156878612	0.078365810	0.9995371
S-A	0.0100754237	-0.103418398	0.123569246	1.0000000
T-A	0.1035305588	-0.010427452	0.217488570	0.1285330
V-A	0.0913764269	-0.022117395	0.204870249	0.3043976
W-A	-0.0839188068	-0.197412629	0.029575015	0.4650114
Y-A	-0.0678269774	-0.181320799	0.045666845	0.8217720
E-D	-0.0500062985	-0.163500120	0.063487523	0.9874732
F-D	-0.1954426897	-0.308936512	-0.081948868	0.0000004
G-D	-0.1358190951	-0.249312917	-0.022325273	0.0040162
H-D	-0.2520174607	-0.365511283	-0.138523639	0.0000000
I/L-D	-0.0988960361	-0.212389858	0.014597786	0.1798358
K-D	-0.1963292035	-0.309823025	-0.082835382	0.0000003
M-D	-0.1256530926	-0.239146914	-0.012159271	0.0136767
N-D	-0.1018911177	-0.215384940	0.011602704	0.1420506
P-D	-0.0660619319	-0.179555754	0.047431890	0.8513349
Q-D	-0.1425088621	-0.256002684	-0.029015040	0.0016811
R-D	-0.2150271433	-0.332649354	-0.097404933	0.0000000
S-D	-0.1656953184	-0.279189140	-0.052201496	0.0000573
T-D	-0.0722401832	-0.186198194	0.041717828	0.7420538
V-D	-0.0843943152	-0.197888137	0.029099507	0.4539850
W-D	-0.2596895489	-0.373183371	-0.146195727	0.0000000
Y-D	-0.2435977194	-0.357091541	-0.130103898	0.0000000
F-E	-0.1454363912	-0.258930213	-0.031942569	0.0011309
G-E	-0.0858127966	-0.199306618	0.027681025	0.4215805
H-E	-0.2020111622	-0.315504984	-0.088517340	0.0000001
I/L-E	-0.0488897376	-0.162383559	0.064604084	0.9901557
K-E	-0.1463229050	-0.259816727	-0.032829083	0.0010012
M-E	-0.0756467940	-0.189140616	0.037847028	0.6603793
N-E	-0.0518848192	-0.165378641	0.061609003	0.9816517
P-E	-0.0160556334	-0.129549455	0.097438188	1.0000000
Q-E	-0.0925025635	-0.205996385	0.020991258	0.2830723
R-E	-0.1650208448	-0.282643056	-0.047398634	0.0001551
S-E	-0.1156890198	-0.229182842	-0.002195198	0.0401467
T-E	-0.0222338847	-0.136191896	0.091724126	0.9999998
V-E	-0.0343880166	-0.147881839	0.079105805	0.9998728
W-E	-0.2096832504	-0.323177072	-0.096189429	0.0000000
Y-E	-0.1935914209	-0.307085243	-0.080097599	0.0000005
G-F	0.0596235946	-0.053870227	0.173117416	0.9329647
H-F	-0.0565747710	-0.170068593	0.056919051	0.9577398
I/L-F	0.0965466536	-0.016947168	0.210040475	0.2141716
K-F	-0.0008865138	-0.114380336	0.112607308	1.0000000
M-F	0.0697895971	-0.043704225	0.183283419	0.7855322
N-F	0.0935515720	-0.019942250	0.207045394	0.2640272
P-F	0.1293807578	0.015886936	0.242874580	0.0088518
Q-F	0.0529338276	-0.060559994	0.166427650	0.9775717
R-F	-0.0195844536	-0.137206664	0.098037757	1.0000000
S-F	0.0297473713	-0.083746451	0.143241193	0.9999835
T-F	0.1232025064	0.009244495	0.237160518	0.0190700
V-F	0.1110483745	-0.002445447	0.224542196	0.0633443
W-F	-0.0642468592	-0.177740681	0.049246963	0.8785525
Y-F	-0.0481550298	-0.161648852	0.065338792	0.9916496

H-G	-0.1161983656	-0.229692187	-0.002704544	0.0381172
I/L-G	0.0369230590	-0.076570763	0.150416881	0.9996690
K-G	-0.0605101084	-0.174003930	0.052983713	0.9241648
M-G	0.0101660025	-0.103327819	0.123659824	1.0000000
N-G	0.0339279774	-0.079565844	0.147421799	0.9998943
P-G	0.0697571632	-0.043736659	0.183250985	0.7861582
Q-G	-0.0066897669	-0.120183589	0.106804055	1.0000000
R-G	-0.0792080482	-0.196830259	0.038414163	0.6423060
S-G	-0.0298762233	-0.143370045	0.083617599	0.9999824
T-G	0.0635789119	-0.050379099	0.177536923	0.8911749
V-G	0.0514247799	-0.062069042	0.164918602	0.9832443
W-G	-0.1238704538	-0.237364276	-0.010376632	0.0167386
Y-G	-0.1077786243	-0.221272446	0.005715198	0.0857491
I/L-H	0.1531214246	0.039627603	0.266615246	0.0003828
K-H	0.0556882572	-0.057805565	0.169182079	0.9634656
M-H	0.1263643682	0.012870546	0.239858190	0.0126036
N-H	0.1501263430	0.036632521	0.263620165	0.0005881
P-H	0.1859555288	0.072461707	0.299449351	0.0000020
Q-H	0.1095085987	-0.003985223	0.223002421	0.0731963
R-H	0.0369903174	-0.080631893	0.154612528	0.9997893
S-H	0.0863221424	-0.027171680	0.199815964	0.4101494
T-H	0.1797772775	0.065819266	0.293735289	0.0000065
V-H	0.1676231456	0.054129324	0.281116967	0.0000422
W-H	-0.0076720882	-0.121165910	0.105821734	1.0000000
Y-H	0.0084197413	-0.105074081	0.121913563	1.0000000
K-I/L	-0.0974331674	-0.210926989	0.016060654	0.2007222
M-I/L	-0.0267570565	-0.140250878	0.086736765	0.9999965
N-I/L	-0.0029950816	-0.116488903	0.110498740	1.0000000
P-I/L	0.0328341042	-0.080659718	0.146327926	0.9999328
Q-I/L	-0.0436128260	-0.157106648	0.069880996	0.9973099
R-I/L	-0.1161311072	-0.233753318	0.001491104	0.0575256
S-I/L	-0.0667992823	-0.180293104	0.046694540	0.8393481
T-I/L	0.0266558529	-0.087302158	0.140613864	0.9999969
V-I/L	0.0145017209	-0.098992101	0.127995543	1.0000000
W-I/L	-0.1607935128	-0.274287335	-0.047299691	0.0001223
Y-I/L	-0.1447016834	-0.258195505	-0.031207861	0.0012503
M-K	0.0706761109	-0.042817711	0.184169933	0.7680902
N-K	0.0944380858	-0.019055736	0.207931908	0.2485655
P-K	0.1302672716	0.016773450	0.243761093	0.0079620
Q-K	0.0538203414	-0.059673480	0.167314163	0.9735982
R-K	-0.0186979398	-0.136320151	0.098924271	1.0000000
S-K	0.0306338851	-0.082859937	0.144127707	0.9999748
T-K	0.1240890203	0.010131009	0.238047031	0.0172853
V-K	0.1119348883	-0.001558934	0.225428710	0.0581955
W-K	-0.0633603454	-0.176854167	0.050133476	0.8906427
Y-K	-0.0472685160	-0.160762338	0.066225306	0.9931985
N-M	0.0237619749	-0.089731847	0.137255797	0.9999994
P-M	0.0595911607	-0.053902661	0.173084983	0.9332725
Q-M	-0.0168557695	-0.130349591	0.096638052	1.0000000
R-M	-0.0893740507	-0.206996261	0.028248160	0.4120489
S-M	-0.0400422258	-0.153536048	0.073451596	0.9990562
T-M	0.0534129093	-0.060545102	0.167370921	0.9764516
V-M	0.0412587774	-0.072235044	0.154752599	0.9986283
W-M	-0.1340364563	-0.247530278	-0.020542634	0.0050226

Y-M	-0.1179446269	-0.231438449	-0.004450805	0.0318220
P-N	0.0358291858	-0.077664636	0.149323008	0.9997782
Q-N	-0.0406177444	-0.154111566	0.072876078	0.9988710
R-N	-0.1131360256	-0.230758236	0.004486185	0.0755597
S-N	-0.0638042007	-0.177298023	0.049689621	0.8846887
T-N	0.0296509344	-0.084307077	0.143608946	0.9999852
V-N	0.0174968025	-0.095997019	0.130990624	1.0000000
W-N	-0.1577984312	-0.271292253	-0.044304609	0.0001923
Y-N	-0.1417066018	-0.255200424	-0.028212780	0.0018710
Q-P	-0.0764469302	-0.189940752	0.037046892	0.6418642
R-P	-0.1489652114	-0.266587422	-0.031343001	0.0014252
S-P	-0.0996333865	-0.213127208	0.013860435	0.1699198
T-P	-0.0061782513	-0.120136263	0.107779760	1.0000000
V-P	-0.0183323833	-0.131826205	0.095161439	1.0000000
W-P	-0.1936276170	-0.307121439	-0.080133795	0.0000005
Y-P	-0.1775357876	-0.291029609	-0.064041966	0.0000083
R-Q	-0.0725182812	-0.190140492	0.045103930	0.7819764
S-Q	-0.0231864563	-0.136680278	0.090307366	0.9999996
T-Q	0.0702686788	-0.043689332	0.184226690	0.7817920
V-Q	0.0581145469	-0.055379275	0.171608369	0.9462545
W-Q	-0.1171806869	-0.230674509	-0.003686865	0.0344542
Y-Q	-0.1010888574	-0.214582679	0.012404964	0.1515292
S-R	0.0493318249	-0.068290386	0.166954036	0.9926493
T-R	0.1427869601	0.024716789	0.260857131	0.0033464
V-R	0.1306328281	0.013010617	0.248255039	0.0130714
W-R	-0.0446624056	-0.162284616	0.072959805	0.9976678
Y-R	-0.0285705762	-0.146192787	0.089051635	0.9999946
T-S	0.0934551351	-0.020502876	0.207413146	0.2725897
V-S	0.0813010032	-0.032192819	0.194794825	0.5267417
W-S	-0.0939942305	-0.207488052	0.019499591	0.2562335
Y-S	-0.0779024011	-0.191396223	0.035591421	0.6076844
V-T	-0.0121541319	-0.126112143	0.101803879	1.0000000
W-T	-0.1874493657	-0.301407377	-0.073491354	0.0000017
Y-T	-0.1713575362	-0.285315547	-0.057399525	0.0000260
W-V	-0.1752952337	-0.288789056	-0.061801412	0.0000121
Y-V	-0.1592034043	-0.272697226	-0.045709582	0.0001557
Y-W	0.0160918295	-0.097401992	0.129585651	1.0000000

G Peptides with either an azide or PEG functionalization at 48 hours

48 hours	Df	Sum Sq	Mean Sq	F value	Pr(>F)
celltype	2	0.610	0.3050	8.218	0.000459 ***
endgroup	6	4.996	0.8326	22.435	<2e-16 ***
functionalization	1	2.784	2.7837	75.004	3.19e-14 ***
residuals	116	4.305	0.0371		

Tukey multiple comparisons of means
95% family-wise confidence level
Fit: aov(formula = ave ~ cell + endgroup + functionalization)

**Cell type**	diff	lwr	upr	p adj
hUVEC-hMSC.	0.09160999	-0.008199751	0.1914197	0.0790515
mac-hMSC	0.17027349	0.070463754	0.2700832	0.0002719
mac-hUVEC.	0.07866350	-0.021146233	0.1784732	0.1516898

**Endgroup**	diff	lwr	upr	p adj
AcBA-Ac	0.003644893	-0.18905250	0.196342286	1.0000000
Am-Ac	-0.147102880	-0.33980027	0.045594513	0.2574162
CBA-Ac	0.069312949	-0.12338444	0.262010342	0.9329731
COOH-Ac	-0.208820203	-0.40151760	-0.016122810	0.0245513
NBA-Ac	-0.193966891	-0.38666428	-0.001269498	0.0473857
NH2-Ac	-0.571832555	-0.76452995	-0.379135162	0.0000000
Am-AcBA	-0.150747773	-0.34344517	0.041949620	0.2308972
CBA-AcBA	0.065668056	-0.12702934	0.258365449	0.9478292
COOH-AcBA	-0.212465097	-0.40516249	-0.019767704	0.0207275
NBA-AcBA.	-0.197611784	-0.39030918	-0.004914391	0.0405243
NH2-AcBA	-0.575477449	-0.76817484	-0.382780056	0.0000000
CBA-Am	0.216415829	0.02371844	0.409113222	0.0171938
COOH-Am	-0.061717323	-0.25441472	0.130980070	0.9612073
NBA-Am	-0.046864011	-0.23956140	0.145833382	0.9904628
NH2-Am	-0.424729675	-0.61742707	-0.232032282	0.0000000
COOH-CBA	-0.278133152	-0.47083055	-0.085435759	0.0006162
NBA-CBA	-0.263279840	-0.45597723	-0.070582447	0.0014642
NH2-CBA	-0.641145504	-0.83384290	-0.448448111	0.0000000
NBA-COOH	0.014853312	-0.17784408	0.207550705	0.9999867
NH2-COOH	-0.363012352	-0.55570975	-0.170314959	0.0000024
NH2-NBA	-0.377865664	-0.57056306	-0.185168271	0.0000008

**Functionalization**	diff	lwr	upr.	p adj
PEG-azide	0.2972747	0.2292891	0.3652603	0

Figure S24. Statistical analyses using multi-way ANOVAs with a Turkey post-hoc test. Three technical replicates were averaged to a single values, and statistics were done on experimental/biological replicates. Statistical analysis on a) soluble peptides cultured with cells on tissue culture plastic, all time points b) soluble peptides cultured with cells on tissue culture plastic, 48 hours, c) concentration study peptides at 48 hours, d) LIAANK peptides at 48 hours, e) IVKVA peptides at 48 hours, f) soluble peptides cultured with cells in PEG hydrogels at 48 hours, g) azide/PEG functionalized peptides cultured with cells at 48 hours.
